# Supplementary material for: Informed consent in cancer clinical care: Perspectives of healthcare professionals on information disclosure at a tertiary institution in Uganda
Source: PLoS One. 2024 Apr 4;19(4):e0301586. doi: 10.1371/journal.pone.0301586 (PMC10994281; doi:10.1371/journal.pone.0301586)
Supplement: S1 File — (DOCX) [file pone.0301586.s002.docx]

| 8/15/2023 8:10 AM | | | | | | | | | | | | | | | | | |
| --- | --- | --- | --- | --- | --- | --- | --- | --- | --- | --- | --- | --- | --- | --- | --- | --- | --- |
| Coding Summary By Code | | | | | | | | | | | | | | | | | |
| **Information disclosure during the consenting process in cancer clinical care: perspectives of healthcare professionals at Uganda Cancer Institute** | | | | | | | | | | | | | | | | | |
| 8/15/2023 8:10 AM | | | | | | | | | | | | | | | | | |
|  | | | **Aggregate** |  | **Classification** |  | **Coverage** |  | **Number Of Coding References** | |  | **Reference Number** |  | **Coded By Initials** |  | **Modified On** |  |
| **Node** | | | | | | | | | | | | | | | | |  |
|  | **Nodes\\Advantages of disclosing immediately\Accountability** | | | | | | | | | | | | | | | |  |
|  | | **Document** | | | | | | | | | | | | | | |  |
|  | | | **Files\\IDI - doctor - UCI -08** | | | | | | | | | | | | | |  |
|  |  |  | No |  |  |  | 0.0117 |  | 1 | |  | | | | | |  |
|  | | |  |  |  |  |  |  |  | |  | | | | | | |
|  | | | | | | | | | | | | 1 |  | AT |  | 7/24/2020 7:59 AM |  |
|  | But in terms of accountability , I think the only thing is about documentation like this patient is starting on this, he or she has been indicated on may be the disease, the diagnosis , the stage of the disease , the prognosis and the treatment intend because most time the treatment intention is permission so it needs to be communicated. | | | | | | | | | | | | | | | |  |
|  |  | | | | | | | | | | | | | | | |  |
|  | **Nodes\\Advantages of disclosing immediately\Enhance access to Palliative care** | | | | | | | | | | | | | | | |  |
|  | | **Document** | | | | | | | | | | | | | | |  |
|  | | | **Files\\IDI - Specialist palliative care_10** | | | | | | | | | | | | | |  |
|  |  |  | No |  |  |  | 0.0160 |  | 1 | |  | | | | | |  |
|  | | |  |  |  |  |  |  |  | |  | | | | | | |
|  | | | | | | | | | | | | 1 |  | AT |  | 7/24/2020 10:00 AM |  |
|  | I think some times they are concerned but it depends on which training you’ve had because of course in palliative care it’s a very important aspect that you must think about the social issues but then there is also a prevailing belief among their workers that ok I’m going to discourage the patients if I give too much information and then they will give up and so what they may look at more is just the person only there and their physical support and they forget everything else that they may be leaving around like how that non-disclosure may impact on the wider social life of the person. | | | | | | | | | | | | | | | |  |
|  |  | | | | | | | | | | | | | | | |  |
|  | | | | | | | | | | | | | | | | | |
|  | | | | | | | | | | | | | | | | | |
| Reports\\Coding Summary By Code Report | | | | | | | | | | Page 1 of 117 | | | | | | | |
| 8/15/2023 8:10 AM | | | | | | | | | | | | | | | | | |
|  | | | **Aggregate** |  | **Classification** |  | **Coverage** |  | **Number Of Coding References** | |  | **Reference Number** |  | **Coded By Initials** |  | **Modified On** |  |
|  | **Nodes\\Advantages of disclosing immediately\enhances psychosocial support from family members** | | | | | | | | | | | | | | | |  |
|  | | **Document** | | | | | | | | | | | | | | |  |
|  | | | **Files\\IDI_ Nurse _UCI_02** | | | | | | | | | | | | | |  |
|  |  |  | No |  |  |  | 0.0109 |  | 1 | |  | | | | | |  |
|  | | |  |  |  |  |  |  |  | |  | | | | | | |
|  | | | | | | | | | | | | 1 |  | AT |  | 7/26/2020 9:42 AM |  |
|  | If you disclose and even the family members are aware they will support financially because at one point the medicine will not be there but you’ve never disclosed to me that you have this disease which needs a lot of money. You’re telling me now that I don’t have drugs at least if you had disclosed to me then if you come to me then I know so it is good to disclose. For financial support. | | | | | | | | | | | | | | | |  |
|  |  | | | | | | | | | | | | | | | |  |
|  | | | **Files\\IDI Nurse -_UCI_04** | | | | | | | | | | | | | |  |
|  |  |  | No |  |  |  | 0.0169 |  | 1 | |  | | | | | |  |
|  | | |  |  |  |  |  |  |  | |  | | | | | | |
|  | | | | | | | | | | | | 1 |  | AT |  | 7/26/2020 10:24 AM |  |
|  | Yeah, I think the advantage here is you know its kind of like a way as we are disclosing this information that’s why it’s important, I said in the first place we need to disclose this information when we have the next of kin, we have the care takers. Now socially if I disclose this information it will then use your family members or your loved ones, they actually get to me that actually this may be the time now that we need to be closed to her loved ones. So, we need to be closure to our loved ones in an advantageous way to help in social life. | | | | | | | | | | | | | | | |  |
|  |  | | | | | | | | | | | | | | | |  |
|  | **Nodes\\Advantages of disclosing immediately\Enhances treatment plan** | | | | | | | | | | | | | | | |  |
|  | | **Document** | | | | | | | | | | | | | | |  |
|  | | | **Files\\IDI - doctor - UCI -08** | | | | | | | | | | | | | |  |
|  |  |  | No |  |  |  | 0.0055 |  | 1 | |  | | | | | |  |
|  | | |  |  |  |  |  |  |  | |  | | | | | | |
|  | | | | | | | | | | | | 1 |  | AT |  | 7/24/2020 8:23 AM |  |
|  | Now it is true, it affects them positively and negatively but different patients different plan. To some they receive the treatment and they plan their treatment, | | | | | | | | | | | | | | | |  |
|  |  | | | | | | | | | | | | | | | |  |
|  | | | **Files\\IDI - doctor - UCI_06** | | | | | | | | | | | | | |  |
|  |  |  | No |  |  |  | 0.084 |  | 1 | |  | | | | | |  |
|  | | |  |  |  |  |  |  |  | |  | | | | | | |
|  | | | | | | | | | | | | 1 |  | AT |  | 7/24/2020 8:49 AM |  |
|  | I would go for immediate disclosure because that helps us as clinicians when the patient arrive and comply to the treatment plan which should be started immediately, if it is surgery it is important to be done out of the way if its radiotherapy the earlier we start always the better. | | | | | | | | | | | | | | | |  |
|  |  | | | | | | | | | | | | | | | |  |
|  | | | | | | | | | | | | | | | | | |
| Reports\\Coding Summary By Code Report | | | | | | | | | | Page 2 of 117 | | | | | | | |
| 8/15/2023 8:10 AM | | | | | | | | | | | | | | | | | |
|  | | | **Aggregate** |  | **Classification** |  | **Coverage** |  | **Number Of Coding References** | |  | **Reference Number** |  | **Coded By Initials** |  | **Modified On** |  |
|  | | | **Files\\IDI - Specialist palliative care_UCI 10** | | | | | | | | | | | | | | uci |
|  |  |  | No |  |  |  | 0.087 |  | 1 | |  | | | | | |  |
|  | | |  |  |  |  |  |  |  | |  | | | | | | |
|  | | | | | | | | | | | | 1 |  | AT |  | 7/24/2020 9:46 AM |  |
|  | it is good that they know because then in terms of decision making, treatment it will be easier and therefore it always better to disclose because with that respect of the person’s wishes but if they don’t want to know then you don’t tell them but you give them continuous opportunities because people adjust in different ways. | | | | | | | | | | | | | | | |  |
|  |  | | | | | | | | | | | | | | | |  |
|  | | | **Files\\IDI- Health Educator- UCI-09** | | | | | | | | | | | | | |  |
|  |  |  | No |  |  |  | 0.082 |  | 1 | |  | | | | | |  |
|  | | |  |  |  |  |  |  |  | |  | | | | | | |
|  | | | | | | | | | | | | 1 |  | AT |  | 7/25/2020 9:41 PM |  |
|  | Two: if you sink in to treatment, if you don’t disclose the treatment plan and what the patients will be going through then adherence will be a problem because this doesn’t understand why they are giving him this medicine, how this medicine acts, the care now will be tampered with, look in to the quality of life and care, you have not told this person that this type of treatment will no longer work for you, we are going this other alternative, palliative care now if you don’t disclose at this point, you don’t engage them in providing other care, supportive care to this person. | | | | | | | | | | | | | | | |  |
|  |  | | | | | | | | | | | | | | | |  |
|  | | | **Files\\IDI_ Nurse - _UCI_07** | | | | | | | | | | | | | |  |
|  |  |  | No |  |  |  | 0.0226 |  | 3 | |  | | | | | |  |
|  | | |  |  |  |  |  |  |  | |  | | | | | | |
|  | | | | | | | | | | | | 1 |  | AT |  | 7/26/2020 8:26 AM |  |
|  | Yes, you have to give treatment and the whole treatment plan because usually we have the surgery so how are going to work on a patient whom you’ve not disclosed to about their disease. It is usually immediate. | | | | | | | | | | | | | | | |  |
|  |  | | | | | | | | | | | | | | | |  |
|  | | | | | | | | | | | | 2 |  | AT |  | 7/26/2020 8:27 AM |  |
|  | For immediate disclosure the advantages would be that it gives a patient time to take in the bad news because when they go back, they have to really think about it, it also helps the physician to plan earlier and better and that gives us better outcomes. | | | | | | | | | | | | | | | |  |
|  |  | | | | | | | | | | | | | | | |  |
|  | | | | | | | | | | | | 3 |  | AT |  | 7/26/2020 8:29 AM |  |
|  | In delaying to disclose? definitely every thing is going to be delayed. The treatment plan is going to be delayed, accepting information is going to be delayed, everything is going to be delayed. | | | | | | | | | | | | | | | |  |
|  |  | | | | | | | | | | | | | | | |  |
|  | | | **Files\\IDI_ Social worker _UCI_03** | | | | | | | | | | | | | |  |
|  |  |  | No |  |  |  | 0.0271 |  | 3 | |  | | | | | |  |
|  | | |  |  |  |  |  |  |  | |  | | | | | | |
|  | | | | | | | | | | | | 1 |  | AT |  | 7/26/2020 8:53 AM |  |
|  | In my opinion as a social worker, I would prefer that the diagnosis is disclosed early, immediately they have seen the results of the patient, they have done all other tests for the patient and they want to initiate treatment for the patient so that the patient can now be able to ask questions | | | | | | | | | | | | | | | |  |
|  |  | | | | | | | | | | | | | | | |  |
|  | | | | | | | | | | | | 2 |  | AT |  | 7/26/2020 8:53 AM |  |
|  | You know when you have not disclosed something to the patient, what questions do you expect the patient to ask, you may think they have understood yet not, they may need to ask questions but about what they don’t know. | | | | | | | | | | | | | | | |  |
|  |  | | | | | | | | | | | | | | | |  |
|  | | | | | | | | | | | | | | | | | |
| Reports\\Coding Summary By Code Report | | | | | | | | | | Page 3 of 117 | | | | | | | |
| 8/15/2023 8:10 AM | | | | | | | | | | | | | | | | | |
|  | | | **Aggregate** |  | **Classification** |  | **Coverage** |  | **Number Of Coding References** | |  | **Reference Number** |  | **Coded By Initials** |  | **Modified On** |  |
|  | | | | | | | | | | | | | | | | | |
|  | | | | | | | | | | | | 3 |  | AT |  | 7/26/2020 8:54 AM |  |
|  | Two, if the best practice is when the patient knows their diagnosis an d their disease stages, it will give them confidence to support the family, one to the patient, the patient will also decide for themselves with the encouragement of the staff, counsellor they have all gone through this, it is a process that is involved in the disclosure, the patient needs to be counselled not even the treatment whatever the patient needs to know the detail about her treatment plan, what kind of drug they are going to go through and also the side effects hey might expect, they length of the treatment and what cost they would incur something like that. | | | | | | | | | | | | | | | |  |
|  |  | | | | | | | | | | | | | | | |  |
|  | | | **Files\\IDI Nurse _UCI_04** | | | | | | | | | | | | | |  |
|  |  |  | No |  |  |  | 0.0555 |  | 3 | |  | | | | | |  |
|  | | |  |  |  |  |  |  |  | |  | | | | | | |
|  | | | | | | | | | | | | 1 |  | AT |  | 7/26/2020 10:08 AM |  |
|  | What would be kind of advisable in this is for the benefit of the patient is to have information or anything regarding the patient. It is always good upon us to get to the patient, the patient’s condition and what we have at hand than us not telling the patient or us lying the patient because as you openly tell the patient, you’re ably going to be able to discuss the treatment plan, what is the way forward. | | | | | | | | | | | | | | | |  |
|  |  | | | | | | | | | | | | | | | |  |
|  | | | | | | | | | | | | 2 |  | AT |  | 7/26/2020 10:09 AM |  |
|  | I would really try to encourage the what would be disadvantage depending on the situation of the patient, I think there are instances whereby I need to let you know the disease that you have is curable lets get to it, lets do the needful for the doctor’s instructions follow whatever they tell you, get the treatment and adequately prompt them. Now if your disease is curable or not it is also upon me to actually inform you there and then that the problem that we have is this and this so I think going forward it is not curable but we can make things better and how can we make things better. We can give you some bit of treatment which can enable us move on for a specific period of time. | | | | | | | | | | | | | | | |  |
|  |  | | | | | | | | | | | | | | | |  |
|  | | | | | | | | | | | | 3 |  | AT |  | 7/26/2020 10:12 AM |  |
|  | I think the good part about that is when information has been given to the patients it has enabled us to have a proper follow up of our patients knowing the unit and knowing the things that they get so it greatly benefits a patient in a way that the patient is able to know that I am at the cancer institute. If I have any prescription I have to go to the pharmacy, while I am at the cancer unit if I have this condition, these are the clinicians that I get to see, I have to be getting these and this is where I am able to get this because you’ve already been shown a navigation of the entire campus you know I need geological investigation this is where I go to, I need these drugs this is where I go to. | | | | | | | | | | | | | | | |  |
|  |  | | | | | | | | | | | | | | | |  |
|  | **Nodes\\Advantages of disclosing immediately\Enhances trust as a result of honesty** | | | | | | | | | | | | | | | |  |
|  | | **Document** | | | | | | | | | | | | | | |  |
|  | | | **Files\\IDI - Doctor - _UCI_06** | | | | | | | | | | | | | |  |
|  |  |  | No |  |  |  | 0.0128 |  | 1 | |  | | | | | |  |
|  | | |  |  |  |  |  |  |  | |  | | | | | | |
|  | | | | | | | | | | | | 1 |  | AT |  | 7/24/2020 8:45 AM |  |
|  | But lying has got a disadvantage that eventually the patient is going to know and from my experience I have researched but most of the patients kind of have an idea that what they are facing is very big so they will even look at you and know that you’re not telling them the whole truth and once hat comes in them they will know you’re not being honest with them which is sometimes I think it kills your trust. It undermines trust. | | | | | | | | | | | | | | | |  |
|  |  | | | | | | | | | | | | | | | |  |
|  | | | | | | | | | | | | | | | | | |
| Reports\\Coding Summary By Code Report | | | | | | | | | | Page 4 of 117 | | | | | | | |
| 8/15/2023 8:10 AM | | | | | | | | | | | | | | | | | |
|  | | | **Aggregate** |  | **Classification** |  | **Coverage** |  | **Number Of Coding References** | |  | **Reference Number** |  | **Coded By Initials** |  | **Modified On** |  |
|  | | | **Files\\IDI - Specialist palliative care_ UCI 10** | | | | | | | | | | | | | |  |
|  |  |  | No |  |  |  | 0.0169 |  | 2 | |  | | | | | |  |
|  | | |  |  |  |  |  |  |  | |  | | | | | | |
|  | | | | | | | | | | | | 1 |  | AT |  | 7/24/2020 9:47 AM |  |
|  | it is good that they know because then in terms of decision making, treatment it will be easier and therefore it always better to disclose because with that respect of the person’s wishes but if they don’t want to know then you don’t tell them but you give them continuous opportunities because people adjust in different ways. | | | | | | | | | | | | | | | |  |
|  |  | | | | | | | | | | | | | | | |  |
|  | | | | | | | | | | | | 2 |  | AT |  | 7/24/2020 10:12 AM |  |
|  | Of course it helps a lot, many times people when people have life limiting illnesses that is when they think about their spirituality and therefore knowing if somebody doesn’t really know or he hasn’t got the information you may be doing them a disservice because they may not give it much attention. | | | | | | | | | | | | | | | |  |
|  |  | | | | | | | | | | | | | | | |  |
|  | | | **Files\\IDI_ Social worker _UCI_03** | | | | | | | | | | | | | |  |
|  |  |  | No |  |  |  | 0.0114 |  | 1 | |  | | | | | |  |
|  | | |  |  |  |  |  |  |  | |  | | | | | | |
|  | | | | | | | | | | | | 1 |  | AT |  | 7/26/2020 8:55 AM |  |
|  | The reason why I am asking you to come back or you can decide with the patient, do want to come back or have you understood what I have told you, do you need more information together that means you are having a mutual discussion and understanding and then you’re going to work together but when it is not done you find the patient falling apart that the doctors do not know what to do with the patient because the patient is not supported to understand clearly what is going to happen. | | | | | | | | | | | | | | | |  |
|  |  | | | | | | | | | | | | | | | |  |
|  | | | **Files\\IDI_ Nurse - _UCI_04** | | | | | | | | | | | | | |  |
|  |  |  | No |  |  |  | 0.0115 |  | 1 | |  | | | | | |  |
|  | | |  |  |  |  |  |  |  | |  | | | | | | |
|  | | | | | | | | | | | | 1 |  | AT |  | 7/26/2020 10:07 AM |  |
|  | If you don’t disclose to the patient then at one point another clinician comes to reveal and then tells this patient, actually the last time you came here, this is what you had then it will greatly affect the relationship that you had with your client because the client is going to be like but I heard this, this is what happened, how comes my clinician never informed me. | | | | | | | | | | | | | | | |  |
|  |  | | | | | | | | | | | | | | | |  |
|  | **Nodes\\Advantages of disclosing immediately\Enhnaces decision making** | | | | | | | | | | | | | | | |  |
|  | | **Document** | | | | | | | | | | | | | | |  |
|  | | | **Files\\IDI - Doctor - UCI -08** | | | | | | | | | | | | | |  |
|  |  |  | No |  |  |  | 0.0179 |  | 1 | |  | | | | | |  |
|  | | |  |  |  |  |  |  |  | |  | | | | | | |
|  | | | | | | | | | | | | 1 |  | AT |  | 7/24/2020 8:09 AM |  |
|  | It can help them to make good decisions in case for some instances when you may not have those services here so disclosing to them helps when they have the capacity to go to another hospital or outside like if someone wants a bone marrow transplant and you don’t tell them that for you, you can benefit from bone marrow transplant yet they are able to afford so when you disclose to them is a good thing, the other is people can make their way, they can manage their families such that they can prepare for their life. | | | | | | | | | | | | | | | |  |
|  |  | | | | | | | | | | | | | | | |  |
| Reports\\Coding Summary By Code Report | | | | | | | | | | Page 5 of 117 | | | | | | | |
| 8/15/2023 8:10 AM | | | | | | | | | | | | | | | | | |
|  | | | **Aggregate** |  | **Classification** |  | **Coverage** |  | **Number Of Coding References** | |  | **Reference Number** |  | **Coded By Initials** |  | **Modified On** |  |
|  | | | **Files\\IDI - _ Doctor_UCI_06** | | | | | | | | | | | | | |  |
|  |  |  | No |  |  |  | 0.0059 |  | 1 | |  | | | | | |  |
|  | | |  |  |  |  |  |  |  | |  | | | | | | |
|  | | | | | | | | | | | | 1 |  | AT |  | 7/24/2020 8:43 AM |  |
|  | The advantage yes, you’re able to take decision earlier in as far as what treatment plan, you’re going to follow and also you enable the patient come to terms with the condition earlier than later. | | | | | | | | | | | | | | | |  |
|  |  | | | | | | | | | | | | | | | |  |
|  | | | **Files\\IDI -Specialist palliative care_10** | | | | | | | | | | | | | |  |
|  |  |  | No |  |  |  | 0.0062 |  | 1 | |  | | | | | |  |
|  | | |  |  |  |  |  |  |  | |  | | | | | | |
|  | | | | | | | | | | | | 1 |  | AT |  | 7/24/2020 10:12 AM |  |
|  | So, at the end when they realize that may be I should have done this, I should have been more spiritual then they have little time they panic because they never had the opportunity to know that that their prognosis was not good. | | | | | | | | | | | | | | | |  |
|  |  | | | | | | | | | | | | | | | |  |
|  | | | **Files\\IDI- - Health Educator- UCI-09** | | | | | | | | | | | | | |  |
|  |  |  | No |  |  |  | 0.0116 |  | 4 | |  | | | | | |  |
|  | | |  |  |  |  |  |  |  | |  | | | | | | |
|  | | | | | | | | | | | | 1 |  | AT |  | 7/25/2020 9:39 PM |  |
|  | Immediate disclosure, one: when we talk of realized care, we want patients to participate from decision making in to their care which disclosure is one way of opening to this person and involving this person in his or her care | | | | | | | | | | | | | | | |  |
|  |  | | | | | | | | | | | | | | | |  |
|  | | | | | | | | | | | | 2 |  | AT |  | 7/25/2020 9:40 PM |  |
|  | so when you disclose and the patient understands what they are supposed to do and what the hospital can do that’s when patients do their part so for them to get to understand that better it comes to disclosure so if disclosure is not made you cannot so to all those other aspects, there is no rationale for doing that, that is one big thing. | | | | | | | | | | | | | | | |  |
|  |  | | | | | | | | | | | | | | | |  |
|  | | | | | | | | | | | | 3 |  | AT |  | 7/25/2020 9:40 PM |  |
|  | Looking in to disclosure at different point initial disclosure of diagnosis involves the person in the care from deciding. | | | | | | | | | | | | | | | |  |
|  |  | | | | | | | | | | | | | | | |  |
|  | | | | | | | | | | | | 4 |  | AT |  | 7/25/2020 9:42 PM |  |
|  | I have said it both ways, this patient cannot take care of himself as required because they didn’t know disclosure means I have been told. | | | | | | | | | | | | | | | |  |
|  |  | | | | | | | | | | | | | | | |  |
|  | | | **Files\\IDI_ Social worker_UCI_03** | | | | | | | | | | | | | |  |
|  |  |  | No |  |  |  | 0.0229 |  | 3 | |  | | | | | |  |
|  | | |  |  |  |  |  |  |  | |  | | | | | | |
|  | | | | | | | | | | | | 1 |  | AT |  | 7/26/2020 8:43 AM |  |
|  | I think one would be that the family will be more involved in trying to contribute to patient’s treatment because they understand what they are getting through. | | | | | | | | | | | | | | | |  |
|  |  | | | | | | | | | | | | | | | |  |
|  | | | | | | | | | | | | 2 |  | AT |  | 7/26/2020 8:45 AM |  |
|  | If I am to give an example there was a patient who came to us yesterday she is from a rural place they have six cows and this lady has sold three cows yet the patient’s condition is advancing but for them because they don’t know the stage of the disease and how much it has affected the patient they are still planning to sell their cows until you know yet it is something that is perhaps not going to heal and just needs now to allow the patient to move through total palliative care so they are wasting more and more resources and that is the disadvantage bit of it. | | | | | | | | | | | | | | | |  |
|  |  | | | | | | | | | | | | | | | |  |
| Reports\\Coding Summary By Code Report | | | | | | | | | | Page 6 of 117 | | | | | | | |
| 8/15/2023 8:10 AM | | | | | | | | | | | | | | | | | |
|  | | | **Aggregate** |  | **Classification** |  | **Coverage** |  | **Number Of Coding References** | |  | **Reference Number** |  | **Coded By Initials** |  | **Modified On** |  |
|  | | | | | | | | | | | | | | | | | |
|  | | | | | | | | | | | | 3 |  | AT |  | 7/26/2020 8:54 AM |  |
|  | Because if they put all this together, they will have their own stand as a family and if the patient do not want at that time to receive the information, give the patient a chance a day or two to come back either by themselves or with somebody. | | | | | | | | | | | | | | | |  |
|  |  | | | | | | | | | | | | | | | |  |
|  | | | **Files\\IDI_ Nurse_UCI_04** | | | | | | | | | | | | | |  |
|  |  |  | No |  |  |  | 0.0320 |  | 1 | |  | | | | | |  |
|  | | |  |  |  |  |  |  |  | |  | | | | | | |
|  | | | | | | | | | | | | 1 |  | AT |  | 7/26/2020 10:23 AM |  |
|  | In the disclosure it is actually good for them because sometimes it enables to make decisions because these conditions that they have, they have to prepare themselves economically as well because it is not a one day’s condition its for a given period of time that your going to have these conditions, sometimes you may need some drugs or something just in case something is out of height or you’re able to get it or may be locally with in the unit and it needs you to go and buy, you need some funding but now what has happened is that given the condition, they are kind of lost out their way they are not able to strongly work and make money so they are not being able to get the money themselves it greatly affects, they are not able to finance their children going to school. Some of these men or women are the bread winners of the home but if he or she is broken down, the disease has already broken them down, they are not able to cater for the family so that is how really this condition gets to them and it really doesn’t do them well. | | | | | | | | | | | | | | | |  |
|  |  |  |  |  |  |  |  |  |  |  |  |  |  |  |  |  |  |
|  |  | | | | | | | | | | | | | | | |  |
|  | | | **Files\\IDI__ Doctor_ UCI_ 05** | | | | | | | | | | | | | |  |
|  |  |  | No |  |  |  | 0.0283 |  | 2 | |  | | | | | |  |
|  | | |  |  |  |  |  |  |  | |  | | | | | | |
|  | | | | | | | | | | | | 1 |  | AT |  | 7/26/2020 10:39 AM |  |
|  | I think its very important why do I say that, 90% of the cancer patients who come here have been else where and that has implications, the first one is financial, they have spent a lot of money. Secondly, they have a lot of information which they have been given both true but also false but thirdly patients have been on different concoctions as well trying to get remedies either to their pain or to their swelling or to whatever problem which they have. So, I think it is very important when you come and we try to know, what are your beliefs and what are also my beliefs so that we can reach an understanding as well | | | | | | | | | | | | | | | |  |
|  |  | | | | | | | | | | | | | | | |  |
|  | | | | | | | | | | | | 2 |  | AT |  | 7/26/2020 10:39 AM |  |
|  | But also, to know what is false because cancer in this country has a lot of myths and beliefs attached to it. And thirdly in relation to the first point which I made financially as well, some patients come here thinking or some people delay coming here thinking they are going to ask for a lot of money because they all the treatment which is here is for paying which is not true. The treatment which is here is free of charge unless if it is not available that’s when you have to pay for it. | | | | | | | | | | | | | | | |  |
|  |  | | | | | | | | | | | | | | | |  |
|  | **Nodes\\Advantages of disclosing immediately\Patient owns the prognosis, treatment, and disease itself** | | | | | | | | | | | | | | | |  |
|  | | **Document** | | | | | | | | | | | | | | |  |
|  | | | **Files\\IDI_ Social worker_UCI_03** | | | | | | | | | | | | | |  |
|  |  |  | No |  |  |  | 0.0280 |  | 2 | |  | | | | | |  |
|  | | |  |  |  |  |  |  |  | |  | | | | | | |
|  | | | | | | | | | | | | 1 |  | AT |  | 7/26/2020 8:44 AM |  |
|  | Two it gives the patient confidence to go through the treatment, the side effects and what because they are assured of either positive outcome quite often because if they say that the disease is like advanced and then you have given the patient treatment and their information that this might not be something that the medical team would do much more about it and the decision to start treatment or just stay or palliative care would be from the patient’s perceptive and three, it would help the patient to plan for their resources that they have. | | | | | | | | | | | | | | | |  |
|  |  | | | | | | | | | | | | | | | |  |
| Reports\\Coding Summary By Code Report | | | | | | | | | | Page 7 of 117 | | | | | | | |
| 8/15/2023 8:10 AM | | | | | | | | | | | | | | | | | |
|  | | | **Aggregate** |  | **Classification** |  | **Coverage** |  | **Number Of Coding References** | |  | **Reference Number** |  | **Coded By Initials** |  | **Modified On** |  |
|  | | | | | | | | | | | | | | | | | |
|  | | | | | | | | | | | | 2 |  | AT |  | 7/26/2020 8:54 AM |  |
|  | Two, if the best practice is when the patient knows their diagnosis an d their disease stages, it will give them confidence to support the family, one to the patient, the patient will also decide for themselves with the encouragement of the staff, counsellor they have all gone through this, it is a process that is involved in the disclosure, the patient needs to be counselled not even the treatment whatever the patient needs to know the detail about her treatment plan, what kind of drug they are going to go through and also the side effects hey might expect, they length of the treatment and what cost they would incur something like that. | | | | | | | | | | | | | | | |  |
|  |  | | | | | | | | | | | | | | | |  |
|  | | | **Files\\IDI_ Nurse_UCI_02** | | | | | | | | | | | | | |  |
|  |  |  | No |  |  |  | 0.0106 |  | 1 | |  | | | | | |  |
|  | | |  |  |  |  |  |  |  | |  | | | | | | |
|  | | | | | | | | | | | | 1 |  | AT |  | 7/26/2020 9:24 AM |  |
|  | Yes, it is helpful if it is communicated well, immediate disclosure has a problem but also even the communication in our key because when you disclose to them, they will not have so much expectations, their expectations that they have, even if a patient changes condition they will know that but the doctor had told me this prognosis is poor so I think it also helps very well. | | | | | | | | | | | | | | | |  |
|  |  | | | | | | | | | | | | | | | |  |
|  | **Nodes\\Advantages of disclosing immediately\plan for life** | | | | | | | | | | | | | | | |  |
|  | | **Document** | | | | | | | | | | | | | | |  |
|  | | | **Files\\IDI - - Doctor- UCI -08** | | | | | | | | | | | | | |  |
|  |  |  | No |  |  |  | 0.0404 |  | 4 | |  | | | | | |  |
|  | | |  |  |  |  |  |  |  | |  | | | | | | |
|  | | | | | | | | | | | | 1 |  | AT |  | 7/24/2020 8:09 AM |  |
|  | It can help them to make good decisions in case for some instances when you may not have those services here so disclosing to them helps when they have the capacity to go to another hospital or outside like if someone wants a bone marrow transplant and you don’t tell them that for you, you can benefit from bone marrow transplant yet they are able to afford so when you disclose to them is a good thing, the other is people can make their way, they can manage their families such that they can prepare for their life. | | | | | | | | | | | | | | | |  |
|  |  | | | | | | | | | | | | | | | |  |
|  | | | | | | | | | | | | 2 |  | AT |  | 7/24/2020 8:23 AM |  |
|  | they prepare money for their care, | | | | | | | | | | | | | | | |  |
|  |  | | | | | | | | | | | | | | | |  |
|  | | | | | | | | | | | | 3 |  | AT |  | 7/24/2020 8:25 AM |  |
|  | I think the only for us to do is to tell them if the drug is not here you need to plan to have some money for your treatment to go well, that is the part of their economic thing that we talk about. The money needed for treatment, investigation that kind of staff, we may not go in to how they need to raise that money, we don’t go in to that. | | | | | | | | | | | | | | | |  |
|  |  | | | | | | | | | | | | | | | |  |
|  | | | | | | | | | | | | 4 |  | AT |  | 7/24/2020 8:29 AM |  |
|  | Yes, because the information they give will not only help the patient but will also help to plan and sometimes they are like so and so also this thing you are saying is a good thing and they like it, I have not seen someone who doesn’t like it and majority of them want to. | | | | | | | | | | | | | | | |  |
|  |  | | | | | | | | | | | | | | | |  |
|  | | | | | | | | | | | | | | | | | |
| Reports\\Coding Summary By Code Report | | | | | | | | | | Page 8 of 117 | | | | | | | |
| 8/15/2023 8:10 AM | | | | | | | | | | | | | | | | | |
|  | | | **Aggregate** |  | **Classification** |  | **Coverage** |  | **Number Of Coding References** | |  | **Reference Number** |  | **Coded By Initials** |  | **Modified On** |  |
|  | | | **Files\\IDI -Specialist palliative care_10** | | | | | | | | | | | | | |  |
|  |  |  | No |  |  |  | 0.0305 |  | 2 | |  | | | | | |  |
|  | | |  |  |  |  |  |  |  | |  | | | | | | |
|  | | | | | | | | | | | | 1 |  | AT |  | 7/24/2020 9:58 AM |  |
|  | For us when you disclose to someone, then you give them chance to plan their future life, if they know their diagnosis and prognosis, then they know that I should put my family affairs in place, may be if I have a huge family I have to plan for it , then also planning treatments, what resources do I need for treatment even I have this diagnosis, it helps them plan for treatments and generally also plan businesses because if you know that your prognosis is not good, you know how to mobilize our businesses knowing that my time is not long I shouldn’t venture in to certain businesses so I think it has advantages in that way, it helps them sort their affairs not to leave the family in wrangles. | | | | | | | | | | | | | | | |  |
|  |  | | | | | | | | | | | | | | | |  |
|  | | | | | | | | | | | | 2 |  | AT |  | 7/24/2020 10:10 AM |  |
|  | The economic decisions based on the information we have someone may plan for their finances may be how to mobilize funds for treatment because you have to think about where am I going to get money to support me but also how do I make a will to this distribute my property, how do I prepare for my children. If you have anything that’s it, sometimes even you may not have any thing but at least if someone has their property it helps. | | | | | | | | | | | | | | | |  |
|  |  | | | | | | | | | | | | | | | |  |
|  | | | **Files\\IDI_ Social worker_UCI_03** | | | | | | | | | | | | | |  |
|  |  |  | No |  |  |  | 0.0050 |  | 1 | |  | | | | | |  |
|  | | |  |  |  |  |  |  |  | |  | | | | | | |
|  | | | | | | | | | | | | 1 |  | AT |  | 7/26/2020 8:50 AM |  |
|  | Yes, there are advantages and disadvantages there so if you delay to give them that information that they do not know they might think thus thing is curable yet it is advanced, that is for those that are advanced. | | | | | | | | | | | | | | | |  |
|  |  | | | | | | | | | | | | | | | |  |
|  | | | **Files\\IDI_ Nurse_UCI_02** | | | | | | | | | | | | | |  |
|  |  |  | No |  |  |  | 0.0269 |  | 2 | |  | | | | | |  |
|  | | |  |  |  |  |  |  |  | |  | | | | | | |
|  | | | | | | | | | | | | 1 |  | AT |  | 7/26/2020 9:42 AM |  |
|  | They all have impacts to the family because if I do it immediately, if I disclose immediately your prognosis, you prepare your self and the family members to get to know that is if you disclose to the family members and they will get support from the family members, if I delay family members may not give support they will think it is casual so you find the patient comes alone because there was no disclosure at all but at least if you disclose immediately may be the patient will go back and tell the family members this is what I am and will call the family members too, so it has both positive and negative impacts. | | | | | | | | | | | | | | | |  |
|  |  | | | | | | | | | | | | | | | |  |
|  | | | | | | | | | | | | 2 |  | AT |  | 7/26/2020 9:46 AM |  |
|  | Also, it can prepare their families timely, mummy is sick in case of any thing if she goes away at least you were prepared, we are told that our prognosis was not good. By the way you tell them, others will go and pray, she was told it cannot be cured but for us lets pray and then they pray with the patient if they have their belief. | | | | | | | | | | | | | | | |  |
|  |  | | | | | | | | | | | | | | | |  |
|  | | | | | | | | | | | | | | | | | |
|  | | | | | | | | | | | | | | | | | |
| Reports\\Coding Summary By Code Report | | | | | | | | | | Page 9 of 117 | | | | | | | |
| 8/15/2023 8:10 AM | | | | | | | | | | | | | | | | | |
|  | | | **Aggregate** |  | **Classification** |  | **Coverage** |  | **Number Of Coding References** | |  | **Reference Number** |  | **Coded By Initials** |  | **Modified On** |  |
|  | | | **Files\\IDI_ _ Nurse_UCI_04** | | | | | | | | | | | | | |  |
|  |  |  | No |  |  |  | 0.0183 |  | 1 | |  | | | | | |  |
|  | | |  |  |  |  |  |  |  | |  | | | | | | |
|  | | | | | | | | | | | | 1 |  | AT |  | 7/26/2020 10:09 AM |  |
|  | You’re also give the patient an opportunity to plan for him or her self what it is what if is the palliative case. Then if it is a palliative case, what do I have to do but if you don’t disclose information to this patient, it is not going to know that it is a palliative case, it is not going enable him organize himself to make proper decisions for himself because this patient needs holistic care maybe you may realise the time when this patient has to make amends for his or her spiritual life or his social life so you’re not really enabling us once we don’t disclose information to patients. | | | | | | | | | | | | | | | |  |
|  |  | | | | | | | | | | | | | | | |  |
|  | **Nodes\\Advantages of disclosing immediately\Promotes access to conventional cancer medicine** | | | | | | | | | | | | | | | |  |
|  | | **Document** | | | | | | | | | | | | | | |  |
|  | | | **Files\\IDI_ Nurse_UCI_07** | | | | | | | | | | | | | |  |
|  |  |  | No |  |  |  | 0.0214 |  | 2 | |  | | | | | |  |
|  | | |  |  |  |  |  |  |  | |  | | | | | | |
|  | | | | | | | | | | | | 1 |  | AT |  | 7/26/2020 8:24 AM |  |
|  | However we disclose to the patients immediately, there is no delay in disclosure because at the end of the day you’re going to give them treatment but what kind of treatment are you giving and which kind of disease is it treating, it is usually immediate to the patients. | | | | | | | | | | | | | | | |  |
|  |  | | | | | | | | | | | | | | | |  |
|  | | | | | | | | | | | | 2 |  | AT |  | 7/26/2020 8:29 AM |  |
|  | You see, sometimes where they go they try all kinds of treatment and its not working. The herbal medicine that is one, the most dangerous one they take all their time taking all the herbal medicine and when they see it is not working then they decide to come back and then the other thing usually is that you find that some patients or attendants are ones that advise these patients that no you see I was badly off but when I started treatment I became better so that tends to push them back in to the system. | | | | | | | | | | | | | | | |  |
|  |  | | | | | | | | | | | | | | | |  |
|  | **Nodes\\Advantages of disclosing immediately\Promotes adherence to clinic routine clinic appointment** | | | | | | | | | | | | | | | |  |
|  | | **Document** | | | | | | | | | | | | | | |  |
|  | | | **Files\\IDI_ Nurse_UCI_02** | | | | | | | | | | | | | |  |
|  |  |  | No |  |  |  | 0.044 |  | 1 | |  | | | | | |  |
|  | | |  |  |  |  |  |  |  | |  | | | | | | |
|  | | | | | | | | | | | | 1 |  | AT |  | 7/26/2020 9:24 AM |  |
|  | If you delay disclosure many people will come up that is if it is bad disease prognosis and again disclosure, the patients will come for their appointments. | | | | | | | | | | | | | | | |  |
|  |  | | | | | | | | | | | | | | | |  |
|  | | | | | | | | | | | | | | | | | |
| Reports\\Coding Summary By Code Report | | | | | | | | | | Page 10 of 117 | | | | | | | |
| 8/15/2023 8:10 AM | | | | | | | | | | | | | | | | | |
|  | | | **Aggregate** |  | **Classification** |  | **Coverage** |  | **Number Of Coding References** | |  | **Reference Number** |  | **Coded By Initials** |  | **Modified On** |  |
|  | **Nodes\\Advantages of disclosing immediately\Promotes adherence to treatment** | | | | | | | | | | | | | | | |  |
|  | | **Document** | | | | | | | | | | | | | | |  |
|  | | | **Files\\IDI- - Health Educator- UCI-09** | | | | | | | | | | | | | |  |
|  |  |  | No |  |  |  | 0.082 |  | 1 | |  | | | | | |  |
|  | | |  |  |  |  |  |  |  | |  | | | | | | |
|  | | | | | | | | | | | | 1 |  | AT |  | 7/25/2020 9:41 PM |  |
|  | Two: if you sink in to treatment, if you don’t disclose the treatment plan and what the patients will be going through then adherence will be a problem because this doesn’t understand why they are giving him this medicine, how this medicine acts, the care now will be tampered with, look in to the quality of life and care, you have not told this person that this type of treatment will no longer work for you, we are going this other alternative, palliative care now if you don’t disclose at this point, you don’t engage them in providing other care, supportive care to this person. | | | | | | | | | | | | | | | |  |
|  |  | | | | | | | | | | | | | | | |  |
|  | | | **Files\\IDI_ Counselor_UCI_01** | | | | | | | | | | | | | |  |
|  |  |  | No |  |  |  | 0.0123 |  | 1 | |  | | | | | |  |
|  | | |  |  |  |  |  |  |  | |  | | | | | | |
|  | | | | | | | | | | | | 1 |  | AT |  | 7/25/2020 11:00 PM |  |
|  | One of the advantages is the patient will understand the situation he is in which is better you know when you understand as a patient then you’re able to find a solution so if patients go immediately, they are diagnosed they will understand and then they are able to adhere well to treatment because they know why they are on care. | | | | | | | | | | | | | | | |  |
|  |  | | | | | | | | | | | | | | | |  |
|  | | | **Files\\IDI_ Nurse_UCI_02** | | | | | | | | | | | | | |  |
|  |  |  | No |  |  |  | 0.0200 |  | 1 | |  | | | | | |  |
|  | | |  |  |  |  |  |  |  | |  | | | | | | |
|  | | | | | | | | | | | | 1 |  | AT |  | 7/26/2020 9:22 AM |  |
|  | Not so hopeful because it is a chronic illness that sometimes you have to treat them then you expect some relapse, we are not going to treat you 100% because I think what guides in the stage will help them disclose better. Also to guides is that if you see some one that they have to take their treatment and their distance is far so you can disclose to them such that they are serious with their treatment that we are aiming cure now I come back to the other point if you’re aiming cure let me disclose to you that this disease is curable and this is its prognosis, 90%we treat are cured so if put in some luxury you might loose it and then for you you’re not counted in the other part that we are aiming cure. | | | | | | | | | | | | | | | |  |
|  |  | | | | | | | | | | | | | | | |  |
|  | | | | | | | | | | | | | | | | | |
|  | | | | | | | | | | | | | | | | | |
|  | | | | | | | | | | | | | | | | | |
| Reports\\Coding Summary By Code Report | | | | | | | | | | Page 11 of 117 | | | | | | | |
| 8/15/2023 8:10 AM | | | | | | | | | | | | | | | | | |
|  | | | **Aggregate** |  | **Classification** |  | **Coverage** |  | **Number Of Coding References** | |  | **Reference Number** |  | **Coded By Initials** |  | **Modified On** |  |
|  | **Nodes\\Advantages of disclosing immediately\Relieves burden** | | | | | | | | | | | | | | | |  |
|  | | **Document** | | | | | | | | | | | | | | |  |
|  | | | **Files\\IDI- - Health Educator- UCI-09** | | | | | | | | | | | | | |  |
|  |  |  | No |  |  |  | 0.0039 |  | 1 | |  | | | | | |  |
|  | | |  |  |  |  |  |  |  | |  | | | | | | |
|  | | | | | | | | | | | | 1 |  | AT |  | 7/25/2020 9:32 PM |  |
|  | Because in directives in relation to care like such care in cancer, it gives the patient some relief to communicate part of their will while they are in care, they communicate who will take charge of me, who will decide for me when I cant talk or do this or what do I opt to do? | | | | | | | | | | | | | | | |  |
|  |  | | | | | | | | | | | | | | | |  |
|  | **Nodes\\Advantages of disclosing immediately\Removes myths and misconceptions about HIV** | | | | | | | | | | | | | | | |  |
|  | | **Document** | | | | | | | | | | | | | | |  |
|  | | | **Files\\IDI_ Social worker_UCI_03** | | | | | | | | | | | | | |  |
|  |  |  | No |  |  |  | 0.0161 |  | 1 | |  | | | | | |  |
|  | | |  |  |  |  |  |  |  | |  | | | | | | |
|  | | | | | | | | | | | | 1 |  | AT |  | 7/26/2020 8:52 AM |  |
|  | Not disclosing causes more misconceptions or myths about cancer because whoever leaves here goes out and explains it in their own way but it better when you explain the truth to the patient and they also from the symptoms and whatever that led to their disease then perhaps when they go back, they are able to help others in the community saying that what is manifesting in you seems to be similar to what my ABCD patient went through can you go and see the doctor, ask or test early because now this one would have known the testing early is the better way to go other than testing late because I believe when patients come here doctors always emphasis the benefits of testing early. | | | | | | | | | | | | | | | |  |
|  |  | | | | | | | | | | | | | | | |  |
|  | **Nodes\\Advantages of disclosing immediately\Restores hope** | | | | | | | | | | | | | | | |  |
|  | | **Document** | | | | | | | | | | | | | | |  |
|  | | | **Files\\IDI_ Nurse_UCI_02** | | | | | | | | | | | | | |  |
|  |  |  | No |  |  |  | 0.0344 |  | 2 | |  | | | | | |  |
|  | | |  |  |  |  |  |  |  | |  | | | | | | |
|  | | | | | | | | | | | | 1 |  | AT |  | 7/26/2020 9:23 AM |  |
|  | Advantages of knowing some body’s prognosis, if the stage is early and someone is told that we are aiming at cure they have hopes, they will never loose hope because they were told the prognosis is good. If some one has come at a late stage then they tell them that the prognosis is bad sometimes they lose hope and they will never carry on with the treatment and you know for us in life we are not the ones who are going to save the life. So, it has two things, you can be disclosed to in a bad way and then you lose hope. From the practice I see here, people lose hope, they even get tired of the treatment and start telling them first of all your disease is stage four, prognosis is not good, second of all we are going to put you on the drug which is very strong so it will heat so those are two things , my disease is not going to prognose properly then again they have to add me so someone will lose hope and then they will be like I think I better leave it. However, when you give nice news that your prognosis is good they will encouraged to come for treatment and finish it. | | | | | | | | | | | | | | | |  |
|  |  |  |  |  |  |  |  |  |  |  |  |  |  |  |  |  |  |
|  |  | | | | | | | | | | | | | | | |  |
| Reports\\Coding Summary By Code Report | | | | | | | | | | Page 12 of 117 | | | | | | | |
| 8/15/2023 8:10 AM | | | | | | | | | | | | | | | | | |
|  | | | **Aggregate** |  | **Classification** |  | **Coverage** |  | **Number Of Coding References** | |  | **Reference Number** |  | **Coded By Initials** |  | **Modified On** |  |
|  | | | | | | | | | | | | | | | | | |
|  | | | | | | | | | | | | 2 |  | AT |  | 7/26/2020 9:27 AM |  |
|  | When you tell them that they will be ok yet you know they are going to die, it gives them some hope and they go on with their treatment. | | | | | | | | | | | | | | | |  |
|  |  | | | | | | | | | | | | | | | |  |
|  | **Nodes\\Advantages of disclosing immediately\Sign of best medical practice** | | | | | | | | | | | | | | | |  |
|  | | **Document** | | | | | | | | | | | | | | |  |
|  | | | **Files\\IDI_ Nurse_UCI_02** | | | | | | | | | | | | | |  |
|  |  |  | No |  |  |  | 0.045 |  | 1 | |  | | | | | |  |
|  | | |  |  |  |  |  |  |  | |  | | | | | | |
|  | | | | | | | | | | | | 1 |  | AT |  | 7/26/2020 9:28 AM |  |
|  | It is a bad practice to us because you will not have done your profession very well because you’re supposed to disclose, you will have hindered them information. | | | | | | | | | | | | | | | |  |
|  |  | | | | | | | | | | | | | | | |  |
|  | | | **Files\\IDI_ _ Nurse_UCI_04** | | | | | | | | | | | | | |  |
|  |  |  | No |  |  |  | 0.0053 |  | 1 | |  | | | | | |  |
|  | | |  |  |  |  |  |  |  | |  | | | | | | |
|  | | | | | | | | | | | | 1 |  | AT |  | 7/26/2020 10:06 AM |  |
|  | No, I think its kind of an error in the practice because there is no reason why, it negatively impacts as I talked about it earlier. It negatively impacts on the patient. | | | | | | | | | | | | | | | |  |
|  |  | | | | | | | | | | | | | | | |  |
|  | | | **Files\\IDI__ Doctor_ UCI_ 05** | | | | | | | | | | | | | |  |
|  |  |  | No |  |  |  | 0.044 |  | 1 | |  | | | | | |  |
|  | | |  |  |  |  |  |  |  | |  | | | | | | |
|  | | | | | | | | | | | | 1 |  | AT |  | 7/26/2020 10:43 AM |  |
|  | To me the best practice is give information immediately but just try to reinforce and seek understanding. On each visit try to reinforce because these patients need support. | | | | | | | | | | | | | | | |  |
|  |  | | | | | | | | | | | | | | | |  |
|  | **Nodes\\Advantages of disclosing immediately\Stregthens faith and spirituality** | | | | | | | | | | | | | | | |  |
|  | | **Document** | | | | | | | | | | | | | | |  |
|  | | | **Files\\IDI - - Doctor- UCI -08** | | | | | | | | | | | | | |  |
|  |  |  | No |  |  |  | 0.094 |  | 2 | |  | | | | | |  |
|  | | |  |  |  |  |  |  |  | |  | | | | | | |
|  | | | | | | | | | | | | 1 |  | AT |  | 7/24/2020 8:26 AM |  |
|  | Their faith is impacted on positively, many who are not serious become more serious. | | | | | | | | | | | | | | | |  |
|  |  | | | | | | | | | | | | | | | |  |
| Reports\\Coding Summary By Code Report | | | | | | | | | | Page 13 of 117 | | | | | | | |
| 8/15/2023 8:10 AM | | | | | | | | | | | | | | | | | |
|  | | | **Aggregate** |  | **Classification** |  | **Coverage** |  | **Number Of Coding References** | |  | **Reference Number** |  | **Coded By Initials** |  | **Modified On** |  |
|  | | | | | | | | | | | | | | | | | |
|  | | | | | | | | | | | | 2 |  | AT |  | 7/24/2020 8:26 AM |  |
|  | And many who didn’t have may now get, they get born again and their family of course the family is in two ways some will impact positive like people will come to care for them like that, | | | | | | | | | | | | | | | |  |
|  |  | | | | | | | | | | | | | | | |  |
|  | | | **Files\\IDI - _ Doctor_UCI_06** | | | | | | | | | | | | | |  |
|  |  |  | No |  |  |  | 0.0035 |  | 1 | |  | | | | | |  |
|  | | |  |  |  |  |  |  |  | |  | | | | | | |
|  | | | | | | | | | | | | 1 |  | AT |  | 7/24/2020 9:08 AM |  |
|  | Yes, they generally become more prayerful that’s what you observe then they become more prayerful and more religious. | | | | | | | | | | | | | | | |  |
|  |  | | | | | | | | | | | | | | | |  |
|  | | | **Files\\IDI_ _ Nurse_UCI_04** | | | | | | | | | | | | | |  |
|  |  |  | No |  |  |  | 0.0193 |  | 1 | |  | | | | | |  |
|  | | |  |  |  |  |  |  |  | |  | | | | | | |
|  | | | | | | | | | | | | 1 |  | AT |  | 7/26/2020 10:23 AM |  |
|  | Yeah, it does because its given them room, some of them as they hear of this diagnosis the first thing that gets in to their mind is I’m going to die, when you’re going to die the only thing remember is what is relationship with my God and now the thing is the next person will always want to communicate to is their God. In one way or the other the ones who have always been strong in the spiritual world will always want to be closer to their God, the ones who have been weak spiritually will now start looking for how can I strengthen myself, how can I streamline myself spiritually because I know in a while I am going to die. | | | | | | | | | | | | | | | |  |
|  |  | | | | | | | | | | | | | | | |  |
|  | **Nodes\\Advantages of disclosing immediately\Strengthen patients rights to information** | | | | | | | | | | | | | | | |  |
|  | | **Document** | | | | | | | | | | | | | | |  |
|  | | | **Files\\IDI- - Health Educator- UCI-09** | | | | | | | | | | | | | |  |
|  |  |  | No |  |  |  | 0.0062 |  | 1 | |  | | | | | |  |
|  | | |  |  |  |  |  |  |  | |  | | | | | | |
|  | | | | | | | | | | | | 1 |  | AT |  | 7/25/2020 9:33 PM |  |
|  | It is a right of a patient to know information, it is the patient’s right to know their condition and a doctor must not with hold it. The doctor can only withhold such information when he or she feels the patient is not ready to receive that information so patients have a right to information so disclosure has to be done as first as possible but to the benefits of the patients because any other person may want to know about their disease. | | | | | | | | | | | | | | | |  |
|  |  | | | | | | | | | | | | | | | |  |
|  | | | **Files\\IDI_ Nurse_UCI_02** | | | | | | | | | | | | | |  |
|  |  |  | No |  |  |  | 0.0243 |  | 3 | |  | | | | | |  |
|  | | |  |  |  |  |  |  |  | |  | | | | | | |
|  | | | | | | | | | | | | 1 |  | AT |  | 7/26/2020 9:28 AM |  |
|  | They are entitled to that information its their right, they have a right to information so you will have hindered them. It is a disadvantage to them that we hinder giving them the information they are supposed to know. | | | | | | | | | | | | | | | |  |
|  |  | | | | | | | | | | | | | | | |  |
| Reports\\Coding Summary By Code Report | | | | | | | | | | Page 14 of 117 | | | | | | | |
| 8/15/2023 8:10 AM | | | | | | | | | | | | | | | | | |
|  | | | **Aggregate** |  | **Classification** |  | **Coverage** |  | **Number Of Coding References** | |  | **Reference Number** |  | **Coded By Initials** |  | **Modified On** |  |
|  | | | | | | | | | | | | | | | | | |
|  | | | | | | | | | | | | 2 |  | AT |  | 7/26/2020 9:30 AM |  |
|  | I don’t want to mean delay, I want to mean ongoing because there is information which we get as we go on with treatment but it is conditional but my drug because of the side effects as given you for example if they give you a drug you’ve been knocking but somehow I have put my drug in the wrong vein maybe I’ve Burt you, I have to give you that information you mean I would have told you immediately there that. | | | | | | | | | | | | | | | |  |
|  |  | | | | | | | | | | | | | | | |  |
|  | | | | | | | | | | | | 3 |  | AT |  | 7/26/2020 9:31 AM |  |
|  | that information is different from when I have done a mistake and I have not given you the information for example me personally I have ever done a mistake I gave a higher dose of a chemotherapy, the drug was supposed to go infusion. | | | | | | | | | | | | | | | |  |
|  |  | | | | | | | | | | | | | | | |  |
|  | **Nodes\\Barriers to consenting or communicating information about Cancer prognosis to patients and its related effects\Big patient crowds** | | | | | | | | | | | | | | | |  |
|  | | **Document** | | | | | | | | | | | | | | |  |
|  | | | **Files\\IDI - _ Doctor_UCI_06** | | | | | | | | | | | | | |  |
|  |  |  | No |  |  |  | 0.0343 |  | 2 | |  | | | | | |  |
|  | | |  |  |  |  |  |  |  | |  | | | | | | |
|  | | | | | | | | | | | | 1 |  | AT |  | 7/24/2020 8:51 AM |  |
|  | if it is adequacy it is about limitations of our part, we are not able to go through the full depth because as we are dealing with each patient we are also looking at the patient numbers around so we will touch something on the diagnosis, plan of treatment,, anticipated side effects and prognosis, we will say something but its not to a full depth radiation because of the numbers, I won’t go in to all the side effects that the patient will to know its not realistic because of the numbers. So, time is a constraint because of the large patients number and we feel the clinicians are not enough. | | | | | | | | | | | | | | | |  |
|  |  | | | | | | | | | | | | | | | |  |
|  | | | | | | | | | | | | 2 |  | AT |  | 7/24/2020 9:10 AM |  |
|  | The challenges are: we are few clinicians really compared to he numbers of patients we are seeing so we don’t adequately give all the information we would have wanted to give, we given basic information and when you give basic information you leave out certain aspects which later may interfere with the patient’s treatment so that is a big challenge. And also, the number of cancer patient cases have gone up to about three times while the number of health workers has also gone up, it has even up as high as the patient number so there are still gaps. | | | | | | | | | | | | | | | |  |
|  |  | | | | | | | | | | | | | | | |  |
|  | | | **Files\\IDI -Specialist palliative care_10** | | | | | | | | | | | | | |  |
|  |  |  | No |  |  |  | 0.0053 |  | 1 | |  | | | | | |  |
|  | | |  |  |  |  |  |  |  | |  | | | | | | |
|  | | | | | | | | | | | | 1 |  | AT |  | 7/24/2020 9:50 AM |  |
|  | And also the patient load its not possible to give as much information as you can because there is a line to clear and there are so many patients on the ward so you may not give as much information | | | | | | | | | | | | | | | |  |
|  |  | | | | | | | | | | | | | | | |  |
|  | | | **Files\\IDI- - Health Educator- UCI-09** | | | | | | | | | | | | | |  |
|  |  |  | No |  |  |  | 0.0113 |  | 3 | |  | | | | | |  |
|  | | |  |  |  |  |  |  |  | |  | | | | | | |
|  | | | | | | | | | | | | 1 |  | AT |  | 7/24/2020 10:28 AM |  |
|  | Because of the high number of patients even nurses do not have time to give additional information and because of the few staff like we have only one health educator for the entire patients so patients do not have a chance to listen or to hear personal information finally they are very few if at all there are no materials where patients can access to read more about their condition so they go to search on the net which sometimes gives false information so generally information given to patients is really scanty, its inadequate and not coordinated in a way. | | | | | | | | | | | | | | | |  |
|  |  | | | | | | | | | | | | | | | |  |
| Reports\\Coding Summary By Code Report | | | | | | | | | | Page 15 of 117 | | | | | | | |
| 8/15/2023 8:10 AM | | | | | | | | | | | | | | | | | |
|  | | | **Aggregate** |  | **Classification** |  | **Coverage** |  | **Number Of Coding References** | |  | **Reference Number** |  | **Coded By Initials** |  | **Modified On** |  |
|  | | | | | | | | | | | | | | | | | |
|  | | | | | | | | | | | | 2 |  | AT |  | 7/25/2020 9:50 PM |  |
|  | I say that the challenge is we have many patients and the doctors are few. | | | | | | | | | | | | | | | |  |
|  |  | | | | | | | | | | | | | | | |  |
|  | | | | | | | | | | | | 3 |  | AT |  | 7/25/2020 10:24 PM |  |
|  | we gain nurses by the number of patients they have given chemotherapy, doctors: by the number of patients they have clerked information is not one measure of performance. | | | | | | | | | | | | | | | |  |
|  |  | | | | | | | | | | | | | | | |  |
|  | | | **Files\\IDI__ Doctor_ UCI_ 05** | | | | | | | | | | | | | |  |
|  |  |  | No |  |  |  | 0.0101 |  | 1 | |  | | | | | |  |
|  | | |  |  |  |  |  |  |  | |  | | | | | | |
|  | | | | | | | | | | | | 1 |  | AT |  | 7/26/2020 10:44 AM |  |
|  | In the developed world, on a particular visit you see three to four patients who are just beginning, three to four. Me I see between ten to fifteen beginning and yet I am also reevaluating those who are finished who may also be between ten to fifteen so those are about twenty to thirty patients on a particular visit now that’s time secondly, we have so many patients from different walks of life. | | | | | | | | | | | | | | | |  |
|  |  | | | | | | | | | | | | | | | |  |
|  | **Nodes\\Barriers to consenting or communicating information about Cancer prognosis to patients and its related effects\Delayed disclosure** | | | | | | | | | | | | | | | |  |
|  | | **Document** | | | | | | | | | | | | | | |  |
|  | | | **Files\\IDI - _ Doctor_UCI_06** | | | | | | | | | | | | | |  |
|  |  |  | No |  |  |  | 0.0113 |  | 1 | |  | | | | | |  |
|  | | |  |  |  |  |  |  |  | |  | | | | | | |
|  | | | | | | | | | | | | 1 |  | AT |  | 7/24/2020 8:42 AM |  |
|  | We first manage some symptoms then when they are stronger, we bring out the topic and prognosis. But the disadvantage also is that there is delaying taking critical decision because we are waiting for the patient to first recover well as you may want to actually initiate radiotherapy or chemotherapy early, you will want to get the disease where it is responsible for treatment. | | | | | | | | | | | | | | | |  |
|  |  | | | | | | | | | | | | | | | |  |
|  | **Nodes\\Barriers to consenting or communicating information about Cancer prognosis to patients and its related effects\Denial** | | | | | | | | | | | | | | | |  |
|  | | **Document** | | | | | | | | | | | | | | |  |
|  | | | **Files\\IDI - _ Doctor_UCI_06** | | | | | | | | | | | | | |  |
|  |  |  | No |  |  |  | 0.0303 |  | 2 | |  | | | | | |  |
|  | | |  |  |  |  |  |  |  | |  | | | | | | |
|  | | | | | | | | | | | | 1 |  | AT |  | 7/24/2020 8:46 AM |  |
|  | I think most times where that scenario arises is, the patients actually are told but maybe we as clinicians we have not asked them whether they have understood what we are telling them or a good number of our patients live in denial, you will talk to the patient and tell them the diagnosis and plan of treatment and they will go to another health worker and say no one has talked to them about what they are suffering from then they will go to another one again because they want to get some hope or positive information so its like they keep moving from one doctor because when you talk to the patients and their attendants. | | | | | | | | | | | | | | | |  |
|  |  | | | | | | | | | | | | | | | |  |
| Reports\\Coding Summary By Code Report | | | | | | | | | | Page 16 of 117 | | | | | | | |
| 8/15/2023 8:10 AM | | | | | | | | | | | | | | | | | |
|  | | | **Aggregate** |  | **Classification** |  | **Coverage** |  | **Number Of Coding References** | |  | **Reference Number** |  | **Coded By Initials** |  | **Modified On** |  |
|  | | | | | | | | | | | | | | | | | |
|  | | | | | | | | | | | | 2 |  | AT |  | 7/24/2020 8:46 AM |  |
|  | Attendants will come out and say oh we went to the other doctor he told this then we went to another one that sort of thing and then when you ask them again they are like do you understand what you’re facing! no, so sometimes I think it is leaving in denial, a good number of them because really by just being in the cancer institute and getting treatment that 99% mean that they have cancer. | | | | | | | | | | | | | | | |  |
|  |  | | | | | | | | | | | | | | | |  |
|  | | | **Files\\IDI -Specialist palliative care_10** | | | | | | | | | | | | | |  |
|  |  |  | No |  |  |  | 0.0070 |  | 1 | |  | | | | | |  |
|  | | |  |  |  |  |  |  |  | |  | | | | | | |
|  | | | | | | | | | | | | 1 |  | AT |  | 7/24/2020 9:42 AM |  |
|  | They really do but also you find that this is a patient in denial who doesn’t want to tell them and has not even checked and say when you tell them they say oh ok you are discouraging me or relatives have kept them away from any internet that they don’t know. | | | | | | | | | | | | | | | |  |
|  |  | | | | | | | | | | | | | | | |  |
|  | | | **Files\\IDI_ Counselor_UCI_01** | | | | | | | | | | | | | |  |
|  |  |  | No |  |  |  | 0.0280 |  | 1 | |  | | | | | |  |
|  | | |  |  |  |  |  |  |  | |  | | | | | | |
|  | | | | | | | | | | | | 1 |  | AT |  | 7/25/2020 10:53 PM |  |
|  | One of the things is denial like you mentioned. Two, stigma also can cause it, they don’t want to associate them selves with cancer then three lack of information, they were not told sometimes doctors will just work and assume the patient is aware of what is happening and the patient goes starts care two years down the road the patient will be saying I don’t know that I have cancer they have been telling me I have cancer sometimes they say it is not there such things, you find that the patient is having a double mind to say sometimes they told me I have cancer then later they say no it is not there I don’t have cancer then you wonder someone has been on treatment for two years but is still saying that he doesn’t have cancer so it has happened. | | | | | | | | | | | | | | | |  |
|  |  | | | | | | | | | | | | | | | |  |
|  | | | **Files\\IDI_ Nurse_UCI_07** | | | | | | | | | | | | | |  |
|  |  |  | No |  |  |  | 0.0193 |  | 2 | |  | | | | | |  |
|  | | |  |  |  |  |  |  |  | |  | | | | | | |
|  | | | | | | | | | | | | 1 |  | AT |  | 7/26/2020 8:27 AM |  |
|  | The disadvantages for immediate disclosure is that it is really not easy for some individuals to accept bad news yes so denial is common and you find that some patients don’t come back until after a certain period of time. | | | | | | | | | | | | | | | |  |
|  |  | | | | | | | | | | | | | | | |  |
|  | | | | | | | | | | | | 2 |  | AT |  | 7/26/2020 8:28 AM |  |
|  | Yeah, it is somehow different, denial you no and then you take time like six months to come back but shock if we are giving you treatment, you don’t even get treatment that day but you can decide to come back in two days’ time because the news has really shocked you but at least you come back in the nest two days but with denial they take some time, some even take a year to come back. Today there is someone who has taken a year, they got lost to follow up and it has been a year. | | | | | | | | | | | | | | | |  |
|  |  | | | | | | | | | | | | | | | |  |
|  | | | **Files\\IDI_ _ Nurse_UCI_04** | | | | | | | | | | | | | |  |
|  |  |  | No |  |  |  | 0.0201 |  | 1 | |  | | | | | |  |
|  | | |  |  |  |  |  |  |  | |  | | | | | | |
|  | | | | | | | | | | | | 1 |  | AT |  | 7/26/2020 10:04 AM |  |
|  | If I could look at it from this angle, one of it is the way the patient presents in denial state, first of all if the patient has presented when he or she is alone but has got other pressing issues to find so as to stabilize the patient we may first want to stabilize the patient then once the patient is stable then get to talk to the patient other than you know the patient is struggling and then we are going to aggreviate the suffering but when we break in information there already the person may go in to shock so you need this person in a well sound mind so that you are ready to talk to the person communicate and break information in a proper way. | | | | | | | | | | | | | | | |  |
|  |  | | | | | | | | | | | | | | | |  |
| Reports\\Coding Summary By Code Report | | | | | | | | | | Page 17 of 117 | | | | | | | |
| 8/15/2023 8:10 AM | | | | | | | | | | | | | | | | | |
|  | | | **Aggregate** |  | **Classification** |  | **Coverage** |  | **Number Of Coding References** | |  | **Reference Number** |  | **Coded By Initials** |  | **Modified On** |  |
|  | | | **Files\\IDI__ Doctor_ UCI_ 05** | | | | | | | | | | | | | |  |
|  |  |  | No |  |  |  | 0.0077 |  | 1 | |  | | | | | |  |
|  | | |  |  |  |  |  |  |  | |  | | | | | | |
|  | | | | | | | | | | | | 1 |  | AT |  | 7/26/2020 10:41 AM |  |
|  | they are very simple, the more you delay telling this patient the more they continue in denial. Most patients come in denial so the more you delay giving information, I think you’re to impact on the denial of this patient may be the only thing that you can agree with is giving this information in bits. | | | | | | | | | | | | | | | |  |
|  |  | | | | | | | | | | | | | | | |  |
|  | **Nodes\\Barriers to consenting or communicating information about Cancer prognosis to patients and its related effects\Dilemma of telling the truth** | | | | | | | | | | | | | | | |  |
|  | | **Document** | | | | | | | | | | | | | | |  |
|  | | | **Files\\IDI- - Health Educator- UCI-09** | | | | | | | | | | | | | |  |
|  |  |  | No |  |  |  | 0.0134 |  | 2 | |  | | | | | |  |
|  | | |  |  |  |  |  |  |  | |  | | | | | | |
|  | | | | | | | | | | | | 1 |  | AT |  | 7/24/2020 12:51 PM |  |
|  | I mean for a patient to know his cancer occurs in the clerking room the day they diagnose the patient so the treatment happens when the senior doctor reviews the person now after reviewing this person, when this person is not fit for treatment any more that is the disclosure that will happen at this point or shed another day but part of the practice that I have experienced some doctors will be trapped between saying the truth and lying to the patient and you realise that patients who can no longer benefit in that kind of care still are compelled because this doctor now doesn’t want to tell this patient that my dear this will not help you. | | | | | | | | | | | | | | | |  |
|  |  | | | | | | | | | | | | | | | |  |
|  | | | | | | | | | | | | 2 |  | AT |  | 7/24/2020 12:53 PM |  |
|  | Lies are happening because you do not want, these people came for cure they do not know that kind of words you want to tell them that here we do not these tests, that’s all so may be out of their experience they have ever crossed ways with cancer treatment support after them trying to speak out the truth. | | | | | | | | | | | | | | | |  |
|  |  | | | | | | | | | | | | | | | |  |
|  | | | **Files\\IDI_ Nurse_UCI_02** | | | | | | | | | | | | | |  |
|  |  |  | No |  |  |  | 0.0704 |  | 7 | |  | | | | | |  |
|  | | |  |  |  |  |  |  |  | |  | | | | | | |
|  | | | | | | | | | | | | 1 |  | AT |  | 7/26/2020 9:27 AM |  |
|  | The patients will lose trust in you because once they find out they will be like you told us we are going to be fine now she goes to another doctor and the doctor says no then the patient loses trust and it is a very big disadvantage and sometimes they don’t even come back for the treatment. | | | | | | | | | | | | | | | |  |
|  |  | | | | | | | | | | | | | | | |  |
|  | | | | | | | | | | | | 2 |  | AT |  | 7/26/2020 9:47 AM |  |
|  | If I lie that you will be fine tells the other people the social people I’m going to be fine, yes there will be the stigma and even the people surrounding you will still have that hope that you will be fine. | | | | | | | | | | | | | | | |  |
|  |  | | | | | | | | | | | | | | | |  |
|  | | | | | | | | | | | | 3 |  | AT |  | 7/26/2020 9:47 AM |  |
|  | you lie that they will be fine, if the social people have been helping aiming cure then they will withdraw so you find the patient not getting social care. | | | | | | | | | | | | | | | |  |
|  |  | | | | | | | | | | | | | | | |  |
|  | | | | | | | | | | | | | | | | | |
| Reports\\Coding Summary By Code Report | | | | | | | | | | Page 18 of 117 | | | | | | | |
| 8/15/2023 8:10 AM | | | | | | | | | | | | | | | | | |
|  | | | **Aggregate** |  | **Classification** |  | **Coverage** |  | **Number Of Coding References** | |  | **Reference Number** |  | **Coded By Initials** |  | **Modified On** |  |
|  | | | | | | | | | | | | | | | | | |
|  | | | | | | | | | | | | 4 |  | AT |  | 7/26/2020 9:47 AM |  |
|  | If you lie and the family doesn’t know, they will not get that best care, some of them have to know that information and then cancer I’m I going to get the cancer when am caring for my patient so if you lie some of them will deny the care. Like that patient I told you whom we did not want to disclose, they did not want to touch her yet she was in a very critical condition the faeces the what, they were doing everything for her but she had refused to disclose to them what she is suffering from and they kept on asking until they got these books, is cancer transmitted to another person so if you lie to family members it is somehow a disservice that one. Two the prognosis at least some members will know and prepare themselves by planning for the family and then the will making. | | | | | | | | | | | | | | | |  |
|  |  | | | | | | | | | | | | | | | |  |
|  | | | | | | | | | | | | 5 |  | AT |  | 7/26/2020 9:48 AM |  |
|  | To some it has an impact, to some they will not, some people don’t believe our words, what they will not tell them sand others have called us the liars. In case the patient passes on when they got a lie from the health providers it is accountable on you who lied. | | | | | | | | | | | | | | | |  |
|  |  | | | | | | | | | | | | | | | |  |
|  | | | | | | | | | | | | 6 |  | AT |  | 7/26/2020 9:48 AM |  |
|  | It affects them also by the way it affects the patient so much because they will know if they have told me I can be able to give my life like the born agains give their lives to Christ and they know where they are heading if you lie to them, they will not prepare their souls for where they are going. | | | | | | | | | | | | | | | |  |
|  |  | | | | | | | | | | | | | | | |  |
|  | | | | | | | | | | | | 7 |  | AT |  | 7/26/2020 9:48 AM |  |
|  | It is not easy but also sometimes I was still telling you that there are those ones who think that you’re lying even if you’re telling them the truth that she will be fine, they will say those are musawo’s words. So sometimes it is according to their fate that if you have faith you can heal. They have trust in our words and some may not trust and they also challenge us like they are there knowing that someone is going to die then you see some one living one year second year and on. | | | | | | | | | | | | | | | |  |
|  |  | | | | | | | | | | | | | | | |  |
|  | | | **Files\\IDI_ _ Nurse_UCI_04** | | | | | | | | | | | | | |  |
|  |  |  | No |  |  |  | 0.0559 |  | 4 | |  | | | | | |  |
|  | | |  |  |  |  |  |  |  | |  | | | | | | |
|  | | | | | | | | | | | | 1 |  | AT |  | 7/26/2020 10:04 AM |  |
|  | I think ideally as a health worker you’re not meant to really lie in the care of you patient you’re meant to be clear to your patient and tell your patient ideally what is important to do because if you lie to the patient then you’re going to lose trust of the patient. | | | | | | | | | | | | | | | |  |
|  |  | | | | | | | | | | | | | | | |  |
|  | | | | | | | | | | | | 2 |  | AT |  | 7/26/2020 10:04 AM |  |
|  | I don’t really see why the health worker would actually see how to really lie. Well of course they may be there but I think they are more disadvantages to that lying than the advantages that you would want to look at. It mainly negatively impacts on the patients care. | | | | | | | | | | | | | | | |  |
|  |  | | | | | | | | | | | | | | | |  |
|  | | | | | | | | | | | | 3 |  | AT |  | 7/26/2020 10:05 AM |  |
|  | Yeah, there are those instances where by the patient given kind of hope like everything will be okay, your thing is still very early I know, I think we can do this, I think lets get to do this but in the process when things are not working out then they tell the patient your things are like this, eventually that patient in the process loses trust of that doctor and will not want to return back to that doctor. Whenever he or she comes will rather go and look for some other doctor and say get me someone else or get to the nurses and say please. In most cases they tend to confine o the nurses and they tend to express themselves say please I don’t want such and such a doctor or I don’t want Mr. X and like Mr. M is the one going to help and sort my issues out. So, it negatively impacts on the patient’s care. | | | | | | | | | | | | | | | |  |
|  |  | | | | | | | | | | | | | | | |  |
|  | | | | | | | | | | | | 4 |  | AT |  | 7/26/2020 10:07 AM |  |
|  | Sometimes what could may be make them lie in this essence is possibly when you realise the patient is not in a proper mental state and the patient is kind of confused so this sometimes kind of affect and sometimes the information you give sometimes you want to give information that can tend to give the individual so you just talk of things that can tend to come to the individual not only talking the truth because you see the person is not a proper mental state. | | | | | | | | | | | | | | | |  |
|  |  | | | | | | | | | | | | | | | |  |
|  | | | **Files\\IDI__ Doctor_ UCI_ 05** | | | | | | | | | | | | | |  |
|  |  |  | No |  |  |  | 0.0155 |  | 2 | |  | | | | | |  |
|  | | |  |  |  |  |  |  |  | |  | | | | | | |
|  | | | | | | | | | | | | 1 |  | AT |  | 7/26/2020 10:37 AM |  |
|  | I never lie, I am not boasting or anything but I hold patients in a very high regard especially cancer patients and I don’t lie. | | | | | | | | | | | | | | | |  |
|  |  | | | | | | | | | | | | | | | |  |
| Reports\\Coding Summary By Code Report | | | | | | | | | | Page 19 of 117 | | | | | | | |
| 8/15/2023 8:10 AM | | | | | | | | | | | | | | | | | |
|  | | | **Aggregate** |  | **Classification** |  | **Coverage** |  | **Number Of Coding References** | |  | **Reference Number** |  | **Coded By Initials** |  | **Modified On** |  |
|  | | | | | | | | | | | | | | | | | |
|  | | | | | | | | | | | | 2 |  | AT |  | 7/26/2020 10:37 AM |  |
|  | I prefer to start speaking about my self because I see patients, I don’t hold back information, if you’re stage 1 I say stage 1, if you’re stage 2 you’re stage 2 and all that has implications. If you’re stage 4 and you’re advanced I cant change that and ii usually like using a phrase which goes by “I’m not God, am just a human being” And am able to do so much so I don’t lie to patients I tell them the truth according to the information I have because am just a medical provider. | | | | | | | | | | | | | | | |  |
|  |  | | | | | | | | | | | | | | | |  |
|  | **Nodes\\Barriers to consenting or communicating information about Cancer prognosis to patients and its related effects\Fear** | | | | | | | | | | | | | | | |  |
|  | | **Document** | | | | | | | | | | | | | | |  |
|  | | | **Files\\IDI_ Social worker_UCI_03** | | | | | | | | | | | | | |  |
|  |  |  | No |  |  |  | 0.0070 |  | 2 | |  | | | | | |  |
|  | | |  |  |  |  |  |  |  | |  | | | | | | |
|  | | | | | | | | | | | | 1 |  | AT |  | 7/26/2020 9:10 AM |  |
|  | Yes, because somebody might also be fearing you never the doctor also have fear to disclose that. | | | | | | | | | | | | | | | |  |
|  |  | | | | | | | | | | | | | | | |  |
|  | | | | | | | | | | | | 2 |  | AT |  | 7/26/2020 9:11 AM |  |
|  | When I tell them the patient might die they say I am scaring the patient or if it is relative or your like your father you become scared, you don’t have the skills, fear can also contribute to that. | | | | | | | | | | | | | | | |  |
|  |  | | | | | | | | | | | | | | | |  |
|  | **Nodes\\Barriers to consenting or communicating information about Cancer prognosis to patients and its related effects\Heavy work load** | | | | | | | | | | | | | | | |  |
|  | | **Document** | | | | | | | | | | | | | | |  |
|  | | | **Files\\IDI -Specialist palliative care_10** | | | | | | | | | | | | | |  |
|  |  |  | No |  |  |  | 0.0105 |  | 1 | |  | | | | | |  |
|  | | |  |  |  |  |  |  |  | |  | | | | | | |
|  | | | | | | | | | | | | 1 |  | AT |  | 7/24/2020 9:35 AM |  |
|  | In the outpatients people tend to talk to them although you know how the patient load is in outpatient of course I have not been there to really say what goes on there as I cant say with confidence but I think the doctors who work on them the outpatients talk to them because many times eventually when they are sick and come to the ward you know that they have been told they have a diagnosis. | | | | | | | | | | | | | | | |  |
|  |  | | | | | | | | | | | | | | | |  |
|  | | | **Files\\IDI- - Health Educator- UCI-09** | | | | | | | | | | | | | |  |
|  |  |  | No |  |  |  | 0.0027 |  | 1 | |  | | | | | |  |
|  | | |  |  |  |  |  |  |  | |  | | | | | | |
|  | | | | | | | | | | | | 1 |  | AT |  | 7/25/2020 10:25 PM |  |
|  | sometimes when a patient takes time to ask a question, will be asking questions for a deeper reason, the attitude towards patients influence the response towards patients so besides the workload, | | | | | | | | | | | | | | | |  |
|  |  | | | | | | | | | | | | | | | |  |
| Reports\\Coding Summary By Code Report | | | | | | | | | | Page 20 of 117 | | | | | | | |
| 8/15/2023 8:10 AM | | | | | | | | | | | | | | | | | |
|  | | | **Aggregate** |  | **Classification** |  | **Coverage** |  | **Number Of Coding References** | |  | **Reference Number** |  | **Coded By Initials** |  | **Modified On** |  |
|  | | | **Files\\IDI_ Counselor_UCI_01** | | | | | | | | | | | | | |  |
|  |  |  | No |  |  |  | 0.0150 |  | 1 | |  | | | | | |  |
|  | | |  |  |  |  |  |  |  | |  | | | | | | |
|  | | | | | | | | | | | | 1 |  | AT |  | 7/25/2020 10:57 PM |  |
|  | Sometimes it could be because of the work load when there are too many patients on the line and they decide to keep pushing to another person to do the disclosure. If there is someone near the senior doctors especially with counsellor they will always put in writing and then they say go to the counsellor so that you disclose and also guide the patients on where to go next. So, work load is one of them | | | | | | | | | | | | | | | |  |
|  |  | | | | | | | | | | | | | | | |  |
|  | | | **Files\\IDI_ Social worker_UCI_03** | | | | | | | | | | | | | |  |
|  |  |  | No |  |  |  | 0.0050 |  | 1 | |  | | | | | |  |
|  | | |  |  |  |  |  |  |  | |  | | | | | | |
|  | | | | | | | | | | | | 1 |  | AT |  | 7/26/2020 8:59 AM |  |
|  | I think the volume of work influence that to me, there could be that added. At the beginning they can start well and as time goes on the number of patients that come in the later patients, I think they are affected. | | | | | | | | | | | | | | | |  |
|  |  | | | | | | | | | | | | | | | |  |
|  | | | **Files\\IDI_ _ Nurse_UCI_04** | | | | | | | | | | | | | |  |
|  |  |  | No |  |  |  | 0.0104 |  | 1 | |  | | | | | |  |
|  | | |  |  |  |  |  |  |  | |  | | | | | | |
|  | | | | | | | | | | | | 1 |  | AT |  | 7/26/2020 10:00 AM |  |
|  | I think its because sometimes there are times when the clinicians tend to be overwhelmed and when patients come here, we don’t bounce patients that we are tired, we will not see you so you’re pushed to try to see patients but in most cases patients who get when the clinicians are already overwhelmed don’t get all the adequate information. | | | | | | | | | | | | | | | |  |
|  |  | | | | | | | | | | | | | | | |  |
|  | **Nodes\\Barriers to consenting or communicating information about Cancer prognosis to patients and its related effects\Inadequate information** | | | | | | | | | | | | | | | |  |
|  | | **Document** | | | | | | | | | | | | | | |  |
|  | | | **Files\\IDI - - Doctor- UCI -08** | | | | | | | | | | | | | |  |
|  |  |  | No |  |  |  | 0.0078 |  | 1 | |  | | | | | |  |
|  | | |  |  |  |  |  |  |  | |  | | | | | | |
|  | | | | | | | | | | | | 1 |  | AT |  | 7/24/2020 8:17 AM |  |
|  | It will depend on who is giving the information for the doctors and nurses at least I would say they give adequate information but these other cadres who are not clinical staff probably they may not give adequate information. | | | | | | | | | | | | | | | |  |
|  |  | | | | | | | | | | | | | | | |  |
|  | | | **Files\\IDI - _ Doctor_UCI_06** | | | | | | | | | | | | | |  |
|  |  |  | No |  |  |  | 0.0197 |  | 2 | |  | | | | | |  |
|  | | |  |  |  |  |  |  |  | |  | | | | | | |
|  | | | | | | | | | | | | 1 |  | AT |  | 7/24/2020 8:52 AM |  |
|  | there are limitations there I must admit, a good number of times we tell the patient some aspect of the informed consent, the other aspects we find they are not covered but we do give them the basics of informed consent. | | | | | | | | | | | | | | | |  |
|  |  | | | | | | | | | | | | | | | |  |
| Reports\\Coding Summary By Code Report | | | | | | | | | | Page 21 of 117 | | | | | | | |
| 8/15/2023 8:10 AM | | | | | | | | | | | | | | | | | |
|  | | | **Aggregate** |  | **Classification** |  | **Coverage** |  | **Number Of Coding References** | |  | **Reference Number** |  | **Coded By Initials** |  | **Modified On** |  |
|  | | | | | | | | | | | | | | | | | |
|  | | | | | | | | | | | | 2 |  | AT |  | 7/24/2020 9:01 AM |  |
|  | Yes, generally speaking am sure your are aware informed consent is an evolving process, its not a one off so when that process is obtained in opening of the file its based on the basic so when they do come to the clinic we do again, we don’t do a separate consent but we confirm because they are at least aware of what is happening. But I make also a note that they may not be given all that information right when they are opening the file. | | | | | | | | | | | | | | | |  |
|  |  | | | | | | | | | | | | | | | |  |
|  | | | **Files\\IDI -Specialist palliative care_10** | | | | | | | | | | | | | |  |
|  |  |  | No |  |  |  | 0.0109 |  | 1 | |  | | | | | |  |
|  | | |  |  |  |  |  |  |  | |  | | | | | | |
|  | | | | | | | | | | | | 1 |  | AT |  | 7/24/2020 9:50 AM |  |
|  | and even when we talk about side effects we are not giving enough information on side effects because you can’t come and say everything in one go or two sessions so there should be opportunities for people to share, ask or read books and when someone is like this is something I would have wanted to ask the doctor in OPD but I was so tensed I forgot now I want to ask and they can find out that information. | | | | | | | | | | | | | | | |  |
|  |  | | | | | | | | | | | | | | | |  |
|  | | | **Files\\IDI- - Health Educator- UCI-09** | | | | | | | | | | | | | |  |
|  |  |  | No |  |  |  | 0.0497 |  | 11 | |  | | | | | |  |
|  | | |  |  |  |  |  |  |  | |  | | | | | | |
|  | | | | | | | | | | | | 1 |  | AT |  | 7/24/2020 10:28 AM |  |
|  | Because of the high number of patients even nurses do not have time to give additional information and because of the few staff like we have only one health educator for the entire patients so patients do not have a chance to listen or to hear personal information finally they are very few if at all there are no materials where patients can access to read more about their condition so they go to search on the net which sometimes gives false information so generally information given to patients is really scanty, its inadequate and not coordinated in a way. | | | | | | | | | | | | | | | |  |
|  |  | | | | | | | | | | | | | | | |  |
|  | | | | | | | | | | | | 2 |  | AT |  | 7/24/2020 10:30 AM |  |
|  | Many times, we do not get the patients what they want so patients leave cancer institute when they have that information they end asking their colleagues that they don’t have accurate information so the expectation of patients as pertains information ranges from update of information and most times their information needs are not met because of those reasons I have told you. | | | | | | | | | | | | | | | |  |
|  |  | | | | | | | | | | | | | | | |  |
|  | | | | | | | | | | | | 3 |  | AT |  | 7/25/2020 9:57 PM |  |
|  | Generally, the information we give to patients is very inadequate because information giving is not a priority so it is totally inadequate. | | | | | | | | | | | | | | | |  |
|  |  | | | | | | | | | | | | | | | |  |
|  | | | | | | | | | | | | 4 |  | AT |  | 7/25/2020 9:57 PM |  |
|  | It is not a priority in care, in cancer care information is not a priority. | | | | | | | | | | | | | | | |  |
|  |  | | | | | | | | | | | | | | | |  |
|  | | | | | | | | | | | | 5 |  | AT |  | 7/25/2020 9:57 PM |  |
|  | This is how our systems have been set - a doctor sees the patient and pays no regard to patient’s understanding. The doctor writes everything but doesn’t spare little time to make a person know what he/she has written | | | | | | | | | | | | | | | |  |
|  |  | | | | | | | | | | | | | | | |  |
|  | | | | | | | | | | | | 6 |  | AT |  | 7/25/2020 9:58 PM |  |
|  | The doctor prescribes treatment and it is given to the patient when the patient is not part of the package. when you give information with in the communications there are those elements that must be very clear who is the person giving information, in which form is the information given, is it understandable? So, some of these are silent because it is not a priority. It is rare to find in the guidelines for cancer care that information is indicated. | | | | | | | | | | | | | | | |  |
|  |  | | | | | | | | | | | | | | | |  |
|  | | | | | | | | | | | | 7 |  | AT |  | 7/25/2020 10:00 PM |  |
|  | They are signing if you look in to their forms, they sign if you through it, you will go and find a patient who has hard six cycles of chemo and patient doesn’t understand what is going to happen next. | | | | | | | | | | | | | | | |  |
|  |  | | | | | | | | | | | | | | | |  |
|  | | | | | | | | | | | | 8 |  | AT |  | 7/25/2020 10:17 PM |  |
|  | Maybe I will sorry for this in that what happens is not informed consent that is consent, I would take the word informed consent look in to one, what is the magnitude of information given at the registration point, there are no tools at all to support giving information at the registration point. | | | | | | | | | | | | | | | |  |
|  |  | | | | | | | | | | | | | | | |  |
| Reports\\Coding Summary By Code Report | | | | | | | | | | Page 22 of 117 | | | | | | | |
| 8/15/2023 8:10 AM | | | | | | | | | | | | | | | | | |
|  | | | **Aggregate** |  | **Classification** |  | **Coverage** |  | **Number Of Coding References** | |  | **Reference Number** |  | **Coded By Initials** |  | **Modified On** |  |
|  | | | | | | | | | | | | | | | | | |
|  | | | | | | | | | | | | 9 |  | AT |  | 7/25/2020 10:21 PM |  |
|  | The information given is incomplete from what we are talking about, we are now going to where and when consent should take place. Improvement of consent at intervention point is more important, first of all it will enhance giving information and at that point it enhances patients understanding of the procedures he or she is consenting for so consent has to be given at intervention points. | | | | | | | | | | | | | | | |  |
|  |  | | | | | | | | | | | | | | | |  |
|  | | | | | | | | | | | | 10 |  | AT |  | 7/25/2020 10:40 PM |  |
|  | The second challenge is providing information before consent some of the persons may not be technical to provide detailed information on the content of the consent form but also technical people who should have given this information accurately may be very few so at what point and how better can informed consent be given is a challenge, it’s a question which requires a little bit of critical analysis to come up with the way of doing that. | | | | | | | | | | | | | | | |  |
|  |  | | | | | | | | | | | | | | | |  |
|  | | | | | | | | | | | | 11 |  | AT |  | 7/25/2020 10:40 PM |  |
|  | The third challenge I would come up with is the challenge related to communication because informed consent requires information, the way information is given from the communication point of view may not make informed consent retain the meaning of informed consent either it is not explained or the patient has not understood all those come around communication challenge. | | | | | | | | | | | | | | | |  |
|  |  | | | | | | | | | | | | | | | |  |
|  | | | **Files\\IDI_ Social worker_UCI_03** | | | | | | | | | | | | | |  |
|  |  |  | No |  |  |  | 0.0417 |  | 4 | |  | | | | | |  |
|  | | |  |  |  |  |  |  |  | |  | | | | | | |
|  | | | | | | | | | | | | 1 |  | AT |  | 7/26/2020 8:52 AM |  |
|  | I have said that and am repeating it that it is nearly 80% do not know about their diagnosis. | | | | | | | | | | | | | | | |  |
|  |  | | | | | | | | | | | | | | | |  |
|  | | | | | | | | | | | | 2 |  | AT |  | 7/26/2020 8:56 AM |  |
|  | The information is not adequate because if the information was adequate then we wouldn’t find patients that are falling off the treatment, we wouldn’t find families that are not coming to support the patient, we wouldn’t find may be like sometimes patients deciding to leave under the veranda here and not to go home because of sometimes that causes misunderstandings in the family because if they all have understood or the patient himself has understood then can explain to the family or the family have understood together then they will ensure that we support this one to go through it, we have patients who are lying under the veranda because of understanding vaguely that they have cancer now the rest of the information is not known so now they base on those myths so the myths now help them to take action and understand so the information is not adequate and the other thing is that even referral because information also comes with referral. | | | | | | | | | | | | | | | |  |
|  |  |  |  |  |  |  |  |  |  |  |  |  |  |  |  |  |  |
|  |  | | | | | | | | | | | | | | | |  |
|  | | | | | | | | | | | | 3 |  | AT |  | 7/26/2020 8:56 AM |  |
|  | If it is not proper, you cannot give information you send to the other bit of the information that is remaining because we don’t receive such referral often we only receive referral when now there is trouble in the family, instead of also referring patients to understand what is required of social aspect of care we don’t, we are only stormed when there is already violence, there is already divorce, tension that it is very difficult sometimes to handle and here again cancer institute does not have legal processes that the other side would give the patient care until the end. | | | | | | | | | | | | | | | |  |
|  |  | | | | | | | | | | | | | | | |  |
|  | | | | | | | | | | | | 4 |  | AT |  | 7/26/2020 9:07 AM |  |
|  | I don’t know about other departments what they have identified but for us as social workers we think patients one they don’t have enough information. | | | | | | | | | | | | | | | |  |
|  |  | | | | | | | | | | | | | | | |  |
|  | | | **Files\\IDI_ Nurse_UCI_02** | | | | | | | | | | | | | |  |
|  |  |  | No |  |  |  | 0.0410 |  | 6 | |  | | | | | |  |
|  | | |  |  |  |  |  |  |  | |  | | | | | | |
|  | | | | | | | | | | | | 1 |  | AT |  | 7/26/2020 9:32 AM |  |
|  | If it is 40%, is it inadequate because I don’t even think we go to 50%. | | | | | | | | | | | | | | | |  |
|  |  | | | | | | | | | | | | | | | |  |
|  | | | | | | | | | | | | 2 |  | AT |  | 7/26/2020 9:32 AM |  |
|  | The information you give them is inadequate. But why do say that it is inadequate, do you take these other aspects of human source, time you will maybe take that. | | | | | | | | | | | | | | | |  |
|  |  | | | | | | | | | | | | | | | |  |
| Reports\\Coding Summary By Code Report | | | | | | | | | | Page 23 of 117 | | | | | | | |
| 8/15/2023 8:10 AM | | | | | | | | | | | | | | | | | |
|  | | | **Aggregate** |  | **Classification** |  | **Coverage** |  | **Number Of Coding References** | |  | **Reference Number** |  | **Coded By Initials** |  | **Modified On** |  |
|  | | | | | | | | | | | | | | | | | |
|  | | | | | | | | | | | | 3 |  | AT |  | 7/26/2020 9:37 AM |  |
|  | I am going to mention from the ones I see who can give you information are the cadres of staff first are the counsellors, they just with the patient and they will go deep and I don’t know why patients like opening up to those counsellors more than the nurses, I don’t know because she will tell you I have told all my issues to the counsellor so the counselors I think they empty the information. Followed by the nurses then the doctors but they may not empty everything. | | | | | | | | | | | | | | | |  |
|  |  | | | | | | | | | | | | | | | |  |
|  | | | | | | | | | | | | 4 |  | AT |  | 7/26/2020 9:38 AM |  |
|  | They don’t give them information, but what information would they give them? I don’t think they have enough information to give those people. | | | | | | | | | | | | | | | |  |
|  |  | | | | | | | | | | | | | | | |  |
|  | | | | | | | | | | | | 5 |  | AT |  | 7/26/2020 9:48 AM |  |
|  | In that process there is inadequate information, patients don’t get adequate information about what they are signing for. The personnel who are doing that informed consent, they don’t have the information and probably the are not the right people to do the informed consent process. It is general, where some has to consent for chemotherapy, surgery, they don’t give them that option then is consenting for everything then time. It is a general thing then there is limited time for them to do that consent, there is limited time | | | | | | | | | | | | | | | |  |
|  |  | | | | | | | | | | | | | | | |  |
|  | | | | | | | | | | | | 6 |  | AT |  | 7/26/2020 9:50 AM |  |
|  | They just sign, sign here but there don’t understand what am I signing for. | | | | | | | | | | | | | | | |  |
|  |  | | | | | | | | | | | | | | | |  |
|  | | | **Files\\IDI_ _ Nurse_UCI_04** | | | | | | | | | | | | | |  |
|  |  |  | No |  |  |  | 0.0217 |  | 2 | |  | | | | | |  |
|  | | |  |  |  |  |  |  |  | |  | | | | | | |
|  | | | | | | | | | | | | 1 |  | AT |  | 7/26/2020 10:12 AM |  |
|  | I think they are not getting adequate information given the numbers we have because we still have a great number of people who keep on rotating u and down, they even don’t know and we still have come across those patients that are coming for treatment and they don’t know why they are here. For example, someone is says but why I’m I here, what condition do I have, what has exactly brought me here so I think the information is not adequately given to the patients. | | | | | | | | | | | | | | | |  |
|  |  | | | | | | | | | | | | | | | |  |
|  | | | | | | | | | | | | 2 |  | AT |  | 7/26/2020 10:13 AM |  |
|  | They usually just show to them that this is it now you sign here let me open the file for you so your work is basically sign because you know that is the way the file is going to be opened well that is first of all one thing you’re bounding | | | | | | | | | | | | | | | |  |
|  |  | | | | | | | | | | | | | | | |  |
|  | | | **Files\\IDI__ Doctor_ UCI_ 05** | | | | | | | | | | | | | |  |
|  |  |  | No |  |  |  | 0.0182 |  | 2 | |  | | | | | |  |
|  | | |  |  |  |  |  |  |  | |  | | | | | | |
|  | | | | | | | | | | | | 1 |  | AT |  | 7/26/2020 10:38 AM |  |
|  | However, I have also seen people who may not give all the information and I think they have their reasons why they do that but like I said unless the patient doesn’t want a specific person to know then I ask them to kindly step out but I always tell my patients all the information that I have at present. | | | | | | | | | | | | | | | |  |
|  |  | | | | | | | | | | | | | | | |  |
|  | | | | | | | | | | | | 2 |  | AT |  | 7/26/2020 10:44 AM |  |
|  | I don’t think its very adequate just because mainly I would think about three factors number one is time the senior clinic where we see chemotherapy has so many patients. In my situation I think the new patient takes me between 45 minutes to one hour on a patient and its because there is so much to talk about, there is so much to find out, there is so much information to give but I don’t its very sufficient. | | | | | | | | | | | | | | | |  |
|  |  | | | | | | | | | | | | | | | |  |
|  | | | | | | | | | | | | | | | | | |
| Reports\\Coding Summary By Code Report | | | | | | | | | | Page 24 of 117 | | | | | | | |
| 8/15/2023 8:10 AM | | | | | | | | | | | | | | | | | |
|  | | | **Aggregate** |  | **Classification** |  | **Coverage** |  | **Number Of Coding References** | |  | **Reference Number** |  | **Coded By Initials** |  | **Modified On** |  |
|  | **Nodes\\Barriers to consenting or communicating information about Cancer prognosis to patients and its related effects\Informed conscent not cancer specific** | | | | | | | | | | | | | | | |  |
|  | | **Document** | | | | | | | | | | | | | | |  |
|  | | | **Files\\IDI - _ Doctor_UCI_06** | | | | | | | | | | | | | |  |
|  |  |  | No |  |  |  | 0.0161 |  | 1 | |  | | | | | |  |
|  | | |  |  |  |  |  |  |  | |  | | | | | | |
|  | | | | | | | | | | | | 1 |  | AT |  | 7/24/2020 8:53 AM |  |
|  | Normally this informed consent is obtained, its not cancer specific its for general purpose treatment when opening a file, there is a sheet generally about the care they are going to get and asking for permission but I must say that cancer specific consent forms are not generally but I will tell like when you are going to do surgery there is a separate consent form for a patient and that consent form again will give that information of what they are going to do, what possible complications may arise in future both immediate and after. | | | | | | | | | | | | | | | |  |
|  |  | | | | | | | | | | | | | | | |  |
|  | | | **Files\\IDI -Specialist palliative care_10** | | | | | | | | | | | | | |  |
|  |  |  | No |  |  |  | 0.0143 |  | 1 | |  | | | | | |  |
|  | | |  |  |  |  |  |  |  | |  | | | | | | |
|  | | | | | | | | | | | | 1 |  | AT |  | 7/24/2020 9:56 AM |  |
|  | But of course, because blanket consent is not specific to what to what you’re consenting for so if you agree to all treatments given and all procedures, what are the procedures because informed consent is supposed to be detailed about all treatments, their side effects and options and also same with procedures. I may be comfortable with these procedures but not all and yet I have accepted that everything will be done. so I think as much as that consent may be for admission or for specific things we should have other consents. | | | | | | | | | | | | | | | |  |
|  |  | | | | | | | | | | | | | | | |  |
|  | | | **Files\\IDI_ Nurse_UCI_02** | | | | | | | | | | | | | |  |
|  |  |  | No |  |  |  | 0.0063 |  | 1 | |  | | | | | |  |
|  | | |  |  |  |  |  |  |  | |  | | | | | | |
|  | | | | | | | | | | | | 1 |  | AT |  | 7/26/2020 9:33 AM |  |
|  | They don’t know because sometimes for me in my department we have surgeries so you tell them you’re supposed to consent for this procedure sometimes we used not to consent them but now we are just re consenting for the data. | | | | | | | | | | | | | | | |  |
|  |  | | | | | | | | | | | | | | | |  |
|  | | | | | | | | | | | | | | | | | |
|  | | | | | | | | | | | | | | | | | |
|  | | | | | | | | | | | | | | | | | |
| Reports\\Coding Summary By Code Report | | | | | | | | | | Page 25 of 117 | | | | | | | |
| 8/15/2023 8:10 AM | | | | | | | | | | | | | | | | | |
|  | | | **Aggregate** |  | **Classification** |  | **Coverage** |  | **Number Of Coding References** | |  | **Reference Number** |  | **Coded By Initials** |  | **Modified On** |  |
|  | **Nodes\\Barriers to consenting or communicating information about Cancer prognosis to patients and its related effects\Lack of confidence to disclose** | | | | | | | | | | | | | | | |  |
|  | | **Document** | | | | | | | | | | | | | | |  |
|  | | | **Files\\IDI -Specialist palliative care_10** | | | | | | | | | | | | | |  |
|  |  |  | No |  |  |  | 0.0116 |  | 1 | |  | | | | | |  |
|  | | |  |  |  |  |  |  |  | |  | | | | | | |
|  | | | | | | | | | | | | 1 |  | AT |  | 7/24/2020 9:42 AM |  |
|  | Of course there are times when people fear to give the information and they want someone else to do it so that’s where it will delay or not comfortable to give the information it happens a lot and I think that’s why sometimes people are referred to a team for disclosure of information because the initial person was not comfortable doing it. It happens a lot especially if patients have this profile political you know those things | | | | | | | | | | | | | | | |  |
|  |  | | | | | | | | | | | | | | | |  |
|  | **Nodes\\Barriers to consenting or communicating information about Cancer prognosis to patients and its related effects\Lack of designated person to offer information or informed consent** | | | | | | | | | | | | | | | |  |
|  | | **Document** | | | | | | | | | | | | | | |  |
|  | | | **Files\\IDI -Specialist palliative care_10** | | | | | | | | | | | | | |  |
|  |  |  | No |  |  |  | 0.0110 |  | 2 | |  | | | | | |  |
|  | | |  |  |  |  |  |  |  | |  | | | | | | |
|  | | | | | | | | | | | | 1 |  | AT |  | 7/24/2020 10:17 AM |  |
|  | unless in future there is a group of people who are specifically assigned to that otherwise if you are the same person or you have to go consent you have to do the procedure then you may only do a third of all, the ministry of works is supposed to do that. | | | | | | | | | | | | | | | |  |
|  |  | | | | | | | | | | | | | | | |  |
|  | | | | | | | | | | | | 2 |  | AT |  | 7/24/2020 10:22 AM |  |
|  | Apart from saying that we should have designated people should have ready forms, may be that will make us think about it more and not make it blanket. | | | | | | | | | | | | | | | |  |
|  |  | | | | | | | | | | | | | | | |  |
|  | | | **Files\\IDI_ Counselor_UCI_01** | | | | | | | | | | | | | |  |
|  |  |  | No |  |  |  | 0.0290 |  | 3 | |  | | | | | |  |
|  | | |  |  |  |  |  |  |  | |  | | | | | | |
|  | | | | | | | | | | | | 1 |  | AT |  | 7/25/2020 10:53 PM |  |
|  | I don’t know who is supposed to give the information about the treatment plan because if it is treatment plan the doctor is supposed to do that say we have prescribed for you this medication, you’re supposed to tell the patient this medication you take it like this. | | | | | | | | | | | | | | | |  |
|  |  | | | | | | | | | | | | | | | |  |
|  | | | | | | | | | | | | 2 |  | AT |  | 7/25/2020 10:54 PM |  |
|  | I don’t know who is supposed to do it. I could be missing out that and patients stay in darkness without proper information about treatment plan. It should be clear actually so that we know who is giving that information. | | | | | | | | | | | | | | | |  |
|  |  | | | | | | | | | | | | | | | |  |
|  | | | | | | | | | | | | 3 |  | AT |  | 7/25/2020 10:55 PM |  |
|  | It is not clear that this one is supposed to give this kind of information because I know health distributors are there, counsellors are there, nurses are there so I don’t who is supposed to, who is responsible for that but what I know is that every health worker is supposed to give care. | | | | | | | | | | | | | | | |  |
|  |  | | | | | | | | | | | | | | | |  |
| Reports\\Coding Summary By Code Report | | | | | | | | | | Page 26 of 117 | | | | | | | |
| 8/15/2023 8:10 AM | | | | | | | | | | | | | | | | | |
|  | | | **Aggregate** |  | **Classification** |  | **Coverage** |  | **Number Of Coding References** | |  | **Reference Number** |  | **Coded By Initials** |  | **Modified On** |  |
|  | | | **Files\\IDI_ Nurse_UCI_02** | | | | | | | | | | | | | |  |
|  |  |  | No |  |  |  | 0.0039 |  | 2 | |  | | | | | |  |
|  | | |  |  |  |  |  |  |  | |  | | | | | | |
|  | | | | | | | | | | | | 1 |  | AT |  | 7/26/2020 9:38 AM |  |
|  | The records personnel, they are the ones who make them sign. | | | | | | | | | | | | | | | |  |
|  |  | | | | | | | | | | | | | | | |  |
|  | | | | | | | | | | | | 2 |  | AT |  | 7/26/2020 9:39 AM |  |
|  | The nurses and the doctors. But any way I don’t know how they delegate duties. | | | | | | | | | | | | | | | |  |
|  |  | | | | | | | | | | | | | | | |  |
|  | **Nodes\\Barriers to consenting or communicating information about Cancer prognosis to patients and its related effects\lack of informed consent document** | | | | | | | | | | | | | | | |  |
|  | | **Document** | | | | | | | | | | | | | | |  |
|  | | | **Files\\IDI - - Doctor- UCI -08** | | | | | | | | | | | | | |  |
|  |  |  | No |  |  |  | 0.0064 |  | 1 | |  | | | | | |  |
|  | | |  |  |  |  |  |  |  | |  | | | | | | |
|  | | | | | | | | | | | | 1 |  | AT |  | 7/24/2020 8:19 AM |  |
|  | I think there is no much proper documented informed consent again they give, it is only that one the rest the just give them information, the only part I wouldn’t say informed consent. | | | | | | | | | | | | | | | |  |
|  |  | | | | | | | | | | | | | | | |  |
|  | | | **Files\\IDI -Specialist palliative care_10** | | | | | | | | | | | | | |  |
|  |  |  | No |  |  |  | 0.0274 |  | 4 | |  | | | | | |  |
|  | | |  |  |  |  |  |  |  | |  | | | | | | |
|  | | | | | | | | | | | | 1 |  | AT |  | 7/24/2020 10:13 AM |  |
|  | I think may be the limitations are that may be we don’t have forms where patients can sign for particular procedures so probably if we where ever of course we do it blanket consent if we where ever to be challenged in court then we would be in trouble | | | | | | | | | | | | | | | |  |
|  |  | | | | | | | | | | | | | | | |  |
|  | | | | | | | | | | | | 2 |  | AT |  | 7/24/2020 10:14 AM |  |
|  | I think so I think going forward it would be something important to put in place and also because many patients don’t know informed consent, they are not used to informed consent generally in practice in Uganda. | | | | | | | | | | | | | | | |  |
|  |  | | | | | | | | | | | | | | | |  |
|  | | | | | | | | | | | | 3 |  | AT |  | 7/24/2020 10:15 AM |  |
|  | when the husband comes, oh let me ask her mother then when the mother comes let me ask the father so you go in to circles so when you tell the to sign its like thy its very serious matter that its life and death and I think maybe that’s why this blanket consent was to demystify procedures but of course it can be dangerous when something goes wrong if you didn’t really have a written informed consent. | | | | | | | | | | | | | | | |  |
|  |  | | | | | | | | | | | | | | | |  |
|  | | | | | | | | | | | | 4 |  | AT |  | 7/24/2020 10:23 AM |  |
|  | Apart from saying that we should have designated people should have ready forms, may be that will make us think about it more and not make it blanket. | | | | | | | | | | | | | | | |  |
|  |  | | | | | | | | | | | | | | | |  |
|  | | | | | | | | | | | | | | | | | |
| Reports\\Coding Summary By Code Report | | | | | | | | | | Page 27 of 117 | | | | | | | |
| 8/15/2023 8:10 AM | | | | | | | | | | | | | | | | | |
|  | | | **Aggregate** |  | **Classification** |  | **Coverage** |  | **Number Of Coding References** | |  | **Reference Number** |  | **Coded By Initials** |  | **Modified On** |  |
|  | | | **Files\\IDI- - Health Educator- UCI-09** | | | | | | | | | | | | | |  |
|  |  |  | No |  |  |  | 0.0033 |  | 1 | |  | | | | | |  |
|  | | |  |  |  |  |  |  |  | |  | | | | | | |
|  | | | | | | | | | | | | 1 |  | AT |  | 7/25/2020 9:47 PM |  |
|  | Today cancer disease is more serious than HIV but because we do not have a protocol of how we do get people to know their condition? It is not straight there is no policy governing that so that’s something that needs to be explored. | | | | | | | | | | | | | | | |  |
|  |  | | | | | | | | | | | | | | | |  |
|  | | | **Files\\IDI_ Social worker_UCI_03** | | | | | | | | | | | | | |  |
|  |  |  | No |  |  |  | 0.0214 |  | 4 | |  | | | | | |  |
|  | | |  |  |  |  |  |  |  | |  | | | | | | |
|  | | | | | | | | | | | | 1 |  | AT |  | 7/26/2020 8:57 AM |  |
|  | patients don’t consent especially when it comes to opening a file out, that is the first place where they start, they come with their referral maybe when somebody has written there malignancy cancer, they will just open for you a file straight away, they send you to the clerking doctor, he will just tell you this is cancer, you need to do ABCD test then you start treatment and that is it no consent nothing. | | | | | | | | | | | | | | | |  |
|  |  | | | | | | | | | | | | | | | |  |
|  | | | | | | | | | | | | 2 |  | AT |  | 7/26/2020 8:57 AM |  |
|  | The yellow sheet about understanding the patient’s details, next of kin, it doesn’t talk about consenting. | | | | | | | | | | | | | | | |  |
|  |  | | | | | | | | | | | | | | | |  |
|  | | | | | | | | | | | | 3 |  | AT |  | 7/26/2020 8:57 AM |  |
|  | It doesn’t talk about anything to do with consenting, treatment but they just agree to disclose who they are, where they come from and who can be their next of kin to consent if there is anything so I don’t think that is related to cancer as cancer but this is just to know, who you are, where you come from, your occupation, your village, basically that. | | | | | | | | | | | | | | | |  |
|  |  | | | | | | | | | | | | | | | |  |
|  | | | | | | | | | | | | 4 |  | AT |  | 7/26/2020 9:00 AM |  |
|  | I have not seen them sign any form. | | | | | | | | | | | | | | | |  |
|  |  | | | | | | | | | | | | | | | |  |
|  | | | **Files\\IDI_ Nurse_UCI_02** | | | | | | | | | | | | | |  |
|  |  |  | No |  |  |  | 0.0247 |  | 4 | |  | | | | | |  |
|  | | |  |  |  |  |  |  |  | |  | | | | | | |
|  | | | | | | | | | | | | 1 |  | AT |  | 7/26/2020 9:32 AM |  |
|  | There I don’t think we go deep to explain because the common practice I see here, when you come we open up a file but during that process of opening up a file there is that part where you consent to every thing so you consent once and just tell patient that write your name. | | | | | | | | | | | | | | | |  |
|  |  | | | | | | | | | | | | | | | |  |
|  | | | | | | | | | | | | 2 |  | AT |  | 7/26/2020 9:33 AM |  |
|  | They don’t give them that whole information before they consent it’s just they give you a file here sign here, there and they don’t know what they are signing for. | | | | | | | | | | | | | | | |  |
|  |  | | | | | | | | | | | | | | | |  |
|  | | | | | | | | | | | | 3 |  | AT |  | 7/26/2020 9:34 AM |  |
|  | The yellow one is the one is the one they have been using for years, there is a different consent form that has just been introduced in the system recently because they could tell them you only consent for theatre but now it has just been introduced and it is not yet there perfectly we are like two months since September. | | | | | | | | | | | | | | | |  |
|  |  | | | | | | | | | | | | | | | |  |
|  | | | | | | | | | | | | 4 |  | AT |  | 7/26/2020 9:39 AM |  |
|  | But that thing is not informed consent yes the sheets say that informed consent form but I don’t think it is it. | | | | | | | | | | | | | | | |  |
|  |  | | | | | | | | | | | | | | | |  |
|  | | | | | | | | | | | | | | | | | |
| Reports\\Coding Summary By Code Report | | | | | | | | | | Page 28 of 117 | | | | | | | |
| 8/15/2023 8:10 AM | | | | | | | | | | | | | | | | | |
|  | | | **Aggregate** |  | **Classification** |  | **Coverage** |  | **Number Of Coding References** | |  | **Reference Number** |  | **Coded By Initials** |  | **Modified On** |  |
|  | | | **Files\\IDI_ _ Nurse_UCI_04** | | | | | | | | | | | | | |  |
|  |  |  | No |  |  |  | 0.0317 |  | 2 | |  | | | | | |  |
|  | | |  |  |  |  |  |  |  | |  | | | | | | |
|  | | | | | | | | | | | | 1 |  | AT |  | 7/26/2020 10:13 AM |  |
|  | On patients file opening informed consent is often obtained from them but the only thing about them of informed consent that is, I think it is not so much clearly explained to them and also during times….. | | | | | | | | | | | | | | | |  |
|  |  | | | | | | | | | | | | | | | |  |
|  | | | | | | | | | | | | 2 |  | AT |  | 7/26/2020 10:20 AM |  |
|  | That’s very interesting actually, I was going to explain that during the time of consent. The consent just happens, you’re consenting to be taken care of in terms of the different care when you go to specialized care because we don’t have those documents which are actually there for people to get to sign like for chemotherapy like I am signing that I am actually getting chemotherapy so your consenting towards getting that or you’re consenting towards getting like radiotherapy, you don’t know everything is just dependant on the initial consent which I think might need to look in. I think someone can be okay to be taken care of in the institute but might be certain to treatment modalities may not be okay with the person so it requires you again seeking the person’s consent. But we seek that verbally but not written. | | | | | | | | | | | | | | | |  |
|  |  | | | | | | | | | | | | | | | |  |
|  | | | **Files\\IDI__ Doctor_ UCI_ 05** | | | | | | | | | | | | | |  |
|  |  |  | No |  |  |  | 0.0199 |  | 3 | |  | | | | | |  |
|  | | |  |  |  |  |  |  |  | |  | | | | | | |
|  | | | | | | | | | | | | 1 |  | AT |  | 7/26/2020 10:45 AM |  |
|  | The reality is, in the clinic we don’t do informed consent. Informed consent is done when the file is being opened. | | | | | | | | | | | | | | | |  |
|  |  | | | | | | | | | | | | | | | |  |
|  | | | | | | | | | | | | 2 |  | AT |  | 7/26/2020 10:47 AM |  |
|  | I think there is room for negotiation and however the challenge with cancer treatment is for each cancer you have a protocol so the negotiation may be that you have breast cancer if you have stage one and stage 2 protocol is ABCD and do you agree to follow ABCD and then I will tell you depending on, if we follow ABCD our chances are going to be better or if we don’t follow ABCD will be compromised in terms of you having better outcomes but it is your choice, if you want to receive treatment it solely depends on you because you’re the patient and you have rights. | | | | | | | | | | | | | | | |  |
|  |  | | | | | | | | | | | | | | | |  |
|  | | | | | | | | | | | | 3 |  | AT |  | 7/26/2020 10:52 AM |  |
|  | No, we don’t have a specific form but we write on the clinic notes form and we put it in the file. | | | | | | | | | | | | | | | |  |
|  |  | | | | | | | | | | | | | | | |  |
|  | **Nodes\\Barriers to consenting or communicating information about Cancer prognosis to patients and its related effects\lack of social support for patient** | | | | | | | | | | | | | | | |  |
|  | | **Document** | | | | | | | | | | | | | | |  |
|  | | | **Files\\IDI - - Doctor- UCI -08** | | | | | | | | | | | | | |  |
|  |  |  | No |  |  |  | 0.0056 |  | 1 | |  | | | | | |  |
|  | | |  |  |  |  |  |  |  | |  | | | | | | |
|  | | | | | | | | | | | | 1 |  | AT |  | 7/24/2020 8:28 AM |  |
|  | others people go away and almost remain without support. They say this one has cancer there are even men who leave their wives and wives who leave their husbands. | | | | | | | | | | | | | | | |  |
|  |  | | | | | | | | | | | | | | | |  |
|  | | | | | | | | | | | | | | | | | |
| Reports\\Coding Summary By Code Report | | | | | | | | | | Page 29 of 117 | | | | | | | |
| 8/15/2023 8:10 AM | | | | | | | | | | | | | | | | | |
|  | | | **Aggregate** |  | **Classification** |  | **Coverage** |  | **Number Of Coding References** | |  | **Reference Number** |  | **Coded By Initials** |  | **Modified On** |  |
|  | | | **Files\\IDI -Specialist palliative care_10** | | | | | | | | | | | | | |  |
|  |  |  | No |  |  |  | 0.0225 |  | 2 | |  | | | | | |  |
|  | | |  |  |  |  |  |  |  | |  | | | | | | |
|  | | | | | | | | | | | | 1 |  | AT |  | 7/24/2020 10:10 AM |  |
|  | I think in our health care its not doing so much support given in other circles except mainly the physical issues and socially they are there. I don’t know how much social workers can do in this respect but I think it may not be too much but I know there is now a para legal group which helps patients in financial decisions for example if you are very sick and you may need your money from NSSF then they can help you get that money. | | | | | | | | | | | | | | | |  |
|  |  | | | | | | | | | | | | | | | |  |
|  | | | | | | | | | | | | 2 |  | AT |  | 7/24/2020 10:22 AM |  |
|  | Palliative care team is also given complex ones so because the palliative care team is not there full time, it’s a consultancy team, they will counsel the patients most of the time if they are complex or talk to them and discuss about prognosis but then the counsellor is a person who is on the ground most of the time and starts to continue with the process or brain folds what is already started. | | | | | | | | | | | | | | | |  |
|  |  | | | | | | | | | | | | | | | |  |
|  | | | **Files\\IDI_ _ Nurse_UCI_04** | | | | | | | | | | | | | |  |
|  |  |  | No |  |  |  | 0.0165 |  | 1 | |  | | | | | |  |
|  | | |  |  |  |  |  |  |  | |  | | | | | | |
|  | | | | | | | | | | | | 1 |  | AT |  | 7/26/2020 10:21 AM |  |
|  | I think not so much is really focused on to the social life, it might be the care but I think the social life is something which is there but it is not so much focused in and they tend to just ask the social workers to come on board but I think this could be something which could have been done on the initial visit or we needed to have done pyscho social kind of assessment whereby we test the patient and have the patient’s physical, social and psychological life so that is something that something that I think we don’t so much do. | | | | | | | | | | | | | | | |  |
|  |  | | | | | | | | | | | | | | | |  |
|  | **Nodes\\Barriers to consenting or communicating information about Cancer prognosis to patients and its related effects\Lack of time** | | | | | | | | | | | | | | | |  |
|  | | **Document** | | | | | | | | | | | | | | |  |
|  | | | **Files\\IDI - - Doctor- UCI -08** | | | | | | | | | | | | | |  |
|  |  |  | No |  |  |  | 0.0053 |  | 1 | |  | | | | | |  |
|  | | |  |  |  |  |  |  |  | |  | | | | | | |
|  | | | | | | | | | | | | 1 |  | AT |  | 7/24/2020 8:28 AM |  |
|  | First of all I think the biggest challenge is time of the health workers to administer informed consent because of too much work that is a big challenge. | | | | | | | | | | | | | | | |  |
|  |  | | | | | | | | | | | | | | | |  |
|  | | | **Files\\IDI - _ Doctor_UCI_06** | | | | | | | | | | | | | |  |
|  |  |  | No |  |  |  | 0.0194 |  | 2 | |  | | | | | |  |
|  | | |  |  |  |  |  |  |  | |  | | | | | | |
|  | | | | | | | | | | | | 1 |  | AT |  | 7/24/2020 8:51 AM |  |
|  | Informed consent is a very broad concept when it comes to diagnosis, your treatment plan, intended benefits of that treatment plan possible or side effects. It’s a big topic again the constraint of time vice versa the number of health personnel, | | | | | | | | | | | | | | | |  |
|  |  | | | | | | | | | | | | | | | |  |
| Reports\\Coding Summary By Code Report | | | | | | | | | | Page 30 of 117 | | | | | | | |
| 8/15/2023 8:10 AM | | | | | | | | | | | | | | | | | |
|  | | | **Aggregate** |  | **Classification** |  | **Coverage** |  | **Number Of Coding References** | |  | **Reference Number** |  | **Coded By Initials** |  | **Modified On** |  |
|  | | | | | | | | | | | | | | | | | |
|  | | | | | | | | | | | | 2 |  | AT |  | 7/24/2020 9:04 AM |  |
|  | We are pretty receptive to patients concerns and cares but I must also add again time constraints plus human resource, we are not adequate prepared to handle that because of the large patient numbers. We should be addressing many things and mental health, things like diet, all the side effects experienced and physiotherapy so there is a big limitation as far as man power and human resources is concerned. | | | | | | | | | | | | | | | |  |
|  |  | | | | | | | | | | | | | | | |  |
|  | | | **Files\\IDI -Specialist palliative care_10** | | | | | | | | | | | | | |  |
|  |  |  | No |  |  |  | 0.0421 |  | 4 | |  | | | | | |  |
|  | | |  |  |  |  |  |  |  | |  | | | | | | |
|  | | | | | | | | | | | | 1 |  | AT |  | 7/24/2020 9:40 AM |  |
|  | Some times its time issue, you know time factor, the patient is being referred and they are going home its difficult to talk, this is the first time you seeing them, they want to leave and then you don’t have that time because you will also want to break bad news but also support them that know you talking the bad news bad they are going home, | | | | | | | | | | | | | | | |  |
|  |  | | | | | | | | | | | | | | | |  |
|  | | | | | | | | | | | | 2 |  | AT |  | 7/24/2020 9:48 AM |  |
|  | many times there is not enough time to disclose the information and there are not enough opportunities to talk to patients because as I said that you cant give all the information in one go and therefore you have to find other subsequent opportunities to give the information but many times those opportunities may not be there because will not absorb all that information at once | | | | | | | | | | | | | | | |  |
|  |  | | | | | | | | | | | | | | | |  |
|  | | | | | | | | | | | | 3 |  | AT |  | 7/24/2020 9:57 AM |  |
|  | I can only speak for a small portion because sometimes its hard when a patient asks a colleague you may not be there so I don’t know how they really respond but sometimes when you hear from patients they feel that sometimes people don’t have time for them. Even when they ask them some questions, they do not respond to them. Sometimes they feel bad,but I don’t know if this is patients’ perception but I feel that people may be offended. | | | | | | | | | | | | | | | |  |
|  |  | | | | | | | | | | | | | | | |  |
|  | | | | | | | | | | | | 4 |  | AT |  | 7/24/2020 10:17 AM |  |
|  | I think that why its not happening in the proper way because if you were to do a real proper informed consent it needs enough time, we don’t even have enough time to sit down patients and talk about enough issues an give them chance to ask questions or given time to think about it, most of the time we want to finish the job because there are so many more coming on the ward so it is very difficult | | | | | | | | | | | | | | | |  |
|  |  | | | | | | | | | | | | | | | |  |
|  | | | **Files\\IDI- - Health Educator- UCI-09** | | | | | | | | | | | | | |  |
|  |  |  | No |  |  |  | 0.0128 |  | 3 | |  | | | | | |  |
|  | | |  |  |  |  |  |  |  | |  | | | | | | |
|  | | | | | | | | | | | | 1 |  | AT |  | 7/24/2020 10:31 AM |  |
|  | Either the doctor doesn’t have a lot of time or, because the workers are few because however is at a certain point is so much occupied that you cannot fulfil the other information needs of the patient so you find patients going through treatment four five cycles do not know why they are being treated, they don’t know the cancer conditions exist, do not the cancer cycle, why am I going to another stage so you find patients treatment plan is not known to many patients because of such. | | | | | | | | | | | | | | | |  |
|  |  | | | | | | | | | | | | | | | |  |
|  | | | | | | | | | | | | 2 |  | AT |  | 7/24/2020 12:38 PM |  |
|  | This patient may not have a chance to sit down with the nurse because the nurse has a hundred patients and she is the one to give information on the cancer you’re going through the type of treatment, | | | | | | | | | | | | | | | |  |
|  |  | | | | | | | | | | | | | | | |  |
|  | | | | | | | | | | | | 3 |  | AT |  | 7/25/2020 10:24 PM |  |
|  | The response is that most times that one: our staff are overwhelmed, they see additional questions from patients as waste of time, bothering them and of all things it’s not a priority as a package of service they need to offer, | | | | | | | | | | | | | | | |  |
|  |  | | | | | | | | | | | | | | | |  |
|  | | | | | | | | | | | | | | | | | |
| Reports\\Coding Summary By Code Report | | | | | | | | | | Page 31 of 117 | | | | | | | |
| 8/15/2023 8:10 AM | | | | | | | | | | | | | | | | | |
|  | | | **Aggregate** |  | **Classification** |  | **Coverage** |  | **Number Of Coding References** | |  | **Reference Number** |  | **Coded By Initials** |  | **Modified On** |  |
|  | | | **Files\\IDI_ Counselor_UCI_01** | | | | | | | | | | | | | |  |
|  |  |  | No |  |  |  | 0.0123 |  | 1 | |  | | | | | |  |
|  | | |  |  |  |  |  |  |  | |  | | | | | | |
|  | | | | | | | | | | | | 1 |  | AT |  | 7/25/2020 10:57 PM |  |
|  | Like I said before work load then two if the care takers they don’t want the patient to know then the doctor will not want to waste time trying to open up, convincing them like you have to spend with the patient 30min then you go in to counselling then to the counselling may take like an hour that is another point time constraint. | | | | | | | | | | | | | | | |  |
|  |  | | | | | | | | | | | | | | | |  |
|  | | | **Files\\IDI_ Nurse_UCI_02** | | | | | | | | | | | | | |  |
|  |  |  | No |  |  |  | 0.0026 |  | 1 | |  | | | | | |  |
|  | | |  |  |  |  |  |  |  | |  | | | | | | |
|  | | | | | | | | | | | | 1 |  | AT |  | 7/26/2020 9:36 AM |  |
|  | Like when approach a doctor and you ask them like you, go and ask that one or he is hurrying. | | | | | | | | | | | | | | | |  |
|  |  | | | | | | | | | | | | | | | |  |
|  | | | **Files\\IDI_ _ Nurse_UCI_04** | | | | | | | | | | | | | |  |
|  |  |  | No |  |  |  | 0.0250 |  | 1 | |  | | | | | |  |
|  | | |  |  |  |  |  |  |  | |  | | | | | | |
|  | | | | | | | | | | | | 1 |  | AT |  | 7/26/2020 10:00 AM |  |
|  | The clinicians don’t give them enough time to really express themselves and to really expound on these issues so they don’t have that much time because of those over whelming numbers. Then also some of them its actually because patients are there and when they don’t have attendants who are so inquisitive and wanting to know about the patients condition about the well being that can also affect the breaking of that information and then some of them you find that the person already has presented in and the person kind of lacks people around him or her to try to support him during that time of breaking that bad news. So, you look at it like you tell the person and the person is in denial so the next time the person is telling you what is my stage of disease and yet actually information has already been given. | | | | | | | | | | | | | | | |  |
|  |  | | | | | | | | | | | | | | | |  |
|  | **Nodes\\Barriers to consenting or communicating information about Cancer prognosis to patients and its related effects\lack of training** | | | | | | | | | | | | | | | |  |
|  | | **Document** | | | | | | | | | | | | | | |  |
|  | | | **Files\\IDI -Specialist palliative care_10** | | | | | | | | | | | | | |  |
|  |  |  | No |  |  |  | 0.0062 |  | 1 | |  | | | | | |  |
|  | | |  |  |  |  |  |  |  | |  | | | | | | |
|  | | | | | | | | | | | | 1 |  | AT |  | 7/24/2020 10:23 AM |  |
|  | I talked about training already when I said that consenting team do not who to consent, training is very essential and reminding people you know when you don’t practice everyday you forget so reminder courses all the time, ethics. | | | | | | | | | | | | | | | |  |
|  |  | | | | | | | | | | | | | | | |  |
|  | | | | | | | | | | | | | | | | | |
| Reports\\Coding Summary By Code Report | | | | | | | | | | Page 32 of 117 | | | | | | | |
| 8/15/2023 8:10 AM | | | | | | | | | | | | | | | | | |
|  | | | **Aggregate** |  | **Classification** |  | **Coverage** |  | **Number Of Coding References** | |  | **Reference Number** |  | **Coded By Initials** |  | **Modified On** |  |
|  | | | **Files\\IDI- - Health Educator- UCI-09** | | | | | | | | | | | | | |  |
|  |  |  | No |  |  |  | 0.0114 |  | 2 | |  | | | | | |  |
|  | | |  |  |  |  |  |  |  | |  | | | | | | |
|  | | | | | | | | | | | | 1 |  | AT |  | 7/25/2020 10:40 PM |  |
|  | The second challenge is providing information before consent some of the persons may not be technical to provide detailed information on the content of the consent form but also technical people who should have given this information accurately may be very few so at what point and how better can informed consent be given is a challenge, it’s a question which requires a little bit of critical analysis to come up with the way of doing that. | | | | | | | | | | | | | | | |  |
|  |  | | | | | | | | | | | | | | | |  |
|  | | | | | | | | | | | | 2 |  | AT |  | 7/25/2020 10:41 PM |  |
|  | So I have talked of three four things, there is a little bit of not knowing what of consent is from the perspective of health workers. And the implications if they knew it I am sure they will be doing things right. The I talked about the few numbers and the technicality of giving out information then I talked about communication bit of it which are very few challenges. | | | | | | | | | | | | | | | |  |
|  |  | | | | | | | | | | | | | | | |  |
|  | **Nodes\\Barriers to consenting or communicating information about Cancer prognosis to patients and its related effects\Language barrier** | | | | | | | | | | | | | | | |  |
|  | | **Document** | | | | | | | | | | | | | | |  |
|  | | | **Files\\IDI - _ Doctor_UCI_06** | | | | | | | | | | | | | |  |
|  |  |  | No |  |  |  | 0.0122 |  | 1 | |  | | | | | |  |
|  | | |  |  |  |  |  |  |  | |  | | | | | | |
|  | | | | | | | | | | | | 1 |  | AT |  | 7/24/2020 8:56 AM |  |
|  | That’s another limitation I must admit, they are all in English considering the literacy levels of population and the many languages and bearing in mind that we do not have a national language so they are mostly in English which is a limitation. So we do just summarize the form and put it in a local language for those who do not understand English but that will be verbative but the actual form is in English. | | | | | | | | | | | | | | | |  |
|  |  | | | | | | | | | | | | | | | |  |
|  | | | **Files\\IDI- - Health Educator- UCI-09** | | | | | | | | | | | | | |  |
|  |  |  | No |  |  |  | 0.0283 |  | 8 | |  | | | | | |  |
|  | | |  |  |  |  |  |  |  | |  | | | | | | |
|  | | | | | | | | | | | | 1 |  | AT |  | 7/24/2020 10:26 AM |  |
|  | Most patients do not know that because one either the doctor cannot share the language with the patient like the patient does not speak the language that the doctor speaks, two: sometimes these patients are so much in pain that even when the doctor tries to explain the cancer conditions and so forth, at that situation the patient cannot understand. | | | | | | | | | | | | | | | |  |
|  |  | | | | | | | | | | | | | | | |  |
|  | | | | | | | | | | | | 2 |  | AT |  | 7/25/2020 9:30 PM |  |
|  | Ok I told you the aspect of language, sometimes this doctor, this nurse may not be comfortable communicating with them because of the language | | | | | | | | | | | | | | | |  |
|  |  | | | | | | | | | | | | | | | |  |
|  | | | | | | | | | | | | 3 |  | AT |  | 7/25/2020 9:36 PM |  |
|  | I talked of the language can delay. | | | | | | | | | | | | | | | |  |
|  |  | | | | | | | | | | | | | | | |  |
|  | | | | | | | | | | | | 4 |  | AT |  | 7/25/2020 9:36 PM |  |
|  | Sometimes we don’t have a translator. | | | | | | | | | | | | | | | |  |
|  |  | | | | | | | | | | | | | | | |  |
| Reports\\Coding Summary By Code Report | | | | | | | | | | Page 33 of 117 | | | | | | | |
| 8/15/2023 8:10 AM | | | | | | | | | | | | | | | | | |
|  | | | **Aggregate** |  | **Classification** |  | **Coverage** |  | **Number Of Coding References** | |  | **Reference Number** |  | **Coded By Initials** |  | **Modified On** |  |
|  | | | | | | | | | | | | | | | | | |
|  | | | | | | | | | | | | 5 |  | AT |  | 7/25/2020 10:04 PM |  |
|  | You know this is a national cancer institute and we have very many languages in Uganda and outside so many times some patients cannot communicate or some doctors or service providers cannot communicate, two sides either the patients does not know the language or the doctor doesn’t know the language so they fail to communicate so there is need for an interpreter in between here, someone who can at least help in the communication so language barrier is a very big issue there. | | | | | | | | | | | | | | | |  |
|  |  | | | | | | | | | | | | | | | |  |
|  | | | | | | | | | | | | 6 |  | AT |  | 7/25/2020 10:05 PM |  |
|  | Majorly it is English, the consent form is written in English but most communication in UCI is in Luganda | | | | | | | | | | | | | | | |  |
|  |  | | | | | | | | | | | | | | | |  |
|  | | | | | | | | | | | | 7 |  | AT |  | 7/25/2020 10:20 PM |  |
|  | There are also some people we are finding that we are calling physical navigators, people who are able to move with patients around but we want people with different languages at least three local languages with national representation such that they will be acting as interpreters so where the doctor cannot communicate because of language barrier or the nurse cannot communicate we would be knowing this person knows his language , it’s a matter of calling this person, come at this point when we know then will now come and translate to this patient and back such that being delayed ends there. | | | | | | | | | | | | | | | |  |
|  |  | | | | | | | | | | | | | | | |  |
|  | | | | | | | | | | | | 8 |  | AT |  | 7/25/2020 10:21 PM |  |
|  | Yes, even if someone who stopped in senior four but can speak Luganda, lunyankole and English, another one speaks Acholi, Ateso and English the other one speaks let’s say from West Nile at least we would have narrowed down to about 75% of the language barrier. | | | | | | | | | | | | | | | |  |
|  |  | | | | | | | | | | | | | | | |  |
|  | | | **Files\\IDI_ Social worker_UCI_03** | | | | | | | | | | | | | |  |
|  |  |  | No |  |  |  | 0.0209 |  | 2 | |  | | | | | |  |
|  | | |  |  |  |  |  |  |  | |  | | | | | | |
|  | | | | | | | | | | | | 1 |  | AT |  | 7/26/2020 8:58 AM |  |
|  | I think there are some who have language barrier and those ones even doctors struggle with them and they don’t bother to find somebody to interpret for such patients, for such patients the decision is purely for the clinical team but they only struggle to tell the patient, do ABCD this test come tomorrow and continue. Then after that you are going to be operated and then after that you are going to start treatment. | | | | | | | | | | | | | | | |  |
|  |  | | | | | | | | | | | | | | | |  |
|  | | | | | | | | | | | | 2 |  | AT |  | 7/26/2020 9:08 AM |  |
|  | Two, their level of understanding, you know cancer is also difficult to understand bringing it in Luganda much as they have translated to ankokolo whatever but I am telling you, the treatment and other things is difficult to explain to other people to understand and also to understand the detail of the prognosis, how much is advancing internally, how much it eating her where it has reached and understanding clearly the picture of the disease and the physical body. | | | | | | | | | | | | | | | |  |
|  |  | | | | | | | | | | | | | | | |  |
|  | | | **Files\\IDI_ _ Nurse_UCI_04** | | | | | | | | | | | | | |  |
|  |  |  | No |  |  |  | 0.0260 |  | 2 | |  | | | | | |  |
|  | | |  |  |  |  |  |  |  | |  | | | | | | |
|  | | | | | | | | | | | | 1 |  | AT |  | 7/26/2020 10:15 AM |  |
|  | Its mainly English that is channeled to be used and sometimes Luganda but I think the society that we are in there are so many languages that come inside us so I know that is something that is kind of missing and not all the languages are captured but you find mainly like English is captured and maybe Luganda. | | | | | | | | | | | | | | | |  |
|  |  | | | | | | | | | | | | | | | |  |
|  | | | | | | | | | | | | 2 |  | AT |  | 7/26/2020 10:25 AM |  |
|  | The other challenge can be language barrier for people for the people and the language they are able to speak is language barrier.some of people who come may not be Ugandans and the language that they are speaking you cannot speak and in a way of explaining to that person in that informed consent is very difficult. Mainly within the cancer institute those have been big issues in line with lack of enough informed consent. You don’t have enough information, you don have skilled personnel, language barriers have not really enabled. | | | | | | | | | | | | | | | |  |
|  |  | | | | | | | | | | | | | | | |  |
|  | | | | | | | | | | | | | | | | | |
| Reports\\Coding Summary By Code Report | | | | | | | | | | Page 34 of 117 | | | | | | | |
| 8/15/2023 8:10 AM | | | | | | | | | | | | | | | | | |
|  | | | **Aggregate** |  | **Classification** |  | **Coverage** |  | **Number Of Coding References** | |  | **Reference Number** |  | **Coded By Initials** |  | **Modified On** |  |
|  | | | **Files\\IDI__ Doctor_ UCI_ 05** | | | | | | | | | | | | | |  |
|  |  |  | No |  |  |  | 0.0144 |  | 3 | |  | | | | | |  |
|  | | |  |  |  |  |  |  |  | |  | | | | | | |
|  | | | | | | | | | | | | 1 |  | AT |  | 7/26/2020 10:36 AM |  |
|  | Usually the biggest hinderance is language so unless I have a patient from my country and I don’t understand their language may be but still we usually have someone who comes along with them. But a patient with whom we speak English or Luganda we understand each other they usually open and they really want to know. | | | | | | | | | | | | | | | |  |
|  |  | | | | | | | | | | | | | | | |  |
|  | | | | | | | | | | | | 2 |  | AT |  | 7/26/2020 10:45 AM |  |
|  | Those you speak the same language and then there are those who don’t speak the same language. | | | | | | | | | | | | | | | |  |
|  |  | | | | | | | | | | | | | | | |  |
|  | | | | | | | | | | | | 3 |  | AT |  | 7/26/2020 11:00 AM |  |
|  | But me I think the challenge is more on time but even the language because the informed consent is only in English am not sure, I think it’s only English. | | | | | | | | | | | | | | | |  |
|  |  | | | | | | | | | | | | | | | |  |
|  | **Nodes\\Barriers to consenting or communicating information about Cancer prognosis to patients and its related effects\Limited human resource** | | | | | | | | | | | | | | | |  |
|  | | **Document** | | | | | | | | | | | | | | |  |
|  | | | **Files\\IDI - _ Doctor_UCI_06** | | | | | | | | | | | | | |  |
|  |  |  | No |  |  |  | 0.0307 |  | 4 | |  | | | | | |  |
|  | | |  |  |  |  |  |  |  | |  | | | | | | |
|  | | | | | | | | | | | | 1 |  | AT |  | 7/24/2020 8:51 AM |  |
|  | Informed consent is a very broad concept when it comes to diagnosis, your treatment plan, intended benefits of that treatment plan possible or side effects. It’s a big topic again the constraint of time vice versa the number of health personnel, | | | | | | | | | | | | | | | |  |
|  |  | | | | | | | | | | | | | | | |  |
|  | | | | | | | | | | | | 2 |  | AT |  | 7/24/2020 9:04 AM |  |
|  | We are pretty receptive to patients concerns and cares but I must also add again time constraints plus human resource, we are not adequate prepared to handle that because of the large patient numbers. We should be addressing many things and mental health, things like diet, all the side effects experienced and physiotherapy so there is a big limitation as far as man power and human resources is concerned. | | | | | | | | | | | | | | | |  |
|  |  | | | | | | | | | | | | | | | |  |
|  | | | | | | | | | | | | 3 |  | AT |  | 7/24/2020 9:10 AM |  |
|  | The challenges are: we are few clinicians really compared to he numbers of patients we are seeing so we don’t adequately give all the information we would have wanted to give, we given basic information and when you give basic information you leave out certain aspects which later may interfere with the patient’s treatment so that is a big challenge. | | | | | | | | | | | | | | | |  |
|  |  | | | | | | | | | | | | | | | |  |
|  | | | | | | | | | | | | 4 |  | AT |  | 7/24/2020 9:10 AM |  |
|  | Yeah, human resource is one, | | | | | | | | | | | | | | | |  |
|  |  | | | | | | | | | | | | | | | |  |
|  | | | **Files\\IDI -Specialist palliative care_10** | | | | | | | | | | | | | |  |
|  |  |  | No |  |  |  | 0.0036 |  | 1 | |  | | | | | |  |
|  | | |  |  |  |  |  |  |  | |  | | | | | | |
|  | | | | | | | | | | | | 1 |  | AT |  | 7/24/2020 10:17 AM |  |
|  | I think that’s the main issue and also I think it may be difficult consenting everyone, every time given the numbers and the few staff, | | | | | | | | | | | | | | | |  |
|  |  | | | | | | | | | | | | | | | |  |
| Reports\\Coding Summary By Code Report | | | | | | | | | | Page 35 of 117 | | | | | | | |
| 8/15/2023 8:10 AM | | | | | | | | | | | | | | | | | |
|  | | | **Aggregate** |  | **Classification** |  | **Coverage** |  | **Number Of Coding References** | |  | **Reference Number** |  | **Coded By Initials** |  | **Modified On** |  |
|  | | | **Files\\IDI- - Health Educator- UCI-09** | | | | | | | | | | | | | |  |
|  |  |  | No |  |  |  | 0.0113 |  | 3 | |  | | | | | |  |
|  | | |  |  |  |  |  |  |  | |  | | | | | | |
|  | | | | | | | | | | | | 1 |  | AT |  | 7/24/2020 10:28 AM |  |
|  | Because of the high number of patients even nurses do not have time to give additional information and because of the few staff like we have only one health educator for the entire patients so patients do not have a chance to listen or to hear personal information finally they are very few if at all there are no materials where patients can access to read more about their condition so they go to search on the net which sometimes gives false information so generally information given to patients is really scanty, its inadequate and not coordinated in a way. | | | | | | | | | | | | | | | |  |
|  |  | | | | | | | | | | | | | | | |  |
|  | | | | | | | | | | | | 2 |  | AT |  | 7/25/2020 9:51 PM |  |
|  | I say that the challenge is we have many patients and the doctors are few. | | | | | | | | | | | | | | | |  |
|  |  | | | | | | | | | | | | | | | |  |
|  | | | | | | | | | | | | 3 |  | AT |  | 7/25/2020 10:24 PM |  |
|  | we gain nurses by the number of patients they have given chemotherapy, doctors: by the number of patients they have clerked information is not one measure of performance. | | | | | | | | | | | | | | | |  |
|  |  | | | | | | | | | | | | | | | |  |
|  | | | **Files\\IDI_ _ Nurse_UCI_04** | | | | | | | | | | | | | |  |
|  |  |  | No |  |  |  | 0.0068 |  | 1 | |  | | | | | |  |
|  | | |  |  |  |  |  |  |  | |  | | | | | | |
|  | | | | | | | | | | | | 1 |  | AT |  | 7/26/2020 10:25 AM |  |
|  | One of the challenges of informed consent is first of all, lack of enough skilled personnel to do the work, lack of information on the benefits of informed consent which is actually affecting the informed consent process. | | | | | | | | | | | | | | | |  |
|  |  | | | | | | | | | | | | | | | |  |
|  | **Nodes\\Barriers to consenting or communicating information about Cancer prognosis to patients and its related effects\Limited IEC materials** | | | | | | | | | | | | | | | |  |
|  | | **Document** | | | | | | | | | | | | | | |  |
|  | | | **Files\\IDI -Specialist palliative care_10** | | | | | | | | | | | | | |  |
|  |  |  | No |  |  |  | 0.0039 |  | 1 | |  | | | | | |  |
|  | | |  |  |  |  |  |  |  | |  | | | | | | |
|  | | | | | | | | | | | | 1 |  | AT |  | 7/24/2020 9:55 AM |  |
|  | I don’t think they are there, they may not be used much as they are supposed to be used, we have to get into the habit more of giving out those. | | | | | | | | | | | | | | | |  |
|  |  | | | | | | | | | | | | | | | |  |
|  | | | | | | | | | | | | | | | | | |
|  | | | | | | | | | | | | | | | | | |
| Reports\\Coding Summary By Code Report | | | | | | | | | | Page 36 of 117 | | | | | | | |
| 8/15/2023 8:10 AM | | | | | | | | | | | | | | | | | |
|  | | | **Aggregate** |  | **Classification** |  | **Coverage** |  | **Number Of Coding References** | |  | **Reference Number** |  | **Coded By Initials** |  | **Modified On** |  |
|  | | | **Files\\IDI_ Social worker_UCI_03** | | | | | | | | | | | | | |  |
|  |  |  | No |  |  |  | 0.0228 |  | 1 | |  | | | | | |  |
|  | | |  |  |  |  |  |  |  | |  | | | | | | |
|  | | | | | | | | | | | | 1 |  | AT |  | 7/26/2020 9:00 AM |  |
|  | They are not utilized it is because of time especially these books that cancer society has given, hose books are there because I personally has picked one when a care giver came in our office, I was talking to her when I was asking her, do you understand fairer the disease that your daughter is having? Then she said this is the question I am battling with I don’t understand it very well, I was only told she has cancer of the uterus but I have questions that have no answers, then I asked what are the questions, she said what brings this cancer then I pull this book then I asked her can you read Luganda she said yes then I picked the pink one and the blue one I gave her, as she was still seated there we were still writing her referral form for hostel she was already going through a few of them, she immediately said this is what I wanted to know then she said thank you so much for giving me this book, she started appreciating me and promised to read the book | | | | | | | | | | | | | | | |  |
|  |  |  |  |  |  |  |  |  |  |  |  |  |  |  |  |  |  |
|  |  | | | | | | | | | | | | | | | |  |
|  | | | **Files\\IDI_ Nurse_UCI_02** | | | | | | | | | | | | | |  |
|  |  |  | No |  |  |  | 0.093 |  | 3 | |  | | | | | |  |
|  | | |  |  |  |  |  |  |  | |  | | | | | | |
|  | | | | | | | | | | | | 1 |  | AT |  | 7/26/2020 9:40 AM |  |
|  | we don’t have, there are no materials used. | | | | | | | | | | | | | | | |  |
|  |  | | | | | | | | | | | | | | | |  |
|  | | | | | | | | | | | | 2 |  | AT |  | 7/26/2020 9:40 AM |  |
|  | There were TVs but with no information about cancer on those TVs not until recently with in like four months some plays. | | | | | | | | | | | | | | | |  |
|  |  | | | | | | | | | | | | | | | |  |
|  | | | | | | | | | | | | 3 |  | AT |  | 7/26/2020 9:41 AM |  |
|  | Not so active because I have seen even in the other building there are just pictures, those TVs are there but not having patient’s information regarding cancer. | | | | | | | | | | | | | | | |  |
|  |  | | | | | | | | | | | | | | | |  |
|  | | | **Files\\IDI_ _ Nurse_UCI_04** | | | | | | | | | | | | | |  |
|  |  |  | No |  |  |  | 0.0124 |  | 3 | |  | | | | | |  |
|  | | |  |  |  |  |  |  |  | |  | | | | | | |
|  | | | | | | | | | | | | 1 |  | AT |  | 7/26/2020 10:18 AM |  |
|  | there are some materials that are actually available but I think they are very inadequate, they are available but not so much. | | | | | | | | | | | | | | | |  |
|  |  | | | | | | | | | | | | | | | |  |
|  | | | | | | | | | | | | 2 |  | AT |  | 7/26/2020 10:18 AM |  |
|  | They may be materials of the patient like patient’s information on cancer or taking care of a cancer patient, they may be in books, some are in leaflets but they are very inadequate. | | | | | | | | | | | | | | | |  |
|  |  | | | | | | | | | | | | | | | |  |
|  | | | | | | | | | | | | 3 |  | AT |  | 7/26/2020 10:18 AM |  |
|  | They are not frequently being used, actually I could say they are not frequently being used. | | | | | | | | | | | | | | | |  |
|  |  | | | | | | | | | | | | | | | |  |
|  | | | | | | | | | | | | | | | | | |
|  | | | | | | | | | | | | | | | | | |
| Reports\\Coding Summary By Code Report | | | | | | | | | | Page 37 of 117 | | | | | | | |
| 8/15/2023 8:10 AM | | | | | | | | | | | | | | | | | |
|  | | | **Aggregate** |  | **Classification** |  | **Coverage** |  | **Number Of Coding References** | |  | **Reference Number** |  | **Coded By Initials** |  | **Modified On** |  |
|  | **Nodes\\Barriers to consenting or communicating information about Cancer prognosis to patients and its related effects\Limited patient decision making, doctor decides** | | | | | | | | | | | | | | | |  |
|  | | **Document** | | | | | | | | | | | | | | |  |
|  | | | **Files\\IDI - _ Doctor_UCI_06** | | | | | | | | | | | | | |  |
|  |  |  | No |  |  |  | 0.0112 |  | 1 | |  | | | | | |  |
|  | | |  |  |  |  |  |  |  | |  | | | | | | |
|  | | | | | | | | | | | | 1 |  | AT |  | 7/24/2020 8:57 AM |  |
|  | Yes, that comes out of the information you have given them, if tell them you have an option of radiotherapy, chemotherapy or no treatment then they can opt to choose but may times they will say you choose for me the best and that’s on the basis of information but sometimes that poses some challenges for us because we are supposed to give information and the patient selects. | | | | | | | | | | | | | | | |  |
|  |  | | | | | | | | | | | | | | | |  |
|  | | | **Files\\IDI -Specialist palliative care_10** | | | | | | | | | | | | | |  |
|  |  |  | No |  |  |  | 0.0239 |  | 3 | |  | | | | | |  |
|  | | |  |  |  |  |  |  |  | |  | | | | | | |
|  | | | | | | | | | | | | 1 |  | AT |  | 7/24/2020 9:53 AM |  |
|  | But may be a brief on what the criteria intail, its not like giving a patient a chance to say that there options I don’t know am asking questions, it kind of giving information and expecting a positive response so I think that is a thing that is implied. | | | | | | | | | | | | | | | |  |
|  |  | | | | | | | | | | | | | | | |  |
|  | | | | | | | | | | | | 2 |  | AT |  | 7/24/2020 9:53 AM |  |
|  | Its not so common for us to say what do you think, what would be your options or giving them multiple options. Checking out options. | | | | | | | | | | | | | | | |  |
|  |  | | | | | | | | | | | | | | | |  |
|  | | | | | | | | | | | | 3 |  | AT |  | 7/24/2020 9:54 AM |  |
|  | Yes, they fear to ask questions, I think patients are now bold enough with more education but there are more patients who still have his attitude that doctors know best and therefore I cant challenge the doctor and the nurse, they fear that if I over challenge I will be in trouble so they know that if I want this treatment so I think may be in our communication what we can do is just to let them know that we are free to make better decisions and ask questions that its not only us to decide. | | | | | | | | | | | | | | | |  |
|  |  | | | | | | | | | | | | | | | |  |
|  | | | **Files\\IDI- - Health Educator- UCI-09** | | | | | | | | | | | | | |  |
|  |  |  | No |  |  |  | 0.045 |  | 1 | |  | | | | | |  |
|  | | |  |  |  |  |  |  |  | |  | | | | | | |
|  | | | | | | | | | | | | 1 |  | AT |  | 7/24/2020 12:48 PM |  |
|  | No, patients have to go through to the senior doctor, that is when now the senior doctor will make a decision sometimes the decision may go beyond the senior doctor there is a tumor board when the doctor is not here, this patient is referred to the tumor board so the now decides on what should happen to this patient. | | | | | | | | | | | | | | | |  |
|  |  | | | | | | | | | | | | | | | |  |
|  | | | | | | | | | | | | | | | | | |
|  | | | | | | | | | | | | | | | | | |
| Reports\\Coding Summary By Code Report | | | | | | | | | | Page 38 of 117 | | | | | | | |
| 8/15/2023 8:10 AM | | | | | | | | | | | | | | | | | |
|  | | | **Aggregate** |  | **Classification** |  | **Coverage** |  | **Number Of Coding References** | |  | **Reference Number** |  | **Coded By Initials** |  | **Modified On** |  |
|  | **Nodes\\Barriers to consenting or communicating information about Cancer prognosis to patients and its related effects\Limted space that hinders privacy** | | | | | | | | | | | | | | | |  |
|  | | **Document** | | | | | | | | | | | | | | |  |
|  | | | **Files\\IDI - _ Doctor_UCI_06** | | | | | | | | | | | | | |  |
|  |  |  | No |  |  |  | 0.089 |  | 1 | |  | | | | | |  |
|  | | |  |  |  |  |  |  |  | |  | | | | | | |
|  | | | | | | | | | | | | 1 |  | AT |  | 7/24/2020 9:18 AM |  |
|  | two we would have wanted to have more clinic space for the numbers of patients, bigger rooms more specious where you can sit and have a full privacy also before we mention the full patients mental state some patients come when they are fearful, you are not able to start it right away you have to wait. | | | | | | | | | | | | | | | |  |
|  |  | | | | | | | | | | | | | | | |  |
|  | **Nodes\\Barriers to consenting or communicating information about Cancer prognosis to patients and its related effects\Long ques** | | | | | | | | | | | | | | | |  |
|  | | **Document** | | | | | | | | | | | | | | |  |
|  | | | **Files\\IDI_ Nurse_UCI_02** | | | | | | | | | | | | | |  |
|  |  |  | No |  |  |  | 0.0003 |  | 1 | |  | | | | | |  |
|  | | |  |  |  |  |  |  |  | |  | | | | | | |
|  | | | | | | | | | | | | 1 |  | AT |  | 7/26/2020 9:35 AM |  |
|  | The long que. | | | | | | | | | | | | | | | |  |
|  |  | | | | | | | | | | | | | | | |  |
|  | **Nodes\\Barriers to consenting or communicating information about Cancer prognosis to patients and its related effects\Lost to follow up** | | | | | | | | | | | | | | | |  |
|  | | **Document** | | | | | | | | | | | | | | |  |
|  | | | **Files\\IDI_ Nurse_UCI_07** | | | | | | | | | | | | | |  |
|  |  |  | No |  |  |  | 0.0039 |  | 1 | |  | | | | | |  |
|  | | |  |  |  |  |  |  |  | |  | | | | | | |
|  | | | | | | | | | | | | 1 |  | AT |  | 7/26/2020 8:28 AM |  |
|  | They get lost to follow up yes for immediate disclosure some patients are really in shock and they take some time to come back so they get lost. | | | | | | | | | | | | | | | |  |
|  |  | | | | | | | | | | | | | | | |  |
|  | | | | | | | | | | | | | | | | | |
| Reports\\Coding Summary By Code Report | | | | | | | | | | Page 39 of 117 | | | | | | | |
| 8/15/2023 8:10 AM | | | | | | | | | | | | | | | | | |
|  | | | **Aggregate** |  | **Classification** |  | **Coverage** |  | **Number Of Coding References** | |  | **Reference Number** |  | **Coded By Initials** |  | **Modified On** |  |
|  | | | **Files\\IDI_ Social worker_UCI_03** | | | | | | | | | | | | | |  |
|  |  |  | No |  |  |  | 0.0113 |  | 2 | |  | | | | | |  |
|  | | |  |  |  |  |  |  |  | |  | | | | | | |
|  | | | | | | | | | | | | 1 |  | AT |  | 7/26/2020 8:46 AM |  |
|  | One, it has caused divorce in at least a number of them, you they go back and then somebody will just say I want to go home to try and look for money then send reaching home then the phone is off you call in vain or keep saying am looking for money am going to send thy become resistant. | | | | | | | | | | | | | | | |  |
|  |  | | | | | | | | | | | | | | | |  |
|  | | | | | | | | | | | | 2 |  | AT |  | 7/26/2020 8:48 AM |  |
|  | No, we are explaining that, after that, that guy of that family will go then the patient now come back since then he has gone and has not come back so we are calling his number then we try also. | | | | | | | | | | | | | | | |  |
|  |  | | | | | | | | | | | | | | | |  |
|  | **Nodes\\Barriers to consenting or communicating information about Cancer prognosis to patients and its related effects\Missed procedures** | | | | | | | | | | | | | | | |  |
|  | | **Document** | | | | | | | | | | | | | | |  |
|  | | | **Files\\IDI- - Health Educator- UCI-09** | | | | | | | | | | | | | |  |
|  |  |  | No |  |  |  | 0.0052 |  | 1 | |  | | | | | |  |
|  | | |  |  |  |  |  |  |  | |  | | | | | | |
|  | | | | | | | | | | | | 1 |  | AT |  | 7/24/2020 12:47 PM |  |
|  | That’s a very big problem, why? Because if you see that really this one, the junior doctor has done the part didn’t clerk this patient and says we need this and this kind of test that we will have the doctor judge you and get the stage of the disease and the treatment begin. Now this is already out and this person will not go to the junior doctor again for clerking no. | | | | | | | | | | | | | | | |  |
|  |  | | | | | | | | | | | | | | | |  |
|  | **Nodes\\Barriers to consenting or communicating information about Cancer prognosis to patients and its related effects\Myths and misconceptions about cancer** | | | | | | | | | | | | | | | |  |
|  | | **Document** | | | | | | | | | | | | | | |  |
|  | | | **Files\\IDI - _ Doctor_UCI_06** | | | | | | | | | | | | | |  |
|  |  |  | No |  |  |  | 0.0109 |  | 1 | |  | | | | | |  |
|  | | |  |  |  |  |  |  |  | |  | | | | | | |
|  | | | | | | | | | | | | 1 |  | AT |  | 7/24/2020 8:59 AM |  |
|  | There is this actual fear of doubt of the disease or communication or being told the truth but there are also these fears that are informal by society with culture about that or to be told if you have cancer its bad luck or there is something wrong with you. So instead of them coming to the hospital, they prefer to stay or go to alternative places or witch doctors. | | | | | | | | | | | | | | | |  |
|  |  | | | | | | | | | | | | | | | |  |
|  | | | | | | | | | | | | | | | | | |
| Reports\\Coding Summary By Code Report | | | | | | | | | | Page 40 of 117 | | | | | | | |
| 8/15/2023 8:10 AM | | | | | | | | | | | | | | | | | |
|  | | | **Aggregate** |  | **Classification** |  | **Coverage** |  | **Number Of Coding References** | |  | **Reference Number** |  | **Coded By Initials** |  | **Modified On** |  |
|  | **Nodes\\Barriers to consenting or communicating information about Cancer prognosis to patients and its related effects\Negative attitude toward Chemotherapy** | | | | | | | | | | | | | | | |  |
|  | | **Document** | | | | | | | | | | | | | | |  |
|  | | | **Files\\IDI -Specialist palliative care_10** | | | | | | | | | | | | | |  |
|  |  |  | No |  |  |  | 0.0208 |  | 1 | |  | | | | | |  |
|  | | |  |  |  |  |  |  |  | |  | | | | | | |
|  | | | | | | | | | | | | 1 |  | AT |  | 7/24/2020 9:57 AM |  |
|  | Yeah, even chemo in treatment because people have their beliefs about treatment and I think part of the reasons why people disappear and don’t see them again its because they believe they are going to be forced to have treatments once they sign, they know that they have an option or at least if they can they have an option of opting out then they will stay I think and from there they may have a discussion with someone who will make them understand what chemo is and isn’t based on the mix up they may have with it or particular treatments so its good to explain these treatments and whatever we are giving and some one knows they are getting this treatment, its side effects and then they truly consent to their treatment having known what its problems and benefits are. | | | | | | | | | | | | | | | |  |
|  |  | | | | | | | | | | | | | | | |  |
|  | **Nodes\\Barriers to consenting or communicating information about Cancer prognosis to patients and its related effects\Negative reaction from news about Cancer** | | | | | | | | | | | | | | | |  |
|  | | **Document** | | | | | | | | | | | | | | |  |
|  | | | **Files\\IDI - _ Doctor_UCI_06** | | | | | | | | | | | | | |  |
|  |  |  | No |  |  |  | 0.0412 |  | 3 | |  | | | | | |  |
|  | | |  |  |  |  |  |  |  | |  | | | | | | |
|  | | | | | | | | | | | | 1 |  | AT |  | 7/24/2020 9:05 AM |  |
|  | We always think about that because most times its in the negatives, sometimes someone is told they do have cancer and the stage is advanced. The first symptom that you get is a mood shift they usually change and the next visit you see a patient, they have totally come to terms no patient has taken it positively wit good vibes that oh I have cancer I am going to heal very quickly and everything no, most times they get that initial shock reaction almost 95% go through some sort of depression and depression your sleep is affected, diet is affected your social life is affected all in the negative so yes it’s a big factor. | | | | | | | | | | | | | | | |  |
|  |  | | | | | | | | | | | | | | | |  |
|  | | | | | | | | | | | | 2 |  | AT |  | 7/24/2020 9:06 AM |  |
|  | Because of the mental changes, the actual treatment itself is hard chemotherapy, radiotherapy surgery, their quality of life is reduced they are not able to do all that they would have wanted to do, they are less productive at work. So yes, the economic wellbeing changes and also let’s remember that while the cancer institute provides most of the drugs, there are times when there are certain medications that we need and the patient has got to buy them out of pocket so that also places an extra burden on them. | | | | | | | | | | | | | | | |  |
|  |  | | | | | | | | | | | | | | | |  |
|  | | | | | | | | | | | | 3 |  | AT |  | 7/24/2020 9:06 AM |  |
|  | The majority of the family it’s a hard effect knowing that their loved one has been diagnosed with cancer so the family is also affected. And you can see that quite a number of them are depressed about it so it negatively affects the family. | | | | | | | | | | | | | | | |  |
|  |  | | | | | | | | | | | | | | | |  |
|  | | | | | | | | | | | | | | | | | |
|  | | | | | | | | | | | | | | | | | |
| Reports\\Coding Summary By Code Report | | | | | | | | | | Page 41 of 117 | | | | | | | |
| 8/15/2023 8:10 AM | | | | | | | | | | | | | | | | | |
|  | | | **Aggregate** |  | **Classification** |  | **Coverage** |  | **Number Of Coding References** | |  | **Reference Number** |  | **Coded By Initials** |  | **Modified On** |  |
|  | **Nodes\\Barriers to consenting or communicating information about Cancer prognosis to patients and its related effects\patient lack of knowldge about Informed concent** | | | | | | | | | | | | | | | |  |
|  | | **Document** | | | | | | | | | | | | | | |  |
|  | | | **Files\\IDI - - Doctor- UCI -08** | | | | | | | | | | | | | |  |
|  |  |  | No |  |  |  | 0.0102 |  | 1 | |  | | | | | |  |
|  | | |  |  |  |  |  |  |  | |  | | | | | | |
|  | | | | | | | | | | | | 1 |  | AT |  | 7/24/2020 8:18 AM |  |
|  | Now there are times when they don’t know about informed consent, you know when you’re opening a file, there is what they call informed consent which is in most cases not done right, they don’t give the patients you first read this then you come, they just say you sign here or put your hand here. | | | | | | | | | | | | | | | |  |
|  |  | | | | | | | | | | | | | | | |  |
|  | | | **Files\\IDI- - Health Educator- UCI-09** | | | | | | | | | | | | | |  |
|  |  |  | No |  |  |  | 0.0033 |  | 1 | |  | | | | | |  |
|  | | |  |  |  |  |  |  |  | |  | | | | | | |
|  | | | | | | | | | | | | 1 |  | AT |  | 7/25/2020 10:42 PM |  |
|  | May be the other challenge I would address previously is there is no guideline on how to give informed consent, how when and to who. But is it there anyway or they are not aware, either the policies are there and people are not aware. | | | | | | | | | | | | | | | |  |
|  |  | | | | | | | | | | | | | | | |  |
|  | **Nodes\\Barriers to consenting or communicating information about Cancer prognosis to patients and its related effects\Patient’s low understanding** | | | | | | | | | | | | | | | |  |
|  | | **Document** | | | | | | | | | | | | | | |  |
|  | | | **Files\\IDI- - Health Educator- UCI-09** | | | | | | | | | | | | | |  |
|  |  |  | No |  |  |  | 0.0119 |  | 3 | |  | | | | | |  |
|  | | |  |  |  |  |  |  |  | |  | | | | | | |
|  | | | | | | | | | | | | 1 |  | AT |  | 7/24/2020 10:26 AM |  |
|  | sometimes these patients are so much in pain that even when the doctor tries to explain the cancer conditions and so forth, at that situation the patient cannot understand. | | | | | | | | | | | | | | | |  |
|  |  | | | | | | | | | | | | | | | |  |
|  | | | | | | | | | | | | 2 |  | AT |  | 7/24/2020 10:27 AM |  |
|  | The pain may not be like the physical pain due to cancer, a cancer diagnosis is a shock like a death sentence to many people so at that time the patient is pre-occupied with am I going to die as always said it is not true so most patients live at that point without understanding their cancer condition. | | | | | | | | | | | | | | | |  |
|  |  | | | | | | | | | | | | | | | |  |
|  | | | | | | | | | | | | 3 |  | AT |  | 7/25/2020 9:38 PM |  |
|  | As I said disclosure is complex may be when the doctor has studied that may be some one is not ready for disclosure looking at the condition of the patient and the situation on the condition of the patient, I can’t disclosure to the patient depending on this and that. Doctors will say lets disclose this, lets support this patient until he is strong enough then we say it out. | | | | | | | | | | | | | | | |  |
|  |  | | | | | | | | | | | | | | | |  |
|  | | | | | | | | | | | | | | | | | |
| Reports\\Coding Summary By Code Report | | | | | | | | | | Page 42 of 117 | | | | | | | |
| 8/15/2023 8:10 AM | | | | | | | | | | | | | | | | | |
|  | | | **Aggregate** |  | **Classification** |  | **Coverage** |  | **Number Of Coding References** | |  | **Reference Number** |  | **Coded By Initials** |  | **Modified On** |  |
|  | | | **Files\\IDI_ Counselor_UCI_01** | | | | | | | | | | | | | |  |
|  |  |  | No |  |  |  | 0.093 |  | 1 | |  | | | | | |  |
|  | | |  |  |  |  |  |  |  | |  | | | | | | |
|  | | | | | | | | | | | | 1 |  | AT |  | 7/25/2020 10:52 PM |  |
|  | Yes, we have received them coming for other issues then we find out the patient has not been knowing the diagnosis they say for me I have been here for two years now I don’t know, I didn’t know I have been suffering from cancer, yes it has happened. | | | | | | | | | | | | | | | |  |
|  |  | | | | | | | | | | | | | | | |  |
|  | | | **Files\\IDI_ Social worker_UCI_03** | | | | | | | | | | | | | |  |
|  |  |  | No |  |  |  | 0.0168 |  | 2 | |  | | | | | |  |
|  | | |  |  |  |  |  |  |  | |  | | | | | | |
|  | | | | | | | | | | | | 1 |  | AT |  | 7/26/2020 8:41 AM |  |
|  | Perhaps that’s my thinking and that is usually related to the patient’s stay if the patient is knowledgeable if they get information if the patient is not knowledgeable meaning uneducated. They usually have very limited information about disease, stages and everything in fact they don’t have that because I have noticed that difference. | | | | | | | | | | | | | | | |  |
|  |  | | | | | | | | | | | | | | | |  |
|  | | | | | | | | | | | | 2 |  | AT |  | 7/26/2020 8:41 AM |  |
|  | For we noticed because of their explanation of how they try to express the information they have picked from the doctor because if a patient is a bit more educated at least they also ask some questions by themselves they don’t just listen to what the doctor says and get out but when a patient is not educated the patient just walks out after hearing one or two from the doctor. | | | | | | | | | | | | | | | |  |
|  |  | | | | | | | | | | | | | | | |  |
|  | | | **Files\\IDI__ Doctor_ UCI_ 05** | | | | | | | | | | | | | |  |
|  |  |  | No |  |  |  | 0.0194 |  | 2 | |  | | | | | |  |
|  | | |  |  |  |  |  |  |  | |  | | | | | | |
|  | | | | | | | | | | | | 1 |  | AT |  | 7/26/2020 10:40 AM |  |
|  | In my own thinking, the only disadvantage which I think about is understanding the patient having a lot of information and sometimes when you know too much then it may be a challenge so that first challenge patients get a lot of information so retention may be a very big challenge so I am trying to relate that to information that they may not remember everything. | | | | | | | | | | | | | | | |  |
|  |  | | | | | | | | | | | | | | | |  |
|  | | | | | | | | | | | | 2 |  | AT |  | 7/26/2020 10:45 AM |  |
|  | Education I think plays a big role but its not so much especially if I am speaking in the native language like Luganda, whether you have gone to school or you’ve not gone to school, I will figure a way in which to pass on this information to the one who has gone to school and to even that one who may not have to school. I usually know how to go around to put myself in the shoes of the patient. | | | | | | | | | | | | | | | |  |
|  |  | | | | | | | | | | | | | | | |  |
|  | | | | | | | | | | | | | | | | | |
|  | | | | | | | | | | | | | | | | | |
|  | | | | | | | | | | | | | | | | | |
| Reports\\Coding Summary By Code Report | | | | | | | | | | Page 43 of 117 | | | | | | | |
| 8/15/2023 8:10 AM | | | | | | | | | | | | | | | | | |
|  | | | **Aggregate** |  | **Classification** |  | **Coverage** |  | **Number Of Coding References** | |  | **Reference Number** |  | **Coded By Initials** |  | **Modified On** |  |
|  | **Nodes\\Barriers to consenting or communicating information about Cancer prognosis to patients and its related effects\Patients differing to know info about Cancer** | | | | | | | | | | | | | | | |  |
|  | | **Document** | | | | | | | | | | | | | | |  |
|  | | | **Files\\IDI - _ Doctor_UCI_06** | | | | | | | | | | | | | |  |
|  |  |  | No |  |  |  | 0.0166 |  | 1 | |  | | | | | |  |
|  | | |  |  |  |  |  |  |  | |  | | | | | | |
|  | | | | | | | | | | | | 1 |  | AT |  | 7/24/2020 8:48 AM |  |
|  | Sometimes you may differ giving them that information some thing that will happen to me is a good number times when patients come to the clinic we also ask them what do you want to know about the disease that question is commonly asked so there are patients who come out clearly and say that I don’t want to know how far it has gone o whatever you tell me I don’t want treatment or whatever you do, they will tell you what they want. But I haven’t faced a situation where a patient doesn’t want to know, most of them actually want to know what is happening. | | | | | | | | | | | | | | | |  |
|  |  | | | | | | | | | | | | | | | |  |
|  | **Nodes\\Barriers to consenting or communicating information about Cancer prognosis to patients and its related effects\Patients don’t want to sign for procedures that are risky** | | | | | | | | | | | | | | | |  |
|  | | **Document** | | | | | | | | | | | | | | |  |
|  | | | **Files\\IDI -Specialist palliative care_10** | | | | | | | | | | | | | |  |
|  |  |  | No |  |  |  | 0.090 |  | 1 | |  | | | | | |  |
|  | | |  |  |  |  |  |  |  | |  | | | | | | |
|  | | | | | | | | | | | | 1 |  | AT |  | 7/24/2020 10:15 AM |  |
|  | Every time you ask them to tell them about a procedure or a treatment and say now these are the disadvantages and then you want to get their informed consent they will fear, they don’t want to sign , every time you tell a patient to sign, they will be suspicious and say why sign, but Musawo am not willing, let me first ask my husband, | | | | | | | | | | | | | | | |  |
|  |  | | | | | | | | | | | | | | | |  |
|  | | | **Files\\IDI__ Doctor_ UCI_ 05** | | | | | | | | | | | | | |  |
|  |  |  | No |  |  |  | 0.0590 |  | 4 | |  | | | | | |  |
|  | | |  |  |  |  |  |  |  | |  | | | | | | |
|  | | | | | | | | | | | | 1 |  | AT |  | 7/26/2020 10:52 AM |  |
|  | Yeah, absolutely, two situations which off head one if a patient declines treatment we humbly request them to sign that they don’t want to receive some cancer specific treatment because we have seen patients who say no and then they come when the disease has spread everywhere and then the attendants say the patient came here and you refused to give them treatment so if a patient declines whether its chemotherapy, whether its surgery, whether radiotherapy, whether its tablets we kindly request them to document. | | | | | | | | | | | | | | | |  |
|  |  | | | | | | | | | | | | | | | |  |
|  | | | | | | | | | | | | 2 |  | AT |  | 7/26/2020 10:52 AM |  |
|  | Then there are also those patient who are like oh allow me to first go back home and then come back on a later date we also request them to sign because we have seen that some patients say that when they want to go and try other forms whether its witchcraft, herbs then they come back again when the disease is advanced then they tend to blame but when they documented it, it makes more sense. | | | | | | | | | | | | | | | |  |
|  |  | | | | | | | | | | | | | | | |  |
|  | | | | | | | | | | | | 3 |  | AT |  | 7/26/2020 10:53 AM |  |
|  | I think people respond differently, I think like I said when you’re treating cancer you follow a specific protocol which is different from the different disease so it means that if I don’t follow a specific protocol, there may be challenges and this translates in to challenges also with your outcomes at the same time so sometimes as health workers you may not take it well if we say that oh the cancer you have we can get cure from surgery per say and then the patient is like no I don’t want surgery so that means I’m going to change the protocol because you are meant to be treated by surgery alone and you’re cured now if its not that, it means we are going to use either tablets, whether its radiotherapy or chemotherapy which may not concur the same outcomes or we may need to combine, we need to do surgery, we need to do chemotherapy together and find for you say no I want chemotherapy alone, I don’t want surgery so that brings challenges. | | | | | | | | | | | | | | | |  |
|  |  |  |  |  |  |  |  |  |  |  |  |  |  |  |  |  |  |
|  |  | | | | | | | | | | | | | | | |  |
| Reports\\Coding Summary By Code Report | | | | | | | | | | Page 44 of 117 | | | | | | | |
| 8/15/2023 8:10 AM | | | | | | | | | | | | | | | | | |
|  | | | **Aggregate** |  | **Classification** |  | **Coverage** |  | **Number Of Coding References** | |  | **Reference Number** |  | **Coded By Initials** |  | **Modified On** |  |
|  | | | | | | | | | | | | | | | | | |
|  | | | | | | | | | | | | 4 |  | AT |  | 7/26/2020 10:54 AM |  |
|  | It’s a very simple way to do that, its very simple, give the information to the patient which is very important and let them make an informed decision but also try to revisit the same situation when they come back, maybe they will have changed their mind but also bring in another party as well to talk to the patient that’s where a counsellor plays a role but also my colleague can see this patient on the next visit and they still try to discuss the same thing. | | | | | | | | | | | | | | | |  |
|  |  | | | | | | | | | | | | | | | |  |
|  | **Nodes\\Barriers to consenting or communicating information about Cancer prognosis to patients and its related effects\Patient's unreadiness to be disclosed to** | | | | | | | | | | | | | | | |  |
|  | | **Document** | | | | | | | | | | | | | | |  |
|  | | | **Files\\IDI- - Health Educator- UCI-09** | | | | | | | | | | | | | |  |
|  |  |  | No |  |  |  | 0.049 |  | 1 | |  | | | | | |  |
|  | | |  |  |  |  |  |  |  | |  | | | | | | |
|  | | | | | | | | | | | | 1 |  | AT |  | 7/25/2020 9:43 PM |  |
|  | Sometimes some patients are not ready, immediate disclosure is blowing them up, you see them bursting in to tears and walking away. I would say they are shocked yes immediate disclosure shocks the patients totally if the patient is not prepared for it so if a patient is now ready for disclosure is now ready for it as can be done by doctors and nurses. | | | | | | | | | | | | | | | |  |
|  |  | | | | | | | | | | | | | | | |  |
|  | | | **Files\\IDI__ Doctor_ UCI_ 05** | | | | | | | | | | | | | |  |
|  |  |  | No |  |  |  | 0.081 |  | 1 | |  | | | | | |  |
|  | | |  |  |  |  |  |  |  | |  | | | | | | |
|  | | | | | | | | | | | | 1 |  | AT |  | 7/26/2020 10:59 AM |  |
|  | Its time, time is a challenge because you’re meant to give a patient time to make an informed decision, they don’t even have to sign on that day they can even go and come back on another day but here it is the reverse because you open the file on that day so they make an informed decision so I think it’s mainly time. | | | | | | | | | | | | | | | |  |
|  |  | | | | | | | | | | | | | | | |  |
|  | **Nodes\\Barriers to consenting or communicating information about Cancer prognosis to patients and its related effects\poor documentation** | | | | | | | | | | | | | | | |  |
|  | | **Document** | | | | | | | | | | | | | | |  |
|  | | | **Files\\IDI - - Doctor- UCI -08** | | | | | | | | | | | | | |  |
|  |  |  | No |  |  |  | 0.0064 |  | 1 | |  | | | | | |  |
|  | | |  |  |  |  |  |  |  | |  | | | | | | |
|  | | | | | | | | | | | | 1 |  | AT |  | 7/24/2020 8:20 AM |  |
|  | I think there is no much proper documented informed consent again they give, it is only that one the rest the just give them information, the only part I wouldn’t say informed consent. | | | | | | | | | | | | | | | |  |
|  |  | | | | | | | | | | | | | | | |  |
| Reports\\Coding Summary By Code Report | | | | | | | | | | Page 45 of 117 | | | | | | | |
| 8/15/2023 8:10 AM | | | | | | | | | | | | | | | | | |
|  | | | **Aggregate** |  | **Classification** |  | **Coverage** |  | **Number Of Coding References** | |  | **Reference Number** |  | **Coded By Initials** |  | **Modified On** |  |
|  | | | **Files\\IDI_ _ Nurse_UCI_04** | | | | | | | | | | | | | |  |
|  |  |  | No |  |  |  | 0.0389 |  | 3 | |  | | | | | |  |
|  | | |  |  |  |  |  |  |  | |  | | | | | | |
|  | | | | | | | | | | | | 1 |  | AT |  | 7/26/2020 10:14 AM |  |
|  | I most case we have actually got most patient who have ended up writing their name on the file because the names are not fully appearing in the files and later on either when they pass on is when the relatives tend to come and check out and they are like she was called such and such, he or she missed out these names, he did not use all the names so in that process of consenting people I think there is not so much explanation being given to them. | | | | | | | | | | | | | | | |  |
|  |  | | | | | | | | | | | | | | | |  |
|  | | | | | | | | | | | | 2 |  | AT |  | 7/26/2020 10:14 AM |  |
|  | Now that is something that is actually a missing the link which is happening there, I don’t know but ideally I think this personnel in the process of opening the patient’s file would help to guide the patients what kind of documents they are opening, what exactly needs to be in that document and explain to them that whatever they put in there may not be reversed. | | | | | | | | | | | | | | | |  |
|  |  | | | | | | | | | | | | | | | |  |
|  | | | | | | | | | | | | 3 |  | AT |  | 7/26/2020 10:14 AM |  |
|  | It would be informed consent of care, its actually the doctor that first explains and also the nurses tend to explain. The doctor explains to you the condition you have the treatment and then asks you are okay with it? You’re either going to say you’re okay or not. If you’re okay with the treatment, if you’re not going to take chemotherapy like for example then its written and documented in the file there that you’re not able to take chemotherapy. | | | | | | | | | | | | | | | |  |
|  |  | | | | | | | | | | | | | | | |  |
|  | **Nodes\\Barriers to consenting or communicating information about Cancer prognosis to patients and its related effects\poor staff attitude** | | | | | | | | | | | | | | | |  |
|  | | **Document** | | | | | | | | | | | | | | |  |
|  | | | **Files\\IDI -Specialist palliative care_10** | | | | | | | | | | | | | |  |
|  |  |  | No |  |  |  | 0.0058 |  | 1 | |  | | | | | |  |
|  | | |  |  |  |  |  |  |  | |  | | | | | | |
|  | | | | | | | | | | | | 1 |  | AT |  | 7/24/2020 9:58 AM |  |
|  | Especially if they communicate a preference which is against the health providers decision for example: when they say I don’t want radiotherapy then the person is offended and says you go home so sometimes it happens. | | | | | | | | | | | | | | | |  |
|  |  | | | | | | | | | | | | | | | |  |
|  | | | **Files\\IDI- - Health Educator- UCI-09** | | | | | | | | | | | | | |  |
|  |  |  | No |  |  |  | 0.0108 |  | 2 | |  | | | | | |  |
|  | | |  |  |  |  |  |  |  | |  | | | | | | |
|  | | | | | | | | | | | | 1 |  | AT |  | 7/25/2020 10:05 PM |  |
|  | They are not given that time because there are patients who don’t even know that they are supposed to know. I know I don’t know who brought the white coat sometimes the clinical coat is highly feared by the patients, whoever is putting on the clinical coat you do not have any strength to ask. | | | | | | | | | | | | | | | |  |
|  |  | | | | | | | | | | | | | | | |  |
|  | | | | | | | | | | | | 2 |  | AT |  | 7/25/2020 10:26 PM |  |
|  | the attitude of our nurses towards patients influence how they respond to the questions asked to patients, you will find this nurse with patients crowed on the desk and generally say walk away, do not stand here go and stand there but does not say go and stand there I will call you one by one and I listen to you so you go and stand there and no one calls you back. Its literally chasing us away so those are the kind of things we are talking about the attitude of the nurse. | | | | | | | | | | | | | | | |  |
|  |  | | | | | | | | | | | | | | | |  |
| Reports\\Coding Summary By Code Report | | | | | | | | | | Page 46 of 117 | | | | | | | |
| 8/15/2023 8:10 AM | | | | | | | | | | | | | | | | | |
|  | | | **Aggregate** |  | **Classification** |  | **Coverage** |  | **Number Of Coding References** | |  | **Reference Number** |  | **Coded By Initials** |  | **Modified On** |  |
|  | | | **Files\\IDI_ Social worker_UCI_03** | | | | | | | | | | | | | |  |
|  |  |  | No |  |  |  | 0.0655 |  | 4 | |  | | | | | |  |
|  | | |  |  |  |  |  |  |  | |  | | | | | | |
|  | | | | | | | | | | | | 1 |  | AT |  | 7/26/2020 8:58 AM |  |
|  | I wish I can ask one of the care takers to explain this at this time but I am going to explain it on behalf of that care taker, the care taker has identified two staff in the OPD that are very difficult to talk to and they are even scared to go to them, once they see that person they dodge so now you see the time wasted doing all this kind of thing they cannot go back to ask questions because if they see this particular person they wait and wait up to that time, supposing his person is off. | | | | | | | | | | | | | | | |  |
|  |  | | | | | | | | | | | | | | | |  |
|  | | | | | | | | | | | | 2 |  | AT |  | 7/26/2020 9:01 AM |  |
|  | I think to me this is a part which is very heart breaking, I don’t think patients are listened to and this has created fear in patients to ask questions because I remember myself bring a patient who has been referred three times and the four time is when I bring that patient back to the doctor to try and explain to the patient or to help the patient understand what other ways can the patient be supported because to me the patient would have asked the doctor to discuss but now that might be ever going not actually being lifted up because they always refer her back , that you go back immunity is low then the patient came back to me and asked so I asked her did you ask the doctor what can now be done because this is now the forth time you’re going back there could be some other ways to help you get out of this. | | | | | | | | | | | | | | | |  |
|  |  | | | | | | | | | | | | | | | |  |
|  | | | | | | | | | | | | 3 |  | AT |  | 7/26/2020 9:01 AM |  |
|  | I personally know sometimes it can be boasted so when I came back with the patient we together went to inquire from the doctor and he became very wild to the extent that the in charge had to hold my hand and the doctor’s hand and pulled us aside to try and understand because that was the patient’s role to maybe find out from the doctor what can be done to help the patient because the patient now is trying to what he or she now do in order to continue with her treatment if it is the nutrifeed that is a problem and that is purely the doctor’s role to try and help the patient understand, give the patient the information but the doctor could not give the patient the information he was very wild and the patient was also fearing so I had to come in and come together with the patient. | | | | | | | | | | | | | | | |  |
|  |  | | | | | | | | | | | | | | | |  |
|  | | | | | | | | | | | | 4 |  | AT |  | 7/26/2020 9:03 AM |  |
|  | The solution was now to go and see the senior doctor, book for a senior doctor to reveal why this is and hat can be done which would have been the doctor’s potion like now this would have been the junior doctor to advice the patient if that is the case let us book for senior doctor and again we here from a senior doctor and unfortunately this patient passed on and am very sad about this. Patients are a not supported to understand how they can cope with their life during this treatment process and yet this is pure their mutual relationship with the doctor to understand, for patients to be free to express themselves of what they are going through and how they can be supported. | | | | | | | | | | | | | | | |  |
|  |  | | | | | | | | | | | | | | | |  |
|  | | | **Files\\IDI__ Doctor_ UCI_ 05** | | | | | | | | | | | | | |  |
|  |  |  | No |  |  |  | 0.0319 |  | 3 | |  | | | | | |  |
|  | | |  |  |  |  |  |  |  | |  | | | | | | |
|  | | | | | | | | | | | | 1 |  | AT |  | 7/26/2020 10:48 AM |  |
|  | I think there are mainly two reasons, number one, I think in this country patients still see doctors as very special creatures or big people who you don’t question but secondly I think people who come from the rural areas I think are sometimes intimidated as well either by the doctor who sees them or by the stage. Maybe they come to Kampala for the first time they come to cancer institute for the first time and maybe they are intimidated, that’s what I think they get overwhelmed by the stage it happens. | | | | | | | | | | | | | | | |  |
|  |  | | | | | | | | | | | | | | | |  |
|  | | | | | | | | | | | | 2 |  | AT |  | 7/26/2020 10:48 AM |  |
|  | Absolutely, I think I have heard about patients who are like oh no I don’t want to be seen by that specific person he doesn’t explain well for me to understand and may be some patients speak that about me I don’t know but I have also patients who come and are like oh me I want to be seen by that person because he is going to give me time, he is going to explain for me and also understand and you know we are all different. | | | | | | | | | | | | | | | |  |
|  |  | | | | | | | | | | | | | | | |  |
|  | | | | | | | | | | | | 3 |  | AT |  | 7/26/2020 10:48 AM |  |
|  | Even the mood, even the way you give information, me I think the way you give information you as the doctor you have all the information, there is the black board information, we have the book information but the way you translate to a lay person to make me understand in a simple way me I think is what is important. | | | | | | | | | | | | | | | |  |
|  |  | | | | | | | | | | | | | | | |  |
|  | | | | | | | | | | | | | | | | | |
| Reports\\Coding Summary By Code Report | | | | | | | | | | Page 47 of 117 | | | | | | | |
| 8/15/2023 8:10 AM | | | | | | | | | | | | | | | | | |
|  | | | **Aggregate** |  | **Classification** |  | **Coverage** |  | **Number Of Coding References** | |  | **Reference Number** |  | **Coded By Initials** |  | **Modified On** |  |
|  | **Nodes\\Barriers to consenting or communicating information about Cancer prognosis to patients and its related effects\Providers lack of knowledge** | | | | | | | | | | | | | | | |  |
|  | | **Document** | | | | | | | | | | | | | | |  |
|  | | | **Files\\IDI- - Health Educator- UCI-09** | | | | | | | | | | | | | |  |
|  |  |  | No |  |  |  | 0.0133 |  | 3 | |  | | | | | |  |
|  | | |  |  |  |  |  |  |  | |  | | | | | | |
|  | | | | | | | | | | | | 1 |  | AT |  | 7/25/2020 10:27 PM |  |
|  | The third one we need to talk about is sometimes we work at cancer institute but we do not know the information to tell patients so there are times that patients have asked the health workers when the health workers have no response to such questions, they actually do not know. | | | | | | | | | | | | | | | |  |
|  |  | | | | | | | | | | | | | | | |  |
|  | | | | | | | | | | | | 2 |  | AT |  | 7/25/2020 10:28 PM |  |
|  | Knowledge about the cancer itself , some of the nurses you are seeing here have never been trained on cancer, they know how to give chemo but they do not understand the cancer processes so they can’t respond to a patient who has asked so what does that mean to a nurse? So the technical cancer knowledge is also lacking. | | | | | | | | | | | | | | | |  |
|  |  | | | | | | | | | | | | | | | |  |
|  | | | | | | | | | | | | 3 |  | AT |  | 7/25/2020 10:39 PM |  |
|  | Now the actual challenge first of all is I see it as a challenge, the providers perception of informed consent. Perception I mean referred to what do providers know about informed consent, do they understand informed consent ideally how it is supposed to be and its done? Its implications on either party, so I see like that’s the first challenge. | | | | | | | | | | | | | | | |  |
|  |  | | | | | | | | | | | | | | | |  |
|  | | | **Files\\IDI_ Counselor_UCI_01** | | | | | | | | | | | | | |  |
|  |  |  | No |  |  |  | 0.0075 |  | 1 | |  | | | | | |  |
|  | | |  |  |  |  |  |  |  | |  | | | | | | |
|  | | | | | | | | | | | | 1 |  | AT |  | 7/25/2020 10:59 PM |  |
|  | There is no such guideline actually it is a common practice that they refer the disclosure, it is a rare case rarely they do it rarely just that some patients they leave the room when they already know | | | | | | | | | | | | | | | |  |
|  |  | | | | | | | | | | | | | | | |  |
|  | **Nodes\\Barriers to consenting or communicating information about Cancer prognosis to patients and its related effects\Resistance from family members** | | | | | | | | | | | | | | | |  |
|  | | **Document** | | | | | | | | | | | | | | |  |
|  | | | **Files\\IDI - - Doctor- UCI -08** | | | | | | | | | | | | | |  |
|  |  |  | No |  |  |  | 0.0117 |  | 2 | |  | | | | | |  |
|  | | |  |  |  |  |  |  |  | |  | | | | | | |
|  | | | | | | | | | | | | 1 |  | AT |  | 7/24/2020 8:06 AM |  |
|  | so but sometimes the family also ask you please don’t tell him or her now but when you go to the patient they say doctor what is the state of the disease I have so sometimes you say may be we are still doing a work up. | | | | | | | | | | | | | | | |  |
|  |  | | | | | | | | | | | | | | | |  |
|  | | | | | | | | | | | | 2 |  | AT |  | 7/24/2020 8:17 AM |  |
|  | Sometimes patients say don’t tell my relatives and sometimes relatives say don’t tell my patient so those hinderances. | | | | | | | | | | | | | | | |  |
|  |  | | | | | | | | | | | | | | | |  |
| Reports\\Coding Summary By Code Report | | | | | | | | | | Page 48 of 117 | | | | | | | |
| 8/15/2023 8:10 AM | | | | | | | | | | | | | | | | | |
|  | | | **Aggregate** |  | **Classification** |  | **Coverage** |  | **Number Of Coding References** | |  | **Reference Number** |  | **Coded By Initials** |  | **Modified On** |  |
|  | | | **Files\\IDI - _ Doctor_UCI_06** | | | | | | | | | | | | | |  |
|  |  |  | No |  |  |  | 0.0126 |  | 3 | |  | | | | | |  |
|  | | |  |  |  |  |  |  |  | |  | | | | | | |
|  | | | | | | | | | | | | 1 |  | AT |  | 7/24/2020 9:18 AM |  |
|  | Sometimes also the family is a big factor which may interfere with patients’ treatment they want to know like they want to override the patient. | | | | | | | | | | | | | | | |  |
|  |  | | | | | | | | | | | | | | | |  |
|  | | | | | | | | | | | | 2 |  | AT |  | 7/24/2020 9:19 AM |  |
|  | In that case we normally take the patients decision, we don’t take the family’s unless the patient is not of a sound mind but we normally take the patient’s decision. | | | | | | | | | | | | | | | |  |
|  |  | | | | | | | | | | | | | | | |  |
|  | | | | | | | | | | | | 3 |  | AT |  | 7/24/2020 9:19 AM |  |
|  | Those are really not common, they may arise but to us as health workers we normally take the patient’s decision. | | | | | | | | | | | | | | | |  |
|  |  | | | | | | | | | | | | | | | |  |
|  | | | **Files\\IDI -Specialist palliative care_10** | | | | | | | | | | | | | |  |
|  |  |  | No |  |  |  | 0.0294 |  | 2 | |  | | | | | |  |
|  | | |  |  |  |  |  |  |  | |  | | | | | | |
|  | | | | | | | | | | | | 1 |  | AT |  | 7/24/2020 9:38 AM |  |
|  | And then of course there are times when it will be difficult to disclose immediately even if the patient may not be ready because the family may be keeping you away from it, they may say don’t disclose this person is going to be depressed and die and therefore you have to work with the family first to bring them to understand why it is important to disclose then eventually disclose to the patient so that can be a delaying factor, sometimes patients are very sick and not really comprehending your information so you can’t hold a conversation. | | | | | | | | | | | | | | | |  |
|  |  | | | | | | | | | | | | | | | |  |
|  | | | | | | | | | | | | 2 |  | AT |  | 7/24/2020 9:40 AM |  |
|  | Not disclosing is mainly because relatives refuse to disclose, yeah that is where the big problem is and sometimes even there are some relationships on the ward where you find that a patient is under someone’s care and a senior person who doesn’t want you to disclose to their patients information of that nature because there are health professionals who believe that if you give that information you destroy patients’ hope so they an stop you and say you discourage our patient so there are a few people like that not many but there are those. | | | | | | | | | | | | | | | |  |
|  |  | | | | | | | | | | | | | | | |  |
|  | | | **Files\\IDI- - Health Educator- UCI-09** | | | | | | | | | | | | | |  |
|  |  |  | No |  |  |  | 0.0132 |  | 2 | |  | | | | | |  |
|  | | |  |  |  |  |  |  |  | |  | | | | | | |
|  | | | | | | | | | | | | 1 |  | AT |  | 7/24/2020 12:41 PM |  |
|  | Many times I have seen difficulties from relatives, patients are anxious to know and many times doctors test them, actually doctors are ones who are mandated to disclose to patients, initial visit that you have this cancer and this is the role of them but sometimes we interface with relatives who restrains you that do not tell that person that he or she has cancer and you now go back to digging deeper to who should actually receive the information about the disease of this person, is it the relatives or the patients. | | | | | | | | | | | | | | | |  |
|  |  | | | | | | | | | | | | | | | |  |
|  | | | | | | | | | | | | 2 |  | AT |  | 7/25/2020 9:34 PM |  |
|  | Sometimes the relatives will not want the doctor to tell the patient so the doctor must first of all make sure, they interface with the relatives. Two, sometimes the language may not be easy for the doctor to precisely disclose, the patient wants to know, the doctor wants to tell the patient but the language is a barrier which is something which needs to be handled. Now the other delay is the patient’s condition. | | | | | | | | | | | | | | | |  |
|  |  | | | | | | | | | | | | | | | |  |
|  | | | | | | | | | | | | | | | | | |
| Reports\\Coding Summary By Code Report | | | | | | | | | | Page 49 of 117 | | | | | | | |
| 8/15/2023 8:10 AM | | | | | | | | | | | | | | | | | |
|  | | | **Aggregate** |  | **Classification** |  | **Coverage** |  | **Number Of Coding References** | |  | **Reference Number** |  | **Coded By Initials** |  | **Modified On** |  |
|  | | | **Files\\IDI_ Counselor_UCI_01** | | | | | | | | | | | | | |  |
|  |  |  | No |  |  |  | 0.0061 |  | 1 | |  | | | | | |  |
|  | | |  |  |  |  |  |  |  | |  | | | | | | |
|  | | | | | | | | | | | | 1 |  | AT |  | 7/25/2020 10:50 PM |  |
|  | sometimes even the care takers they will tell the doctors we don’t want this person to know what they are suffering from then the doctors will send to counsellors. | | | | | | | | | | | | | | | |  |
|  |  | | | | | | | | | | | | | | | |  |
|  | | | **Files\\IDI_ Nurse_UCI_07** | | | | | | | | | | | | | |  |
|  |  |  | No |  |  |  | 0.0319 |  | 3 | |  | | | | | |  |
|  | | |  |  |  |  |  |  |  | |  | | | | | | |
|  | | | | | | | | | | | | 1 |  | AT |  | 7/26/2020 8:24 AM |  |
|  | Some of them tend to say they don’t know so we ask where are you, am in Uganda cancer institute so for us we treat cancer so you find that some patients prefer not to disclose to the attendants or care takers. | | | | | | | | | | | | | | | |  |
|  |  | | | | | | | | | | | | | | | |  |
|  | | | | | | | | | | | | 2 |  | AT |  | 7/26/2020 8:26 AM |  |
|  | Those instances are there but minimal , usually when these attendants don’t want the patient to know sometimes it is the attendants that know but they are afraid to tell the patient may be because the patient is very old or. There is usually delay but those delays are minimal because at the end of the day the patient will ask why am I being treated here but those ones are minimal. | | | | | | | | | | | | | | | |  |
|  |  | | | | | | | | | | | | | | | |  |
|  | | | | | | | | | | | | 3 |  | AT |  | 7/26/2020 8:26 AM |  |
|  | Usually we have to counsel those attendants because at the end of the day when a patient refuses to go to the hospital you will not force them, we usually counsel the attendants we take them to the counsellor, we tell them the importance of disclosing to the patient. We have to take them to the counsellor, the counsellor talks to the attendant first and when they really get the importance of disclosing, they still have to talk to the counsellor with the patient because disclosure is important for a patient to own the prognosis, the treatment and the disease its self. | | | | | | | | | | | | | | | |  |
|  |  | | | | | | | | | | | | | | | |  |
|  | | | **Files\\IDI_ Social worker_UCI_03** | | | | | | | | | | | | | |  |
|  |  |  | No |  |  |  | 0.0026 |  | 1 | |  | | | | | |  |
|  | | |  |  |  |  |  |  |  | |  | | | | | | |
|  | | | | | | | | | | | | 1 |  | AT |  | 7/26/2020 8:40 AM |  |
|  | And two they think that the family will not make a decision of taking a step to start their patient on the drug. | | | | | | | | | | | | | | | |  |
|  |  | | | | | | | | | | | | | | | |  |
|  | | | **Files\\IDI_ Nurse_UCI_02** | | | | | | | | | | | | | |  |
|  |  |  | No |  |  |  | 0.0296 |  | 2 | |  | | | | | |  |
|  | | |  |  |  |  |  |  |  | |  | | | | | | |
|  | | | | | | | | | | | | 1 |  | AT |  | 7/26/2020 9:26 AM |  |
|  | We are not going to be on the same track with the patient, the doctor and the relatives because you’ve not disclosed to them, they don’t know so they will take things for granted, 2. you see with disclosure it depends on the patient when the patient is disclosed to, he is the one to tell us to disclose to others, maybe the care takers. There is a scenario I got recently the patient did not want to disclose to the care takers the type of cancer she has so I was in that dilemma but also the care takers needed to know which cancer but the patient had refused me to disclose it’s a disadvantage. | | | | | | | | | | | | | | | |  |
|  |  | | | | | | | | | | | | | | | |  |
|  | | | | | | | | | | | | | | | | | |
| Reports\\Coding Summary By Code Report | | | | | | | | | | Page 50 of 117 | | | | | | | |
| 8/15/2023 8:10 AM | | | | | | | | | | | | | | | | | |
|  | | | **Aggregate** |  | **Classification** |  | **Coverage** |  | **Number Of Coding References** | |  | **Reference Number** |  | **Coded By Initials** |  | **Modified On** |  |
|  | | | | | | | | | | | | | | | | | |
|  | | | | | | | | | | | | 2 |  | AT |  | 7/26/2020 9:26 AM |  |
|  | I took them to the counsellors to solve issues from their because me am not supposed to disclosed what the patient has refused to disclose and it was very bad so if you don’t disclose both of them it gets to us, it was very bad to the point that even when she died still at her last breath people wanted to know what she is suffering from. So, disclosure helps sometimes but how. So, there are also those challenges of patients not wanting to disclose. | | | | | | | | | | | | | | | |  |
|  |  | | | | | | | | | | | | | | | |  |
|  | **Nodes\\Barriers to consenting or communicating information about Cancer prognosis to patients and its related effects\Resistance of refferal** | | | | | | | | | | | | | | | |  |
|  | | **Document** | | | | | | | | | | | | | | |  |
|  | | | **Files\\IDI- - Health Educator- UCI-09** | | | | | | | | | | | | | |  |
|  |  |  | No |  |  |  | 0.0065 |  | 1 | |  | | | | | |  |
|  | | |  |  |  |  |  |  |  | |  | | | | | | |
|  | | | | | | | | | | | | 1 |  | AT |  | 7/25/2020 9:31 PM |  |
|  | Last week I interfaced with one who was deceived, this one didn’t want to go home once referred now even when they are again try to bribe the nurse after understanding that they don’t want to take them home, what am trying to say is sometimes disclosure or kind of life care may mean differently to a patient, may mean you’re sending me home to go die, I want to die from the hospital so they will want to do everything to keep them around with in the hospital. | | | | | | | | | | | | | | | |  |
|  |  | | | | | | | | | | | | | | | |  |
|  | **Nodes\\Barriers to consenting or communicating information about Cancer prognosis to patients and its related effects\Stigma** | | | | | | | | | | | | | | | |  |
|  | | **Document** | | | | | | | | | | | | | | |  |
|  | | | **Files\\IDI - _ Doctor_UCI_06** | | | | | | | | | | | | | |  |
|  |  |  | No |  |  |  | 0.0361 |  | 2 | |  | | | | | |  |
|  | | |  |  |  |  |  |  |  | |  | | | | | | |
|  | | | | | | | | | | | | 1 |  | AT |  | 7/24/2020 8:58 AM |  |
|  | Oh yes that’s a big factor, fear is also cultural perception and community expectations of what’s happening and generally speaking the diagnosis of cancer carries a stigma in the community and most patients are apprehensive of everyone knowing the diagnosis even at the work place, they are private at what should be given so that sometimes works against the system because the patient will have say in the case of cervical cancer vaginal discharge for over a year but she will fear coming out or telling her friends or telling or going to a health clinic where they could have picked up and referred her enough. Its considered so private, | | | | | | | | | | | | | | | |  |
|  |  | | | | | | | | | | | | | | | |  |
|  | | | | | | | | | | | | 2 |  | AT |  | 7/24/2020 8:59 AM |  |
|  | its considered a taboo to tell someone that you have a flaw smelling discharge so by the time the patients come to the hospital its really late, the disease has really progressed so I would say that fears. There is this actual fear of doubt of the disease or communication or being told the truth but there are also these fears that are informal by society with culture about that or to be told if you have cancer its bad luck or there is something wrong with you. So instead of them coming to the hospital, they prefer to stay or go to alternative places or witch doctors. | | | | | | | | | | | | | | | |  |
|  |  | | | | | | | | | | | | | | | |  |
|  | | | | | | | | | | | | | | | | | |
| Reports\\Coding Summary By Code Report | | | | | | | | | | Page 51 of 117 | | | | | | | |
| 8/15/2023 8:10 AM | | | | | | | | | | | | | | | | | |
|  | | | **Aggregate** |  | **Classification** |  | **Coverage** |  | **Number Of Coding References** | |  | **Reference Number** |  | **Coded By Initials** |  | **Modified On** |  |
|  | | | **Files\\IDI- - Health Educator- UCI-09** | | | | | | | | | | | | | |  |
|  |  |  | No |  |  |  | 0.0185 |  | 2 | |  | | | | | |  |
|  | | |  |  |  |  |  |  |  | |  | | | | | | |
|  | | | | | | | | | | | | 1 |  | AT |  | 7/25/2020 10:31 PM |  |
|  | One: because cancer is a dangerous disease whenever you mention you have cancer people sympathize with you, you now run on people’s sympathy to obtain a little help either on your own behalf or on behalf of other patients so that is a social impact we are talking about. Two: | | | | | | | | | | | | | | | |  |
|  |  | | | | | | | | | | | | | | | |  |
|  | | | | | | | | | | | | 2 |  | AT |  | 7/25/2020 10:31 PM |  |
|  | it has also fetched them a little bit of stigmatization and rejection to some points because disclosure that you have cancer to some communities it is a death sentence so you are deprived of your properties because you will die soon or you also begin to have a feeling that because I am going to die let me exhaust what I had kept because it looks like tomorrow is darkened so it has the two arms, the survivors have taken cancer for survival, they have taken it as an opportunity o go and beg or to obtain some resources not for a good reason, some are doing for a good reason others are doing for a bad reason but also has fetched them the negative arm from the community or society where they live, they have been rejected, they are deprived of their rights and participation in communities because cancer will kill them, they have been deprived like you know the divorce, I married the woman because of the very good breast , now that the breast is not there you are not my wife, my wife had a breast so you will look in to all these aspects. | | | | | | | | | | | | | | | |  |
|  |  |  |  |  |  |  |  |  |  |  |  |  |  |  |  |  |  |
|  |  | | | | | | | | | | | | | | | |  |
|  | | | **Files\\IDI_ _ Nurse_UCI_04** | | | | | | | | | | | | | |  |
|  |  |  | No |  |  |  | 0.0107 |  | 1 | |  | | | | | |  |
|  | | |  |  |  |  |  |  |  | |  | | | | | | |
|  | | | | | | | | | | | | 1 |  | AT |  | 7/26/2020 10:23 AM |  |
|  | it can really affect your social life you don’t want to be so much with your friends, you’re just isolated or like you’re on treatment, you have already lost your hair and you have a stigma of coming in to the community that you don’t have hair at all so you’re kind of like hiding, you want to be way from family and friends so there is that stigma. | | | | | | | | | | | | | | | |  |
|  |  | | | | | | | | | | | | | | | |  |
|  | | | **Files\\IDI__ Doctor_ UCI_ 05** | | | | | | | | | | | | | |  |
|  |  |  | No |  |  |  | 0.0393 |  | 3 | |  | | | | | |  |
|  | | |  |  |  |  |  |  |  | |  | | | | | | |
|  | | | | | | | | | | | | 1 |  | AT |  | 7/26/2020 10:55 AM |  |
|  | I have and the thing which comes to my mind immediately is stigma, actually I know of a particular patient who is in the social lime light who doesn’t any one to know that they have cancer so I think stigma may impact on the social life or the social well being of a particular person because if he hears that oh I have cancer may be people may look at me in a different way than what they were looking at me before. Economically, if somebody may be has cancer. | | | | | | | | | | | | | | | |  |
|  |  | | | | | | | | | | | | | | | |  |
|  | | | | | | | | | | | | 2 |  | AT |  | 7/26/2020 10:55 AM |  |
|  | Stigma is both, it may be your self but you may also think people look at you that way and yet you know that its not that. | | | | | | | | | | | | | | | |  |
|  |  | | | | | | | | | | | | | | | |  |
|  | | | | | | | | | | | | 3 |  | AT |  | 7/26/2020 10:56 AM |  |
|  | INTERVIEWER: Then the social withdrawing?  RESPONDENT: There are a few still and then few who have seen are mainly the adolescents, the teenagers a few and I have actually seen some who get over that depression as well and its mainly related to one, because some loose their hair so they somehow get isolated they no longer engage with their friends , they angry as well because the made to be in a specific place like you’re meant to be at school now you’re ending up being in hospital especially adolescents and teenagers, however I have also seen the elderly as well are like already i’m old why are bringing to hospital they are like I have already lived my life but you know they attendants are the one who want to push these people so actually depression really happens to cancer patients, it really does. And some people think that they are helping, the attendants sometimes think that they are helping but when they are making the situation worse. | | | | | | | | | | | | | | | |  |
|  |  |  |  |  |  |  |  |  |  |  |  |  |  |  |  |  |  |
|  |  | | | | | | | | | | | | | | | |  |
|  | | | | | | | | | | | | | | | | | |
| Reports\\Coding Summary By Code Report | | | | | | | | | | Page 52 of 117 | | | | | | | |
| 8/15/2023 8:10 AM | | | | | | | | | | | | | | | | | |
|  | | | **Aggregate** |  | **Classification** |  | **Coverage** |  | **Number Of Coding References** | |  | **Reference Number** |  | **Coded By Initials** |  | **Modified On** |  |
|  | **Nodes\\Barriers to consenting or communicating information about Cancer prognosis to patients and its related effects\Uncertainity how patient will react after disclosure** | | | | | | | | | | | | | | | |  |
|  | | **Document** | | | | | | | | | | | | | | |  |
|  | | | **Files\\IDI -Specialist palliative care_10** | | | | | | | | | | | | | |  |
|  |  |  | No |  |  |  | 0.0323 |  | 3 | |  | | | | | |  |
|  | | |  |  |  |  |  |  |  | |  | | | | | | |
|  | | | | | | | | | | | | 1 |  | AT |  | 7/24/2020 9:41 AM |  |
|  | you don’t know how they will be responding and then you have left them to go home. So sometimes that doesn’t happen and you want to refer them to a home for the best palliative care services which can help to do that. | | | | | | | | | | | | | | | |  |
|  |  | | | | | | | | | | | | | | | |  |
|  | | | | | | | | | | | | 2 |  | AT |  | 7/24/2020 9:44 AM |  |
|  | May be the advantage is to the doctor to prevent discomfort but I think because cancer is a progressive disease even if you lie someone will come to reality that they are very ill and are not going to get well and I think many times they confess and say that I knew all along that me I knew that I was sick. | | | | | | | | | | | | | | | |  |
|  |  | | | | | | | | | | | | | | | |  |
|  | | | | | | | | | | | | 3 |  | AT |  | 7/24/2020 9:44 AM |  |
|  | I think it is a disadvantage that if someone had their plans you’ve never told them and they had these high hopes/ false hopes you have done them a disservice they get weaker and cannot implement plans they had earlier on to do their things when they were stronger yet they don’t have that opportunity any more. They loose trust in you with time that you are not truthful. People have learnt to think that health professionals always have to lie to comfort us, they think it is even normal, they think it is a good doctor who lies. Sometimes some of them think so although they feel angry sometimes when you lie to them and they are like I wish someone had told me. | | | | | | | | | | | | | | | |  |
|  |  | | | | | | | | | | | | | | | |  |
|  | | | **Files\\IDI- - Health Educator- UCI-09** | | | | | | | | | | | | | |  |
|  |  |  | No |  |  |  | 0.0149 |  | 2 | |  | | | | | |  |
|  | | |  |  |  |  |  |  |  | |  | | | | | | |
|  | | | | | | | | | | | | 1 |  | AT |  | 7/24/2020 12:44 PM |  |
|  | So disclosure has been difficult because there is disclosure at the initial visit we do disclosure on the treatment, the purpose of the treatment because there is treatment with objective of cure, there is treatment with objective of reducing symptoms which goes with palliation but there is where treatment does not help that this person came for cure but now you are trapped between telling the patient that your condition may not be treatable at this point. | | | | | | | | | | | | | | | |  |
|  |  | | | | | | | | | | | | | | | |  |
|  | | | | | | | | | | | | 2 |  | AT |  | 7/25/2020 9:37 PM |  |
|  | The doctor now wants to prove to these people that we want cure yet the thing cannot be cured. I have learnt of many but if you what is the rationale of this treatment, it is not there. I think it also came from the pressure from the patients and the care givers that we came here and we want to receive some treatment so that the doctor is now compelled to write treatment, consciously knowing that even this treatment is not going to help but because one wants to make those people happy when you move to please people you don’t have to do the right thing though some doctors are trapped in there. | | | | | | | | | | | | | | | |  |
|  |  | | | | | | | | | | | | | | | |  |
|  | | | **Files\\IDI_ Counselor_UCI_01** | | | | | | | | | | | | | |  |
|  |  |  | No |  |  |  | 0.0067 |  | 1 | |  | | | | | |  |
|  | | |  |  |  |  |  |  |  | |  | | | | | | |
|  | | | | | | | | | | | | 1 |  | AT |  | 7/25/2020 10:59 PM |  |
|  | some dodge others come when they don’t know, what makes them after their assessment they dodge to tell the patient and push to somebody else sometimes they are scared of doing it. | | | | | | | | | | | | | | | |  |
|  |  | | | | | | | | | | | | | | | |  |
|  | | | | | | | | | | | | | | | | | |
| Reports\\Coding Summary By Code Report | | | | | | | | | | Page 53 of 117 | | | | | | | |
| 8/15/2023 8:10 AM | | | | | | | | | | | | | | | | | |
|  | | | **Aggregate** |  | **Classification** |  | **Coverage** |  | **Number Of Coding References** | |  | **Reference Number** |  | **Coded By Initials** |  | **Modified On** |  |
|  | | | **Files\\IDI_ Social worker_UCI_03** | | | | | | | | | | | | | |  |
|  |  |  | No |  |  |  | 0.0062 |  | 1 | |  | | | | | |  |
|  | | |  |  |  |  |  |  |  | |  | | | | | | |
|  | | | | | | | | | | | | 1 |  | AT |  | 7/26/2020 9:07 AM |  |
|  | They choose to die but also depends , people fear death that is the truth and that is also the reason that might influence the disclosure by the doctor that people think they will die quickly so they think let us not tell him they might think they are going to die. | | | | | | | | | | | | | | | |  |
|  |  | | | | | | | | | | | | | | | |  |
|  | **Nodes\\Barriers to consenting or communicating information about Cancer prognosis to patients and its related effects\Wrong information** | | | | | | | | | | | | | | | |  |
|  | | **Document** | | | | | | | | | | | | | | |  |
|  | | | **Files\\IDI- - Health Educator- UCI-09** | | | | | | | | | | | | | |  |
|  |  |  | No |  |  |  | 0.0024 |  | 1 | |  | | | | | |  |
|  | | |  |  |  |  |  |  |  | |  | | | | | | |
|  | | | | | | | | | | | | 1 |  | AT |  | 7/25/2020 9:48 PM |  |
|  | Actually, the delay I’m talking about must not delay beyond 24 hours, in the cancer environment everyone is providing information and up to 80% of the information is wrong. | | | | | | | | | | | | | | | |  |
|  |  | | | | | | | | | | | | | | | |  |
|  | **Nodes\\Enablers of immediate disclosure and informed consent process\Adequate preparation** | | | | | | | | | | | | | | | |  |
|  | | **Document** | | | | | | | | | | | | | | |  |
|  | | | **Files\\IDI_ Social worker_UCI_03** | | | | | | | | | | | | | |  |
|  |  |  | No |  |  |  | 0.0061 |  | 1 | |  | | | | | |  |
|  | | |  |  |  |  |  |  |  | |  | | | | | | |
|  | | | | | | | | | | | | 1 |  | AT |  | 7/26/2020 8:49 AM |  |
|  | Yes, to them yes, immediate disclosure that is why am saying the doctor who discloses it needs to first prepare them and judge for himself, how much more should give these people and maybe I give them another appointment or I refer them to someone to disclose. | | | | | | | | | | | | | | | |  |
|  |  | | | | | | | | | | | | | | | |  |
|  | | | | | | | | | | | | | | | | | |
|  | | | | | | | | | | | | | | | | | |
| Reports\\Coding Summary By Code Report | | | | | | | | | | Page 54 of 117 | | | | | | | |
| 8/15/2023 8:10 AM | | | | | | | | | | | | | | | | | |
|  | | | **Aggregate** |  | **Classification** |  | **Coverage** |  | **Number Of Coding References** | |  | **Reference Number** |  | **Coded By Initials** |  | **Modified On** |  |
|  | **Nodes\\Enablers of immediate disclosure and informed consent process\Adequate time** | | | | | | | | | | | | | | | |  |
|  | | **Document** | | | | | | | | | | | | | | |  |
|  | | | **Files\\IDI- - Health Educator- UCI-09** | | | | | | | | | | | | | |  |
|  |  |  | No |  |  |  | 0.092 |  | 3 | |  | | | | | |  |
|  | | |  |  |  |  |  |  |  | |  | | | | | | |
|  | | | | | | | | | | | | 1 |  | AT |  | 7/25/2020 9:51 PM |  |
|  | Giving information needs time, the time the doctor spends with the patient is so short that patients cannot understand so the doctor ends up communicating that you have this cancer but forgets to verify whether this person has understood their condition, very short time. | | | | | | | | | | | | | | | |  |
|  |  | | | | | | | | | | | | | | | |  |
|  | | | | | | | | | | | | 2 |  | AT |  | 7/25/2020 10:09 PM |  |
|  | Then communication needs time, the time we have to communicate with patients is very minimal. Then communication needs feedback, we do not have time to verify whether patients have understood us or not so while we say I only say unilaterally, this is breast cancer you will be seen next time finished, one-way communication which is a very big problem. | | | | | | | | | | | | | | | |  |
|  |  | | | | | | | | | | | | | | | |  |
|  | | | | | | | | | | | | 3 |  | AT |  | 7/25/2020 10:09 PM |  |
|  | Time and sometimes the skills. | | | | | | | | | | | | | | | |  |
|  |  | | | | | | | | | | | | | | | |  |
|  | **Nodes\\Enablers of immediate disclosure and informed consent process\Appropriate treatment supporter** | | | | | | | | | | | | | | | |  |
|  | | **Document** | | | | | | | | | | | | | | |  |
|  | | | **Files\\IDI_ _ Nurse_UCI_04** | | | | | | | | | | | | | |  |
|  |  |  | No |  |  |  | 0.0329 |  | 2 | |  | | | | | |  |
|  | | |  |  |  |  |  |  |  | |  | | | | | | |
|  | | | | | | | | | | | | 1 |  | AT |  | 7/26/2020 10:03 AM |  |
|  | The other thing that actually helps to guide us is patient appearing and when we see that this patient needs an attendant somebody to support him or her during the process of the treatment and during the course of this disease process. Now lack of that personnel is kind of going to interfere we want and demand and say can we the attendant here, can we have the next of kin here, we want someone whom we can give information because these patients sometimes present to us when they are weak and they are not able to run up certain things but we need somebody. | | | | | | | | | | | | | | | |  |
|  |  | | | | | | | | | | | | | | | |  |
|  | | | | | | | | | | | | 2 |  | AT |  | 7/26/2020 10:03 AM |  |
|  | Yes, availability of the attendant can influence immediate disclosure. The other thing is really its kind of dependant on the historical diagnosis and the story that the patient presents with is going to guide us because we are able to tell you how you have got like cancer of the breast, so the historical confirms it, some of the diagnosis helps to guide us in that disclosure like biopsy, tumor markers that can help guide and show to us that ok this is what we are dealing with and this is where we are going. | | | | | | | | | | | | | | | |  |
|  |  | | | | | | | | | | | | | | | |  |
|  | | | **Files\\IDI__ Doctor_ UCI_ 05** | | | | | | | | | | | | | |  |
|  |  |  | No |  |  |  | 0.0222 |  | 2 | |  | | | | | |  |
|  | | |  |  |  |  |  |  |  | |  | | | | | | |
|  | | | | | | | | | | | | 1 |  | AT |  | 7/26/2020 10:36 AM |  |
|  | But also, the attendants who come with the patient as well. I think from experience, there are very few patients who i have seen who may not be interested or who may tell me that I don’t want to know, I think they are very few really who have seen but I think all cancer patients really may want to know. I think you will want to know what is in for me, what are you able to do for me, what aren’t you able to do for me so I think all the patients have really interfaced with ask questions unless the patient doesn’t understand what I am talking about. | | | | | | | | | | | | | | | |  |
|  |  | | | | | | | | | | | | | | | |  |
| Reports\\Coding Summary By Code Report | | | | | | | | | | Page 55 of 117 | | | | | | | |
| 8/15/2023 8:10 AM | | | | | | | | | | | | | | | | | |
|  | | | **Aggregate** |  | **Classification** |  | **Coverage** |  | **Number Of Coding References** | |  | **Reference Number** |  | **Coded By Initials** |  | **Modified On** |  |
|  | | | | | | | | | | | | | | | | | |
|  | | | | | | | | | | | | 2 |  | AT |  | 7/26/2020 10:54 AM |  |
|  | But also, we do family conferences in cancer treatment especially when we realise that the patient is may be is having challenges making some decisions we prefer to bring a bigger family and try to talk about the same thing and may be try to find out why the patient is declining in receiving whatever we give the patient. | | | | | | | | | | | | | | | |  |
|  |  | | | | | | | | | | | | | | | |  |
|  | **Nodes\\Enablers of immediate disclosure and informed consent process\Assessing patient’s prior knowledge about Cancer** | | | | | | | | | | | | | | | |  |
|  | | **Document** | | | | | | | | | | | | | | |  |
|  | | | **Files\\IDI -Specialist palliative care_10** | | | | | | | | | | | | | |  |
|  |  |  | No |  |  |  | 0.0218 |  | 2 | |  | | | | | |  |
|  | | |  |  |  |  |  |  |  | |  | | | | | | |
|  | | | | | | | | | | | | 1 |  | AT |  | 7/24/2020 9:29 AM |  |
|  | The first we encounter we want to find out how much that person knows about the condition they so that will guide our conversation and the information we give subsequently, how much do they know about the condition and then what are their expectations from care, once we know that then we can know how to communicate further. | | | | | | | | | | | | | | | |  |
|  |  | | | | | | | | | | | | | | | |  |
|  | | | | | | | | | | | | 2 |  | AT |  | 7/24/2020 9:37 AM |  |
|  | Than someone who may be knowing about cancer the first time. And also the other things I talked about was the investigations, if you don’t have full information or if a patient is not well investigated you cant be confident to say that when you don’t even know the staging of the patient and also its easier when someone goes out straight away and say how long do I have to live to talk about the prognosis than someone who doesn’t and also as you give your information the first time | | | | | | | | | | | | | | | |  |
|  |  | | | | | | | | | | | | | | | |  |
|  | | | **Files\\IDI_ Social worker_UCI_03** | | | | | | | | | | | | | |  |
|  |  |  | No |  |  |  | 0.0066 |  | 1 | |  | | | | | |  |
|  | | |  |  |  |  |  |  |  | |  | | | | | | |
|  | | | | | | | | | | | | 1 |  | AT |  | 7/26/2020 8:51 AM |  |
|  | Yes, that will be a shock. Then they begin saying why didn’t the doctor tell me early that this is the stage of my disease for those who are knowledgeable but for the unknowledgeable it may still remain the same. Except maybe for family members and friends might have those regrets. | | | | | | | | | | | | | | | |  |
|  |  | | | | | | | | | | | | | | | |  |
|  | | | | | | | | | | | | | | | | | |
|  | | | | | | | | | | | | | | | | | |
|  | | | | | | | | | | | | | | | | | |
| Reports\\Coding Summary By Code Report | | | | | | | | | | Page 56 of 117 | | | | | | | |
| 8/15/2023 8:10 AM | | | | | | | | | | | | | | | | | |
|  | | | **Aggregate** |  | **Classification** |  | **Coverage** |  | **Number Of Coding References** | |  | **Reference Number** |  | **Coded By Initials** |  | **Modified On** |  |
|  | **Nodes\\Enablers of immediate disclosure and informed consent process\Assessing patient’s readiness to receive information** | | | | | | | | | | | | | | | |  |
|  | | **Document** | | | | | | | | | | | | | | |  |
|  | | | **Files\\IDI -Specialist palliative care_10** | | | | | | | | | | | | | |  |
|  |  |  | No |  |  |  | 0.0332 |  | 3 | |  | | | | | |  |
|  | | |  |  |  |  |  |  |  | |  | | | | | | |
|  | | | | | | | | | | | | 1 |  | AT |  | 7/24/2020 9:34 AM |  |
|  | I think people grade someone basing on the receptiveness of information, there are some patients who it is okay to disclose to and therefore they will disclose on the ward round quickly but then there are those who may find that it may be complicated and you may even find that they refer that disclosure to the palliative care team may be of the prognosis especially but they would disclose the diagnosis the oncology team and there are some patients who may not even want to know information about their diagnosis and prognosis so sometimes even those may not be told. | | | | | | | | | | | | | | | |  |
|  |  | | | | | | | | | | | | | | | |  |
|  | | | | | | | | | | | | 2 |  | AT |  | 7/24/2020 9:37 AM |  |
|  | immediate disclosure is where I talked about how ready is someone to receive the information at the first time you see them depending on where they came from if already they had heard some idea it is easier to give them more information. | | | | | | | | | | | | | | | |  |
|  |  | | | | | | | | | | | | | | | |  |
|  | | | | | | | | | | | | 3 |  | AT |  | 7/24/2020 9:47 AM |  |
|  | In the beginning they may not need to know but with time slowly I begin to come to terms with my condition and ready to know and therefore I am telling you that there is no one size fits all because I may not be ready to receive the information but with immediate disclosure is not appropriate for me but with time I will be ready but if someone is ready then it will be best to give them the information when they are ready. | | | | | | | | | | | | | | | |  |
|  |  | | | | | | | | | | | | | | | |  |
|  | | | **Files\\IDI- - Health Educator- UCI-09** | | | | | | | | | | | | | |  |
|  |  |  | No |  |  |  | 0.0320 |  | 8 | |  | | | | | |  |
|  | | |  |  |  |  |  |  |  | |  | | | | | | |
|  | | | | | | | | | | | | 1 |  | AT |  | 7/25/2020 9:44 PM |  |
|  | This information must be assessed by the doctor for the patient. | | | | | | | | | | | | | | | |  |
|  |  | | | | | | | | | | | | | | | |  |
|  | | | | | | | | | | | | 2 |  | AT |  | 7/25/2020 9:46 PM |  |
|  | Now right sometimes you will ask if a patient has ever known why he is here or whether he would wish to know why he is here and whether the patient would wish to know why he is in hospital and you can bring it from both perspectives, what if they say you have cancer how would you respond to it? There they will say I really want to know such that I can do this and that but they have found cancer I will die so do you go ahead to kill that one away? | | | | | | | | | | | | | | | |  |
|  |  | | | | | | | | | | | | | | | |  |
|  | | | | | | | | | | | | 3 |  | AT |  | 7/25/2020 9:47 PM |  |
|  | If you delay disclosure, one: if it is based on reason that someone is not ready from the perspective that you have seen this person, disclosing at this time will cause more harm, | | | | | | | | | | | | | | | |  |
|  |  | | | | | | | | | | | | | | | |  |
|  | | | | | | | | | | | | 4 |  | AT |  | 7/25/2020 9:48 PM |  |
|  | it gives time to prepare this patient to receive the diagnosis and coping will now be easy. Two: delaying disclosure to a sick person helps the patient to recover first and you disclose to this person when the person can ably understand their condition than telling the patient when the patient cannot understand their condition. | | | | | | | | | | | | | | | |  |
|  |  | | | | | | | | | | | | | | | |  |
|  | | | | | | | | | | | | 5 |  | AT |  | 7/25/2020 9:49 PM |  |
|  | It must not exceed 24 hours. If this person is only anxious and if you think giving the information right away will do more harm, we need to quickly handle this person and re assess so after 24 hours we will be able. | | | | | | | | | | | | | | | |  |
|  |  | | | | | | | | | | | | | | | |  |
|  | | | | | | | | | | | | 6 |  | AT |  | 7/25/2020 9:50 PM |  |
|  | If it goes beyond that, it should be for the best interest of the patient because I have seen these patients going into these shock episodes but after two hours they recollect themselves doctor I did not understand what you were telling me, what was it exactly so that means they have now recollected themselves so the challenge will now come, seeing a doctor will be very difficult, what are the other options where a patient willingly can come and understand what is that provision which is not there, that is where the issue is. | | | | | | | | | | | | | | | |  |
|  |  | | | | | | | | | | | | | | | |  |
| Reports\\Coding Summary By Code Report | | | | | | | | | | Page 57 of 117 | | | | | | | |
| 8/15/2023 8:10 AM | | | | | | | | | | | | | | | | | |
|  | | | **Aggregate** |  | **Classification** |  | **Coverage** |  | **Number Of Coding References** | |  | **Reference Number** |  | **Coded By Initials** |  | **Modified On** |  |
|  | | | | | | | | | | | | | | | | | |
|  | | | | | | | | | | | | 7 |  | AT |  | 7/25/2020 9:56 PM |  |
|  | I would talk about immediate disclosure when the patient is assessed to be ready to receive the information. For those few patients who may not be ready, delay should be done but be accessed within 24 hours so immediate disclose I would go with that because you will now have started the path towards care is the best. | | | | | | | | | | | | | | | |  |
|  |  | | | | | | | | | | | | | | | |  |
|  | | | | | | | | | | | | 8 |  | AT |  | 7/25/2020 9:56 PM |  |
|  | For some particular patients it is not uniform, there are those ones who would not require delay for some specific decisions but that can only happen when the doctor assesses them. | | | | | | | | | | | | | | | |  |
|  |  | | | | | | | | | | | | | | | |  |
|  | | | **Files\\IDI_ Social worker_UCI_03** | | | | | | | | | | | | | |  |
|  |  |  | No |  |  |  | 0.0035 |  | 1 | |  | | | | | |  |
|  | | |  |  |  |  |  |  |  | |  | | | | | | |
|  | | | | | | | | | | | | 1 |  | AT |  | 7/26/2020 8:40 AM |  |
|  | To me I can’t clearly tell that but one of the reasons is that if you think that the patient will be very scared and two, they think that the patient | | | | | | | | | | | | | | | |  |
|  |  | | | | | | | | | | | | | | | |  |
|  | | | **Files\\IDI_ _ Nurse_UCI_04** | | | | | | | | | | | | | |  |
|  |  |  | No |  |  |  | 0.0148 |  | 1 | |  | | | | | |  |
|  | | |  |  |  |  |  |  |  | |  | | | | | | |
|  | | | | | | | | | | | | 1 |  | AT |  | 7/26/2020 10:02 AM |  |
|  | One of the thing that tends to guide us on the disclosure of the patient’s disease and condition, one it can be dependent on the type of disease like in terms of the females for example you get a female who has got chorial carcinoma, now this is kind of a condition that is easily heal once you know it aggressively being attended to now you have to, you want to achieve possibility yet knowing there and then so if you still keep information to you self then it may not be quite easy. | | | | | | | | | | | | | | | |  |
|  |  | | | | | | | | | | | | | | | |  |
|  | | | **Files\\IDI__ Doctor_ UCI_ 05** | | | | | | | | | | | | | |  |
|  |  |  | No |  |  |  | 0.0155 |  | 2 | |  | | | | | |  |
|  | | |  |  |  |  |  |  |  | |  | | | | | | |
|  | | | | | | | | | | | | 1 |  | AT |  | 7/26/2020 10:35 AM |  |
|  | Really it is the environment but also the patient’s appearance so it’s the whole scenario between me and the patient is what guides me | | | | | | | | | | | | | | | |  |
|  |  | | | | | | | | | | | | | | | |  |
|  | | | | | | | | | | | | 2 |  | AT |  | 7/26/2020 10:36 AM |  |
|  | I like I said for question 2 if the patient tells me that I don’t want this person to know then I may delay but its my responsibility as the primary care provider to give as much information as possible but also I am just trying to think of a situation where a particular patient has advanced disease, cant talk may be that but still I usually want to know who the next of kin is because its their responsibility as well to know what’s going on in the case of their patient. | | | | | | | | | | | | | | | |  |
|  |  | | | | | | | | | | | | | | | |  |
|  | | | | | | | | | | | | | | | | | |
|  | | | | | | | | | | | | | | | | | |
| Reports\\Coding Summary By Code Report | | | | | | | | | | Page 58 of 117 | | | | | | | |
| 8/15/2023 8:10 AM | | | | | | | | | | | | | | | | | |
|  | | | **Aggregate** |  | **Classification** |  | **Coverage** |  | **Number Of Coding References** | |  | **Reference Number** |  | **Coded By Initials** |  | **Modified On** |  |
|  | **Nodes\\Enablers of immediate disclosure and informed consent process\Availability of appropriate consent document** | | | | | | | | | | | | | | | |  |
|  | | **Document** | | | | | | | | | | | | | | |  |
|  | | | **Files\\IDI_ Nurse_UCI_02** | | | | | | | | | | | | | |  |
|  |  |  | No |  |  |  | 0.0064 |  | 1 | |  | | | | | |  |
|  | | |  |  |  |  |  |  |  | |  | | | | | | |
|  | | | | | | | | | | | | 1 |  | AT |  | 7/26/2020 9:34 AM |  |
|  | On that new consent form, they talk to you about the kind of surgery they are going to do, what are the side effects of that surgery then the time you are going to spend here then you agree after they have told you everything. | | | | | | | | | | | | | | | |  |
|  |  | | | | | | | | | | | | | | | |  |
|  | **Nodes\\Enablers of immediate disclosure and informed consent process\Calm environment** | | | | | | | | | | | | | | | |  |
|  | | **Document** | | | | | | | | | | | | | | |  |
|  | | | **Files\\IDI- - Health Educator- UCI-09** | | | | | | | | | | | | | |  |
|  |  |  | No |  |  |  | 0.0007 |  | 1 | |  | | | | | |  |
|  | | |  |  |  |  |  |  |  | |  | | | | | | |
|  | | | | | | | | | | | | 1 |  | AT |  | 7/25/2020 9:27 PM |  |
|  | Many times, ok disclosure requires a calm environment, | | | | | | | | | | | | | | | |  |
|  |  | | | | | | | | | | | | | | | |  |
|  | **Nodes\\Enablers of immediate disclosure and informed consent process\Drug availability** | | | | | | | | | | | | | | | |  |
|  | | **Document** | | | | | | | | | | | | | | |  |
|  | | | **Files\\IDI_ Social worker_UCI_03** | | | | | | | | | | | | | |  |
|  |  |  | No |  |  |  | 0.0112 |  | 1 | |  | | | | | |  |
|  | | |  |  |  |  |  |  |  | |  | | | | | | |
|  | | | | | | | | | | | | 1 |  | AT |  | 7/26/2020 8:42 AM |  |
|  | I think also it is because of the drug that is available to start the patient on, because if the drug is available, they are more willing to try and give the patients better details on what the patient is going through and that the treatment is available but when the treatment is off and not expensive and available. They will decide what to start the patient on perhaps just to try and get through palliative care and then don’t disclose much more concerning their treatment. | | | | | | | | | | | | | | | |  |
|  |  | | | | | | | | | | | | | | | |  |
|  | | | | | | | | | | | | | | | | | |
| Reports\\Coding Summary By Code Report | | | | | | | | | | Page 59 of 117 | | | | | | | |
| 8/15/2023 8:10 AM | | | | | | | | | | | | | | | | | |
|  | | | **Aggregate** |  | **Classification** |  | **Coverage** |  | **Number Of Coding References** | |  | **Reference Number** |  | **Coded By Initials** |  | **Modified On** |  |
|  | **Nodes\\Enablers of immediate disclosure and informed consent process\Good body language for patient** | | | | | | | | | | | | | | | |  |
|  | | **Document** | | | | | | | | | | | | | | |  |
|  | | | **Files\\IDI -Specialist palliative care_10** | | | | | | | | | | | | | |  |
|  |  |  | No |  |  |  | 0.0069 |  | 1 | |  | | | | | |  |
|  | | |  |  |  |  |  |  |  | |  | | | | | | |
|  | | | | | | | | | | | | 1 |  | AT |  | 7/24/2020 9:37 AM |  |
|  | you will see someone’s body language, their reaction and will see how much you can talk about so it may not be worth giving them the prognosis at that time when you see that it is not the right time, you need to give them time to absorb the first information | | | | | | | | | | | | | | | |  |
|  |  | | | | | | | | | | | | | | | |  |
|  | **Nodes\\Enablers of immediate disclosure and informed consent process\Good collaboration with family members** | | | | | | | | | | | | | | | |  |
|  | | **Document** | | | | | | | | | | | | | | |  |
|  | | | **Files\\IDI - - Doctor- UCI -08** | | | | | | | | | | | | | |  |
|  |  |  | No |  |  |  | 0.0230 |  | 2 | |  | | | | | |  |
|  | | |  |  |  |  |  |  |  | |  | | | | | | |
|  | | | | | | | | | | | | 1 |  | AT |  | 7/24/2020 8:03 AM |  |
|  | Especially when you’re not going to do much to add on to the patient’s condition , the person is too sick, the disease is stage four they have come to the hospital and you think you don’t want to waste their time, money and many other things then you call them and have a conference with them, make a family conference then you can reach to them and disclose it, those are the instances. | | | | | | | | | | | | | | | |  |
|  |  | | | | | | | | | | | | | | | |  |
|  | | | | | | | | | | | | 2 |  | AT |  | 7/24/2020 8:07 AM |  |
|  | It is not good and if we don’t do it unless the patient doesn’t have an attendant and the patient cannot talk and staff because we depend so much on what the patient says so there must communication so usually communication even if not the patient but the attendant at least. | | | | | | | | | | | | | | | |  |
|  |  | | | | | | | | | | | | | | | |  |
|  | | | **Files\\IDI - _ Doctor_UCI_06** | | | | | | | | | | | | | |  |
|  |  |  | No |  |  |  | 0.0277 |  | 3 | |  | | | | | |  |
|  | | |  |  |  |  |  |  |  | |  | | | | | | |
|  | | | | | | | | | | | | 1 |  | AT |  | 7/24/2020 9:19 AM |  |
|  | when we gudge that the patient’s mental state is not sound then we may listen to the family | | | | | | | | | | | | | | | |  |
|  |  | | | | | | | | | | | | | | | |  |
|  | | | | | | | | | | | | 2 |  | AT |  | 7/24/2020 9:20 AM |  |
|  | Its very important because it avoids the confusion and tensions that are arise in the family when the patient is very sick or even later on when the patient dies, its not a will per say because for it, it is medical care only and that speaks around the diagnosis but helps because there are patients who get cardiac arrest some patients may not want to be resastated they say this is a terminal problem I want to go but the family may say no get them back, resastate them we want them back so in such a situation what do you do or a patient is in comma for so long in an ICU are you going to keep them in the intensive care unit forever so those are the aspects of care that I think can be improved right now in our setting especially in cancer care. | | | | | | | | | | | | | | | |  |
|  |  | | | | | | | | | | | | | | | |  |
|  | | | | | | | | | | | | 3 |  | AT |  | 7/24/2020 9:21 AM |  |
|  | Because you see these family tensions here once they learn that the patient is so sick. | | | | | | | | | | | | | | | |  |
|  |  | | | | | | | | | | | | | | | |  |
| Reports\\Coding Summary By Code Report | | | | | | | | | | Page 60 of 117 | | | | | | | |
| 8/15/2023 8:10 AM | | | | | | | | | | | | | | | | | |
|  | | | **Aggregate** |  | **Classification** |  | **Coverage** |  | **Number Of Coding References** | |  | **Reference Number** |  | **Coded By Initials** |  | **Modified On** |  |
|  | | | **Files\\IDI -Specialist palliative care_10** | | | | | | | | | | | | | |  |
|  |  |  | No |  |  |  | 0.097 |  | 1 | |  | | | | | |  |
|  | | |  |  |  |  |  |  |  | |  | | | | | | |
|  | | | | | | | | | | | | 1 |  | AT |  | 7/24/2020 9:39 AM |  |
|  | Yeah, but I cant speak for them because I can guess because the reason why people may lie is when they feel that may be like I don’t know like attachment to the patient, someone may have been a good friend of a patient and say I am going to disappoint them or I am going to be blamed because I didn’t say this earlier I think that is when the lying may come in. | | | | | | | | | | | | | | | |  |
|  |  | | | | | | | | | | | | | | | |  |
|  | | | **Files\\IDI- - Health Educator- UCI-09** | | | | | | | | | | | | | |  |
|  |  |  | No |  |  |  | 0.0298 |  | 2 | |  | | | | | |  |
|  | | |  |  |  |  |  |  |  | |  | | | | | | |
|  | | | | | | | | | | | | 1 |  | AT |  | 7/24/2020 12:45 PM |  |
|  | So in most cases there is what we call a family conference where the doctor after evaluating the patient with the nurses says this patient can no longer benefit from treatment, at the stage of this disease this person cannot benefit from treatment. In the other way he understands that this person may not leave with this cancer and will die with this cancer so why is it difficult someone left home to come and get treatment in the hospital and now you are saying the hospital cannot treat you, you need to go back home so the doctor calls a family conference and brings out the treatment option | | | | | | | | | | | | | | | |  |
|  |  | | | | | | | | | | | | | | | |  |
|  | | | | | | | | | | | | 2 |  | AT |  | 7/25/2020 10:35 PM |  |
|  | Now there is one I had said, a family is part of the care supporters which needs to be on top but I am saying sometimes, families are left out, how will this mother tell her lovely daughter that I have cancer. This is the mother who walked to the hospital with a sister and the sister has known but this mother has now been left with a challenge of going to tell her children that she has been found with cancer so we do not prepare patients to communicate to the relatives and the loved ones which is a very big thing, you will find a mother hiding and children who are observant will see a mother not looking good so we need to train these people how to disclose their cancer situation to the relatives especially the loved ones and the children but on the side when they are in the institute here always disclosure has to be known to them they need to know that their mother, their father or brother or sister or child has cancer because disclosure at that point is one way of soliciting for support so now this person cancer he is cared for in this way, will require this kind of food, needs to be walked around, needs to be kept clean which is your role as a family so from a hospital setting, disclosure is always by a health worker but very few attendants come to the hospital now that patient is left with a challenge of disclosing it to the family members, at home how will I tell them I have cancer and they know cancer is for everyone. Then cancer gives everyone the immediate question so you’re going to die mama? | | | | | | | | | | | | | | | |  |
|  |  |  |  |  |  |  |  |  |  |  |  |  |  |  |  |  |  |
|  |  | | | | | | | | | | | | | | | |  |
|  | | | **Files\\IDI__ Doctor_ UCI_ 05** | | | | | | | | | | | | | |  |
|  |  |  | No |  |  |  | 0.0241 |  | 3 | |  | | | | | |  |
|  | | |  |  |  |  |  |  |  | |  | | | | | | |
|  | | | | | | | | | | | | 1 |  | AT |  | 7/26/2020 10:54 AM |  |
|  | But also, we do family conferences in cancer treatment especially when we realise that the patient is may be is having challenges making some decisions we prefer to bring a bigger family and try to talk about the same thing and may be try to find out why the patient is declining in receiving whatever we give the patient. | | | | | | | | | | | | | | | |  |
|  |  | | | | | | | | | | | | | | | |  |
|  | | | | | | | | | | | | 2 |  | AT |  | 7/26/2020 10:58 AM |  |
|  | Even family, once you have cancer, the whole family is affected whether its social, whether psychologically in terms of stress, whether its economic in terms of transport, feeding, treatment, whether it is spiritual the whole family is affected even health wise because am thinking about a specific scenario where a sole bread winner for the whole family gets affected and needs to be admitted, hospitalized for weeks or months, really the family gets affected so in the different far sets families are affected. | | | | | | | | | | | | | | | |  |
|  |  | | | | | | | | | | | | | | | |  |
|  | | | | | | | | | | | | 3 |  | AT |  | 7/26/2020 11:00 AM |  |
|  | Cancer treatment is a team effort, it’s for the whole team, really its for the whole team that’s how I put it. | | | | | | | | | | | | | | | |  |
|  |  | | | | | | | | | | | | | | | |  |
|  | | | | | | | | | | | | | | | | | |
| Reports\\Coding Summary By Code Report | | | | | | | | | | Page 61 of 117 | | | | | | | |
| 8/15/2023 8:10 AM | | | | | | | | | | | | | | | | | |
|  | | | **Aggregate** |  | **Classification** |  | **Coverage** |  | **Number Of Coding References** | |  | **Reference Number** |  | **Coded By Initials** |  | **Modified On** |  |
|  | **Nodes\\Enablers of immediate disclosure and informed consent process\Good mental capacity of Patient** | | | | | | | | | | | | | | | |  |
|  | | **Document** | | | | | | | | | | | | | | |  |
|  | | | **Files\\IDI - - Doctor- UCI -08** | | | | | | | | | | | | | |  |
|  |  |  | No |  |  |  | 0.0763 |  | 7 | |  | | | | | |  |
|  | | |  |  |  |  |  |  |  | |  | | | | | | |
|  | | | | | | | | | | | | 1 |  | AT |  | 7/24/2020 8:02 AM |  |
|  | The patient is not talking so you first manage the patient, if the person is too sick, when the patient is confused or having a mental problem and you don’t see a proper attendant to disclose to. | | | | | | | | | | | | | | | |  |
|  |  | | | | | | | | | | | | | | | |  |
|  | | | | | | | | | | | | 2 |  | AT |  | 7/24/2020 8:03 AM |  |
|  | I think its not ideal for every patient to get their information but as I told you sometimes it is the way how I perceive how you’re taking the information because there are instances where people have been given information and that’s when they stop talking and they died. | | | | | | | | | | | | | | | |  |
|  |  | | | | | | | | | | | | | | | |  |
|  | | | | | | | | | | | | 3 |  | AT |  | 7/24/2020 8:04 AM |  |
|  | Not immediately but there was a medical personnel actually a nurse who was sick with lymphoma and she was asking this was Dr Omoding way back in 2013, so doctor what is the stage of my disease and she was very anxious but Dr Omoding just had to tell her, that was the last time she talked to us so there is need to weigh. Just weigh the patient’s reaction to information, others you tell them this thing is very good they are too happy when you say this thing is not very good so they have to be. | | | | | | | | | | | | | | | |  |
|  |  | | | | | | | | | | | | | | | |  |
|  | | | | | | | | | | | | 4 |  | AT |  | 7/24/2020 8:08 AM |  |
|  | People can get in to depression, people will loose hope and when you loose hope and your depressed your immunity goes down it can also be a problem for your treatment for the uptake of the treatment and then the outcome. | | | | | | | | | | | | | | | |  |
|  |  | | | | | | | | | | | | | | | |  |
|  | | | | | | | | | | | | 5 |  | AT |  | 7/24/2020 8:09 AM |  |
|  | You avoid people getting in to depression. You allow the information to sink because they also realise like some come and they don’t know they have cancer but they are in cancer institute and they will even open a file then they ask is it really true that I have cancer so you see its already confirmed. | | | | | | | | | | | | | | | |  |
|  |  | | | | | | | | | | | | | | | |  |
|  | | | | | | | | | | | | 6 |  | AT |  | 7/24/2020 8:15 AM |  |
|  | I think the best practice is for all of those to be in play, the doctor or the nurse should have all those at play. Ideal is to have the patient have their information but it may not be at the initial or the first time you’re meeting them or the first review to give everything and as I told it depends on how perceive the understanding or the way this patient takes information so the doctor or the nurse should know when to give all the information at the first time when to give some and hold some and when to postpone giving information but at the end of it the patient should have their information. | | | | | | | | | | | | | | | |  |
|  |  | | | | | | | | | | | | | | | |  |
|  | | | | | | | | | | | | 7 |  | AT |  | 7/24/2020 8:24 AM |  |
|  | to others when they receive that they even commit suicide, others become depressed and come with many things | | | | | | | | | | | | | | | |  |
|  |  | | | | | | | | | | | | | | | |  |
|  | | | **Files\\IDI - _ Doctor_UCI_06** | | | | | | | | | | | | | |  |
|  |  |  | No |  |  |  | 0.0454 |  | 3 | |  | | | | | |  |
|  | | |  |  |  |  |  |  |  | |  | | | | | | |
|  | | | | | | | | | | | | 1 |  | AT |  | 7/24/2020 8:38 AM |  |
|  | A good number of times its at the discretion of the health worker, you will see a patient talk to them and having a general discussion you will get to know the general performance status of the mental health aspect of that patient. There are some patients who I must say very few of them, they are very anxious, there is a lot of anxiety, fear and worry but even those what I find about most of our patients they want to know what is disturbing them that’s the most positive thing so there it is easy that you can start a discussion. | | | | | | | | | | | | | | | |  |
|  |  | | | | | | | | | | | | | | | |  |
|  | | | | | | | | | | | | 2 |  | AT |  | 7/24/2020 8:40 AM |  |
|  | Immediate disclosure is challenging for a patient who is coming in for the very first time more so if they really don’t know any thing about what’s happening or they have been given storage results it may be over whelming for the patient to be told you have an advanced stage, the treatment is going to be palliative, there is no cure and they are going to have all these side effects, that can be very overwhelming for a patient. So that affects the quality of mental health, you become anxious, fearful, afraid. Depression is a big factor in cancer care so those are the challenges that you have but there is a lot of information being given to a patient who is not psychologically ready. | | | | | | | | | | | | | | | |  |
|  |  | | | | | | | | | | | | | | | |  |
| Reports\\Coding Summary By Code Report | | | | | | | | | | Page 62 of 117 | | | | | | | |
| 8/15/2023 8:10 AM | | | | | | | | | | | | | | | | | |
|  | | | **Aggregate** |  | **Classification** |  | **Coverage** |  | **Number Of Coding References** | |  | **Reference Number** |  | **Coded By Initials** |  | **Modified On** |  |
|  | | | | | | | | | | | | | | | | | |
|  | | | | | | | | | | | | 3 |  | AT |  | 7/24/2020 8:44 AM |  |
|  | when you have a very terminally ill patient, a very advanced and you gauge that his or her mental health is not adequate enough to take in this information or where you judge that cost of giving the information more harmful than the benefit of the patient so sometimes you may differ on that information. | | | | | | | | | | | | | | | |  |
|  |  | | | | | | | | | | | | | | | |  |
|  | | | **Files\\IDI- - Health Educator- UCI-09** | | | | | | | | | | | | | |  |
|  |  |  | No |  |  |  | 0.0051 |  | 2 | |  | | | | | |  |
|  | | |  |  |  |  |  |  |  | |  | | | | | | |
|  | | | | | | | | | | | | 1 |  | AT |  | 7/25/2020 9:27 PM |  |
|  | disclosure is deep in the motive so sometimes the condition of the patient may not warrant disclosure at that point so the doctors may write in the file and pushes them to the nurses to disclose at an appropriate time. | | | | | | | | | | | | | | | |  |
|  |  | | | | | | | | | | | | | | | |  |
|  | | | | | | | | | | | | 2 |  | AT |  | 7/25/2020 9:34 PM |  |
|  | This patient has come when he is so sick, very sick and even if you say you are going to tell this patient she can’t understand you now on that, | | | | | | | | | | | | | | | |  |
|  |  | | | | | | | | | | | | | | | |  |
|  | **Nodes\\Enablers of immediate disclosure and informed consent process\Good rapport** | | | | | | | | | | | | | | | |  |
|  | | **Document** | | | | | | | | | | | | | | |  |
|  | | | **Files\\IDI_ _ Nurse_UCI_04** | | | | | | | | | | | | | |  |
|  |  |  | No |  |  |  | 0.0201 |  | 1 | |  | | | | | |  |
|  | | |  |  |  |  |  |  |  | |  | | | | | | |
|  | | | | | | | | | | | | 1 |  | AT |  | 7/26/2020 10:17 AM |  |
|  | Yeah, they are given chance to ask questions but inmost cases actually like the way I told you, you need to get they trust before they ask you their necessary questions because when they are in the clinical rooms, they are given chance to as questions but when they come out you find them again have like a flood of information because they tend to go to the person that they are very free and comfortable and they are always saying what about, what about this but while they were inside the room they were actually given room so I think its dependant on how you create the rapport with these patients so that these patients get to know openly talk to you. | | | | | | | | | | | | | | | |  |
|  |  | | | | | | | | | | | | | | | |  |
|  | | | | | | | | | | | | | | | | | |
|  | | | | | | | | | | | | | | | | | |
|  | | | | | | | | | | | | | | | | | |
| Reports\\Coding Summary By Code Report | | | | | | | | | | Page 63 of 117 | | | | | | | |
| 8/15/2023 8:10 AM | | | | | | | | | | | | | | | | | |
|  | | | **Aggregate** |  | **Classification** |  | **Coverage** |  | **Number Of Coding References** | |  | **Reference Number** |  | **Coded By Initials** |  | **Modified On** |  |
|  | **Nodes\\Enablers of immediate disclosure and informed consent process\Good staff attitude** | | | | | | | | | | | | | | | |  |
|  | | **Document** | | | | | | | | | | | | | | |  |
|  | | | **Files\\IDI- - Health Educator- UCI-09** | | | | | | | | | | | | | |  |
|  |  |  | No |  |  |  | 0.096 |  | 1 | |  | | | | | |  |
|  | | |  |  |  |  |  |  |  | |  | | | | | | |
|  | | | | | | | | | | | | 1 |  | AT |  | 7/25/2020 10:08 PM |  |
|  | communication creates a relationship, care needs a relationship so because of communication that relationship gap or skill, there is a relationship in care its not always so good so I see the element of communication may be specific to cancer. Sometimes we have medical terminologies that as an element of communication we are talking to lay people so we are sometimes taken unknowingly that we talk our medical terms while communicating to patients but patients do not have strength to ask that what is that you’re calling so they also now listen and do not understand that some noise are for this no, so still we communicate in those medical jargons which is not consumable to patients. | | | | | | | | | | | | | | | |  |
|  |  | | | | | | | | | | | | | | | |  |
|  | **Nodes\\Enablers of immediate disclosure and informed consent process\Gradual or partial disclosure** | | | | | | | | | | | | | | | |  |
|  | | **Document** | | | | | | | | | | | | | | |  |
|  | | | **Files\\IDI - - Doctor- UCI -08** | | | | | | | | | | | | | |  |
|  |  |  | No |  |  |  | 0.0417 |  | 5 | |  | | | | | |  |
|  | | |  |  |  |  |  |  |  | |  | | | | | | |
|  | | | | | | | | | | | | 1 |  | AT |  | 7/24/2020 8:05 AM |  |
|  | Not really lying but withholding some information then you give later maybe you can give slowly not everything at ago. | | | | | | | | | | | | | | | |  |
|  |  | | | | | | | | | | | | | | | |  |
|  | | | | | | | | | | | | 2 |  | AT |  | 7/24/2020 8:06 AM |  |
|  | there are some patients who are too anxious and then you do not want to give them the whole information because if you give it will cause more trouble but ideally it is really not protection because you’re supposed to give them | | | | | | | | | | | | | | | |  |
|  |  | | | | | | | | | | | | | | | |  |
|  | | | | | | | | | | | | 3 |  | AT |  | 7/24/2020 8:10 AM |  |
|  | One: I think delaying will allow the little information to sink slowly so they will take it well and then it will avoid making suicidal information or tendencies like that so it is good. There is one who took the whole tin of morphine she said I was feeling pain, I wanted pain to go away so she came with toxicity of morphine so we had to help then another one took Organ phosdiphen. | | | | | | | | | | | | | | | |  |
|  |  | | | | | | | | | | | | | | | |  |
|  | | | | | | | | | | | | 4 |  | AT |  | 7/24/2020 8:15 AM |  |
|  | The best is to withhold certain information actually I would strongly say lying is not good at least withholding some information yes. | | | | | | | | | | | | | | | |  |
|  |  | | | | | | | | | | | | | | | |  |
|  | | | | | | | | | | | | 5 |  | AT |  | 7/24/2020 8:16 AM |  |
|  | I would say it can be withheld for three weeks because the patients are reviewed every three weeks so I give you some today then next week or the other week you come for another encounter with the doctor again I give you more you now the challenge also when many doctors review the patient. Toady you see this another day another like that. | | | | | | | | | | | | | | | |  |
|  |  | | | | | | | | | | | | | | | |  |
|  | | | | | | | | | | | | | | | | | |
| Reports\\Coding Summary By Code Report | | | | | | | | | | Page 64 of 117 | | | | | | | |
| 8/15/2023 8:10 AM | | | | | | | | | | | | | | | | | |
|  | | | **Aggregate** |  | **Classification** |  | **Coverage** |  | **Number Of Coding References** | |  | **Reference Number** |  | **Coded By Initials** |  | **Modified On** |  |
|  | | | **Files\\IDI - _ Doctor_UCI_06** | | | | | | | | | | | | | |  |
|  |  |  | No |  |  |  | 0.0268 |  | 3 | |  | | | | | |  |
|  | | |  |  |  |  |  |  |  | |  | | | | | | |
|  | | | | | | | | | | | | 1 |  | AT |  | 7/24/2020 8:41 AM |  |
|  | Delaying disclosure has got a main advantage of allowing the patient to first probably recover or get in to better health before. | | | | | | | | | | | | | | | |  |
|  |  | | | | | | | | | | | | | | | |  |
|  | | | | | | | | | | | | 2 |  | AT |  | 7/24/2020 8:42 AM |  |
|  | We first manage some symptoms then when they are stronger, we bring out the topic and prognosis. But the disadvantage also is that there is delaying taking critical decision because we are waiting for the patient to first recover well as you may want to actually initiate radiotherapy or chemotherapy early, you will want to get the disease where it is responsible for treatment. | | | | | | | | | | | | | | | |  |
|  |  | | | | | | | | | | | | | | | |  |
|  | | | | | | | | | | | | 3 |  | AT |  | 7/24/2020 8:50 AM |  |
|  | If it is delayed I would then recommend not more than two weeks so sometimes I recommend one week I want the patient to first get better or deal with some tensions at home then come again, the hole idea is the longer we take to start treatment the symptoms will get progressively worse so me I would recommend immediate but if there are some other factors not a delay of more than two weeks. | | | | | | | | | | | | | | | |  |
|  |  | | | | | | | | | | | | | | | |  |
|  | | | **Files\\IDI -Specialist palliative care_10** | | | | | | | | | | | | | |  |
|  |  |  | No |  |  |  | 0.0594 |  | 5 | |  | | | | | |  |
|  | | |  |  |  |  |  |  |  | |  | | | | | | |
|  | | | | | | | | | | | | 1 |  | AT |  | 7/24/2020 9:32 AM |  |
|  | We tend to want to grade the information slowly so we don’t come straight away to give prognosis at the first visit and many times I think the reason why they don’t do it is that people are not aware of the stage yet,, the investigations haven’t yet been done but also if you haven’t established a good relationship yet its had to talk about those deep things unless a patient has been one who is already prepared and wants to know then there you may have to do it but its not the common thing. | | | | | | | | | | | | | | | |  |
|  |  | | | | | | | | | | | | | | | |  |
|  | | | | | | | | | | | | 2 |  | AT |  | 7/24/2020 9:35 AM |  |
|  | Most of the time the prognosis is not easy to tell from the first visit and many times it is not something that we can discuss and I think it is probably not easy for many people to go to that discussion because it needs a lot of time and also sometimes patients are not ready to hear the prognosis as long they know the diagnosis and treatment sometimes some people are comfortable just to stay with that but most of the time the people who will know their prognosis are the ones who are very sick because then everyone realizes that oh we have to tell this patient now what the goals of care are but otherwise if they are stable they will be told the prognosis if they have been seen more than once in a few weeks. | | | | | | | | | | | | | | | |  |
|  |  | | | | | | | | | | | | | | | |  |
|  | | | | | | | | | | | | 3 |  | AT |  | 7/24/2020 9:38 AM |  |
|  | If the patient is so sick you can delay down the disclosure so sick or sometimes the relatives are in your way blocking you from taking the information and so are the investigations I talked about here. | | | | | | | | | | | | | | | |  |
|  |  | | | | | | | | | | | | | | | |  |
|  | | | | | | | | | | | | 4 |  | AT |  | 7/24/2020 9:41 AM |  |
|  | Disclosure is an advantage to a patient if they are ready to hear the news but also when to do it whether immediately or later it should be a judgement call because the skill of giving information is to know when to give information at an appropriate time to give the information but of course if you’re delaying just because you fear then that’s a problem but if your delaying just to see that patient is in a condition not to receive it then it is okay to delay it. | | | | | | | | | | | | | | | |  |
|  |  | | | | | | | | | | | | | | | |  |
|  | | | | | | | | | | | | 5 |  | AT |  | 7/24/2020 9:49 AM |  |
|  | so it would be nice if they are at the information or chance for people to access information in different ways like yes I have talked to you today first time I have seen you or second time but now you go home and seek out more as you adjust to your illness and to the news and many times there are no opportunities to ask | | | | | | | | | | | | | | | |  |
|  |  | | | | | | | | | | | | | | | |  |
|  | | | | | | | | | | | | | | | | | |
| Reports\\Coding Summary By Code Report | | | | | | | | | | Page 65 of 117 | | | | | | | |
| 8/15/2023 8:10 AM | | | | | | | | | | | | | | | | | |
|  | | | **Aggregate** |  | **Classification** |  | **Coverage** |  | **Number Of Coding References** | |  | **Reference Number** |  | **Coded By Initials** |  | **Modified On** |  |
|  | | | **Files\\IDI_ Social worker_UCI_03** | | | | | | | | | | | | | |  |
|  |  |  | No |  |  |  | 0.088 |  | 2 | |  | | | | | |  |
|  | | |  |  |  |  |  |  |  | |  | | | | | | |
|  | | | | | | | | | | | | 1 |  | AT |  | 7/26/2020 8:49 AM |  |
|  | The advantages of delaying disclosing may not be much because that would also mean in a way shocking the patient. | | | | | | | | | | | | | | | |  |
|  |  | | | | | | | | | | | | | | | |  |
|  | | | | | | | | | | | | 2 |  | AT |  | 7/26/2020 8:50 AM |  |
|  | If it is delayed the patient might think they are ok, if they think it will heal so they put more zeal they want to focus they want to try all they can at theta particular point yet the truth is that whatever they are going to do is in vain when you delay it. | | | | | | | | | | | | | | | |  |
|  |  | | | | | | | | | | | | | | | |  |
|  | | | **Files\\IDI_ Nurse_UCI_02** | | | | | | | | | | | | | |  |
|  |  |  | No |  |  |  | 0.0118 |  | 2 | |  | | | | | |  |
|  | | |  |  |  |  |  |  |  | |  | | | | | | |
|  | | | | | | | | | | | | 1 |  | AT |  | 7/26/2020 9:29 AM |  |
|  | Immediately depends on the condition you’ve come in, you can come a very bad condition, then am like let her first stabilize it is ongoing, information can be ongoing then there is that immediate information I go for both. | | | | | | | | | | | | | | | |  |
|  |  | | | | | | | | | | | | | | | |  |
|  | | | | | | | | | | | | 2 |  | AT |  | 7/26/2020 9:31 AM |  |
|  | I tell you as you come because when you come to me and I disclose to you this is this, this is this, I don’t think you will come back so I would delay and then on going I tell slowly by slowly. | | | | | | | | | | | | | | | |  |
|  |  | | | | | | | | | | | | | | | |  |
|  | | | **Files\\IDI__ Doctor_ UCI_ 05** | | | | | | | | | | | | | |  |
|  |  |  | No |  |  |  | 0.0060 |  | 1 | |  | | | | | |  |
|  | | |  |  |  |  |  |  |  | |  | | | | | | |
|  | | | | | | | | | | | | 1 |  | AT |  | 7/26/2020 10:42 AM |  |
|  | I think giving in bits is very good as well, that’s where the medical officers continue seeing the patients play another big role as well so you trying to reinforce the information so you just continue repeating, I think that is important. | | | | | | | | | | | | | | | |  |
|  |  | | | | | | | | | | | | | | | |  |
|  | **Nodes\\Enablers of immediate disclosure and informed consent process\Respect for Patient’s autonomy** | | | | | | | | | | | | | | | |  |
|  | | **Document** | | | | | | | | | | | | | | |  |
|  | | | **Files\\IDI -Specialist palliative care_10** | | | | | | | | | | | | | |  |
|  |  |  | No |  |  |  | 0.0193 |  | 3 | |  | | | | | |  |
|  | | |  |  |  |  |  |  |  | |  | | | | | | |
|  | | | | | | | | | | | | 1 |  | AT |  | 7/24/2020 9:45 AM |  |
|  | I think that it was in the past but now people’s autonomy being a very dominant future ethics things have changed. | | | | | | | | | | | | | | | |  |
|  |  | | | | | | | | | | | | | | | |  |
|  | | | | | | | | | | | | 2 |  | AT |  | 7/24/2020 9:45 AM |  |
|  | Of course, in the past when there was this holistic approach that would be sort of paternalistic, but people started arguing that how do you know that it is the best for me I have a right to make my decision to determine what is best for me. | | | | | | | | | | | | | | | |  |
|  |  | | | | | | | | | | | | | | | |  |
| Reports\\Coding Summary By Code Report | | | | | | | | | | Page 66 of 117 | | | | | | | |
| 8/15/2023 8:10 AM | | | | | | | | | | | | | | | | | |
|  | | | **Aggregate** |  | **Classification** |  | **Coverage** |  | **Number Of Coding References** | |  | **Reference Number** |  | **Coded By Initials** |  | **Modified On** |  |
|  | | | | | | | | | | | | | | | | | |
|  | | | | | | | | | | | | 3 |  | AT |  | 7/24/2020 9:45 AM |  |
|  | For me first of all there is no one size fits all, but I think the things that are out maybe are lying which shouldn’t be part of practice but whatever that is made would be good but you have to take in to consideration that if the person really wants to know is another thing because there are people who want to know and you will be violating their autonomy | | | | | | | | | | | | | | | |  |
|  |  | | | | | | | | | | | | | | | |  |
|  | **Nodes\\Enablers of immediate disclosure and informed consent process\Training** | | | | | | | | | | | | | | | |  |
|  | | **Document** | | | | | | | | | | | | | | |  |
|  | | | **Files\\IDI- - Health Educator- UCI-09** | | | | | | | | | | | | | |  |
|  |  |  | No |  |  |  | 0.004 |  | 1 | |  | | | | | |  |
|  | | |  |  |  |  |  |  |  | |  | | | | | | |
|  | | | | | | | | | | | | 1 |  | AT |  | 7/25/2020 10:10 PM |  |
|  | Time and sometimes the skills. | | | | | | | | | | | | | | | |  |
|  |  | | | | | | | | | | | | | | | |  |
|  | **Nodes\\Enablers of immediate disclosure and informed consent process\Understanding pricnciples of breaking bad news** | | | | | | | | | | | | | | | |  |
|  | | **Document** | | | | | | | | | | | | | | |  |
|  | | | **Files\\IDI -Specialist palliative care_10** | | | | | | | | | | | | | |  |
|  |  |  | No |  |  |  | 0.0172 |  | 1 | |  | | | | | |  |
|  | | |  |  |  |  |  |  |  | |  | | | | | | |
|  | | | | | | | | | | | | 1 |  | AT |  | 7/24/2020 9:36 AM |  |
|  | Yeah, just understanding the principles of breaking bad news you have to know how much information you give at a go. For some people it may be easier to get to the point where you give the prognosis. To many it may not be prudent for you to give so much information even up to the level of prognosis the first time you see the patient because they may even be shocked that they even have that diagnosis in the first place and we know that if somebody has not suspected a diagnosis and you give them too much information, they will only hear the diagnosis and the rest will not record it also to know how much to give at a particular time. | | | | | | | | | | | | | | | |  |
|  |  | | | | | | | | | | | | | | | |  |
|  | | | | | | | | | | | | | | | | | |
|  | | | | | | | | | | | | | | | | | |
| Reports\\Coding Summary By Code Report | | | | | | | | | | Page 67 of 117 | | | | | | | |
| 8/15/2023 8:10 AM | | | | | | | | | | | | | | | | | |
|  | | | **Aggregate** |  | **Classification** |  | **Coverage** |  | **Number Of Coding References** | |  | **Reference Number** |  | **Coded By Initials** |  | **Modified On** |  |
|  | **Nodes\\How informed consent or other information is communicated\Blanket consenting** | | | | | | | | | | | | | | | |  |
|  | | **Document** | | | | | | | | | | | | | | |  |
|  | | | **Files\\IDI - _ Doctor_UCI_06** | | | | | | | | | | | | | |  |
|  |  |  | No |  |  |  | 0.0161 |  | 1 | |  | | | | | |  |
|  | | |  |  |  |  |  |  |  | |  | | | | | | |
|  | | | | | | | | | | | | 1 |  | AT |  | 7/24/2020 8:53 AM |  |
|  | Normally this informed consent is obtained, its not cancer specific its for general purpose treatment when opening a file, there is a sheet generally about the care they are going to get and asking for permission but I must say that cancer specific consent forms are not generally but I will tell like when you are going to do surgery there is a separate consent form for a patient and that consent form again will give that information of what they are going to do, what possible complications may arise in future both immediate and after. | | | | | | | | | | | | | | | |  |
|  |  | | | | | | | | | | | | | | | |  |
|  | | | **Files\\IDI -Specialist palliative care_10** | | | | | | | | | | | | | |  |
|  |  |  | No |  |  |  | 0.080 |  | 1 | |  | | | | | |  |
|  | | |  |  |  |  |  |  |  | |  | | | | | | |
|  | | | | | | | | | | | | 1 |  | AT |  | 7/24/2020 9:51 AM |  |
|  | I don’t know if they really understand but I think the blanket informed consent as what we use in our hospitals probably is not enough because we make them sign in the beginning that they will accept all the treatment given and also all the procedures and that it but even it’s a blanket consent. | | | | | | | | | | | | | | | |  |
|  |  | | | | | | | | | | | | | | | |  |
|  | | | **Files\\IDI- - Health Educator- UCI-09** | | | | | | | | | | | | | |  |
|  |  |  | No |  |  |  | 0.0258 |  | 4 | |  | | | | | |  |
|  | | |  |  |  |  |  |  |  | |  | | | | | | |
|  | | | | | | | | | | | | 1 |  | AT |  | 7/25/2020 10:02 PM |  |
|  | When they are going for treatment the doctor has said you are going for treatment, at that point they must interface with a counselor or a nurse who will take them from the perspective of treatment and they must agree to that treatment after through inspection so that is what I am talking about at this point information is very minimal, patients are not given chance to understand or ask questions so generally the consent to treat has been highly abused because who ever has been seen from the papers that has consented from the papers but has no knowledge so is that informed consent. | | | | | | | | | | | | | | | |  |
|  |  | | | | | | | | | | | | | | | |  |
|  | | | | | | | | | | | | 2 |  | AT |  | 7/25/2020 10:03 PM |  |
|  | I have also known of the consent that is obtained at records where a patient signs records and that today he has been enrolled in to care when they are opening the file. I did not take as informed consent because I realized that making a patient accept for their details to be taken for purposes of care, I have seen them sign. | | | | | | | | | | | | | | | |  |
|  |  | | | | | | | | | | | | | | | |  |
|  | | | | | | | | | | | | 3 |  | AT |  | 7/25/2020 10:13 PM |  |
|  | First of all, I’m wondering what is the purpose of consent at the records point? What is it that they are consenting for? For giving their baseline information or it is now the general consent that will be given across that you accepted? Then it is wrong because I can accept one element and refuse the other one, I can accept the doctor the doctor to see me but refuse to take the treatment, so consent has to be done at important points. | | | | | | | | | | | | | | | |  |
|  |  | | | | | | | | | | | | | | | |  |
|  | | | | | | | | | | | | 4 |  | AT |  | 7/25/2020 10:14 PM |  |
|  | There is a danger, I am consenting to receive the services but my opinion and consent must be sought from intervention, I consented to allow you take my information, to allow the doctor examine me but I have not consented to take my body tissue, I have not consented that you give me treatment so specific so that is now if someone finds that details of what you are consenting about must be got out in those consents, if the details are not there then it is not the right one. | | | | | | | | | | | | | | | |  |
|  |  | | | | | | | | | | | | | | | |  |
|  | | | | | | | | | | | | | | | | | |
| Reports\\Coding Summary By Code Report | | | | | | | | | | Page 68 of 117 | | | | | | | |
| 8/15/2023 8:10 AM | | | | | | | | | | | | | | | | | |
|  | | | **Aggregate** |  | **Classification** |  | **Coverage** |  | **Number Of Coding References** | |  | **Reference Number** |  | **Coded By Initials** |  | **Modified On** |  |
|  | **Nodes\\How informed consent or other information is communicated\Both verbally and written consent** | | | | | | | | | | | | | | | |  |
|  | | **Document** | | | | | | | | | | | | | | |  |
|  | | | **Files\\IDI- - Health Educator- UCI-09** | | | | | | | | | | | | | |  |
|  |  |  | No |  |  |  | 0.048 |  | 1 | |  | | | | | |  |
|  | | |  |  |  |  |  |  |  | |  | | | | | | |
|  | | | | | | | | | | | | 1 |  | AT |  | 7/25/2020 10:13 PM |  |
|  | The most important point is when they are taking tissues as specimens, I really feel this has to happen properly, when surgery is going to take place, its part of treatment, it has to happen, when someone is going to be introduced to chemo, it has to happen so am looking into those critical moments when intervention will occur on the patient. | | | | | | | | | | | | | | | |  |
|  |  | | | | | | | | | | | | | | | |  |
|  | **Nodes\\How informed consent or other information is communicated\Direct disclosure** | | | | | | | | | | | | | | | |  |
|  | | **Document** | | | | | | | | | | | | | | |  |
|  | | | **Files\\IDI__ Doctor_ UCI_ 05** | | | | | | | | | | | | | |  |
|  |  |  | No |  |  |  | 0.0119 |  | 1 | |  | | | | | |  |
|  | | |  |  |  |  |  |  |  | |  | | | | | | |
|  | | | | | | | | | | | | 1 |  | AT |  | 7/26/2020 10:34 AM |  |
|  | Most of the times patients come with care takers, and there may be a few patients who may say I don’t want my people to know the disease I have and then there are those few patients especially who come from up country who may come alone. However, my job is to give all the information to the patient but I also need to respect the patient, if a patient is not comfortable with a particular person knowing the information then we usually ask them to step out of the room. | | | | | | | | | | | | | | | |  |
|  |  | | | | | | | | | | | | | | | |  |
|  | **Nodes\\How informed consent or other information is communicated\Goup health education** | | | | | | | | | | | | | | | |  |
|  | | **Document** | | | | | | | | | | | | | | |  |
|  | | | **Files\\IDI- - Health Educator- UCI-09** | | | | | | | | | | | | | |  |
|  |  |  | No |  |  |  | 0.0060 |  | 1 | |  | | | | | |  |
|  | | |  |  |  |  |  |  |  | |  | | | | | | |
|  | | | | | | | | | | | | 1 |  | AT |  | 7/24/2020 12:33 PM |  |
|  | There are junior doctors who clerk patients whenever they come so when the patient comes to the institute the records officers register them then group education is always given but it is not consistent now after group health education, the nurses now take them to the doctor who reveal their information, take the history, examine them and use the pathology report to tell these patients the diagnosis of cancer that patient has. | | | | | | | | | | | | | | | |  |
|  |  | | | | | | | | | | | | | | | |  |
|  | | | | | | | | | | | | | | | | | |
| Reports\\Coding Summary By Code Report | | | | | | | | | | Page 69 of 117 | | | | | | | |
| 8/15/2023 8:10 AM | | | | | | | | | | | | | | | | | |
|  | | | **Aggregate** |  | **Classification** |  | **Coverage** |  | **Number Of Coding References** | |  | **Reference Number** |  | **Coded By Initials** |  | **Modified On** |  |
|  | | | **Files\\IDI__ Doctor_ UCI_ 05** | | | | | | | | | | | | | |  |
|  |  |  | No |  |  |  | 0.0124 |  | 2 | |  | | | | | |  |
|  | | |  |  |  |  |  |  |  | |  | | | | | | |
|  | | | | | | | | | | | | 1 |  | AT |  | 7/26/2020 10:51 AM |  |
|  | I think they are utilized because not all patients but some patients come and tell me oh they gave me a book, oh we had education done in the morning and they were telling me this, is it true? | | | | | | | | | | | | | | | |  |
|  |  | | | | | | | | | | | | | | | |  |
|  | | | | | | | | | | | | 2 |  | AT |  | 7/26/2020 10:51 AM |  |
|  | I’m going to say this in terms of breasts because that’s where I am located currently, every Tuesday and every Thursday for new patients who are being chemotherapy but am not sure I need to find out this, I think they do health education on a daily basis but I am not sure, I have to find out. | | | | | | | | | | | | | | | |  |
|  |  | | | | | | | | | | | | | | | |  |
|  | **Nodes\\How informed consent or other information is communicated\illustrative diagrams** | | | | | | | | | | | | | | | |  |
|  | | **Document** | | | | | | | | | | | | | | |  |
|  | | | **Files\\IDI__ Doctor_ UCI_ 05** | | | | | | | | | | | | | |  |
|  |  |  | No |  |  |  | 0.0105 |  | 1 | |  | | | | | |  |
|  | | |  |  |  |  |  |  |  | |  | | | | | | |
|  | | | | | | | | | | | | 1 |  | AT |  | 7/26/2020 10:49 AM |  |
|  | In the clinic we don’t have images or anything like that but you as a clinician you try to draw illustrations, pictures, diagrams for them to understand but the counsellors I believe have pamphlets, they have books as well which have been even translated in to the local language at the same time. There is also health education which is done for the patients as well so there are some pictorials and flip charts. | | | | | | | | | | | | | | | |  |
|  |  | | | | | | | | | | | | | | | |  |
|  | **Nodes\\How informed consent or other information is communicated\Information leaflets** | | | | | | | | | | | | | | | |  |
|  | | **Document** | | | | | | | | | | | | | | |  |
|  | | | **Files\\IDI - _ Doctor_UCI_06** | | | | | | | | | | | | | |  |
|  |  |  | No |  |  |  | 0.0069 |  | 1 | |  | | | | | |  |
|  | | |  |  |  |  |  |  |  | |  | | | | | | |
|  | | | | | | | | | | | | 1 |  | AT |  | 7/24/2020 9:03 AM |  |
|  | Yes, there are charts that we use, in the screening room we have leaflets, I for one I use diagrams sometimes I draw a diagram of what is happening but in the screening clinic there are a lot of charts and public health materials. | | | | | | | | | | | | | | | |  |
|  |  | | | | | | | | | | | | | | | |  |
|  | | | | | | | | | | | | | | | | | |
| Reports\\Coding Summary By Code Report | | | | | | | | | | Page 70 of 117 | | | | | | | |
| 8/15/2023 8:10 AM | | | | | | | | | | | | | | | | | |
|  | | | **Aggregate** |  | **Classification** |  | **Coverage** |  | **Number Of Coding References** | |  | **Reference Number** |  | **Coded By Initials** |  | **Modified On** |  |
|  | **Nodes\\How informed consent or other information is communicated\Information materials such as cancer booklets** | | | | | | | | | | | | | | | |  |
|  | | **Document** | | | | | | | | | | | | | | |  |
|  | | | **Files\\IDI -Specialist palliative care_10** | | | | | | | | | | | | | |  |
|  |  |  | No |  |  |  | 0.0127 |  | 1 | |  | | | | | |  |
|  | | |  |  |  |  |  |  |  | |  | | | | | | |
|  | | | | | | | | | | | | 1 |  | AT |  | 7/24/2020 9:54 AM |  |
|  | I have seen materials that were developed by Uganda Cancer Institute, in the society and some other group of people. There are booklets that talk about cancer and information on cancer,a bit of information on treatment and they have been translated in some local languages they are in English and some local languages, they are for both care takers and patients yeah, they are there. Most of the time they are given after the person has been diagnosed and is on the ward. | | | | | | | | | | | | | | | |  |
|  |  | | | | | | | | | | | | | | | |  |
|  | | | **Files\\IDI- - Health Educator- UCI-09** | | | | | | | | | | | | | |  |
|  |  |  | No |  |  |  | 0.087 |  | 2 | |  | | | | | |  |
|  | | |  |  |  |  |  |  |  | |  | | | | | | |
|  | | | | | | | | | | | | 1 |  | AT |  | 7/25/2020 10:18 PM |  |
|  | Once I saw some booklets from American cancer society but I no longer see them, | | | | | | | | | | | | | | | |  |
|  |  | | | | | | | | | | | | | | | |  |
|  | | | | | | | | | | | | 2 |  | AT |  | 7/25/2020 10:19 PM |  |
|  | When we did assessment, we realized that most patients would want doctors or nurses to talk to them, we realise a second source of information was the audiovisual, rights like booklets and fax sheets are the option so as per that I was telling you that we are trying to come up with a way of trapping patients, we want every new patient to interface with a nurse to support then will meet after seeing the doctor now the nurse will interface the patient to give additional time for this patient to ask questions to reinforce the information. | | | | | | | | | | | | | | | |  |
|  |  | | | | | | | | | | | | | | | |  |
|  | **Nodes\\How informed consent or other information is communicated\Obtain both verbal and written consent for surgical procedures** | | | | | | | | | | | | | | | |  |
|  | | **Document** | | | | | | | | | | | | | | |  |
|  | | | **Files\\IDI - _ Doctor_UCI_06** | | | | | | | | | | | | | |  |
|  |  |  | No |  |  |  | 0.0158 |  | 2 | |  | | | | | |  |
|  | | |  |  |  |  |  |  |  | |  | | | | | | |
|  | | | | | | | | | | | | 1 |  | AT |  | 7/24/2020 8:55 AM |  |
|  | the yellow consent forms are ones being used at the first visit now the white consent form that is specific to surgery is used in theatre we don’t put it in the consent file because not every patient is going to access surgical services. | | | | | | | | | | | | | | | |  |
|  |  | | | | | | | | | | | | | | | |  |
|  | | | | | | | | | | | | 2 |  | AT |  | 7/24/2020 8:55 AM |  |
|  | Of late now we want to bring them in the clinic and then those who will be getting surgery we have the consent in the clinic and then by the time they go in the theatre they are already covered. later it’s put in the file, those that are going to benefit from surgery yes and its very important. | | | | | | | | | | | | | | | |  |
|  |  | | | | | | | | | | | | | | | |  |
|  | | | | | | | | | | | | | | | | | |
| Reports\\Coding Summary By Code Report | | | | | | | | | | Page 71 of 117 | | | | | | | |
| 8/15/2023 8:10 AM | | | | | | | | | | | | | | | | | |
|  | | | **Aggregate** |  | **Classification** |  | **Coverage** |  | **Number Of Coding References** | |  | **Reference Number** |  | **Coded By Initials** |  | **Modified On** |  |
|  | **Nodes\\How informed consent or other information is communicated\Patient not given chance to chose options** | | | | | | | | | | | | | | | |  |
|  | | **Document** | | | | | | | | | | | | | | |  |
|  | | | **Files\\IDI -Specialist palliative care_10** | | | | | | | | | | | | | |  |
|  |  |  | No |  |  |  | 0.0069 |  | 1 | |  | | | | | |  |
|  | | |  |  |  |  |  |  |  | |  | | | | | | |
|  | | | | | | | | | | | | 1 |  | AT |  | 7/24/2020 9:53 AM |  |
|  | But may be a brief on what the criteria intail, its not like giving a patient a chance to say that there options I don’t know am asking questions, it kind of giving information and expecting a positive response so I think that is a thing that is implied. | | | | | | | | | | | | | | | |  |
|  |  | | | | | | | | | | | | | | | |  |
|  | **Nodes\\How informed consent or other information is communicated\Patients asked to endorse signature** | | | | | | | | | | | | | | | |  |
|  | | **Document** | | | | | | | | | | | | | | |  |
|  | | | **Files\\IDI- - Health Educator- UCI-09** | | | | | | | | | | | | | |  |
|  |  |  | No |  |  |  | 0.0006 |  | 1 | |  | | | | | |  |
|  | | |  |  |  |  |  |  |  | |  | | | | | | |
|  | | | | | | | | | | | | 1 |  | AT |  | 7/25/2020 10:04 PM |  |
|  | They only ask patients that you sign here. | | | | | | | | | | | | | | | |  |
|  |  | | | | | | | | | | | | | | | |  |
|  | **Nodes\\How informed consent or other information is communicated\Relatives sign on behalf of patients who are very sick** | | | | | | | | | | | | | | | |  |
|  | | **Document** | | | | | | | | | | | | | | |  |
|  | | | **Files\\IDI -Specialist palliative care_10** | | | | | | | | | | | | | |  |
|  |  |  | No |  |  |  | 0.0137 |  | 2 | |  | | | | | |  |
|  | | |  |  |  |  |  |  |  | |  | | | | | | |
|  | | | | | | | | | | | | 1 |  | AT |  | 7/24/2020 10:16 AM |  |
|  | I think the person who opens the files is the one who signs its not even the patient, its not usually the patient especially if the patient is very sick sometimes it’s the relatives that sign for you. | | | | | | | | | | | | | | | |  |
|  |  | | | | | | | | | | | | | | | |  |
|  | | | | | | | | | | | | | | | | | |
| Reports\\Coding Summary By Code Report | | | | | | | | | | Page 72 of 117 | | | | | | | |
| 8/15/2023 8:10 AM | | | | | | | | | | | | | | | | | |
|  | | | **Aggregate** |  | **Classification** |  | **Coverage** |  | **Number Of Coding References** | |  | **Reference Number** |  | **Coded By Initials** |  | **Modified On** |  |
|  | | | | | | | | | | | | | | | | | |
|  | | | | | | | | | | | | 2 |  | AT |  | 7/24/2020 10:16 AM |  |
|  | I know that is the weakness of that thing that they always assume the relatives have to consent for you which is wrong so the people may be may need more training on the staff who consent to know that it is actually a personal consent not a relative unless patient is not conscious or has no mental capacity. | | | | | | | | | | | | | | | |  |
|  |  | | | | | | | | | | | | | | | |  |
|  | **Nodes\\How informed consent or other information is communicated\Senior doctors put results in writing then refers to counsellor to disclose** | | | | | | | | | | | | | | | |  |
|  | | **Document** | | | | | | | | | | | | | | |  |
|  | | | **Files\\IDI- - Health Educator- UCI-09** | | | | | | | | | | | | | |  |
|  |  |  | No |  |  |  | 0.0030 |  | 1 | |  | | | | | |  |
|  | | |  |  |  |  |  |  |  | |  | | | | | | |
|  | | | | | | | | | | | | 1 |  | AT |  | 7/25/2020 9:28 PM |  |
|  | disclosure is deep in the motive so sometimes the condition of the patient may not warrant disclosure at that point so the doctors may write in the file and pushes them to the nurses to disclose at an appropriate time. | | | | | | | | | | | | | | | |  |
|  |  | | | | | | | | | | | | | | | |  |
|  | **Nodes\\How informed consent or other information is communicated\Through counselling session** | | | | | | | | | | | | | | | |  |
|  | | **Document** | | | | | | | | | | | | | | |  |
|  | | | **Files\\IDI - - Doctor- UCI -08** | | | | | | | | | | | | | |  |
|  |  |  | No |  |  |  | 0.0279 |  | 1 | |  | | | | | |  |
|  | | |  |  |  |  |  |  |  | |  | | | | | | |
|  | | | | | | | | | | | | 1 |  | AT |  | 7/24/2020 7:47 AM |  |
|  | Now I must say that currently not all patients are given the same information some are given more information depending on how they present for the first time, I think some are talked too more and counselled those who are anxious, they are curious and they asking many questions they get more because of the line sometimes the doctors want to finish up and then sometimes those who are seen by the doctors are seen by the nurses to talk to them or the counsellor, those who will have an opportunity to talk to the counsellor they will get more information added from what the doctors say , the nurses say and the counsellors but those who will only have an opportunity to interact with the doctor they may not have so much information depending on whether that doctor has many lines of patients or not many. | | | | | | | | | | | | | | | |  |
|  |  | | | | | | | | | | | | | | | |  |
|  | | | | | | | | | | | | | | | | | |
|  | | | | | | | | | | | | | | | | | |
| Reports\\Coding Summary By Code Report | | | | | | | | | | Page 73 of 117 | | | | | | | |
| 8/15/2023 8:10 AM | | | | | | | | | | | | | | | | | |
|  | | | **Aggregate** |  | **Classification** |  | **Coverage** |  | **Number Of Coding References** | |  | **Reference Number** |  | **Coded By Initials** |  | **Modified On** |  |
|  | | | **Files\\IDI -Specialist palliative care_10** | | | | | | | | | | | | | |  |
|  |  |  | No |  |  |  | 0.0189 |  | 1 | |  | | | | | |  |
|  | | |  |  |  |  |  |  |  | |  | | | | | | |
|  | | | | | | | | | | | | 1 |  | AT |  | 7/24/2020 10:21 AM |  |
|  | They are very few by the way, most of the time they are giving information to patients about their diseases and supporting especially many of the times they divide the labor so that most information can be given by doctors or nurses then the complicated ones or where they feel someone is distressed or someone has refused the procedure or treatment or someone is not ready to hear it they can hand over to the counsellor or there is a complex family issue, those funny family dynamics, families don’t want what the patient wants that’s when they bring in the counsellor because of the numbers I think they practiced its worked out that way that they only send the most complex ones to the counsellors. | | | | | | | | | | | | | | | |  |
|  |  | | | | | | | | | | | | | | | |  |
|  | | | **Files\\IDI- - Health Educator- UCI-09** | | | | | | | | | | | | | |  |
|  |  |  | No |  |  |  | 0.0222 |  | 6 | |  | | | | | |  |
|  | | |  |  |  |  |  |  |  | |  | | | | | | |
|  | | | | | | | | | | | | 1 |  | AT |  | 7/24/2020 12:43 PM |  |
|  | Two there are others who turn out to be bad, we have counsellors, we get in touch with counsellors to help them go through after the doctor has given the diagnosis, the counsellor now helps them to cope with that and they can share with different sections. | | | | | | | | | | | | | | | |  |
|  |  | | | | | | | | | | | | | | | |  |
|  | | | | | | | | | | | | 2 |  | AT |  | 7/25/2020 9:35 PM |  |
|  | some of the patients are very anxious, you just talking to this person is now like hitting that person the last time so in most cases these ones who are very anxious are referred to the counsellors, the doctor will say no, you go to the counsellor first. The counsellor will support them to cope first, at that point they are helping you understand easily. | | | | | | | | | | | | | | | |  |
|  |  | | | | | | | | | | | | | | | |  |
|  | | | | | | | | | | | | 3 |  | AT |  | 7/25/2020 9:46 PM |  |
|  | You need to send this person to the counsellor first to prepare this person, you know we don’t have a standard way of disclosing cancer disease like in HIV where someone has to go through counselling first before testing and on giving the results you then prepare this patient. | | | | | | | | | | | | | | | |  |
|  |  | | | | | | | | | | | | | | | |  |
|  | | | | | | | | | | | | 4 |  | AT |  | 7/25/2020 10:01 PM |  |
|  | The signature is there on the consent form, every patient going for treatment must go through a counsellor and a counsellor must make them consent but we have two or three counsellors so there are others who cannot have a chance to go there and I have seen its only the patients in the breast | | | | | | | | | | | | | | | |  |
|  |  | | | | | | | | | | | | | | | |  |
|  | | | | | | | | | | | | 5 |  | AT |  | 7/25/2020 10:17 PM |  |
|  | Now at the level of Counsellors they have some tools I have seen, I have seen them having the flip charts which are a little bit talking about cancer but I do not see them with nurses always, I see them with counsellors. | | | | | | | | | | | | | | | |  |
|  |  | | | | | | | | | | | | | | | |  |
|  | | | | | | | | | | | | 6 |  | AT |  | 7/25/2020 10:17 PM |  |
|  | It talks about cancer actually explaining ok supports the counsellor to show the pictures signifying what cancer is and how it grows yes, I have seen those pictures at least. | | | | | | | | | | | | | | | |  |
|  |  | | | | | | | | | | | | | | | |  |
|  | | | **Files\\IDI__ Doctor_ UCI_ 05** | | | | | | | | | | | | | |  |
|  |  |  | No |  |  |  | 0.0252 |  | 3 | |  | | | | | |  |
|  | | |  |  |  |  |  |  |  | |  | | | | | | |
|  | | | | | | | | | | | | 1 |  | AT |  | 7/26/2020 10:34 AM |  |
|  | However, we also involve the counsellor as well in this because hat means there may be issues and from my little experience most of the time these social issues are really there but we give them all the information that they need and we prefer to use open ended questions so that they can ask more questions. Actually, I’m meant to speak less and the patient is meant to really ask and then that’s how I approach. | | | | | | | | | | | | | | | |  |
|  |  | | | | | | | | | | | | | | | |  |
|  | | | | | | | | | | | | 2 |  | AT |  | 7/26/2020 10:40 AM |  |
|  | Absolutely, we all react differently that’s why we work as a team, a doctor alone cannot manage that’s why we need the counsellor as well and they are very good reinforcement actually depending on how the patient. | | | | | | | | | | | | | | | |  |
|  |  | | | | | | | | | | | | | | | |  |
| Reports\\Coding Summary By Code Report | | | | | | | | | | Page 74 of 117 | | | | | | | |
| 8/15/2023 8:10 AM | | | | | | | | | | | | | | | | | |
|  | | | **Aggregate** |  | **Classification** |  | **Coverage** |  | **Number Of Coding References** | |  | **Reference Number** |  | **Coded By Initials** |  | **Modified On** |  |
|  | | | | | | | | | | | | | | | | | |
|  | | | | | | | | | | | | 3 |  | AT |  | 7/26/2020 10:41 AM |  |
|  | Depending on the environment if the patient becomes emotional you handle it as a professional. If the patient does not show any sign of emotions or no questions then that means this patient has not understood so I have to engage them again so you handle it on a patient by patient basis but still I am going to reinforce, the counsellor still has a very big role. | | | | | | | | | | | | | | | |  |
|  |  | | | | | | | | | | | | | | | |  |
|  | **Nodes\\How informed consent or other information is communicated\Verbal consent** | | | | | | | | | | | | | | | |  |
|  | | **Document** | | | | | | | | | | | | | | |  |
|  | | | **Files\\IDI -Specialist palliative care_10** | | | | | | | | | | | | | |  |
|  |  |  | No |  |  |  | 0.095 |  | 2 | |  | | | | | |  |
|  | | |  |  |  |  |  |  |  | |  | | | | | | |
|  | | | | | | | | | | | | 1 |  | AT |  | 7/24/2020 9:52 AM |  |
|  | Even myself I may not know which procedure, even myself I may not know which procedure is going to happen so what is asked from patients is verbal consent | | | | | | | | | | | | | | | |  |
|  |  | | | | | | | | | | | | | | | |  |
|  | | | | | | | | | | | | 2 |  | AT |  | 7/24/2020 9:52 AM |  |
|  | and also many times its probably more of one sided like we are going to do this procedure or may be am going to take out water from the lungs and that it and then the patient says ok but they believe. | | | | | | | | | | | | | | | |  |
|  |  | | | | | | | | | | | | | | | |  |
|  | | | **Files\\IDI__ Doctor_ UCI_ 05** | | | | | | | | | | | | | |  |
|  |  |  | No |  |  |  | 0.0027 |  | 1 | |  | | | | | |  |
|  | | |  |  |  |  |  |  |  | |  | | | | | | |
|  | | | | | | | | | | | | 1 |  | AT |  | 7/26/2020 10:47 AM |  |
|  | My role is simple to try to give you as much information as possible then you make an informed decision. | | | | | | | | | | | | | | | |  |
|  |  | | | | | | | | | | | | | | | |  |
|  | **Nodes\\Information health providers share with Patients with terminal cancer\Address patient expectations** | | | | | | | | | | | | | | | |  |
|  | | **Document** | | | | | | | | | | | | | | |  |
|  | | | **Files\\IDI - - Doctor- UCI -08** | | | | | | | | | | | | | |  |
|  |  |  | No |  |  |  | 0.0117 |  | 2 | |  | | | | | |  |
|  | | |  |  |  |  |  |  |  | |  | | | | | | |
|  | | | | | | | | | | | | 1 |  | AT |  | 7/24/2020 8:00 AM |  |
|  | When am communicating to a patient, I should answer the questions they have at that time some other questions that may come | | | | | | | | | | | | | | | |  |
|  |  | | | | | | | | | | | | | | | |  |
| Reports\\Coding Summary By Code Report | | | | | | | | | | Page 75 of 117 | | | | | | | |
| 8/15/2023 8:10 AM | | | | | | | | | | | | | | | | | |
|  | | | **Aggregate** |  | **Classification** |  | **Coverage** |  | **Number Of Coding References** | |  | **Reference Number** |  | **Coded By Initials** |  | **Modified On** |  |
|  | | | | | | | | | | | | | | | | | |
|  | | | | | | | | | | | | 2 |  | AT |  | 7/24/2020 8:00 AM |  |
|  | Not only the first time, whoever is handling the patient at anytime you should communicate to them, expectations and interests because there are those like we expect you to pay for this should all be communicated. | | | | | | | | | | | | | | | |  |
|  |  | | | | | | | | | | | | | | | |  |
|  | | | **Files\\IDI- - Health Educator- UCI-09** | | | | | | | | | | | | | |  |
|  |  |  | No |  |  |  | 0.0132 |  | 3 | |  | | | | | |  |
|  | | |  |  |  |  |  |  |  | |  | | | | | | |
|  | | | | | | | | | | | | 1 |  | AT |  | 7/24/2020 10:32 AM |  |
|  | Now what the institute is expecting from patients, it has been a little bit of top down approach to the lung cancer disease, very few times patients are involved in designing care. Very few times the institute has taken institute in knowing what patients want so at this point wat we fill is good for patients is what we give them. No patient sits in the annual planning meeting, many times services are not done to inform on the planning or the available literatures are never reviewed to inform the planning so knowing the expectations of clients is very important. | | | | | | | | | | | | | | | |  |
|  |  | | | | | | | | | | | | | | | |  |
|  | | | | | | | | | | | | 2 |  | AT |  | 7/24/2020 10:36 AM |  |
|  | Information need is what we expect that this patient needs the information so they know these patients expect medicine so medicine must be there. | | | | | | | | | | | | | | | |  |
|  |  | | | | | | | | | | | | | | | |  |
|  | | | | | | | | | | | | 3 |  | AT |  | 7/24/2020 12:34 PM |  |
|  | So this patient at this point has to know the diagnosis and is expected to know what will happen in subsequent visits to the institute. So, the first point is the doctor to tell the patient is to do that diagnosis so that is one. | | | | | | | | | | | | | | | |  |
|  |  | | | | | | | | | | | | | | | |  |
|  | | | **Files\\IDI_ Counselor_UCI_01** | | | | | | | | | | | | | |  |
|  |  |  | No |  |  |  | 0.0263 |  | 2 | |  | | | | | |  |
|  | | |  |  |  |  |  |  |  | |  | | | | | | |
|  | | | | | | | | | | | | 1 |  | AT |  | 7/25/2020 10:46 PM |  |
|  | Ok what they should expect, we tell them what is available they will get it because with this being a government institution everything is supposed to be given free unless it is a private patient so we tell them everything is available medication or anything is supposed to be free and if it is out of stock then they will be informed and they can buy and then we encourage them to consistently bid care without missing appointments which would be good for them to be able to improve their quality of life. | | | | | | | | | | | | | | | |  |
|  |  | | | | | | | | | | | | | | | |  |
|  | | | | | | | | | | | | 2 |  | AT |  | 7/25/2020 10:56 PM |  |
|  | They are not, I don’t think they are that is something important that I could be handled such that the patients get that information as they come here, the expectations and what health workers expect., | | | | | | | | | | | | | | | |  |
|  |  | | | | | | | | | | | | | | | |  |
|  | | | **Files\\IDI_ Nurse_UCI_07** | | | | | | | | | | | | | |  |
|  |  |  | No |  |  |  | 0.0065 |  | 1 | |  | | | | | |  |
|  | | |  |  |  |  |  |  |  | |  | | | | | | |
|  | | | | | | | | | | | | 1 |  | AT |  | 7/26/2020 8:23 AM |  |
|  | What I have observed is immediate. Usually when patients come in, you don’t know whom they are with, some patients walk in alone and when they move in alone, they tell you what to do. First of all, we ask them, do you know why you are here? | | | | | | | | | | | | | | | |  |
|  |  | | | | | | | | | | | | | | | |  |
|  | | | | | | | | | | | | | | | | | |
| Reports\\Coding Summary By Code Report | | | | | | | | | | Page 76 of 117 | | | | | | | |
| 8/15/2023 8:10 AM | | | | | | | | | | | | | | | | | |
|  | | | **Aggregate** |  | **Classification** |  | **Coverage** |  | **Number Of Coding References** | |  | **Reference Number** |  | **Coded By Initials** |  | **Modified On** |  |
|  | | | **Files\\IDI_ Nurse_UCI_02** | | | | | | | | | | | | | |  |
|  |  |  | No |  |  |  | 0.0050 |  | 1 | |  | | | | | |  |
|  | | |  |  |  |  |  |  |  | |  | | | | | | |
|  | | | | | | | | | | | | 1 |  | AT |  | 7/26/2020 9:15 AM |  |
|  | For sure that one I don’t tell them I really don’t think however, they also have their expectations from us but we don’t tell them what they expect from us. I think we miss it. | | | | | | | | | | | | | | | |  |
|  |  | | | | | | | | | | | | | | | |  |
|  | **Nodes\\Information health providers share with Patients with terminal cancer\Address psychological emotions** | | | | | | | | | | | | | | | |  |
|  | | **Document** | | | | | | | | | | | | | | |  |
|  | | | **Files\\IDI - _ Doctor_UCI_06** | | | | | | | | | | | | | |  |
|  |  |  | No |  |  |  | 0.0305 |  | 4 | |  | | | | | |  |
|  | | |  |  |  |  |  |  |  | |  | | | | | | |
|  | | | | | | | | | | | | 1 |  | AT |  | 7/24/2020 8:36 AM |  |
|  | There is even that aspect which is the mental health aspect of cancer, there are palliative care specialists who also come and talk to these patients but these are mostly on the wards, we don’t usually have them in the clinic. | | | | | | | | | | | | | | | |  |
|  |  | | | | | | | | | | | | | | | |  |
|  | | | | | | | | | | | | 2 |  | AT |  | 7/24/2020 8:38 AM |  |
|  | To inform them not to be worrying them. | | | | | | | | | | | | | | | |  |
|  |  | | | | | | | | | | | | | | | |  |
|  | | | | | | | | | | | | 3 |  | AT |  | 7/24/2020 9:08 AM |  |
|  | Because of the mental changes, the actual treatment itself is hard chemotherapy, radiotherapy surgery, their quality of life is reduced they are not able to do all that they would have wanted to do, they are less productive at work. So yes, the economic wellbeing changes and also let’s remember that while the cancer institute provides most of the drugs, there are times when there are certain medications that we need and the patient has got to buy them out of pocket so that also places an extra burden on them. | | | | | | | | | | | | | | | |  |
|  |  | | | | | | | | | | | | | | | |  |
|  | | | | | | | | | | | | 4 |  | AT |  | 7/24/2020 9:08 AM |  |
|  | The majority of the family it’s a hard effect knowing that their loved one has been diagnosed with cancer so the family is also affected. And you can see that quite a number of them are depressed about it so it negatively affects the family. | | | | | | | | | | | | | | | |  |
|  |  | | | | | | | | | | | | | | | |  |
|  | | | **Files\\IDI- - Health Educator- UCI-09** | | | | | | | | | | | | | |  |
|  |  |  | No |  |  |  | 0.0147 |  | 1 | |  | | | | | |  |
|  | | |  |  |  |  |  |  |  | |  | | | | | | |
|  | | | | | | | | | | | | 1 |  | AT |  | 7/24/2020 12:46 PM |  |
|  | and broadens that at this stage they discuss that it cannot be cured but the life of a patient, the quality of the blood a patient has can be improved when the patient is made happy, when pain has been relieved, when the patient has been cared for when generally the patient is loved, the love is going to be prolonged so that does not require the hospital, there are some points where you know this person is going to die so it is very tricky to communicate it just like that , that take this patient home he is going to die, no, you need to prepare the family members, prepare the patient to accept that at this point treatment is not going to help that within the days God has given me I can be happy not to feel the pain because he is going to die so that does not require hospital setting can be home and when the situation is bad he can move to nearest hospital like that so he doesn’t need to be at the cancer institute so that is a family conference discusses that and he is a senior doctor or leaves the discussion in the family conference. | | | | | | | | | | | | | | | |  |
|  |  |  |  |  |  |  |  |  |  |  |  |  |  |  |  |  |  |
|  |  | | | | | | | | | | | | | | | |  |
| Reports\\Coding Summary By Code Report | | | | | | | | | | Page 77 of 117 | | | | | | | |
| 8/15/2023 8:10 AM | | | | | | | | | | | | | | | | | |
|  | | | **Aggregate** |  | **Classification** |  | **Coverage** |  | **Number Of Coding References** | |  | **Reference Number** |  | **Coded By Initials** |  | **Modified On** |  |
|  | | | **Files\\IDI_ Counselor_UCI_01** | | | | | | | | | | | | | |  |
|  |  |  | No |  |  |  | 0.0178 |  | 2 | |  | | | | | |  |
|  | | |  |  |  |  |  |  |  | |  | | | | | | |
|  | | | | | | | | | | | | 1 |  | AT |  | 7/25/2020 10:50 PM |  |
|  | sometimes they will send to counsellors and will also continue from there even after junior doctors, some junior doctors also send them to us depending on the patient’s understanding. | | | | | | | | | | | | | | | |  |
|  |  | | | | | | | | | | | | | | | |  |
|  | | | | | | | | | | | | 2 |  | AT |  | 7/25/2020 10:51 PM |  |
|  | Usually the doctors will tell and of course if it is in advanced stage the patient or any one will not want to hear that, they become emotional or the patients the doctors will communicate what they have found out but now of course the patients will become emotional start crying the care takers. | | | | | | | | | | | | | | | |  |
|  |  | | | | | | | | | | | | | | | |  |
|  | | | **Files\\IDI_ Social worker_UCI_03** | | | | | | | | | | | | | |  |
|  |  |  | No |  |  |  | 0.0421 |  | 4 | |  | | | | | |  |
|  | | |  |  |  |  |  |  |  | |  | | | | | | |
|  | | | | | | | | | | | | 1 |  | AT |  | 7/26/2020 8:36 AM |  |
|  | that’s why I said the nurse would be the most appropriate because the nurse who work with the doctor at the time the doctor is explaining to the patient the nurse is there listening and in most cases first time. You know cancer information first time is scary. There was a patient when they told her about cancer, her mind shut off and she did not hear whatever was being said and such a patient will always bring them back and we ask the doctor to explain to the patient in our presence then when we go back we ask again, now do understand what the doctor has said when she says no then we go ahead and continue to explain further. | | | | | | | | | | | | | | | |  |
|  |  | | | | | | | | | | | | | | | |  |
|  | | | | | | | | | | | | 2 |  | AT |  | 7/26/2020 9:04 AM |  |
|  | I don’t think they know it, the one thing as social workers now we are trying to design a tool for psychosocial assessment and that now assesses all psychosocial risks and these risks covers a wider area of needs so perhaps that one covers a wider area when it comes to further assessment. | | | | | | | | | | | | | | | |  |
|  |  | | | | | | | | | | | | | | | |  |
|  | | | | | | | | | | | | 3 |  | AT |  | 7/26/2020 9:06 AM |  |
|  | perhaps it may but it will depend on how now the person is trying to meditate internally about who am I, what is my future, what is going to come out of all this, the disease , the treatment and all that but up to now she is still around but an old woman but very much encouraging her family members to be negative so that one I can of linked it to faith and inner meditation and saying let God take me right now because after all death is there for every on. | | | | | | | | | | | | | | | |  |
|  |  | | | | | | | | | | | | | | | |  |
|  | | | | | | | | | | | | 4 |  | AT |  | 7/26/2020 9:09 AM |  |
|  | They just put her on any treatment because I don’t know that one now is theory decided by the doctor. After discussion or they don’t discuss at all, as I have said when they have just seen your social economic status they ask where are from, how many children do you have, which work do you do, who is responsible for your care so that will now influence what they tell this patient where they need to stop. | | | | | | | | | | | | | | | |  |
|  |  | | | | | | | | | | | | | | | |  |
|  | | | **Files\\IDI_ Nurse_UCI_02** | | | | | | | | | | | | | |  |
|  |  |  | No |  |  |  | 0.0069 |  | 1 | |  | | | | | |  |
|  | | |  |  |  |  |  |  |  | |  | | | | | | |
|  | | | | | | | | | | | | 1 |  | AT |  | 7/26/2020 9:41 AM |  |
|  | They do but the way they pay attention is by sending to the responsible person, you come you tell me your social issue I will take you to a social worker, to a counsellor to solve it from there but when I am not directly so that is what they do. | | | | | | | | | | | | | | | |  |
|  |  | | | | | | | | | | | | | | | |  |
|  | | | | | | | | | | | | | | | | | |
| Reports\\Coding Summary By Code Report | | | | | | | | | | Page 78 of 117 | | | | | | | |
| 8/15/2023 8:10 AM | | | | | | | | | | | | | | | | | |
|  | | | **Aggregate** |  | **Classification** |  | **Coverage** |  | **Number Of Coding References** | |  | **Reference Number** |  | **Coded By Initials** |  | **Modified On** |  |
|  | | | **Files\\IDI_ _ Nurse_UCI_04** | | | | | | | | | | | | | |  |
|  |  |  | No |  |  |  | 0.0555 |  | 4 | |  | | | | | |  |
|  | | |  |  |  |  |  |  |  | |  | | | | | | |
|  | | | | | | | | | | | | 1 |  | AT |  | 7/26/2020 9:58 AM |  |
|  | Now that information usually the nurses partially try to give that information when they come then partially doctors also give that information that actually gets to cut across in between or depending on the stage that the patient comes in, the patient may need counselling so in the process of counselling the patient, when you take the patient to the counsellor the counsellor can also articulate so most of the clinical staffs that get across the patient they tend to give partial information but it cannot really be complete information, its not something that people mainly do actively. | | | | | | | | | | | | | | | |  |
|  |  | | | | | | | | | | | | | | | |  |
|  | | | | | | | | | | | | 2 |  | AT |  | 7/26/2020 10:10 AM |  |
|  | Someone can easily go in to shock if they have told you something. | | | | | | | | | | | | | | | |  |
|  |  | | | | | | | | | | | | | | | |  |
|  | | | | | | | | | | | | 3 |  | AT |  | 7/26/2020 10:21 AM |  |
|  | It greatly impacts on first of all these mothers who have always labelled, they have lost their marriages, because a person has got either cancer of the cervix in that it’s a stage four disease you cant so much satisfy your partner so the only thing she’s just looking at is that she is lying at the treatment in the process, the man is having to go and look out for satisfaction and in the long run they tend to lose their relationship also like sometimes for some men like for men they have gotten like the cancer of the prostate have so much been affected because the man is there is not able to actively satisfy the partner. It has been affecting in a way that ends up making the partner end up going so it breaks up marriages, families. | | | | | | | | | | | | | | | |  |
|  |  | | | | | | | | | | | | | | | |  |
|  | | | | | | | | | | | | 4 |  | AT |  | 7/26/2020 10:22 AM |  |
|  | Of course when you disclose about the state or the condition and sometimes when you tell this person what you’re having and they get to know okay now I have stage 4 the next thing that comes to the mind is that I think I am going to die and only thing he or she is worried about is that am just worried about my life so that aspect of telling the magnitude of your condition, the prognosis of your condition, | | | | | | | | | | | | | | | |  |
|  |  | | | | | | | | | | | | | | | |  |
|  | | | **Files\\IDI__ Doctor_ UCI_ 05** | | | | | | | | | | | | | |  |
|  |  |  | No |  |  |  | 0.0211 |  | 3 | |  | | | | | |  |
|  | | |  |  |  |  |  |  |  | |  | | | | | | |
|  | | | | | | | | | | | | 1 |  | AT |  | 7/26/2020 10:32 AM |  |
|  | The social bit because of the number that’s why we use the counsellors as well because we have so many patients, we try to divide some of these roles and the counsellors usually have a much better approach on how to find out the social situation of the patient. That doesn’t mean that I don’t find out but we do it as a team. Its not a one man’s job. | | | | | | | | | | | | | | | |  |
|  |  | | | | | | | | | | | | | | | |  |
|  | | | | | | | | | | | | 2 |  | AT |  | 7/26/2020 10:41 AM |  |
|  | Depending on the environment if the patient becomes emotional you handle it as a professional. If the patient does not show any sign of emotions or no questions then that means this patient has not understood so I have to engage them again so you handle it on a patient by patient basis but still I am going to reinforce, the counsellor still has a very big role. | | | | | | | | | | | | | | | |  |
|  |  | | | | | | | | | | | | | | | |  |
|  | | | | | | | | | | | | 3 |  | AT |  | 7/26/2020 10:57 AM |  |
|  | In cancer we work as a team so you always ask you psychological colleagues to intervene and review the patient. | | | | | | | | | | | | | | | |  |
|  |  | | | | | | | | | | | | | | | |  |
|  | | | | | | | | | | | | | | | | | |
|  | | | | | | | | | | | | | | | | | |
| Reports\\Coding Summary By Code Report | | | | | | | | | | Page 79 of 117 | | | | | | | |
| 8/15/2023 8:10 AM | | | | | | | | | | | | | | | | | |
|  | | | **Aggregate** |  | **Classification** |  | **Coverage** |  | **Number Of Coding References** | |  | **Reference Number** |  | **Coded By Initials** |  | **Modified On** |  |
|  | **Nodes\\Information health providers share with Patients with terminal cancer\Adherence to routine clinic appointments** | | | | | | | | | | | | | | | |  |
|  | | **Document** | | | | | | | | | | | | | | |  |
|  | | | **Files\\IDI - _ Doctor_UCI_06** | | | | | | | | | | | | | |  |
|  |  |  | No |  |  |  | 0.0121 |  | 1 | |  | | | | | |  |
|  | | |  |  |  |  |  |  |  | |  | | | | | | |
|  | | | | | | | | | | | | 1 |  | AT |  | 7/24/2020 8:37 AM |  |
|  | That one is normally given by the nurses who are involved in booking the subsequent visits who emphasis to the patients the importance of keeping their follow up days and coming for treatment. We really try to emphasize the importance of compliance of the treatment schedule if someone has to come in three weeks its three weeks, if they are supposed to go down for radiotherapy it should be radiotherapy. | | | | | | | | | | | | | | | |  |
|  |  | | | | | | | | | | | | | | | |  |
|  | | | **Files\\IDI_ Counselor_UCI_01** | | | | | | | | | | | | | |  |
|  |  |  | No |  |  |  | 0.0052 |  | 1 | |  | | | | | |  |
|  | | |  |  |  |  |  |  |  | |  | | | | | | |
|  | | | | | | | | | | | | 1 |  | AT |  | 7/25/2020 10:48 PM |  |
|  | And I will also tell them there is booking I tell them you come without appointment that subsequent disease you have to come on appointment. | | | | | | | | | | | | | | | |  |
|  |  | | | | | | | | | | | | | | | |  |
|  | | | **Files\\IDI_ Nurse_UCI_07** | | | | | | | | | | | | | |  |
|  |  |  | No |  |  |  | 0.0178 |  | 3 | |  | | | | | |  |
|  | | |  |  |  |  |  |  |  | |  | | | | | | |
|  | | | | | | | | | | | | 1 |  | AT |  | 7/26/2020 8:18 AM |  |
|  | the counsellor is supposed to give that information, after the counsellor giving that information, the nurse in the treatment room is supposed to emphasize because the nurses give return dates, they give dates of appointment to these patients | | | | | | | | | | | | | | | |  |
|  |  | | | | | | | | | | | | | | | |  |
|  | | | | | | | | | | | | 2 |  | AT |  | 7/26/2020 8:19 AM |  |
|  | Yes. After that as a nurse who is in the treatment room you’re going to give a date of appointment because its your role to give a date so as your giving your date of appointment | | | | | | | | | | | | | | | |  |
|  |  | | | | | | | | | | | | | | | |  |
|  | | | | | | | | | | | | 3 |  | AT |  | 7/26/2020 8:21 AM |  |
|  | We tell them where they should register for their files to be reviewed for the doctor on the next appointment so it is usually the nurse at the booking table and in the treatment room that explains the patients processes to them. | | | | | | | | | | | | | | | |  |
|  |  | | | | | | | | | | | | | | | |  |
|  | | | | | | | | | | | | | | | | | |
|  | | | | | | | | | | | | | | | | | |
|  | | | | | | | | | | | | | | | | | |
| Reports\\Coding Summary By Code Report | | | | | | | | | | Page 80 of 117 | | | | | | | |
| 8/15/2023 8:10 AM | | | | | | | | | | | | | | | | | |
|  | | | **Aggregate** |  | **Classification** |  | **Coverage** |  | **Number Of Coding References** | |  | **Reference Number** |  | **Coded By Initials** |  | **Modified On** |  |
|  | **Nodes\\Information health providers share with Patients with terminal cancer\Adherence to treatment** | | | | | | | | | | | | | | | |  |
|  | | **Document** | | | | | | | | | | | | | | |  |
|  | | | **Files\\IDI_ Social worker_UCI_03** | | | | | | | | | | | | | |  |
|  |  |  | No |  |  |  | 0.096 |  | 1 | |  | | | | | |  |
|  | | |  |  |  |  |  |  |  | |  | | | | | | |
|  | | | | | | | | | | | | 1 |  | AT |  | 7/26/2020 8:37 AM |  |
|  | It is also the nurse because it is the nurse to try and explain to the patient how it is important to adhere to the treatment once they have started it and then it is important to ensure that they give us feedback when they get challenges when they cannot come or when they cannot get the drug or when they feel there is something that is going to affect their schedule or when they don’t have a care giver. | | | | | | | | | | | | | | | |  |
|  |  | | | | | | | | | | | | | | | |  |
|  | **Nodes\\Information health providers share with Patients with terminal cancer\Asses prior knowledge about the disease** | | | | | | | | | | | | | | | |  |
|  | | **Document** | | | | | | | | | | | | | | |  |
|  | | | **Files\\IDI -Specialist palliative care_10** | | | | | | | | | | | | | |  |
|  |  |  | No |  |  |  | 0.088 |  | 1 | |  | | | | | |  |
|  | | |  |  |  |  |  |  |  | |  | | | | | | |
|  | | | | | | | | | | | | 1 |  | AT |  | 7/24/2020 9:30 AM |  |
|  | The first we encounter we want to find out how much that person knows about the condition they so that will guide our conversation and the information we give subsequently, how much do they know about the condition and then what are their expectations from care, once we know that then we can know how to communicate further. | | | | | | | | | | | | | | | |  |
|  |  | | | | | | | | | | | | | | | |  |
|  | | | **Files\\IDI__ Doctor_ UCI_ 05** | | | | | | | | | | | | | |  |
|  |  |  | No |  |  |  | 0.0168 |  | 1 | |  | | | | | |  |
|  | | |  |  |  |  |  |  |  | |  | | | | | | |
|  | | | | | | | | | | | | 1 |  | AT |  | 7/26/2020 10:29 AM |  |
|  | I am going to answer that question in a very simple way and I am going to relate it to myself because I see patients here and most of the patients, I see are actually terminal patients I usually start with a question which says what have you been told so far about your disease? That’s what I usually start with the second question is do you know where you are because to me that’s the question about dialogue because some patients just think they are in Mulago yet Mulago has been really separate in to different entities so if a patient knows that is at the cancer institute I think that’s a good start because I know then your diagnosis is related to cancer. | | | | | | | | | | | | | | | |  |
|  |  | | | | | | | | | | | | | | | |  |
|  | | | | | | | | | | | | | | | | | |
|  | | | | | | | | | | | | | | | | | |
| Reports\\Coding Summary By Code Report | | | | | | | | | | Page 81 of 117 | | | | | | | |
| 8/15/2023 8:10 AM | | | | | | | | | | | | | | | | | |
|  | | | **Aggregate** |  | **Classification** |  | **Coverage** |  | **Number Of Coding References** | |  | **Reference Number** |  | **Coded By Initials** |  | **Modified On** |  |
|  | **Nodes\\Information health providers share with Patients with terminal cancer\Basic information about Cancer disease( benefit of Cancer treatment, health tips regarding cancer)** | | | | | | | | | | | | | | | |  |
|  | | **Document** | | | | | | | | | | | | | | |  |
|  | | | **Files\\IDI - _ Doctor_UCI_06** | | | | | | | | | | | | | |  |
|  |  |  | No |  |  |  | 0.0360 |  | 5 | |  | | | | | |  |
|  | | |  |  |  |  |  |  |  | |  | | | | | | |
|  | | | | | | | | | | | | 1 |  | AT |  | 7/24/2020 9:24 AM |  |
|  | Advanced and terminal are used in touching edges, I really cant go in to the details of particular cancers but generally different cancers have got different staging but most cancers really in the gynecology, they are four stages, stage 3 and 4 are considered to be advanced cancer where as stage 1 and stage 2 that early cancer we are able to treat, we can treat it, the lower the number that better. | | | | | | | | | | | | | | | |  |
|  |  | | | | | | | | | | | | | | | |  |
|  | | | | | | | | | | | | 2 |  | AT |  | 7/24/2020 9:24 AM |  |
|  | Third stage is an advanced stage for most cancers its advanced, it has gone beyond the primary area. | | | | | | | | | | | | | | | |  |
|  |  | | | | | | | | | | | | | | | |  |
|  | | | | | | | | | | | | 3 |  | AT |  | 7/24/2020 9:25 AM |  |
|  | For the gynae its advanced, we will still give you some treatment but we are not curing it no, we are controlling symptoms, giving you a good quality of life. The cancer actually may seem to have disappeared but it will come back that one we are very sure it will come back. | | | | | | | | | | | | | | | |  |
|  |  | | | | | | | | | | | | | | | |  |
|  | | | | | | | | | | | | 4 |  | AT |  | 7/24/2020 9:25 AM |  |
|  | yeah, depending on the investigations CT scan may come and show there is a regioning in the lung that means now the stage has gone up to four or something on the spine or something on the liver that is four it changes or the kidneys. | | | | | | | | | | | | | | | |  |
|  |  | | | | | | | | | | | | | | | |  |
|  | | | | | | | | | | | | 5 |  | AT |  | 7/24/2020 9:25 AM |  |
|  | But sometimes the staging also depends on the clinicians, one clinician may have staged it at a lower stage and then another one comes in more experienced and stages it higher that also happens. | | | | | | | | | | | | | | | |  |
|  |  | | | | | | | | | | | | | | | |  |
|  | | | **Files\\IDI- - Health Educator- UCI-09** | | | | | | | | | | | | | |  |
|  |  |  | No |  |  |  | 0.0155 |  | 3 | |  | | | | | |  |
|  | | |  |  |  |  |  |  |  | |  | | | | | | |
|  | | | | | | | | | | | | 1 |  | AT |  | 7/24/2020 10:24 AM |  |
|  | The first part is generally, a cancer patient is eager to know is the type of cancer and wants to know that now I have cancer, what will happen and in a nut shell whoever comes here comes with a feeling of seeking for a possibility of cure, can this cancer be cured, is there first reason why they come here so in most cases when a new patient comes, the doctor interfaces with the patient that’s when they clerk patients. In the clerking room the doctor is ideally going to tell this patient the diagnosis, that is the type of cancer and explain to this patient what that means. | | | | | | | | | | | | | | | |  |
|  |  | | | | | | | | | | | | | | | |  |
|  | | | | | | | | | | | | 2 |  | AT |  | 7/24/2020 12:42 PM |  |
|  | In most cases you talk to the relatives the importance of making the patient know her condition, the importance of making the patient able to embrace the cancer diagnosis so when the family members or when some of them understand they will even be surprised that when you tell this patient the diagnosis, the patient responds positively so it is sometimes the fear of the relatives. | | | | | | | | | | | | | | | |  |
|  |  | | | | | | | | | | | | | | | |  |
|  | | | | | | | | | | | | 3 |  | AT |  | 7/25/2020 9:26 PM |  |
|  | The doctors know, I came for treatment now you are telling me my disease cannot be treated, which kind of doctor are you? Have failed to treat me? | | | | | | | | | | | | | | | |  |
|  |  | | | | | | | | | | | | | | | |  |
|  | | | | | | | | | | | | | | | | | |
| Reports\\Coding Summary By Code Report | | | | | | | | | | Page 82 of 117 | | | | | | | |
| 8/15/2023 8:10 AM | | | | | | | | | | | | | | | | | |
|  | | | **Aggregate** |  | **Classification** |  | **Coverage** |  | **Number Of Coding References** | |  | **Reference Number** |  | **Coded By Initials** |  | **Modified On** |  |
|  | | | **Files\\IDI_ Counselor_UCI_01** | | | | | | | | | | | | | |  |
|  |  |  | No |  |  |  | 0.0791 |  | 4 | |  | | | | | |  |
|  | | |  |  |  |  |  |  |  | |  | | | | | | |
|  | | | | | | | | | | | | 1 |  | AT |  | 7/25/2020 10:45 PM |  |
|  | At the first visit, these days the health educators meet them and they give them the basics about cancer and try to answer questions they have come with or anything they feel they want to know about cancer, they will always ask and this health educator tries to answer maybe if there is anything that is beyond him then he can refer; in my case if I am the one the question that belongs to the doctor I will encourage them that for that question ask you doctor when you go to see the doctor | | | | | | | | | | | | | | | |  |
|  |  | | | | | | | | | | | | | | | |  |
|  | | | | | | | | | | | | 2 |  | AT |  | 7/25/2020 10:46 PM |  |
|  | Especially questions that are related to the disease it self because me as a counsellor I will only handle psychological issues when it is about the disease I do not want to interfere, I want them to hear from the horse’s mouth if he is going to see the doctor it is the doctor who has read about cancer and knows everything so sometimes I give them a brief answer and then I let them go and get more information. | | | | | | | | | | | | | | | |  |
|  |  | | | | | | | | | | | | | | | |  |
|  | | | | | | | | | | | | 3 |  | AT |  | 7/25/2020 10:51 PM |  |
|  | Now we sit and discuss, we make sure because it is not good for patients to be on care without understanding why they are giving this and that the patients should know why he is here and whatever he is doing or the benefits so it is important for the patient to know. We sit with them tell them that, the importance of the patients knowing then finally they will allow us disclose to the patient but in most cases especially with the adult patients they will have even suspected because they don’t just come here first time they screen them then they tell them they have cancer, they will have gone to so many health centers, they will have passed through other hospitals by the time they reach here they will have already known something that some serious disease is in their body. So sometimes we don’t find so much hustle when we are dealing with patients in disclosing such then when the care takers where denying the patient to know they say yes I know you know. | | | | | | | | | | | | | | | |  |
|  |  |  |  |  |  |  |  |  |  |  |  |  |  |  |  |  |  |
|  |  | | | | | | | | | | | | | | | |  |
|  | | | | | | | | | | | | 4 |  | AT |  | 7/25/2020 10:55 PM |  |
|  | I think the qualified ones, everyone where ever the patient moves so long as the qualified staff is supposed to give information. Giving health tips on the ongoing process at each point of every health worker, the patient is supposed to get some health tip. | | | | | | | | | | | | | | | |  |
|  |  | | | | | | | | | | | | | | | |  |
|  | | | **Files\\IDI_ Nurse_UCI_07** | | | | | | | | | | | | | |  |
|  |  |  | No |  |  |  | 0.0063 |  | 1 | |  | | | | | |  |
|  | | |  |  |  |  |  |  |  | |  | | | | | | |
|  | | | | | | | | | | | | 1 |  | AT |  | 7/26/2020 8:21 AM |  |
|  | To me I usually get these patients in the consultant’s room, so I am assuming the person who clerked these patients has given them information about their disease or their prognosis, however when they come to the consultant’s room… | | | | | | | | | | | | | | | |  |
|  |  | | | | | | | | | | | | | | | |  |
|  | | | **Files\\IDI_ Social worker_UCI_03** | | | | | | | | | | | | | |  |
|  |  |  | No |  |  |  | 0.0070 |  | 1 | |  | | | | | |  |
|  | | |  |  |  |  |  |  |  | |  | | | | | | |
|  | | | | | | | | | | | | 1 |  | AT |  | 7/26/2020 8:32 AM |  |
|  | Then when we ask them because in our consent form we also have a section where we ask them if they know their cancer and they know the schedule, their treatment plan so a number of them nearly 80% they come when they only know that they have the cancer but they don’t know anything about that cancer. | | | | | | | | | | | | | | | |  |
|  |  | | | | | | | | | | | | | | | |  |
|  | | | **Files\\IDI_ Nurse_UCI_02** | | | | | | | | | | | | | |  |
|  |  |  | No |  |  |  | 0.0106 |  | 1 | |  | | | | | |  |
|  | | |  |  |  |  |  |  |  | |  | | | | | | |
|  | | | | | | | | | | | | 1 |  | AT |  | 7/26/2020 9:14 AM |  |
|  | It varies you might find someone will come then they will tell them the type of cancer they have and some will go deep to explain that type of cancer some will tell them you have breast cancer or cancer of the cervix because they first see a doctor who clerks them, what the pain when did you get the disease and ask them that information then they will give them investigations. | | | | | | | | | | | | | | | |  |
|  |  | | | | | | | | | | | | | | | |  |
| Reports\\Coding Summary By Code Report | | | | | | | | | | Page 83 of 117 | | | | | | | |
| 8/15/2023 8:10 AM | | | | | | | | | | | | | | | | | |
|  | | | **Aggregate** |  | **Classification** |  | **Coverage** |  | **Number Of Coding References** | |  | **Reference Number** |  | **Coded By Initials** |  | **Modified On** |  |
|  | | | **Files\\IDI_ _ Nurse_UCI_04** | | | | | | | | | | | | | |  |
|  |  |  | No |  |  |  | 0.087 |  | 1 | |  | | | | | |  |
|  | | |  |  |  |  |  |  |  | |  | | | | | | |
|  | | | | | | | | | | | | 1 |  | AT |  | 7/26/2020 9:57 AM |  |
|  | Before clerking, there is not much of the information that they get they are just waiting for the clerking period now its after clerking or in the process of the clerking the doctor may try to highlight a little bit but its after clerking usually when this information is brought up. | | | | | | | | | | | | | | | |  |
|  |  | | | | | | | | | | | | | | | |  |
|  | | | **Files\\IDI__ Doctor_ UCI_ 05** | | | | | | | | | | | | | |  |
|  |  |  | No |  |  |  | 0.0714 |  | 7 | |  | | | | | |  |
|  | | |  |  |  |  |  |  |  | |  | | | | | | |
|  | | | | | | | | | | | | 1 |  | AT |  | 7/26/2020 11:03 AM |  |
|  | No, the systems may not be the same, when you’re speaking about solid tumors we speak about what we call the TNM, T standing for Tumor meaning that you want to know the dimension of the tumor, is it confined, has it broken through whatever consignment its in. N being the nodes the lymphnodes has the cancer spread to the lymphnodes and the M being Metastasis meaning has it gone to the brain, has it done to the lungs, to the liver and bones or whatever it is so that’s for all the solid tumors we follow the TNM. Now for the Lymphomas | | | | | | | | | | | | | | | |  |
|  |  | | | | | | | | | | | | | | | |  |
|  | | | | | | | | | | | | 2 |  | AT |  | 7/26/2020 11:03 AM |  |
|  | It is really very detailed depending on the specific cancer you’re talking about whether it is the eye, skin, liver or all of them, it is a big thing. For all of them you have to do an assessment, you’ve to do staging work so that you know what the T, N, M is, that’s why we ask for a CT scan or MRI or whatever it is because it gives me information or what I will rely on to tell that oh please you’re stage 1, 2,3 or 4. Now if those things are done its my mandate to tell the patient that you’re this stage however like we were speaking about economics, some of these things you have to pay for them because we may not have them here. Now if you’ve not done then, I can’t adequately tell you what your stage is so that can be a challenge. | | | | | | | | | | | | | | | |  |
|  |  | | | | | | | | | | | | | | | |  |
|  | | | | | | | | | | | | 3 |  | AT |  | 7/26/2020 11:04 AM |  |
|  | Yes, they are there Lymphomas especially because they are compressing, they are first growing so you can actually stage as you treat the patient. Yes, one specific cancer is Burkitts lymphoma it responds excellently well to chemotherapy and its treated by chemotherapy alone so you may not have all the staging work and start in the mini time and the patient gets better as you work up, it is one thing you can always stage. | | | | | | | | | | | | | | | |  |
|  |  | | | | | | | | | | | | | | | |  |
|  | | | | | | | | | | | | 4 |  | AT |  | 7/26/2020 11:04 AM |  |
|  | I mean you can be stage 1 and you become stage 4 it happens but I think when I’m evaluating the patient from my history to physical exam and investigations that I have, I can comprehensively tell whether the patient is stage 1 or stage 4. | | | | | | | | | | | | | | | |  |
|  |  | | | | | | | | | | | | | | | |  |
|  | | | | | | | | | | | | 5 |  | AT |  | 7/26/2020 11:04 AM |  |
|  | Its still 1,2,3,4, its still the same thing the only thing is that you may have A, B, C, X, S,E those things stand for other things now. It means yes your stage 4 but you have B symptoms or you stage 4 but you don’t have B symptoms. | | | | | | | | | | | | | | | |  |
|  |  | | | | | | | | | | | | | | | |  |
|  | | | | | | | | | | | | 6 |  | AT |  | 7/26/2020 11:04 AM |  |
|  | There are other things that come about but the stage is still the same your either 2 with a B or 2 with an A your still stage 2 but it is because you don’t have something or you have something but its still stage 1 up to stage 4. | | | | | | | | | | | | | | | |  |
|  |  | | | | | | | | | | | | | | | |  |
|  | | | | | | | | | | | | 7 |  | AT |  | 7/26/2020 11:05 AM |  |
|  | I think that can be confusing for some people but when we say a disease is advanced we mean stage 3 and stage4 anything in 3 or 4 is advanced and what stage 3 means you have what we call local regional involvement meaning you have lemphnodes involved and you have your breast involved because stage 1 and stage 2 your disease is localized so stage 4 the disease has left and has gone even in other places. | | | | | | | | | | | | | | | |  |
|  |  | | | | | | | | | | | | | | | |  |
|  | | | | | | | | | | | | | | | | | |
| Reports\\Coding Summary By Code Report | | | | | | | | | | Page 84 of 117 | | | | | | | |
| 8/15/2023 8:10 AM | | | | | | | | | | | | | | | | | |
|  | | | **Aggregate** |  | **Classification** |  | **Coverage** |  | **Number Of Coding References** | |  | **Reference Number** |  | **Coded By Initials** |  | **Modified On** |  |
|  | **Nodes\\Information health providers share with Patients with terminal cancer\Communicate about Chemotherapy** | | | | | | | | | | | | | | | |  |
|  | | **Document** | | | | | | | | | | | | | | |  |
|  | | | **Files\\IDI - - Doctor- UCI -08** | | | | | | | | | | | | | |  |
|  |  |  | No |  |  |  | 0.0535 |  | 4 | |  | | | | | |  |
|  | | |  |  |  |  |  |  |  | |  | | | | | | |
|  | | | | | | | | | | | | 1 |  | AT |  | 7/24/2020 7:53 AM |  |
|  | I wouldn’t say it is just the senior doctors only I would say it is the senior doctor who is usually supposed to write the chemo, communicates to the patient. | | | | | | | | | | | | | | | |  |
|  |  | | | | | | | | | | | | | | | |  |
|  | | | | | | | | | | | | 2 |  | AT |  | 7/24/2020 7:54 AM |  |
|  | that is supposed to be the work of the doctor who is writing the chemo the first time. Or the team which is seated to write the chemo at that time but sometimes like in OPD because of the line of patients at times that information is given to the nurse and then the nurse who works with the doctor is the one who gives the information to the patient, that is in an ideal situation but there are also instances which of course are not right ones where by patients are written medicines and told you go to the pharmacy and get that and then sometimes they come back to you may be you’re lower than the one wrote the medicine underlined that doctor you just wrote for me this but I have not understood so those happen. | | | | | | | | | | | | | | | |  |
|  |  | | | | | | | | | | | | | | | |  |
|  | | | | | | | | | | | | 3 |  | AT |  | 7/24/2020 7:55 AM |  |
|  | They do, sometimes some clinics they have a doctor, a nurse and a counsellor near by so sometimes there are some people who refuse to get the chemo so the information goes up to the counsellors. | | | | | | | | | | | | | | | |  |
|  |  | | | | | | | | | | | | | | | |  |
|  | | | | | | | | | | | | 4 |  | AT |  | 7/24/2020 8:01 AM |  |
|  | Now the disclosure to the patient or the attendant is done for the first time the patient has come and they need to talk about a cancer drug chem was investigated now has come back with the investigation results and is going to see to start chemo, usually that information is given to the patient or to the attendant. Remember there are some patients that the doctor may or the doctor may find that he or she is not able to handle all the information so they may give in part. | | | | | | | | | | | | | | | |  |
|  |  | | | | | | | | | | | | | | | |  |
|  | | | **Files\\IDI - _ Doctor_UCI_06** | | | | | | | | | | | | | |  |
|  |  |  | No |  |  |  | 0.089 |  | 1 | |  | | | | | |  |
|  | | |  |  |  |  |  |  |  | |  | | | | | | |
|  | | | | | | | | | | | | 1 |  | AT |  | 7/24/2020 8:34 AM |  |
|  | Most times we are going to give radio therapy plus chemotherapy and we do tell them that they are going to get some side effects of the treatment. That the treatment is not like Panadol or amoxicillin but is much stronger but we are very clear that we are not curing but improving symptom management. | | | | | | | | | | | | | | | |  |
|  |  | | | | | | | | | | | | | | | |  |
|  | | | **Files\\IDI -Specialist palliative care_10** | | | | | | | | | | | | | |  |
|  |  |  | No |  |  |  | 0.0172 |  | 2 | |  | | | | | |  |
|  | | |  |  |  |  |  |  |  | |  | | | | | | |
|  | | | | | | | | | | | | 1 |  | AT |  | 7/24/2020 9:33 AM |  |
|  | Most of the planned treatment especially if it is oncology treatment its chemotherapy it’s the oncologists who will communicate it or the doctors under oncology team and then of course when the palliative care team come in to talk about its treatment like supportive treatment and symptom control. | | | | | | | | | | | | | | | |  |
|  |  | | | | | | | | | | | | | | | |  |
|  | | | | | | | | | | | | 2 |  | AT |  | 7/24/2020 9:33 AM |  |
|  | That information is given by both doctors and nurses and many nurse who administer chemotherapy will be very well versed with information on side effects and will give information about that to patients, I think because the nurses are even more available on the ward when they give more information about that and the doctors in out patients. | | | | | | | | | | | | | | | |  |
|  |  | | | | | | | | | | | | | | | |  |
| Reports\\Coding Summary By Code Report | | | | | | | | | | Page 85 of 117 | | | | | | | |
| 8/15/2023 8:10 AM | | | | | | | | | | | | | | | | | |
|  | | | **Aggregate** |  | **Classification** |  | **Coverage** |  | **Number Of Coding References** | |  | **Reference Number** |  | **Coded By Initials** |  | **Modified On** |  |
|  | | | **Files\\IDI- - Health Educator- UCI-09** | | | | | | | | | | | | | |  |
|  |  |  | No |  |  |  | 0.0031 |  | 1 | |  | | | | | |  |
|  | | |  |  |  |  |  |  |  | |  | | | | | | |
|  | | | | | | | | | | | | 1 |  | AT |  | 7/24/2020 12:35 PM |  |
|  | So, this senior doctor will say now you will benefit from chemotherapy or radiotherapy now at this point the doctor is supposed to prescribe treatment, reviews the patient with results of the tests then prescribes treatment. | | | | | | | | | | | | | | | |  |
|  |  | | | | | | | | | | | | | | | |  |
|  | | | **Files\\IDI_ Counselor_UCI_01** | | | | | | | | | | | | | |  |
|  |  |  | No |  |  |  | 0.0263 |  | 2 | |  | | | | | |  |
|  | | |  |  |  |  |  |  |  | |  | | | | | | |
|  | | | | | | | | | | | | 1 |  | AT |  | 7/25/2020 10:47 PM |  |
|  | so that they can base on them to decide on which intervention to give because we have many interventions, three of them actually chemotherapy, surgery and radio therapy so I always tell them today the doctor is going to treat any other concern but is not going to give you cancer treatment. Cancer treatment will be given when those other supporting tests have been done and they will help the doctor to make a decision because they help the doctor to know how far the disease has gone and how strong the patient’s body is. | | | | | | | | | | | | | | | |  |
|  |  | | | | | | | | | | | | | | | |  |
|  | | | | | | | | | | | | 2 |  | AT |  | 7/25/2020 10:54 PM |  |
|  | Senior doctor because he is the one who prescribes then maybe even those nurses in the infusion room after giving the chemo I don’t know if they also talk to them but for treatment there, | | | | | | | | | | | | | | | |  |
|  |  | | | | | | | | | | | | | | | |  |
|  | | | **Files\\IDI_ Nurse_UCI_07** | | | | | | | | | | | | | |  |
|  |  |  | No |  |  |  | 0.019 |  | 1 | |  | | | | | |  |
|  | | |  |  |  |  |  |  |  | |  | | | | | | |
|  | | | | | | | | | | | | 1 |  | AT |  | 7/26/2020 8:18 AM |  |
|  | as to when to receive another dose or another cycle of chemotherapy. | | | | | | | | | | | | | | | |  |
|  |  | | | | | | | | | | | | | | | |  |
|  | | | **Files\\IDI_ Social worker_UCI_03** | | | | | | | | | | | | | |  |
|  |  |  | No |  |  |  | 0.0493 |  | 5 | |  | | | | | |  |
|  | | |  |  |  |  |  |  |  | |  | | | | | | |
|  | | | | | | | | | | | | 1 |  | AT |  | 7/26/2020 8:34 AM |  |
|  | No, what they say they will say that they told me I should come tomorrow, I will be starting treatment so one they will not be knowing whether that drug is for buying or not for buying so when they come back after the following day that is when they again go back to us and start saying I went to the pharmacy they gave me this list of drugs that they are going to put on me but they said number one and two are not there then now it is us who come back to the pharmacies and start asking them, this patient you’ve given them this drug that is not there | | | | | | | | | | | | | | | |  |
|  |  | | | | | | | | | | | | | | | |  |
|  | | | | | | | | | | | | 2 |  | AT |  | 7/26/2020 8:34 AM |  |
|  | First of all they don’t know the name of the drug, secondly they don’t even know the price so then we help them to find out the price and then we now also help them to try fairer pharmacies in regard to what they have explained sometimes they don’t have money or they have very little money then now when we call that pharmacy, that pharmacy might tell us the price of the drug then now the patient might have little money then we get the medical form then also there is a patient who says when the family has money they send it to the patient’s mobile phone then they go and withdraw buy the drug then bring it to the pharmacy. | | | | | | | | | | | | | | | |  |
|  |  | | | | | | | | | | | | | | | |  |
|  | | | | | | | | | | | | 3 |  | AT |  | 7/26/2020 8:34 AM |  |
|  | For us when we ask them what did they tell you , they just tell they told me tomorrow am starting the drug, you ask what drug, then they say chemotherapy but now the specific details about chemo is what they don’t know, most of them don’t know and that is why we take their prescription and come back to the pharmacy or sometimes go and carry their file where ever it is. | | | | | | | | | | | | | | | |  |
|  |  | | | | | | | | | | | | | | | |  |
|  | | | | | | | | | | | | 4 |  | AT |  | 7/26/2020 8:35 AM |  |
|  | For us we usually start from the doctor’s perceptive, we take the file or we come with the patient and their prescription then you say doctor this patient is saying ABCD, can you help the patient understand what drug the patient needs. | | | | | | | | | | | | | | | |  |
|  |  | | | | | | | | | | | | | | | |  |
| Reports\\Coding Summary By Code Report | | | | | | | | | | Page 86 of 117 | | | | | | | |
| 8/15/2023 8:10 AM | | | | | | | | | | | | | | | | | |
|  | | | **Aggregate** |  | **Classification** |  | **Coverage** |  | **Number Of Coding References** | |  | **Reference Number** |  | **Coded By Initials** |  | **Modified On** |  |
|  | | | | | | | | | | | | | | | | | |
|  | | | | | | | | | | | | 5 |  | AT |  | 7/26/2020 8:35 AM |  |
|  | Yeah, we usually consult doctors, nurses. If the patient has already been receiving the drug maybe like the first time the second time and now it is the third time that the drug is not there and the patient now do not know how to go about the drug that is missing so that they continue then they come to us. | | | | | | | | | | | | | | | |  |
|  |  | | | | | | | | | | | | | | | |  |
|  | | | **Files\\IDI_ Nurse_UCI_02** | | | | | | | | | | | | | |  |
|  |  |  | No |  |  |  | 0.041 |  | 1 | |  | | | | | |  |
|  | | |  |  |  |  |  |  |  | |  | | | | | | |
|  | | | | | | | | | | | | 1 |  | AT |  | 7/26/2020 9:18 AM |  |
|  | senior doctors prescribe nurses give health education about chemotherapy side effects then also the counsellors they also talk about side effects | | | | | | | | | | | | | | | |  |
|  |  | | | | | | | | | | | | | | | |  |
|  | | | **Files\\IDI_ _ Nurse_UCI_04** | | | | | | | | | | | | | |  |
|  |  |  | No |  |  |  | 0.0077 |  | 1 | |  | | | | | |  |
|  | | |  |  |  |  |  |  |  | |  | | | | | | |
|  | | | | | | | | | | | | 1 |  | AT |  | 7/26/2020 10:18 AM |  |
|  | Yes, like the patient says am not able to take chemotherapy, we cannot force the patient to take chemotherapy because we know that chemotherapy is one of the treatments to treat the patient or it’s the only treatment available in the given condition. | | | | | | | | | | | | | | | |  |
|  |  | | | | | | | | | | | | | | | |  |
|  | | | **Files\\IDI__ Doctor_ UCI_ 05** | | | | | | | | | | | | | |  |
|  |  |  | No |  |  |  | 0.0336 |  | 3 | |  | | | | | |  |
|  | | |  |  |  |  |  |  |  | |  | | | | | | |
|  | | | | | | | | | | | | 1 |  | AT |  | 7/26/2020 10:31 AM |  |
|  | However, the counsellors as well really play a very big role and in specific relation patients who have breast cancer before they start chemotherapy the counsellor interfaces with the patient. | | | | | | | | | | | | | | | |  |
|  |  | | | | | | | | | | | | | | | |  |
|  | | | | | | | | | | | | 2 |  | AT |  | 7/26/2020 10:32 AM |  |
|  | All breast cancer patients before they start chemotherapy, the counsellor interfaces with them. And I am saying that because I mainly see breast cancer patients currently and am very sure all breast cancer patients before they start chemotherapy the counsellor interfaces with them and the counsellor adds on to what the senior doctor tells them but also tries to find out the social situation of the patient before they start because we really receive quite a number of patients who have social issues which may hinder the treatment of cancer. | | | | | | | | | | | | | | | |  |
|  |  | | | | | | | | | | | | | | | |  |
|  | | | | | | | | | | | | 3 |  | AT |  | 7/26/2020 11:06 AM |  |
|  | In some forms you can get some cure but that depends on one thesome time this patient has but also how the patient responds to the chemotherapy, these tumors are different, some one may have a low grade tumor, a tumor which really grows very slowly then there are those with very aggressive tumors which are fast but all of them are stage three, are they all responding the same way, may be not. Secondly there are tumors which we call triple negative, those are very aggressive very pure tumors, they respond not so quiet well as patients who have tumors which may be responsive. | | | | | | | | | | | | | | | |  |
|  |  | | | | | | | | | | | | | | | |  |
|  | | | | | | | | | | | | | | | | | |
|  | | | | | | | | | | | | | | | | | |
| Reports\\Coding Summary By Code Report | | | | | | | | | | Page 87 of 117 | | | | | | | |
| 8/15/2023 8:10 AM | | | | | | | | | | | | | | | | | |
|  | | | **Aggregate** |  | **Classification** |  | **Coverage** |  | **Number Of Coding References** | |  | **Reference Number** |  | **Coded By Initials** |  | **Modified On** |  |
|  | **Nodes\\Information health providers share with Patients with terminal cancer\Communicate prognosis** | | | | | | | | | | | | | | | |  |
|  | | **Document** | | | | | | | | | | | | | | |  |
|  | | | **Files\\IDI - - Doctor- UCI -08** | | | | | | | | | | | | | |  |
|  |  |  | No |  |  |  | 0.0219 |  | 2 | |  | | | | | |  |
|  | | |  |  |  |  |  |  |  | |  | | | | | | |
|  | | | | | | | | | | | | 1 |  | AT |  | 7/24/2020 7:51 AM |  |
|  | Now the ideal is the most senior person is supposed to be the one to communicate like here it is supposed to be Dr. Ddungu if he is not there, it is supposed to be Dr. Okello if he is not there then Namaganda or me or the nurses but usually the decisions about prognosis are made during major ward rounds for patients who are inside but for patients who are outside it is the seniors. | | | | | | | | | | | | | | | |  |
|  |  | | | | | | | | | | | | | | | |  |
|  | | | | | | | | | | | | 2 |  | AT |  | 7/24/2020 7:52 AM |  |
|  | They all give because even the general doctors see a patient and know this is really stage four and whatever we may not do so much but you try to make the seniors also see so that there is some consensus on what you’re doing on a particular patient. | | | | | | | | | | | | | | | |  |
|  |  | | | | | | | | | | | | | | | |  |
|  | | | **Files\\IDI -Specialist palliative care_10** | | | | | | | | | | | | | |  |
|  |  |  | No |  |  |  | 0.093 |  | 2 | |  | | | | | |  |
|  | | |  |  |  |  |  |  |  | |  | | | | | | |
|  | | | | | | | | | | | | 1 |  | AT |  | 7/24/2020 9:31 AM |  |
|  | No, it is not always usual that when people communicate the prognosis unless the patient has really pushed you and prompted you to ask about the prognosis not even us even palliative care will do it commonly on the first visit. | | | | | | | | | | | | | | | |  |
|  |  | | | | | | | | | | | | | | | |  |
|  | | | | | | | | | | | | 2 |  | AT |  | 7/24/2020 9:32 AM |  |
|  | Prognosis it more the doctors but for us the palliative care people even the specialist nurses can give the prognosis. | | | | | | | | | | | | | | | |  |
|  |  | | | | | | | | | | | | | | | |  |
|  | | | **Files\\IDI_ Nurse_UCI_07** | | | | | | | | | | | | | |  |
|  |  |  | No |  |  |  | 0.0174 |  | 3 | |  | | | | | |  |
|  | | |  |  |  |  |  |  |  | |  | | | | | | |
|  | | | | | | | | | | | | 1 |  | AT |  | 7/26/2020 8:16 AM |  |
|  | The prognosis is usually by the medical doctors, the physicians, the consultants they usually give that information first however the nurse or the counsellor has to emphasize. These ones do the emphasis. | | | | | | | | | | | | | | | |  |
|  |  | | | | | | | | | | | | | | | |  |
|  | | | | | | | | | | | | 2 |  | AT |  | 7/26/2020 8:16 AM |  |
|  | Yes, about the prognosis. Initially it’s the medical doctors and after the medical doctors the patient is supposed to go to the nurse or the nurse counsellor for more counselling so they emphasize. | | | | | | | | | | | | | | | |  |
|  |  | | | | | | | | | | | | | | | |  |
|  | | | | | | | | | | | | 3 |  | AT |  | 7/26/2020 8:21 AM |  |
|  | To me I usually get these patients in the consultant’s room, so I am assuming the person who clerked these patients has given them information about their disease or their prognosis, however when they come to the consultant’s room… | | | | | | | | | | | | | | | |  |
|  |  | | | | | | | | | | | | | | | |  |
|  | | | **Files\\IDI_ Nurse_UCI_02** | | | | | | | | | | | | | |  |
|  |  |  | No |  |  |  | 0.0401 |  | 4 | |  | | | | | |  |
|  | | |  |  |  |  |  |  |  | |  | | | | | | |
|  | | | | | | | | | | | | 1 |  | AT |  | 7/26/2020 9:16 AM |  |
|  | Now from the first contact they see the junior doctors from there they see the senior doctors. The senior doctor ideally is supposed to disclose the prognosis of the disease. That according to this you’re on this stage. According to this stage you’re on this prognosis. The senior doctors are the ones who disclose the prognosis. | | | | | | | | | | | | | | | |  |
|  |  | | | | | | | | | | | | | | | |  |
| Reports\\Coding Summary By Code Report | | | | | | | | | | Page 88 of 117 | | | | | | | |
| 8/15/2023 8:10 AM | | | | | | | | | | | | | | | | | |
|  | | | **Aggregate** |  | **Classification** |  | **Coverage** |  | **Number Of Coding References** | |  | **Reference Number** |  | **Coded By Initials** |  | **Modified On** |  |
|  | | | | | | | | | | | | | | | | | |
|  | | | | | | | | | | | | 2 |  | AT |  | 7/26/2020 9:20 AM |  |
|  | I think it is 50’50 because you will find some will come and they will tell them their prognosis is done at the beginning that is if the stage is alarming but you can come in an early stage so people think you’re aiming at cure but when something is detoriating then they will start disclosing to you, then there are those ones…… | | | | | | | | | | | | | | | |  |
|  |  | | | | | | | | | | | | | | | |  |
|  | | | | | | | | | | | | 3 |  | AT |  | 7/26/2020 9:20 AM |  |
|  | They will tell you in the early stage you are but the prognosis because most of the times it is not really effective, every patient who comes in must not know their prognosis. From what I see maybe they get to know their prognosis maybe when it is the last stage, they are referring you to the palliative care team then they have to sit you that you know what the prognosis of this disease is not really good. I don’t think we are very effective there although we try. | | | | | | | | | | | | | | | |  |
|  |  | | | | | | | | | | | | | | | |  |
|  | | | | | | | | | | | | 4 |  | AT |  | 7/26/2020 9:20 AM |  |
|  | That is why I was telling you sometimes it depends on the stage, when you come to me and your stage is 4, I have to tell you my dear really your prognosis is not good but we are going to try. Me am thinking there are those ones who come early so people are thinking these ones we are aiming cure. | | | | | | | | | | | | | | | |  |
|  |  | | | | | | | | | | | | | | | |  |
|  | | | **Files\\IDI_ _ Nurse_UCI_04** | | | | | | | | | | | | | |  |
|  |  |  | No |  |  |  | 0.0341 |  | 3 | |  | | | | | |  |
|  | | |  |  |  |  |  |  |  | |  | | | | | | |
|  | | | | | | | | | | | | 1 |  | AT |  | 7/26/2020 9:55 AM |  |
|  | The prognosis is not very clearly brought out at the first time before they do the investigations, its important they have to first do these other investigations then now you’re able to get to know what stage of cancer you’re dealing with and that’s when you’re able to state what would be the prognosis of this patient. | | | | | | | | | | | | | | | |  |
|  |  | | | | | | | | | | | | | | | |  |
|  | | | | | | | | | | | | 2 |  | AT |  | 7/26/2020 9:57 AM |  |
|  | Both of them can actually give the prognosis of the disease depending on the interface, it can be the initial interface or it can be in the middle of the treatment so at any interface both of them can actually give that information of the prognosis. | | | | | | | | | | | | | | | |  |
|  |  | | | | | | | | | | | | | | | |  |
|  | | | | | | | | | | | | 3 |  | AT |  | 7/26/2020 9:59 AM |  |
|  | The disclosure of the patient’s diagnosis and prognosis, often it is something that is on but sometimes we have few cases were by people have not been explained about their prognosis and state of the disease and still tend to ask what is prognosis of my disease, what is the stage of my disease, it is a practice that is existing, people tend to do it but we have those few cases that actually still come out and say I don’t know about our state here, what is the stage of the disease, what do we have so we always tend to get those cases. | | | | | | | | | | | | | | | |  |
|  |  | | | | | | | | | | | | | | | |  |
|  | | | **Files\\IDI__ Doctor_ UCI_ 05** | | | | | | | | | | | | | |  |
|  |  |  | No |  |  |  | 0.0197 |  | 1 | |  | | | | | |  |
|  | | |  |  |  |  |  |  |  | |  | | | | | | |
|  | | | | | | | | | | | | 1 |  | AT |  | 7/26/2020 10:31 AM |  |
|  | No, all the stage really why I was saying stage 1 and stage 2, it means that the chances of cure are really high. Unlike a patient who has stage 3 or stage 4 so that’s about stage, then in terms of treatment or if I use the word management you knowing the stage has implications like I have just said, treatment also has implications so the treatment may be for cure or for palliation. I think I would prefer to use total palliation because when I just say palliation even stage 1 even stage2, these patients benefit from palliative care. And then prognosis is very important so prognosis meaning what are my chances I’m I going to be cured or I’m I not going to be cured now that’s all very important. So really in summary I think that’s what takes place on the first visit. | | | | | | | | | | | | | | | |  |
|  |  | | | | | | | | | | | | | | | |  |
|  | | | | | | | | | | | | | | | | | |
| Reports\\Coding Summary By Code Report | | | | | | | | | | Page 89 of 117 | | | | | | | |
| 8/15/2023 8:10 AM | | | | | | | | | | | | | | | | | |
|  | | | **Aggregate** |  | **Classification** |  | **Coverage** |  | **Number Of Coding References** | |  | **Reference Number** |  | **Coded By Initials** |  | **Modified On** |  |
|  | **Nodes\\Information health providers share with Patients with terminal cancer\Communicate treatment plan and outcome** | | | | | | | | | | | | | | | |  |
|  | | **Document** | | | | | | | | | | | | | | |  |
|  | | | **Files\\IDI - - Doctor- UCI -08** | | | | | | | | | | | | | |  |
|  |  |  | No |  |  |  | 0.0053 |  | 1 | |  | | | | | |  |
|  | | |  |  |  |  |  |  |  | |  | | | | | | |
|  | | | | | | | | | | | | 1 |  | AT |  | 7/24/2020 7:53 AM |  |
|  | You who is communicating to the patient about the treatment they are going to get , how are they going to get it, how many times are they going to get it, | | | | | | | | | | | | | | | |  |
|  |  | | | | | | | | | | | | | | | |  |
|  | | | **Files\\IDI - _ Doctor_UCI_06** | | | | | | | | | | | | | |  |
|  |  |  | No |  |  |  | 0.089 |  | 1 | |  | | | | | |  |
|  | | |  |  |  |  |  |  |  | |  | | | | | | |
|  | | | | | | | | | | | | 1 |  | AT |  | 7/24/2020 8:35 AM |  |
|  | All of us are involved in this aspect of care but the first visit is really by the clinicians or the doctors or the specialist really because it’s the specialist who does the examination and stages so principally in as far as diagnosis and staging is concerned and plan of treatment, it’s the doctor. | | | | | | | | | | | | | | | |  |
|  |  | | | | | | | | | | | | | | | |  |
|  | | | **Files\\IDI -Specialist palliative care_10** | | | | | | | | | | | | | |  |
|  |  |  | No |  |  |  | 0.0064 |  | 1 | |  | | | | | |  |
|  | | |  |  |  |  |  |  |  | |  | | | | | | |
|  | | | | | | | | | | | | 1 |  | AT |  | 7/24/2020 9:28 AM |  |
|  | However, if they don’t have enough information then it will be mainly the diagnosis and the tests that are required and in terms of palliative care if it is then what symptoms are going to be treated and how they are going to be treated. | | | | | | | | | | | | | | | |  |
|  |  | | | | | | | | | | | | | | | |  |
|  | **Nodes\\Information health providers share with Patients with terminal cancer\Emphasize diagnosis** | | | | | | | | | | | | | | | |  |
|  | | **Document** | | | | | | | | | | | | | | |  |
|  | | | **Files\\IDI - - Doctor- UCI -08** | | | | | | | | | | | | | |  |
|  |  |  | No |  |  |  | 0.0248 |  | 2 | |  | | | | | |  |
|  | | |  |  |  |  |  |  |  | |  | | | | | | |
|  | | | | | | | | | | | | 1 |  | AT |  | 7/24/2020 7:48 AM |  |
|  | Those who have clerked like one or two patients, they may spend more time talking to the patient telling them what the treatment is the stage is of the disease probably but if you have very many people in the line then the time to spend on them also reduces so information given to them usually is about the disease, diagnosis and the type of the cancer, | | | | | | | | | | | | | | | |  |
|  |  | | | | | | | | | | | | | | | |  |
|  | | | | | | | | | | | | 2 |  | AT |  | 7/24/2020 7:49 AM |  |
|  | where it is realized from and then whether they think this will really confirm the diagnosis of cancer or not because sometimes they come and they are saying he has cancer of the lymph nodes and there are many types but when you take history, examine then you see it is a different kind of cancer then you send them for biopsy because most of that is not constant. | | | | | | | | | | | | | | | |  |
|  |  | | | | | | | | | | | | | | | |  |
| Reports\\Coding Summary By Code Report | | | | | | | | | | Page 90 of 117 | | | | | | | |
| 8/15/2023 8:10 AM | | | | | | | | | | | | | | | | | |
|  | | | **Aggregate** |  | **Classification** |  | **Coverage** |  | **Number Of Coding References** | |  | **Reference Number** |  | **Coded By Initials** |  | **Modified On** |  |
|  | | | **Files\\IDI - _ Doctor_UCI_06** | | | | | | | | | | | | | |  |
|  |  |  | No |  |  |  | 0.0231 |  | 2 | |  | | | | | |  |
|  | | |  |  |  |  |  |  |  | |  | | | | | | |
|  | | | | | | | | | | | | 1 |  | AT |  | 7/24/2020 8:35 AM |  |
|  | Then we follow them up, each time they come to the clinic they see someone but it doesn’t follow like this, sometimes we may not go through all the steps but at least the minimum that they are told is the diagnosis that it is cancer, you give them the stage and tell them what treatment they are going to get. | | | | | | | | | | | | | | | |  |
|  |  | | | | | | | | | | | | | | | |  |
|  | | | | | | | | | | | | 2 |  | AT |  | 7/24/2020 8:36 AM |  |
|  | diagnosis and stage its doctors, its always that we need to be organized like that, if a patient asked me that’s they are fearful about starting treatment, I would also talk to them about mental health aspect of the disease. So sometimes it cuts across that a good number of times the first doctor sometimes goes through all of those but I must also add that because of the high numbers we may not go so deep as may have wanted to but at least we mention something. | | | | | | | | | | | | | | | |  |
|  |  | | | | | | | | | | | | | | | |  |
|  | | | **Files\\IDI -Specialist palliative care_10** | | | | | | | | | | | | | |  |
|  |  |  | No |  |  |  | 0.0113 |  | 1 | |  | | | | | |  |
|  | | |  |  |  |  |  |  |  | |  | | | | | | |
|  | | | | | | | | | | | | 1 |  | AT |  | 7/24/2020 9:28 AM |  |
|  | Some doctors will tell the patients their diagnosis and also tell them what further tests are needed and from there I think that is mostly what is given unless already someone has the tests done and that’s why I said information varies based on how far the person has been investigated so if they have heard a lot of investigations then may be someone will tell them about the type of treatment they are going to be given. | | | | | | | | | | | | | | | |  |
|  |  | | | | | | | | | | | | | | | |  |
|  | | | **Files\\IDI_ Nurse_UCI_07** | | | | | | | | | | | | | |  |
|  |  |  | No |  |  |  | 0.0585 |  | 5 | |  | | | | | |  |
|  | | |  |  |  |  |  |  |  | |  | | | | | | |
|  | | | | | | | | | | | | 1 |  | AT |  | 7/26/2020 8:15 AM |  |
|  | When you say the first visit it is tricky because the first visit would be first time at the Uganda Cancer Institute right? Those are the first timers but also this first visit can be the senior’s review because that is where every thing starts so to me I think for the first timers who have just come to cancer institute they are told of their diagnosis and after they have been clerked they are given investigations to do ideally so when they come to the senior clinic that is when they have come with the results ready to be reviewed by the consultant so in the consultant’s room still the consultant emphasizes the diagnosis that is the information they give to these patients, they emphasize the diagnosis, the treatment plan and the outcome. And also the kind of information given to these patients, it varies from patient to patient, some patients really yearn for more information and others don’t so it depends on the individual. | | | | | | | | | | | | | | | |  |
|  |  |  |  |  |  |  |  |  |  |  |  |  |  |  |  |  |  |
|  |  | | | | | | | | | | | | | | | |  |
|  | | | | | | | | | | | | 2 |  | AT |  | 7/26/2020 8:15 AM |  |
|  | For those individuals who have more questions they are given more information compared to these ones who do not have many questions reason being when a consultant has a long line they usually tend to look at the long line not the number of patients as an individual so an individual benefits best if they ask more questions but if you don’t have questions and there is a long line you will definitely go out with less information so that varies. | | | | | | | | | | | | | | | |  |
|  |  | | | | | | | | | | | | | | | |  |
|  | | | | | | | | | | | | 3 |  | AT |  | 7/26/2020 8:16 AM |  |
|  | No, every individual who asks there is always time to give them answers how ever long the line is, the consultants have to always given these patients time and they always do incase an individual asks questions. | | | | | | | | | | | | | | | |  |
|  |  | | | | | | | | | | | | | | | |  |
|  | | | | | | | | | | | | 4 |  | AT |  | 7/26/2020 8:22 AM |  |
|  | So inside the consultants room, patients are told of their stage, it is their stage mainly and also emphasizing the kind of cancer they have because you see at first you might not take it in very well that you have cancer but in the consultants room, the consultant takes the initiative to explain to you the stage on to which the cancer is, that is one. | | | | | | | | | | | | | | | |  |
|  |  | | | | | | | | | | | | | | | |  |
|  | | | | | | | | | | | | 5 |  | AT |  | 7/26/2020 8:23 AM |  |
|  | And also to plan for the treatment so as the consultant is reviewing these patients they tell them of their diagnosis, their stage, their outcome and the prognosis in the consultants room. | | | | | | | | | | | | | | | |  |
|  |  | | | | | | | | | | | | | | | |  |
| Reports\\Coding Summary By Code Report | | | | | | | | | | Page 91 of 117 | | | | | | | |
| 8/15/2023 8:10 AM | | | | | | | | | | | | | | | | | |
|  | | | **Aggregate** |  | **Classification** |  | **Coverage** |  | **Number Of Coding References** | |  | **Reference Number** |  | **Coded By Initials** |  | **Modified On** |  |
|  | | | **Files\\IDI_ Social worker_UCI_03** | | | | | | | | | | | | | |  |
|  |  |  | No |  |  |  | 0.0350 |  | 4 | |  | | | | | |  |
|  | | |  |  |  |  |  |  |  | |  | | | | | | |
|  | | | | | | | | | | | | 1 |  | AT |  | 7/26/2020 8:32 AM |  |
|  | They are not informed about their disease but they are only told that they have cancer either of the breast or maybe the cervix or maybe of the uterus, of the ovary or maybe the swelling that they have they say that they know the diagnosis that I have cancer of the what but they don’t understand the detail of it. | | | | | | | | | | | | | | | |  |
|  |  | | | | | | | | | | | | | | | |  |
|  | | | | | | | | | | | | 2 |  | AT |  | 7/26/2020 8:35 AM |  |
|  | I think the juniors as well if they can interpret the test results and clearly explain to the patients the level of the disease the stage if the disease, how | | | | | | | | | | | | | | | |  |
|  |  | | | | | | | | | | | | | | | |  |
|  | | | | | | | | | | | | 3 |  | AT |  | 7/26/2020 8:38 AM |  |
|  | What I have witnessed once not with adults but with children there will give a time when all the results are out then they will sit them down the care giver and the patient then they explain to them what the result is then they will explain to them the drug that they are going to start with the patient and then they will tell them. They will ask them if they have any question and usually tell them they will not know the outcome of the treatment, it may heal or it may not heal, that is the information that I have at least seen the doctor explaining to the patient. | | | | | | | | | | | | | | | |  |
|  |  | | | | | | | | | | | | | | | |  |
|  | | | | | | | | | | | | 4 |  | AT |  | 7/26/2020 8:38 AM |  |
|  | What they always tell us is that the doctor told me my disease like if I am to give an example of a teacher from Arua she said the doctor told me I have breast cancer that he is going to put me on treatment for about four cycles and then they are going to reveal it if it will need surgery or if it will not need surgery and they say that it is after the four cycles that is when they are going to reveal her so that is where the decision stops. | | | | | | | | | | | | | | | |  |
|  |  | | | | | | | | | | | | | | | |  |
|  | | | **Files\\IDI_ Nurse_UCI_02** | | | | | | | | | | | | | |  |
|  |  |  | No |  |  |  | 0.0077 |  | 2 | |  | | | | | |  |
|  | | |  |  |  |  |  |  |  | |  | | | | | | |
|  | | | | | | | | | | | | 1 |  | AT |  | 7/26/2020 9:16 AM |  |
|  | Disclosing to them what type of cancer they have, it is supposed to be doctors to disclose to them then also nurses sometimes do and the counsellor | | | | | | | | | | | | | | | |  |
|  |  | | | | | | | | | | | | | | | |  |
|  | | | | | | | | | | | | 2 |  | AT |  | 7/26/2020 9:19 AM |  |
|  | And you found them in the middle, well if you really have time you can sit her down and tell her that is if you found out. | | | | | | | | | | | | | | | |  |
|  |  | | | | | | | | | | | | | | | |  |
|  | | | **Files\\IDI_ _ Nurse_UCI_04** | | | | | | | | | | | | | |  |
|  |  |  | No |  |  |  | 0.0447 |  | 4 | |  | | | | | |  |
|  | | |  |  |  |  |  |  |  | |  | | | | | | |
|  | | | | | | | | | | | | 1 |  | AT |  | 7/26/2020 9:53 AM |  |
|  | Usually whenever patients come at the cancer institute at their first time, information which is given to them is about the condition that they have and that they are suffering from. | | | | | | | | | | | | | | | |  |
|  |  | | | | | | | | | | | | | | | |  |
|  | | | | | | | | | | | | 2 |  | AT |  | 7/26/2020 9:54 AM |  |
|  | Usually the challenge is that we have got the majority of the patients who come here they come when they don’t know what has exactly brought them to the cancer institute so they are asking but what exactly do I have so we tell them about their disease and after we tell them about their disease that’s when now we open up a file for them and in the process of opening the file then the file will be sent to one of the clerking rooms whereby the patient will be initially clerked, preparing the patient to start, that will be the entry to prepare the patient for care. | | | | | | | | | | | | | | | |  |
|  |  | | | | | | | | | | | | | | | |  |
|  | | | | | | | | | | | | 3 |  | AT |  | 7/26/2020 9:55 AM |  |
|  | Usually we tell them about what condition exactly they have and its mainly what their diagnosis, its mainly about the diagnosis. At the first sight they may not tell them exactly what is the extent of the disease we need to work them up and it gets to tell us what would be their stage of the disease. | | | | | | | | | | | | | | | |  |
|  |  | | | | | | | | | | | | | | | |  |
| Reports\\Coding Summary By Code Report | | | | | | | | | | Page 92 of 117 | | | | | | | |
| 8/15/2023 8:10 AM | | | | | | | | | | | | | | | | | |
|  | | | **Aggregate** |  | **Classification** |  | **Coverage** |  | **Number Of Coding References** | |  | **Reference Number** |  | **Coded By Initials** |  | **Modified On** |  |
|  | | | | | | | | | | | | | | | | | |
|  | | | | | | | | | | | | 4 |  | AT |  | 7/26/2020 9:57 AM |  |
|  | Its mainly the clinicians that are actually responsible to give the patients information about his or her disease, mainly the doctors, the nurses. Its starts with the doctors then the nurses then eventually inform them so mainly the doctors and the nurses are the ones who are responsible for giving the information on the patient’s diagnosis. The senior doctors will be able to give in that information. | | | | | | | | | | | | | | | |  |
|  |  | | | | | | | | | | | | | | | |  |
|  | | | **Files\\IDI__ Doctor_ UCI_ 05** | | | | | | | | | | | | | |  |
|  |  |  | No |  |  |  | 0.0297 |  | 2 | |  | | | | | |  |
|  | | |  |  |  |  |  |  |  | |  | | | | | | |
|  | | | | | | | | | | | | 1 |  | AT |  | 7/26/2020 10:30 AM |  |
|  | Then of course the other thing I have realized is that most patients may not know the diagnosis they have and usually prefer starting with what have you been told what you’re suffering from because close to 90% of the patients we have with diagnosis of cancer, it’s only a few where we have to reevaluate them and then try to confirm the diagnosis and of course in a way we take history on the first visit and we also do a physical exam which is detailed and its targeted as well and then we make a diagnosis of cancer which means that we have to stage the patient so by the time a patient steps out of my room he or she must be knowing what the stage is and the stage also has implications. Because if it is stage1 or stage 2 that means the chances of cure are really high and if it is stage 3 or stage 4 then palliation may be one of the options and by palliation, I mean total palliation. | | | | | | | | | | | | | | | |  |
|  |  |  |  |  |  |  |  |  |  |  |  |  |  |  |  |  |  |
|  |  | | | | | | | | | | | | | | | |  |
|  | | | | | | | | | | | | 2 |  | AT |  | 7/26/2020 10:42 AM |  |
|  | You can decide but however that also has challenges in our settings because the senior doctor assists this patient when they are starting and then sees the when they are finishing so the people who continue to see the patient are meant to reinforce to know this information. | | | | | | | | | | | | | | | |  |
|  |  | | | | | | | | | | | | | | | |  |
|  | **Nodes\\Information health providers share with Patients with terminal cancer\Explain treatment procedures such as investigations, further laboratory tests required.** | | | | | | | | | | | | | | | |  |
|  | | **Document** | | | | | | | | | | | | | | |  |
|  | | | **Files\\IDI - - Doctor- UCI -08** | | | | | | | | | | | | | |  |
|  |  |  | No |  |  |  | 0.0182 |  | 1 | |  | | | | | |  |
|  | | |  |  |  |  |  |  |  | |  | | | | | | |
|  | | | | | | | | | | | | 1 |  | AT |  | 7/24/2020 7:52 AM |  |
|  | The reason is depending on how these patients preserve or restrict information, how they react to information, their health because am treating you we give this and see how you react, if I don’t have the time to sit and really explain to you then its better I tell the counsellor who may have more time to sit with them that with patient we are doing this and that then they can do but usually we try to talk to the family if we cant talk to the patient directly but of course enforcing for the counsellor to talk to the family. | | | | | | | | | | | | | | | |  |
|  |  | | | | | | | | | | | | | | | |  |
|  | | | **Files\\IDI - _ Doctor_UCI_06** | | | | | | | | | | | | | |  |
|  |  |  | No |  |  |  | 0.0165 |  | 2 | |  | | | | | |  |
|  | | |  |  |  |  |  |  |  | |  | | | | | | |
|  | | | | | | | | | | | | 1 |  | AT |  | 7/24/2020 8:34 AM |  |
|  | For those who are not told the contents of their histology report, that one is a little tougher because most times they don’t know they just come to the cancer institute and you have it to them that it is cancer again you walk them through those steps of the history, knowing the stage then you send them for some investigation and we also tell them what treatment we are going to give. | | | | | | | | | | | | | | | |  |
|  |  | | | | | | | | | | | | | | | |  |
|  | | | | | | | | | | | | 2 |  | AT |  | 7/24/2020 8:47 AM |  |
|  | That is true but when the patient is going to get radiotherapy or chemotherapy that is definitive cancer, screening is different, they will clearly be told that you.. | | | | | | | | | | | | | | | |  |
|  |  | | | | | | | | | | | | | | | |  |
| Reports\\Coding Summary By Code Report | | | | | | | | | | Page 93 of 117 | | | | | | | |
| 8/15/2023 8:10 AM | | | | | | | | | | | | | | | | | |
|  | | | **Aggregate** |  | **Classification** |  | **Coverage** |  | **Number Of Coding References** | |  | **Reference Number** |  | **Coded By Initials** |  | **Modified On** |  |
|  | | | **Files\\IDI -Specialist palliative care_10** | | | | | | | | | | | | | |  |
|  |  |  | No |  |  |  | 0.0056 |  | 1 | |  | | | | | |  |
|  | | |  |  |  |  |  |  |  | |  | | | | | | |
|  | | | | | | | | | | | | 1 |  | AT |  | 7/24/2020 9:27 AM |  |
|  | its mainly to talk about which tests to be done and why the tests are going to be done and sometimes yes it is also true because many times when they come to cancer institute, they already have their diagnosis | | | | | | | | | | | | | | | |  |
|  |  | | | | | | | | | | | | | | | |  |
|  | | | **Files\\IDI- - Health Educator- UCI-09** | | | | | | | | | | | | | |  |
|  |  |  | No |  |  |  | 0.0130 |  | 3 | |  | | | | | |  |
|  | | |  |  |  |  |  |  |  | |  | | | | | | |
|  | | | | | | | | | | | | 1 |  | AT |  | 7/24/2020 12:35 PM |  |
|  | The second is the nurse to guide this patient on, now that the doctor has said you have this type of cancer, the doctor has requested for several tests, this nurse must explain the purpose of those tests, why should the patient do them, when they should do them and when they should come back when all these tests have been done so the third visit is now to see the senior doctor who is going to put this patient on treatment basing on the type of cancer, the stage of cancer and the patient’s condition. | | | | | | | | | | | | | | | |  |
|  |  | | | | | | | | | | | | | | | |  |
|  | | | | | | | | | | | | 2 |  | AT |  | 7/24/2020 12:36 PM |  |
|  | That is when the staging takes place because those tests are supposed to stage and inform the senior doctors to arrive at a point where if the patient needs treatment, which treatment will benefit this patient. | | | | | | | | | | | | | | | |  |
|  |  | | | | | | | | | | | | | | | |  |
|  | | | | | | | | | | | | 3 |  | AT |  | 7/24/2020 12:37 PM |  |
|  | Now the doctor is supposed to generate a treatment plan, after the treatment plan, this nurse who is going to treat this patient should explain what that treatment plan looks like, what the patient should do. | | | | | | | | | | | | | | | |  |
|  |  | | | | | | | | | | | | | | | |  |
|  | | | **Files\\IDI_ Counselor_UCI_01** | | | | | | | | | | | | | |  |
|  |  |  | No |  |  |  | 0.0115 |  | 1 | |  | | | | | |  |
|  | | |  |  |  |  |  |  |  | |  | | | | | | |
|  | | | | | | | | | | | | 1 |  | AT |  | 7/25/2020 10:47 PM |  |
|  | And others I think I always tell them what to expect I usually give them a quick navigation about how we work the work system, the steps we take so I always tell them today don’t expect to get cancer treatment because for the first you will clubbed then the doctors will need other things other investigations | | | | | | | | | | | | | | | |  |
|  |  | | | | | | | | | | | | | | | |  |
|  | | | **Files\\IDI_ Nurse_UCI_07** | | | | | | | | | | | | | |  |
|  |  |  | No |  |  |  | 0.0447 |  | 5 | |  | | | | | |  |
|  | | |  |  |  |  |  |  |  | |  | | | | | | |
|  | | | | | | | | | | | | 1 |  | AT |  | 7/26/2020 8:17 AM |  |
|  | The treatment plan inside the consultant’s the consultant hints on the treatment plan say like you’re going to be getting your treatment like for three weeks or every after this period of time however from the consultants room | | | | | | | | | | | | | | | |  |
|  |  | | | | | | | | | | | | | | | |  |
|  | | | | | | | | | | | | 2 |  | AT |  | 7/26/2020 8:20 AM |  |
|  | you emphasize, your treatment is every after this period of time and the date for your next doze is this because we usually write a lab request form for them so that lab request form usually has a date for the next treatment. | | | | | | | | | | | | | | | |  |
|  |  | | | | | | | | | | | | | | | |  |
|  | | | | | | | | | | | | 3 |  | AT |  | 7/26/2020 8:20 AM |  |
|  | Patient’s processes are usually cumbersome when you’re a new patient because you have very many investigations to do so usually after clerking they come with their investigation papers so from there when they come to book for an appointment it’s the nurse at the reception, we call it a booking table who explains to these patients what is done where because we have the x-ray department, lab , heart institute so it is the nurse at that table that gives an explanation of those patient processes. | | | | | | | | | | | | | | | |  |
|  |  | | | | | | | | | | | | | | | |  |
| Reports\\Coding Summary By Code Report | | | | | | | | | | Page 94 of 117 | | | | | | | |
| 8/15/2023 8:10 AM | | | | | | | | | | | | | | | | | |
|  | | | **Aggregate** |  | **Classification** |  | **Coverage** |  | **Number Of Coding References** | |  | **Reference Number** |  | **Coded By Initials** |  | **Modified On** |  |
|  | | | | | | | | | | | | | | | | | |
|  | | | | | | | | | | | | 4 |  | AT |  | 7/26/2020 8:20 AM |  |
|  | However, after we have initiated their treatment, it’s the nurse in the treatment room to explain to the patient what to do and when they are going to come back. So, we tell them they are supposed to have a CBC check and then we tell them where they do the CBC from. | | | | | | | | | | | | | | | |  |
|  |  | | | | | | | | | | | | | | | |  |
|  | | | | | | | | | | | | 5 |  | AT |  | 7/26/2020 8:22 AM |  |
|  | You see, what happens in the clerking room is, the patients come with a referral or they have their Histopathology results that’s it. So, after they open up a file for them, they go for clerking to intergret them in to the Uganda cancer institute’s system. After hat they are given investigations, those investigations help the consultant to kind of stage their disease to also look at the best treatment plan for them. | | | | | | | | | | | | | | | |  |
|  |  | | | | | | | | | | | | | | | |  |
|  | | | **Files\\IDI_ Social worker_UCI_03** | | | | | | | | | | | | | |  |
|  |  |  | No |  |  |  | 0.0514 |  | 5 | |  | | | | | |  |
|  | | |  |  |  |  |  |  |  | |  | | | | | | |
|  | | | | | | | | | | | | 1 |  | AT |  | 7/26/2020 8:31 AM |  |
|  | Usually for those that come to us because for us social workers we start from what the patient knows so when we ask them why have been referred to us, then they say because I am going to start my drug or I am supposed to do investigation and they said I am going to take three I said we usually start by asking them the reason why they were referred to us so they will tell us either they have told them to do investigation and they are coming from or they are starting treatment and they are asking them to find a nearby place so that they cannot miss their treatment. | | | | | | | | | | | | | | | |  |
|  |  | | | | | | | | | | | | | | | |  |
|  | | | | | | | | | | | | 2 |  | AT |  | 7/26/2020 8:36 AM |  |
|  | One because the doctors have a lot of patients to see, if we can have a nurse who gives a patient directives to take the next step and before they start their chemo, the nurse will be in a better position to explain, the doctor has seen you visit, this is what he has written down, have you understood this then from there you say ok you’re going to do ABCD test then when we do this test we will check and then you start on the drug, the drug is going to be for these days. | | | | | | | | | | | | | | | |  |
|  |  | | | | | | | | | | | | | | | |  |
|  | | | | | | | | | | | | 3 |  | AT |  | 7/26/2020 8:47 AM |  |
|  | The care takers when they come to us first time after they have known then based on the information, they already know then they will explain further to them. Like sometimes they come at an advanced stage then they come straight away for radiation and something like that. Then will also try and explain to them what he doctors by stage 4, stage 4 means they are only going to use radiation and this chemo but the possibility of disease healing quickly or healing is limited so now you need to do is to her the love, the care she needs the good food, be available to her to talk to her | | | | | | | | | | | | | | | |  |
|  |  | | | | | | | | | | | | | | | |  |
|  | | | | | | | | | | | | 4 |  | AT |  | 7/26/2020 9:09 AM |  |
|  | Ensuring that the patients do their tests at the required time with in the period requested because sometimes the patient is like CT scanned. | | | | | | | | | | | | | | | |  |
|  |  | | | | | | | | | | | | | | | |  |
|  | | | | | | | | | | | | 5 |  | AT |  | 7/26/2020 9:10 AM |  |
|  | As I said sometimes the patients fail to do other tests so they start them on the drug so the patients are ones to decide now, are these results going to affect the patient on continuation or we still need this to continue. So, the other bit is that I think what should be done here is also early staging because if you have done all the check up and stage the patient, you’re ready to give detailed information. | | | | | | | | | | | | | | | |  |
|  |  | | | | | | | | | | | | | | | |  |
|  | | | **Files\\IDI_ Nurse_UCI_02** | | | | | | | | | | | | | |  |
|  |  |  | No |  |  |  | 0.0336 |  | 3 | |  | | | | | |  |
|  | | |  |  |  |  |  |  |  | |  | | | | | | |
|  | | | | | | | | | | | | 1 |  | AT |  | 7/26/2020 9:14 AM |  |
|  | I don’t think the first contact doctor they disclose so much to them about the disease the information and then the prognosis like they have asked me. Until when they get that much information but what I know they don’t get to know about the disease because you can even ask them what cancer are treating, for me they told me it is in the abdomen when they don’t know the cancer they ae treating but they will know they have to get treatment at a certain time to treat that cancer of which they tell them we are going to do these investigations then they take to the doctor to write for you treatment so they know that they have to first have to do these investigations then they do get the treatment. But I don’t think they get to know more of the prognosis from the first contact of the first doctor. | | | | | | | | | | | | | | | |  |
|  |  | | | | | | | | | | | | | | | |  |
|  | | | | | | | | | | | | 2 |  | AT |  | 7/26/2020 9:16 AM |  |
|  | it is also done by the senior doctors because they are the ones who write then they tell them you’re going to take this treatment for six months or after three weeks. | | | | | | | | | | | | | | | |  |
|  |  | | | | | | | | | | | | | | | |  |
| Reports\\Coding Summary By Code Report | | | | | | | | | | Page 95 of 117 | | | | | | | |
| 8/15/2023 8:10 AM | | | | | | | | | | | | | | | | | |
|  | | | **Aggregate** |  | **Classification** |  | **Coverage** |  | **Number Of Coding References** | |  | **Reference Number** |  | **Coded By Initials** |  | **Modified On** |  |
|  | | | | | | | | | | | | | | | | | |
|  | | | | | | | | | | | | 3 |  | AT |  | 7/26/2020 9:19 AM |  |
|  | I think all of us for example, Doctor will tell the patient I expect to do these investigations, me also as a nurse I will tell the patient I expect you to be here on time at least at this time to get this drug, all of us. | | | | | | | | | | | | | | | |  |
|  |  | | | | | | | | | | | | | | | |  |
|  | | | **Files\\IDI_ _ Nurse_UCI_04** | | | | | | | | | | | | | |  |
|  |  |  | No |  |  |  | 0.0062 |  | 1 | |  | | | | | |  |
|  | | |  |  |  |  |  |  |  | |  | | | | | | |
|  | | | | | | | | | | | | 1 |  | AT |  | 7/26/2020 9:55 AM |  |
|  | Now that treatment will be initiated on the second visit when the patient is coming to visit the senior doctor that is when the treatment plan eventually comes out or on finishing those investigations. | | | | | | | | | | | | | | | |  |
|  |  | | | | | | | | | | | | | | | |  |
|  | **Nodes\\Information health providers share with Patients with terminal cancer\Handle economic effect, resolve family conflicts related to property ownership** | | | | | | | | | | | | | | | |  |
|  | | **Document** | | | | | | | | | | | | | | |  |
|  | | | **Files\\IDI - _ Doctor_UCI_06** | | | | | | | | | | | | | |  |
|  |  |  | No |  |  |  | 0.0159 |  | 1 | |  | | | | | |  |
|  | | |  |  |  |  |  |  |  | |  | | | | | | |
|  | | | | | | | | | | | | 1 |  | AT |  | 7/24/2020 9:07 AM |  |
|  | Honestly I have not paid much attention to that but there are some domestic tensions that come on when the patient has been diagnosed, some families it has come out in the open, people fighting for property, people wanting their share of things before the patient dies, people advising the patient to sell their property with in the family so the patient will open up and tell oh my wife, my children, mu husband want to sell my property that sort of thing arises. But generally, we don’t go in to those aspects of the patient’s life. | | | | | | | | | | | | | | | |  |
|  |  | | | | | | | | | | | | | | | |  |
|  | | | **Files\\IDI -Specialist palliative care_10** | | | | | | | | | | | | | |  |
|  |  |  | No |  |  |  | 0.0391 |  | 3 | |  | | | | | |  |
|  | | |  |  |  |  |  |  |  | |  | | | | | | |
|  | | | | | | | | | | | | 1 |  | AT |  | 7/24/2020 10:11 AM |  |
|  | There is a group there, the paralegal group, it was trained by the palliative care association of Uganda sometimes they come in they are not an established group into cancer institute but they can come into cancer care for help especially where you find patients has problems, has a lot of property and they are beginning to see issues in the family, wrangles that may come in but its not established into the system like when you’re outsourcing of course that’s what a social worker has to do to get other resources from else where to help the patients. | | | | | | | | | | | | | | | |  |
|  |  | | | | | | | | | | | | | | | |  |
|  | | | | | | | | | | | | 2 |  | AT |  | 7/24/2020 10:11 AM |  |
|  | Yes, they are many patients who have issues like I’m going to start treatment or I want to sell on my land, sell on my property so there are many of those ones and we talk to them and discuss about the advantages and benefits, sometimes especially if the chemo may not be life saving and whether they should consider that but of course its not enough support I should agree, its because that may need stronger discussion bringing the whole family to discuss that given the resources we have the discussion are not enough. | | | | | | | | | | | | | | | |  |
|  |  | | | | | | | | | | | | | | | |  |
|  | | | | | | | | | | | | 3 |  | AT |  | 7/24/2020 10:11 AM |  |
|  | Of course, they are there sometimes you receive them when it is too late when they have sold everything, they just say now I have sold everything am tired. And their families are left poor. Sometimes its lack of information although there are some patients who want to try until the end sometimes it is because they know that the treatment is not really going to save them. | | | | | | | | | | | | | | | |  |
|  |  | | | | | | | | | | | | | | | |  |
| Reports\\Coding Summary By Code Report | | | | | | | | | | Page 96 of 117 | | | | | | | |
| 8/15/2023 8:10 AM | | | | | | | | | | | | | | | | | |
|  | | | **Aggregate** |  | **Classification** |  | **Coverage** |  | **Number Of Coding References** | |  | **Reference Number** |  | **Coded By Initials** |  | **Modified On** |  |
|  | | | **Files\\IDI- - Health Educator- UCI-09** | | | | | | | | | | | | | |  |
|  |  |  | No |  |  |  | 0.0169 |  | 1 | |  | | | | | |  |
|  | | |  |  |  |  |  |  |  | |  | | | | | | |
|  | | | | | | | | | | | | 1 |  | AT |  | 7/25/2020 10:34 PM |  |
|  | In aspects f disclosure, we must explore the capacity of this person to sustain the decision lets say from the treatment perspectives we must prepare this person and preparation means assessing whether this person has the capacity where economic interventions required. Look here, today we have cancer medicine tomorrow we have it, the third day you don’t have it, can this person buy it? So that is a decision that must be discussed before someone begins treatment and if someone needs to mobilize resources before beginning treatment all this comes from disclosure. If someone didn’t know that you’re going to these stages, at this stage you need money lets say CT scan is paid for about ne fifty thousand depending on the size so if we say you have cancer, you have been told you have cancer we need to tell you, now that you have cancer you will go through some tests and examinations, others need money so you do not just walk in to get a trap that we need money so could figure out having to fund his money at this level roughly this will happen so informed decisions on economic aspects is totally lacking. We push them in to where they didn’t know, they didn’t suspect that there will be money. | | | | | | | | | | | | | | | |  |
|  |  |  |  |  |  |  |  |  |  |  |  |  |  |  |  |  |  |
|  |  | | | | | | | | | | | | | | | |  |
|  | | | **Files\\IDI__ Doctor_ UCI_ 05** | | | | | | | | | | | | | |  |
|  |  |  | No |  |  |  | 0.0316 |  | 2 | |  | | | | | |  |
|  | | |  |  |  |  |  |  |  | |  | | | | | | |
|  | | | | | | | | | | | | 1 |  | AT |  | 7/26/2020 10:57 AM |  |
|  | Economically actually it’s a very big challenge, I think cancer care is the most expensive medical form of treatment, we are a bit fortunate that most of the cancer treatments are free of charge and I’m going to say most not all, there are those which are really expensive that patients can’t afford but I also wont miss the challenge of stock outs because we are a government institution and we are the only cancer center so we receive so many patients from the country but also out of the country so sometimes we are faced with stock outs and the patient has to buy their drugs and this really impacts on their economic well being but also not forgetting since most patients come from up country transport but also feeding when they are here because these treatments some are weekly, some two weekly, some are monthly so you know that’s a very big challenge on transport, feeding, people are meant to work so its really a challenge. | | | | | | | | | | | | | | | |  |
|  |  |  |  |  |  |  |  |  |  |  |  |  |  |  |  |  |  |
|  |  | | | | | | | | | | | | | | | |  |
|  | | | | | | | | | | | | 2 |  | AT |  | 7/26/2020 10:57 AM |  |
|  | Absolutely, we do counsel them and now we have partners who help us with accommodation to help those patients who come from especially up country, some stay in hostels near by and the are ferried here because it’s a reality and we are also trying to see whether we can set up regional cancer centers as well. | | | | | | | | | | | | | | | |  |
|  |  | | | | | | | | | | | | | | | |  |
|  | **Nodes\\Information health providers share with Patients with terminal cancer\Initial information related to cancer disease** | | | | | | | | | | | | | | | |  |
|  | | **Document** | | | | | | | | | | | | | | |  |
|  | | | **Files\\IDI - - Doctor- UCI -08** | | | | | | | | | | | | | |  |
|  |  |  | No |  |  |  | 0.0872 |  | 7 | |  | | | | | |  |
|  | | |  |  |  |  |  |  |  | |  | | | | | | |
|  | | | | | | | | | | | | 1 |  | AT |  | 7/24/2020 7:49 AM |  |
|  | they usually direct them say okay now you’ve come to cancer institute, you have cancer, there are these tests you have to do then you come back you need to book then this so what the institute expects of them is communicated to them to a bigger extent | | | | | | | | | | | | | | | |  |
|  |  | | | | | | | | | | | | | | | |  |
|  | | | | | | | | | | | | 2 |  | AT |  | 7/24/2020 7:50 AM |  |
|  | but what a patient is to expect because some of them ask do you think I will get cured or not so it is given to them in bits not in totality and they may be many so sometimes some will ask do you think I will cure so the person is so anxious so you’re also curious like should I give everything I think about this disease to this now or I give in parts then I give now then the next time they come I give more like that. | | | | | | | | | | | | | | | |  |
|  |  | | | | | | | | | | | | | | | |  |
| Reports\\Coding Summary By Code Report | | | | | | | | | | Page 97 of 117 | | | | | | | |
| 8/15/2023 8:10 AM | | | | | | | | | | | | | | | | | |
|  | | | **Aggregate** |  | **Classification** |  | **Coverage** |  | **Number Of Coding References** | |  | **Reference Number** |  | **Coded By Initials** |  | **Modified On** |  |
|  | | | | | | | | | | | | | | | | | |
|  | | | | | | | | | | | | 3 |  | AT |  | 7/24/2020 7:50 AM |  |
|  | All cadres of staff give information to patients currently about disease beginning from say the highest person to the lowest because patients ask any one but like officially it is the doctors, nurses, the counsellors more I would say the clinical staff. | | | | | | | | | | | | | | | |  |
|  |  | | | | | | | | | | | | | | | |  |
|  | | | | | | | | | | | | 4 |  | AT |  | 7/24/2020 7:51 AM |  |
|  | For now most of the time when we are seeing patients we tell them now we are the ones treating you and we are the doctors and nurses so this is what we want you to do, we try to stress the important things they need to know but of course we cannot rule out completely that people may give wrong information , there is yet to know the strict measures given when controlling those things so I wouldn’t say there is really a direct control but what we usually do we stress what we are going to do and as the doctors treating the patient what they need to know and what they need to do. So in most cases they tend to hear what the doctors and nurses say but the reason I say all cadres give information , a patient may come and is seated there, you are still doing a ward round the patients are also there, patients attendants are there so that is still challenging. | | | | | | | | | | | | | | | |  |
|  |  |  |  |  |  |  |  |  |  |  |  |  |  |  |  |  |  |
|  |  | | | | | | | | | | | | | | | |  |
|  | | | | | | | | | | | | 5 |  | AT |  | 7/24/2020 7:52 AM |  |
|  | It is the senior who deal with them. And then they are usually communicated to by the counsellors to counsel them but they still give the same thing and then the palliative care givers. | | | | | | | | | | | | | | | |  |
|  |  | | | | | | | | | | | | | | | |  |
|  | | | | | | | | | | | | 6 |  | AT |  | 7/24/2020 8:02 AM |  |
|  | I can say your disease is stage four, we can treat you can improve which is true but I may not add the we will clear the disease completely from your body so at some point you still come back, I may not tell them that, | | | | | | | | | | | | | | | |  |
|  |  | | | | | | | | | | | | | | | |  |
|  | | | | | | | | | | | | 7 |  | AT |  | 7/24/2020 8:02 AM |  |
|  | Improving is not like curing because some of the masses keep swelling here and I keep giving drugs so they happen to disappear but there are still some cancer cells in your body but at least you have improved so it is usually giving one, when they are having a senior review and then there is a progression in the disease. | | | | | | | | | | | | | | | |  |
|  |  | | | | | | | | | | | | | | | |  |
|  | | | **Files\\IDI - _ Doctor_UCI_06** | | | | | | | | | | | | | |  |
|  |  |  | No |  |  |  | 0.0538 |  | 2 | |  | | | | | |  |
|  | | |  |  |  |  |  |  |  | |  | | | | | | |
|  | | | | | | | | | | | | 1 |  | AT |  | 7/24/2020 8:33 AM |  |
|  | Thank you very much. Most times patients that come to the Uganda cancer institute do have a histology report a good number of times so for those who have a histology report and clearly where the report says its cancer, we ask them whether they have been told the contents of that report. Many times they have been told its cancer also a number of times they do not know they are just told take this report to the cancer institute so for those who know that its cancer, we again tell them it is cancer of the cervix and basically what we do in the clinic is a take history and do an examination and then the purpose of examination is to stage to know what stage it is. 90% of our patients come with advanced cancer say of the cervix in my area of gynecology meaning that it is stage three and above so we talk to them about the stage, the prognosis and we are very clear with them that we are not curing them, we are giving treatment to control symptoms and improve their quality of life but there is a high chance that the disease is going to reoccur. | | | | | | | | | | | | | | | |  |
|  |  |  |  |  |  |  |  |  |  |  |  |  |  |  |  |  |  |
|  |  | | | | | | | | | | | | | | | |  |
|  | | | | | | | | | | | | 2 |  | AT |  | 7/24/2020 8:41 AM |  |
|  | But from my experience, most patients who come to the cancer institute because they present at advanced stages, they have been through so many clinics, they have treated and there is no improvement that alone where someone says go to the cancer institute, they begin preparing I think something is there. And also, sometimes the disease is advanced that some patients just want to leave in any way that you can so its easy to start a conversation with them not easy but its much better, you can tell them you have this cancer it’s at this stage and you need to give this treatment. Patients’ main question will be: will I feel better? And in most times, we have told them that we are not curing you, we are managing symptoms, they handle it remarkably well. | | | | | | | | | | | | | | | |  |
|  |  | | | | | | | | | | | | | | | |  |
|  | | | **Files\\IDI -Specialist palliative care_10** | | | | | | | | | | | | | |  |
|  |  |  | No |  |  |  | 0.081 |  | 1 | |  | | | | | |  |
|  | | |  |  |  |  |  |  |  | |  | | | | | | |
|  | | | | | | | | | | | | 1 |  | AT |  | 7/24/2020 9:27 AM |  |
|  | You know information varies really that is given to the patient, most of the time on the first visit people are finding out from the patient where the problem is and then most of the information given at that point if it is ever the first visit to cancer institute before they come back the second time | | | | | | | | | | | | | | | |  |
|  |  | | | | | | | | | | | | | | | |  |
| Reports\\Coding Summary By Code Report | | | | | | | | | | Page 98 of 117 | | | | | | | |
| 8/15/2023 8:10 AM | | | | | | | | | | | | | | | | | |
|  | | | **Aggregate** |  | **Classification** |  | **Coverage** |  | **Number Of Coding References** | |  | **Reference Number** |  | **Coded By Initials** |  | **Modified On** |  |
|  | | | **Files\\IDI- - Health Educator- UCI-09** | | | | | | | | | | | | | |  |
|  |  |  | No |  |  |  | 0.0113 |  | 3 | |  | | | | | |  |
|  | | |  |  |  |  |  |  |  | |  | | | | | | |
|  | | | | | | | | | | | | 1 |  | AT |  | 7/24/2020 10:29 AM |  |
|  | When you are here a patient expects to know the disease that they have, the patient wants to know what is the stage if at all he knows the stage. The patient has to know, is this cancer curable or not, a patient wants how he is going to be treated, what is his treatment like, how long will it take now all this information. | | | | | | | | | | | | | | | |  |
|  |  | | | | | | | | | | | | | | | |  |
|  | | | | | | | | | | | | 2 |  | AT |  | 7/24/2020 10:34 AM |  |
|  | The intention of the institute from planning, the institute as I said it, what I said is has this person got the treatment, they know this patient needs treatment. | | | | | | | | | | | | | | | |  |
|  |  | | | | | | | | | | | | | | | |  |
|  | | | | | | | | | | | | 3 |  | AT |  | 7/24/2020 12:37 PM |  |
|  | Right the nurse is supposed to be with the senior doctor by the time of making the decision on the treatment so if that one happens this nurse is supposed to carry this information and give detailed education to this patient because it is the nurse going to administer the treatment so at that point a lot is missed out. | | | | | | | | | | | | | | | |  |
|  |  | | | | | | | | | | | | | | | |  |
|  | **Nodes\\Information health providers share with Patients with terminal cancer\Palliative care entails and its importance( breaking bad news, offer supportive treatment and symptom control)** | | | | | | | | | | | | | | | |  |
|  | | **Document** | | | | | | | | | | | | | | |  |
|  | | | **Files\\IDI__ Doctor_ UCI_ 05** | | | | | | | | | | | | | |  |
|  |  |  | No |  |  |  | 0.0128 |  | 1 | |  | | | | | |  |
|  | | |  |  |  |  |  |  |  | |  | | | | | | |
|  | | | | | | | | | | | | 1 |  | AT |  | 7/26/2020 11:06 AM |  |
[truncated: 63,013 more chars]
