# Supplementary material for: Informed consent in cancer clinical care: Perspectives of healthcare professionals on information disclosure at a tertiary institution in Uganda
Source: PLoS One. 2024 Apr 4;19(4):e0301586. doi: 10.1371/journal.pone.0301586 (PMC10994281; doi:10.1371/journal.pone.0301586)
Supplement: S2 File — (DOCX) [file pone.0301586.s003.docx]

**Study Title**:

**Healthcare professional perspectives on information disclosure during the informed consent process for cancer care at Uganda Cancer Institute**

**Disclosure of information**

- - 1. What information is usually communicated to the patients and/or caregivers during the consenting process for care? **Prob**e for diagnosis, drug side effects, treatment options, treatment plan, prognosis etc.
    2. What other information do you routinely ask the patients?

**Informed consent practices**

- - 1. How is information on the patients’ disease /condition often communicated
    2. Are there any criteria or standard operating procedures to guide the communication of the diagnosis of cancer or sensitive prognostic information? If so, please describe the guidance you use. Probe for immediate disclosure, delayed disclosure, withholding of information etc.
    3. As you may know, most Ugandan, particularly those that come from upcountry, live in closely knit nuclear families. What is the role of the family in the consenting process? Probe for involvement of families in information disclosure, decision making, treatment planning etc.

**Challenges and recommendations to improve the informed consent process**

- - 1. Describe some of the challenges/barrier to optimal patient health education and communication of information during the consenting process for patients with advanced cancer.
    2. What suggestions/recommendations do you have for improving the consenting process for cancer clinical care.
    3. Is there anything you think is important to this topic, but we haven’t talked about? Or, do you have any question to ask me?
