# Supplementary material for: Informed consent in cancer clinical care: Perspectives of healthcare professionals on information disclosure at a tertiary institution in Uganda
Source: PLoS One. 2024 Apr 4;19(4):e0301586. doi: 10.1371/journal.pone.0301586 (PMC10994281; doi:10.1371/journal.pone.0301586)
Supplement: Attachment — Submitted filename: PLOS One review_11.21.23.docx [file pone.0301586.s004.docx]

Thank you for the opportunity to review this interesting and important paper.

Overall – primary concerns

- Need to clarify what is meant by ‘informed consent’ - this term has multiple interpretations and conceptualizations, depending on the context. It can mean a specific document related to a specific event (e.g., surgery or procedure) or it can relate to more general discussions between providers and patients regarding prognosis, treatment goals, and disclosure of diagnosis. In the paper, multiple aspects of ‘informed consent’ are discussed and what is meant by ‘information disclosure during the consenting process’ is unclear. It also seems based on the interview data participants interpreted this concept very differently as well.
- Inconsistency with quality of writing and organization; some portions of paper very well written and well organized, others much less so.
- Inappropriate qualitative methodology and concern regarding sampling strategy. Sample size concern – how was data saturation achieved given n=10 of such diverse participant perspectives and roles?

Abstract:

- Needs more detail and organization, especially regarding methods
  - Introduction – need to specify study took place in Uganda
  - Methods – more detail is needed (e.g, type of healthcare providers recruited; how were key informants selected; within same institution; what qualitative approach was used? How were interviews conducted?
  - Results – should be organized by key themes; barriers are referenced but not listed/described

Introduction:

- Need to place study within global context and acknowledge differing cultural norms related to ‘informed consent’
- Need to clarify how the proposed study address the gap in the existing literature
- Similarly, need to provide more citations supporting the need to look at informed consent processes in cancer care specifically

Methods:

- Sections disjointed and not well organized
- More detail regarding how key informants were selected and rationale for sampling strategy is needed
- Potential sampling issue – my experience in global health is that nurses are very rarely/if ever involved in ‘obtaining informed consent’ – although this is the eligibility criteria described under “Participants.” See comment below about being clear what is meant by ‘informed consent.’
- Include more detail about type of institution and rationale for study site in Methods – did not de-identify study site – seems problematic given small sample size
- Authors state they used a phenomenological approach to their qualitative inquiry, but this is not supported by their reported data collection and analysis methods. Phenomenology is a highly specialized and complex type of qualitative inquiry that deeply explores the ‘lived experience’ of human existence (such as grief), with in-depth interviews (often repeated interviews with same participants) grounded in the philosophical orientation of phenomenology and requires a high level of abstraction in data analysis. What is described in this paper is consistent with a qualitative descriptive approach, where the goal is to stay close to the data to answer more concrete and specific questions that have been identified *a priori* (see Sandelowski, PMID: 10940958)
- Interviews were all in English, but some were translated from local language?
- 141-142 – Why was the interview guide pilot tested on cancer patients?
- Data analysis – where is prior analytic framework? (referenced in lines 162-164)
- 166-167 – quotes should inform themes not vice versa
- 169-180 – feels out of place with unnecessary detail; could add a brief statement regarding reflexivity to Discussion or perhaps to Limitations

Results:

- Would help to describe participants and their roles more clearly to help contextualize the themes – e.g., what is meant by medical officer? What is the difference between nurse and nurse counselor? Is the palliative care specialist a physician or a nurse?
- Table 1 would be strengthened by including supporting quotes to justify how themes were developed
- Overall, Results section could be more concise and more clearly organized (some information is repeated – e.g., suggestions are repeated again under ‘barriers’)
- Consider a table that summarizes recommendations discussed by participants in interviews
- 439-443 – repeated quote
- Need to clarify the role the family plays in receiving the information and providing informed consent (e.g., 224 – 225)

Discussion:

- Some information in Discussion is repeated information from Results
- Discussion should focus on how findings differ or support prior work; how findings should be interpreted; how fills a gap in the literature (as it reads, it’s unclear the main contributions or innovation the paper makes to the current literature)
- Differences in responses by discipline/background could be significantly influenced by scope of practice, perceived roles, issues related to power/control, cultural norms, structural barriers related to communication – and critically how participant understood the concept of ‘informed consent.’ These factors are not adequately addressed and need more attention.
- 555 – 557 - “It is for this reason that **some authors** believe that physician led informed consent discussions are often ineffective because of the heavy workload and mechanistic way consent is obtained [41].” What do the authors mean by “some authors”? Why is there an implied discrepancy between authors?

Best practices:

- Would cut this section as does not feel relevant to this paper; reads as section of dissertation inserted into manuscript.

Minor point

- Term ‘opined’ is overused throughout paper and is a bit distracting
